# Diverse selection pressures shaping the genetic architecture of behçet disease susceptibility

**DOI:** 10.3389/fgene.2022.983646

**Published:** 2022-09-30

**Authors:** Efe Sezgin, Elif Kaplan

**Affiliations:** ^1^ Department of Food Engineering, Izmir Institute of Technology, Izmir, Turkey; ^2^ Biotechnology Interdisciplinary Program, Izmir Institute of Technology, Izmir, Turkey

**Keywords:** behcet disease, complex disease evolution, population genetics, population genomics, selection, ancestral allele, derived allele, population differentiation

## Abstract

Behçet disease (BD) is a polygenic, multifactorial, multisystem inflammatory condition with unknown etiology. Global distribution of BD is geographically structured, highest prevalence observed among East Asian, Middle Eastern, and Mediterranean populations. Although adaptive selection on a few BD susceptibility loci is speculated, a thorough evolutionary analysis on the genetic architecture of BD is lacking. We aimed to understand whether increased BD risk in the human populations with high prevalence is due to past selection on BD associated genes. We performed population genetics analyses with East Asian (high BD prevalence), European (low/very low BD prevalence), and African (very low/no BD prevalence) populations. Comparison of ancestral and derived alleles’ frequencies versus their reported susceptible or protective effect on BD showed both derived and ancestral alleles are associated with increased BD risk. Variants showing higher risk to and more significant association with BD had smaller allele frequency differences, and showed less population differentiation compared to variants that showed smaller risk and less significant association with BD. Results suggest BD alleles are not unique to East Asians but are also found in other world populations at appreciable frequencies, and argue against selection favoring these variants only in populations with high BD prevalence. BD associated gene analyses showed similar evolutionary histories driven by neutral processes for many genes or balancing selection for HLA (Human Leukocyte Antigen) genes in all three populations studied. However, nucleotide diversity in several HLA region genes was much higher in East Asians suggesting selection for high nucleotide and haplotype diversity in East Asians. Recent selective sweep for genes involved in antigen recognition, peptide processing, immune and cellular differentiation regulation was observed only in East Asians. We conclude that the evolutionary processes shaping the genetic diversity in BD risk genes are diverse, and elucidating the underlying specific selection mechanisms is complex. Several of the genes examined in this study are risk factors (such as *ERAP1*, *IL23R*, *HLA-G*) for other inflammatory diseases. Thus, our conclusions are not only limited to BD but may have broader implications for other inflammatory diseases.

## 1 Introduction

Behçet disease (BD) is a multisystem arterial and venous inflammatory condition with wide clinical spectrum manifestations including skin, eyes, kidneys, nervous and gastrointestinal systems (Dalvi et al., 2012; Hatemi et al., 2013). Pathogenic mechanisms underlying BD is not fully understood, but polygenic background and interaction with environmental factors are thought to be involved in the disease pathogenesis (Hatemi et al., 2013). BD is mostly seen in populations from East Asia to Mediterranean, and is very rare in Northern European, US, American Indian, Australian, and African populations (Keino and Okada, 2007; Leonardo and McNeil, 2015). Highest prevalence is observed among Mediterranean, Middle Eastern, and East Asian populations, therefore, referred as the so-called ‘Silk Road disease’ (Verity et al., 1999). HLA-B*5 was the first locus identified to be associated with BD based on studies with patients along the Silk Road (Ohno et al., 1982). Multiple follow-up studies confirmed HLA-B*51 as a major genetic risk factor for BD (Verity et al., 1999; Remmers et al., 2010; Kirino et al., 2013b; Gul, 2014). High HLA-B*51 frequency observed in Silk Road populations let to arguments for past pathogen or some other environment factor driven selection favoring HLA-B*51 in these populations, therefore, increased BD risk in contemporary populations is suggested to be a result of past selection on HLA-B alleles (Piga and Mathieu, 2014; Sazzini et al., 2015; Smith et al., 2021).

However, HLA-B is not the only locus associated with BD. Genome-wide association studies (GWAS) with different populations identified over 100 loci that influence BD risk ((Gul, 2014); see Supplementary Table S1 for a comprehensive list of references). Moreover, HLA-B*51 is also observed in populations other than Silk Road, where BD is not reported or with very low prevalence populations (Keino and Okada, 2007; Leonardo and McNeil, 2015). And, only around half of the patients are HLA-B*51 positive in populations with high BD prevalence (Verity et al., 1999). Therefore, a simple explanation for BD being result of a past selection on HLA-B alleles cannot capture the possibly more complicated evolutionary history of BD risk.

BD is not the only complex disease that shows population differentiation. Some human complex traits including common diseases such as cardiovascular diseases (Menotti et al., 1999; Chaturvedi, 2003), body fat percentage (Deurenberg et al., 2002), body mass index (Robinson et al., 2015), and height variation (Stulp and Barrett, 2016) show differentiation among worldwide populations. Whether these phenotypic differentiations have genetic components, and whether selection or neutral (demographic and drift) processes have been shaping the genetic differentiation underlying these phenotypes has drawn much attention. Selection studies using candidate gene approach focusing on brain size (Evans et al., 2005; Mekel-Bobrov et al., 2005), ability to digest lactose (Bersaglieri et al., 2004), Duffy blood group (Hamblin et al., 2002), and malaria resistance (Tishkoff et al., 2001) suggested natural selection acting on the examined genes. Moreover, genome-wide selection scan studies without focusing on any phenotype identified genome regions under selection overlapping with candidate gene studies and also novel ones such as malaria resistance (Sabeti et al., 2002), resistance to viral infection, skin pigmentation, ability to digest lactose, height, and hair follicle type (Voight et al., 2006; Sabeti et al., 2007; Field et al., 2016).

Whereas some of the selected genes are observed in the entire human lineage (Clark et al., 2003; Bustamante et al., 2005; Nielsen et al., 2005), others are population specific, observed in Africans (Stajich and Hahn, 2005; Voight et al., 2006), Asians (Carlson et al., 2005), and mostly Europeans (Kayser et al., 2003; Akey et al., 2004; Storz et al., 2004; Carlson et al., 2005) suggesting adaption to local environmental factors. Indeed, some of the identified regions have medical relevance (Di Rienzo and Hudson, 2005) such as type-2 diabetes (Vander Molen et al., 2005), salt sensitivity (Thompson et al., 2004), and increased fecundity (Stefansson et al., 2005).

In this study, we hypothesized that increased BD risk in modern human populations can be due to past selection on BD associated genes and their variants in regions with high disease prevalence. To test this hypothesis, we conducted population genetic and genomic analyses with BD associated genes and their variants. First, to test whether BD susceptibility is a derived selected trait, we compared the distribution of disease risk status (susceptible vs protective) of alleles with their respective ancestral and derived status among populations with high BD prevalence. Second, we conducted population differentiation analyses among different world populations using BD associated alleles and other variants that are not reported to be associated with BD, and compared the results. We analyzed the correlation between population differentiation and significance of reported disease association test statistics for all BD associated variants. Moreover, we compared the population differentiation of most significantly associated BD alleles with the overall differentiation of their respective genes to test whether selection is unique to the BD associated variant(s) or targets the gene as a whole. Third, we estimated nucleotide and haplotype diversity, and conducted selection tests in populations with high and low BD prevalence.

## 2 Materials and methods

### 2.1 Genes and variants associated with behçet disease

Genes and variants associated with Behcet’s disease were identified by review of articles found in Pubmed (https://www.ncbi.nlm.nih.gov/pubmed/; accessed and searched periodically till 31 May 2022) literature search with the keywords “Behcet disease”, “Behcet’s disease”, “Behcet syndrome”, and “genetics” covering years 1980–2022. Only original research articles written in English where sample size, investigated genes and their variants, and their statistical association with Behcet’s disease reported were further evaluated. In total seventy seven articles matched the search criteria. We further filtered out redundant information. In total, we used gene and SNP information from 18 publications (Supplementary Table S1). For genome-wide association studies, SNPs with reported *p*-values that reach genome-wide statistical significance is used. For candidate gene studies, only genetic associations that were replicated in at least two independent studies, and the statistical association result based on the largest sample size were included the final SNP list (Supplementary Table S1).

World population frequencies, position on the gene structure, nature of change (coding, non-coding, promoter, etc.), and allelic state (ancestral vs derived) information of BD associated variants were extracted from the dbSNP (Sherry et al., 2001) (https://www.ncbi.nlm.nih.gov/snp/; accessed latest on 31 May 2022), and the 1,000 Genomes (1K Genome) (http://www.internationalgenome.org/; accessed latest on 31 May 2022) (1000 Genomes Project Consortium, 2015) databases.

### 2.2 Samples for Single Nucleotide Polymorphism (SNP) based and gene sequence based population genetic analyses

For SNP based and gene sequence based population genetic analyses 1K Genome database (http://www.internationalgenome.org/) (1000 Genomes Project Consortium, 2015) populations was used. The 1K Genome populations used in this study are listed in Supplementary Table S3. Analyses performed in this study focused on East Asian (high BD prevalence), European (low/very low BD prevalence), and African (very low/no BD prevalence) populations. DNA sequence and VCF files for BD associated genes were downloaded via ENSEMBL DataSlicer tool (http://grch37.ensembl.org/Homo_sapiens/Tools/DataSlicer) based on Phase 31,000 Genomes data) (1000 Genomes Project Consortium, 2015). African, East Asian, and European samples consisted of 661, 504, and 503 individuals, respectively.

### 2.3 Population genetic and selection analyses

SNP based fixation index (F_st_) comparisons between 1K Genome populations were calculated using PLINK (a toolset for whole-genome association and population-based linkage analysis) versions 1.9 and 2.0 (www.cog-genomics.org/plink/2.0/) (Purcell et al., 2007; Chang et al., 2015). For gene based analyses, we calculated segregating sites (S), total number of mutations (Eta), number of haplotypes, haplotype diversity, nucleotide diversity *π* (pi) (Nei, 1987), average number of nucleotide differences *θ*_*K*_ (ThetaK) (Tajima, 1983), and Watterson theta *θ*_*W*_ (ThetaW) (Watterson, 1975; Nei, 1987) within each population.

The *θ* (Theta), calculated by *4N*_*e*_ *µ* (multiplication of effective population size and mutation rate), nucleotide diversity estimates are based on the number of segregating sites. The *π* diversity estimate is based on the average number of pairwise differences between gene sequences. For allele frequency spectrum based neutrality tests we estimated Tajima’s D Tajima (1989), Fu and Li’s D Fu and Li (1993), Fu and Li’s F Fu and Li (1993), Fu’s Fs Fu (1997), Achaz’s Y Achaz (2008), Ramos-Onsins and Rozas R2 Ramos-Onsins and Rozas (2002), and *ZnS* statistic (Kelly, 1997). The results of all tests are presented in Table 1.

**TABLE 1:** Comparison of ancestral and derived allele status versus population distribution of BD associated SNPs with respect to their effect on BD.

	Susceptible		Protective		P^a^
	Ancestral N(%)	Derived N(%)	Ancestral N(%)	Derived N(%)	
Chinese	18 (31)	14 (24)	13 (22)	14 (22)	0.64
Japanese	35 (24)	47 (33)	20 (14)	41 (29)	0.23
Korean	5 (62)	3 (38)	-	-	-
Turkish	14 (35)	24 (60)	0 (0)	2 (100)	0.29
					
Total^b^	72 (29)	88 (35)	33 (13)	57 (23)	0.32
SNPs^c^ with *p* < 10^−5^					
	32 (26)	53 (44)	15 (12)	21 (17)	0.68

a Chi-square test result.

b Pooling alleles and their effects from all reported studies.

c Focusing only on reported alleles from studies with larger sample size and more significant BD, association (reported *p* values less then *p* < 10^−5^). Populations represent study populations where the BD, genetic association study was conducted and variants were discovered.

Here we overview these approaches briefly. Tajima’s *D* statistically tests whether the *π* and theta estimates of nucleotide diversity are significantly different from each other. Under neutral evolutionary processes such as no selection and constant population size the *π* and *θ* estimates of nucleotide diversity should be very similar, resulting in Tajima’s *D* values around zero. Excessive rare polymorphisms lead to negative Tajima’s *D* values indicating negative selection, background selection or sudden population growth, whereas excessive intermediate frequency polymorphisms lead to positive Tajima’s *D* values suggesting balancing selection. Fu and Li’s D* and F* tests compare the distribution of mutations on the internal and external branches of the gene. Recently emerged rare frequency variants are found on the external braches, whereas older higher frequency variants are found on the internal branches of the tree. Therefore, negative Fu-Li test results suggest abundance of recent/new mutations, and positive values indicate abundance of old intermediate or high frequency variants. Fu’s Fs Fu (1997), Achaz’s Y Achaz (2008), and Ramos-Onsins and Rozas R2 Ramos-Onsins and Rozas (2002) can model the distribution of allele frequency specturm under different demographic and evolutionary models. They are much less affected by sample size and statistically more powerful than Tajima’s *D* and Fu and Li tests. *ZnS* statistic (Kelly, 1997) estimates linkage disequilibrium as standardized average linkage disequilibrium statistic between all pairs of segregating sites.

We estimated F_st_ (fixation index), D_xy_ (average number of nucleotide substitutions per site between two populations), and H_st_ (haplotype diversity based differentiation) (Hudson et al., 1992a; Hudson et al., 1992b) parameters for population differentiation. In our study, all population genetic parameters were estimated by DnaSP 6 (DNA Sequence Polymorphism) software (Rozas et al., 2017).

Detection of recent selection on BD associated genome regions is performed by iHS (intra-population linkage disequilibrium based Integrated haplotype score) (Voight et al., 2006), and XP-EHH (inter-population haplotype differentiation based Cross population extended haplotype homozygosity) (Sabeti et al., 2007) tests using PopHuman (Casillas et al., 2018). Pairwise XP-EHH analyses compares YRI (Yoruba in Ibadan, Nigeria), CEU (Utah Residents with Northern and Western European Ancestry), and CHB (Han Chinese in Bejing, China) populations, whereas iHS scores are calculated within AFR, EAS, EUR, and SAS subpopulations. The sliding window size is set to 10 kb for iHS and XP-EHH estimates. The highest calculated iHS and XP-EHH scores from the sliding window analyses for each gene are reported. The windows are not necessarily centered on the variants associated with BD in order not to lose selection signals from other SNPs in the gene.

Adaptive protein evolution parameter estimates using chimpanzee sequences included McDonald and Kreitman test (MKT) (McDonald and Kreitman, 1991), Neutrality index (NI) (Rand and Kann, 1996), Alpha value (proportion of adaptive substitutions) (Smith and Eyre-Walker, 2002), and Direction of selection (DoS) (Stoletzki and Eyre-Walker, 2011).

Recent selection and adaptive protein evolution tests were conducted using PopHuman (https://pophuman.uab.cat/) (Casillas et al., 2018) tool. PopHuman is a human genomics-oriented web browser based tool that can conduct the selection tests listed above.

### 2.4 Statistical analyses

Distribution of ancestral, derived, susceptible, and protective effect alleles among populations is compared by Chi-square tests. For continuous variables, deviations from Normal distribution is tested by Shapiro test. Population genetic parameter estimates per gene were compared among Asian, European, and African populations by non-parametric Kruskal–Wallis one-way ANOVA followed by non-parametric Wilcoxon pairwise tests. Principal component analysis with allele frequencies were conducted in R (https://www.r-project.org/), and visualized using the *factoextra* package version 1.0.7 (https://cran.r-project.org/web/packages/factoextra/index.html). Rank based regressions (Terpstra and McKean, 2005) were performed by *Rfit* package version 0.23.0 (Kloke and McKean, 2012). All statistical analyses were conducted in R (https://www.r-project.org/).

### 2.5 Molecular function and biological pathway analyses

Molecular function identification, biological process enrichment, protein class, and protein-protein interaction analyses were conducted using the PANTHER (Protein ANalysis THrough Evolutionary Relationships) (http://www.pantherdb.org/) (Mi and Thomas, 2009), and STRING-DB (Search Tool for the Retrieval of Interacting Genes/Proteins) (Szklarczyk et al., 2021) (https://stringdb.org) online tools. Protein-protein interaction network was constructed from primary interactions based on functional and physical protein associations only from curated databases and experimentally determined sources using the online STRING tool. Minimum required interaction score was set to medium confidence. Protein-protein interaction network was drawn using the Cytoscape tool (Shannon et al., 2003).

## 3 Results

### 3.1 Annotation of behçet disease associated variants

Considering both candidate gene and genome-wide association studies, 241 variants from 114 genes were identified to be associated with BD. For candidate gene studies, only genetic associations that were replicated in at least two independent studies, and the result based on the largest sample size were included in the final list (Supplementary Table S1). Only 9% of the BD associated variants were in the exons, and 64% of them were reported to increase BD risk. dbSNP identification (rs number) was possible for all but two of the variants. Gene ontology and pathway analyses of genes with respect to molecular function, biological process, and protein class showed common representation of immune response related genes involved in antigen processing and presentation (such as *HLA*), cytokine and chemokine signaling pathways (such as *IL10*, *CCR*) in the list. However, limited gene ontology information was found for most of the top hit BD genes such as *PSORS1C1*, and *CCHCR1* (Supplementary Table S2).

### 3.2 Analyses with behçet disease associated variants: Allele status, behçet disease association, and effect of population differentiation on behçet disease risk

**FIGURE 1:**
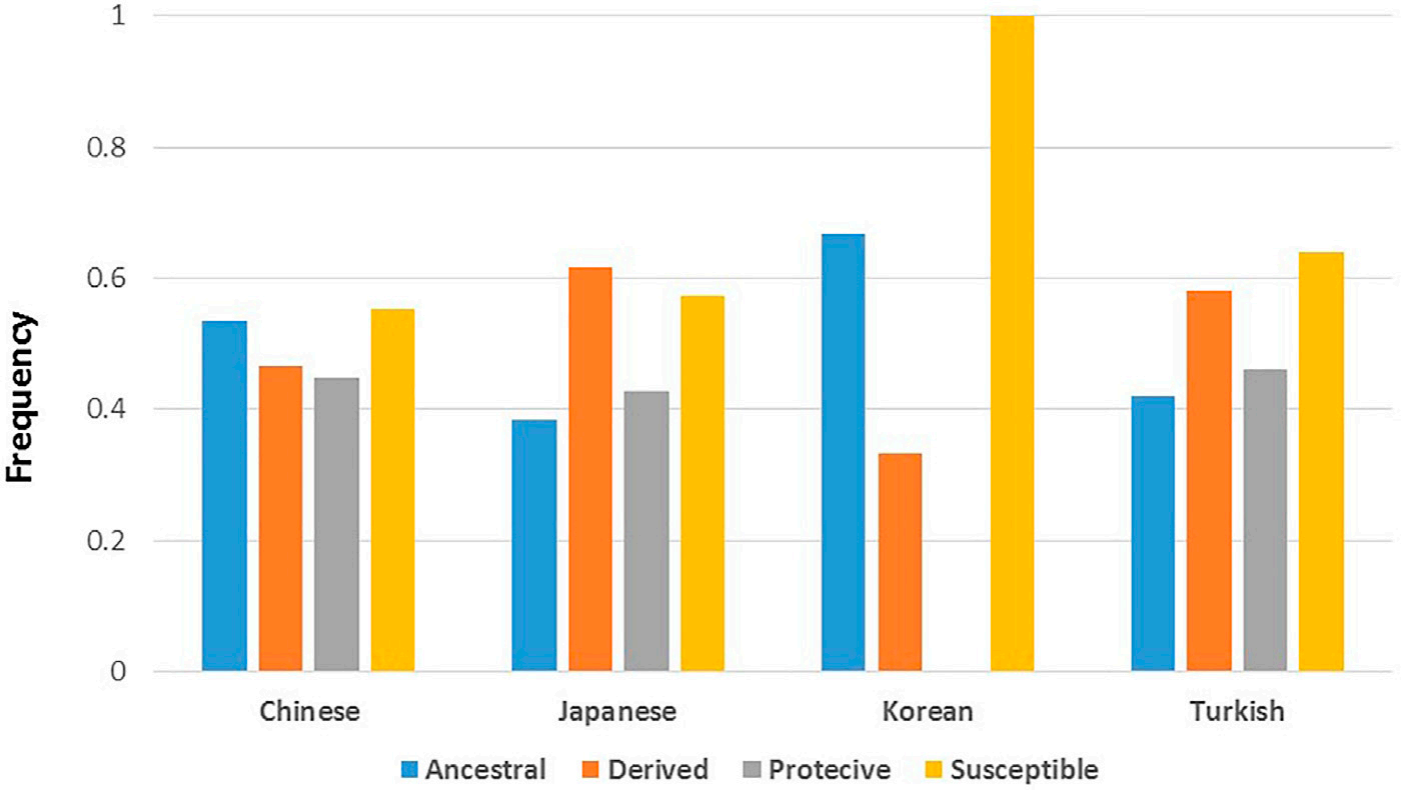
Distribution of BD associated variants’ ancestral and derived allele status, and their effect on BD among the populations with highest BD prevalence. Populations represent study populations where the BD genetic association study was conducted and variants were discovered. Allele count details can be seen in Table 1.

For analyses with BD associated variants, we focused on published results from studies conducted with Chinese, Japanese, Korean, and Turkish populations. The SNP/variant identification number, effect on BD (protective vs susceptible), and reported *p*-values are used to test whether susceptibility to BD is an ancestral or derived trait. Firstly, we identified the ancestral and derived allele status of BD associated variants, and compared the distribution of allele status with respect to their effect on BD. Overall, the percentage of derived alleles were slightly higher compared to ancestral alleles for both susceptible (35 vs 29%) and protective (23 vs 13%) variants but the difference was not statistically significant (Table 1). Pooling all variants from all studies, the distribution of ancestral and derived alleles with respect to their susceptible or protective effect on BD was not statistically significant (Table 1). Stratifying the analysis by study populations again did not suggest enrichment of ancestral or derived alleles within susceptible or protective phenotypes in any population (Table 1). Ancestral/derived status or susceptible/protective effect partitioning of BD variants were not significantly different between the populations (Figure 1).

**FIGURE 2:**
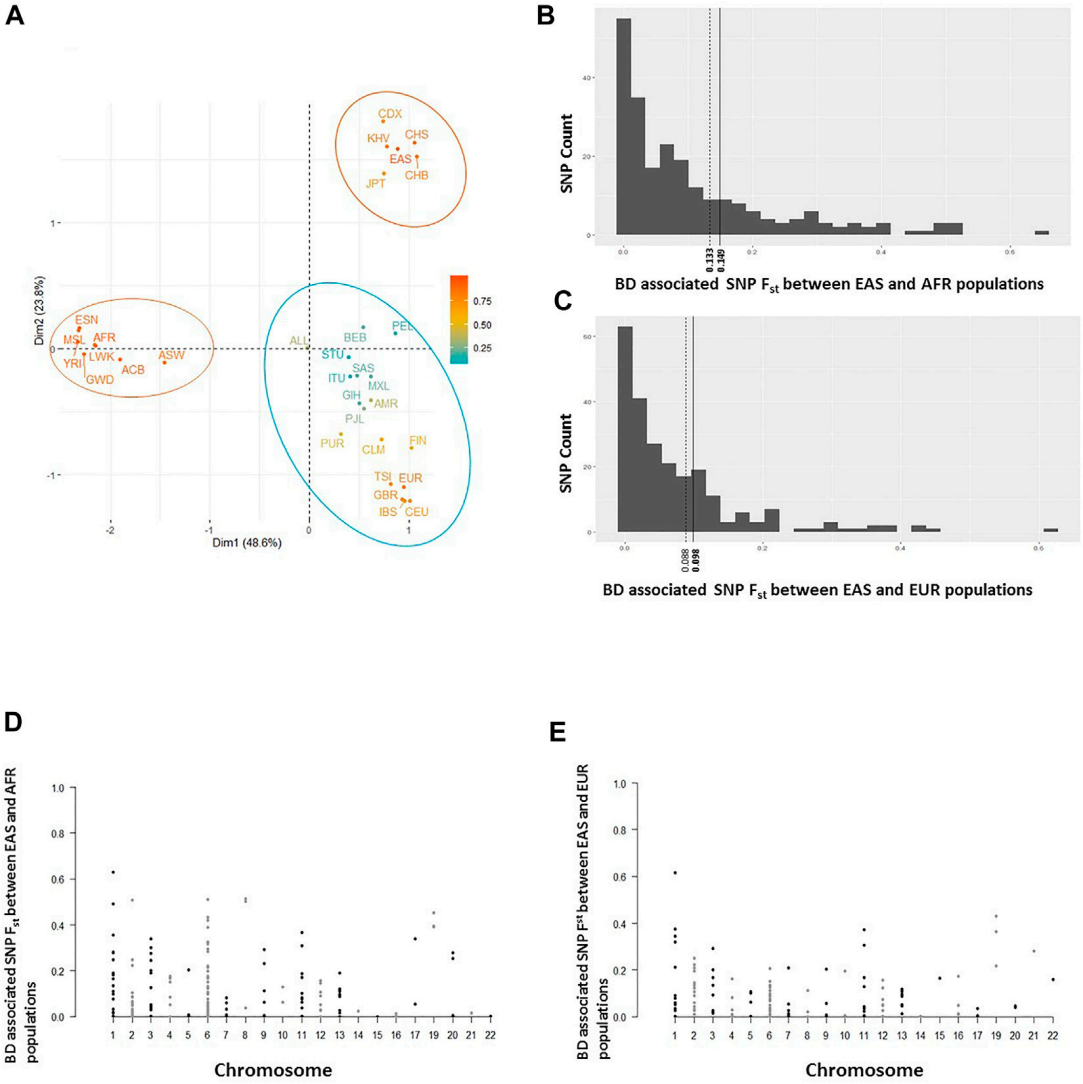
**(A)** Separation of all 26 1K Genome populations along the top 2 principal components (Dim1 and Dim2) based on principal component analyses conducted on a BD associated allele frequency matrix. Ellipses around populations in indicate clusters formed by clustering analysis. **(B)** Population differentiation (F_st_) estimates of BD associated SNPs between East Asian (EAS) and African (AFR) populations. Solid and dashed vertical lines shows genome-wide SNP F_st_ and mean F_st_ of BD associated SNPs, respectively. **(C)** F_st_ estimates of BD associated SNPs between East Asian (EAS) and European (EUR) populations. Solid and dashed vertical lines shows genome-wide SNP F_st_ and mean F_st_ of BD associated SNPs, respectively. **(D)** Distribution of BD associated SNPs’ EAS-AFR F_st_ values along human chromosomes. **(E)** Distribution of BD associated SNPs’ EAS-EUR F_st_ values along human chromosomes.

Allele frequencies of reported BD associated variants with rs numbers were retrieved for all twenty six 1,000 Genomes populations (Supplementary Table S3) and a principal component analysis was conducted on the constructed allele frequency matrix. Differentiation of populations along the most informative top two principal components (PC) was visualized (Figure 2A). The first PC clearly differentiated African populations from the rest of the world populations. BD associated variants in *IL23R, ASB18, SAMD3, EBF2, TNFAIP3, SMARCA2,* and *COL12A1* showed the highest loading on the first PC, indicating large frequency differences between African and other populations. Interestingly, the second PC clearly separated East Asian populations from the other populations including the Southeast Asians. BD associated variants *in IL10, STAT4, TENM4, LYST, LILRB1, DTL, API5, LTN1, FUT2*, *TNFAIP3*, and *PMFBP1* had the largest contributions on the second PC splitting East Asians from other populations. These variants were also more significantly associated with BD in East Asian populations compared to the variants with large contribution on the first PC, some such as *IL10* (rs1518111 and rs1800871), *TNFAIP3* (rs9494885), *API5* (rs16937370) being some of the most significant variants reported to be associated with BD (Supplementary Table S1).

In addition to principal component analyses, we investigated population differentiation (F_st_) between East Asian, African, and European populations. The mean F_st_ estimate of BD associated SNPs between East Asian and African (0.133), and East Asian and European (0.088) populations were lower compared to genome-wide F_st_ estimates between East Asian and African (0.149), and East Asian and European (0.098) populations (Figures 2B,C). Seventy four percent of the SNPs had lower F_st_ values compared to the mean F_st_ of all BD SNPs between East Asians and Africans (less than 0.133; Figure 2B), and 68 percent of the SNPs had lower F_st_ values compared to the mean F_st_ of all BD SNPs between East Asians and Europeans (less than 0.088; Figure 2C) suggesting small number of BD associated SNPs with large differentiation between East Asians and other populations. Distribution of BD SNPs with high and low F_st_ values was even throughout the human chromosomes (Figures 2D,E), indicating lack of confounding due to chromosomal stratification or genomic location. Moreover, population differentiation (F_st_) with respect to African populations was higher for the ancestral BD associated alleles compared to the derived BD associated alleles (medians 0.09 vs 0.06, *p* = 0.008) in East Asians (Supplementary Figure S1).

**FIGURE 3:**
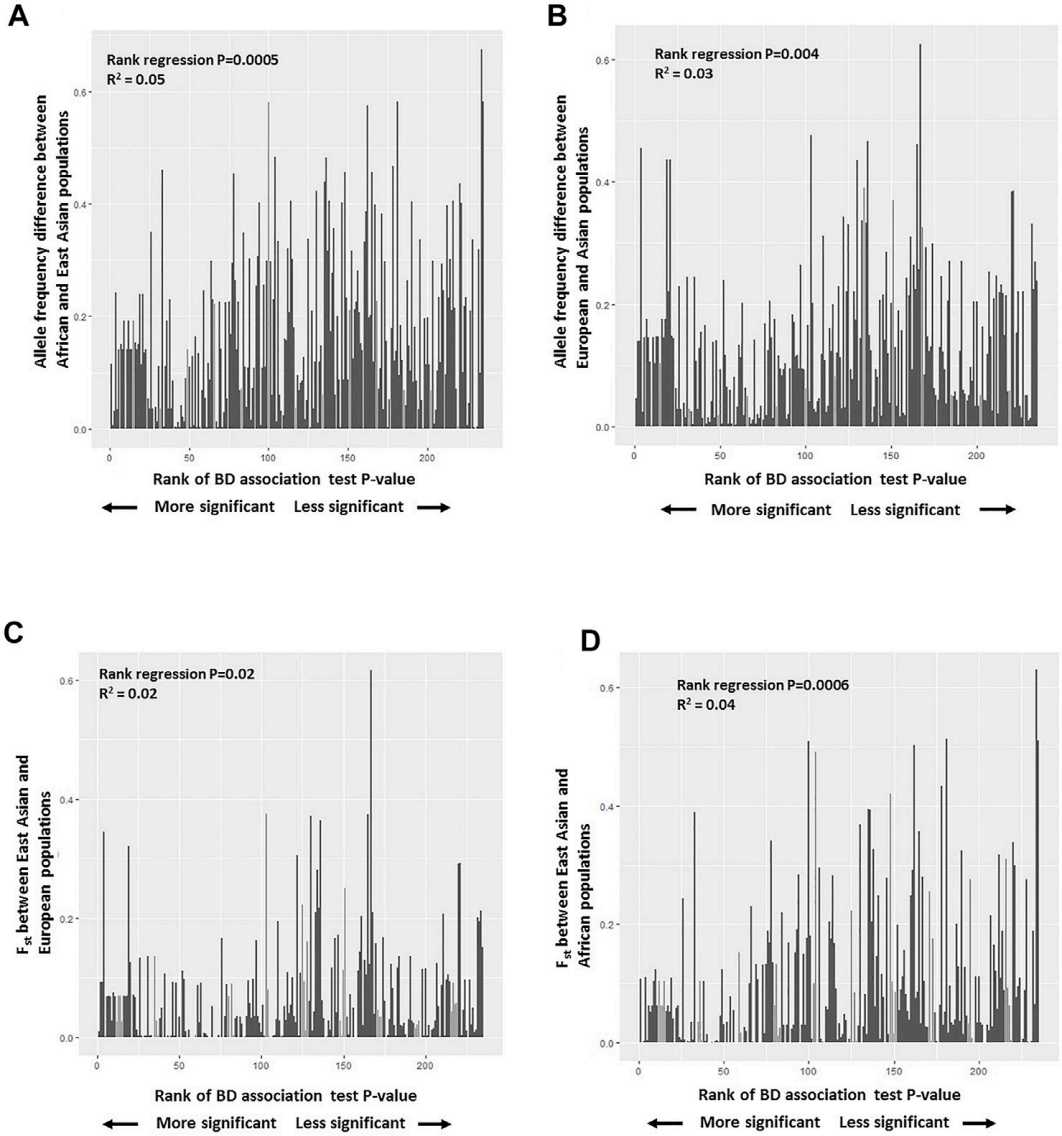
**(A)** Plot of BD associated variants’ allele frequency difference between African and East Asian populations versus the rank of BD association test *p*-values reported. **(B)** Plot of BD associated variants’ allele frequency difference between European and East Asian populations versus the rank of BD association test *p*-values reported. **(C)** Plot of population differentiation (F_st_) between East Asian, and European populations versus the rank of BD association test *p*-values reported. **(D)** Plot of population differentiation (F_st_) between East Asian, and African populations versus the rank of BD association test *p*-values reported.

Further examination of distributions of BD associated SNPs’ allele frequencies among East Asian, African, and European populations showed mostly overlapping histograms but different modalities (Supplementary Figure S2A). No enrichment or systematic skew with respect to rare or more frequent alleles was observed in any population. We hypothesized that BD associated variants with larger allele frequency differences and population differentiation (F*st*) between East Asians (high BD prevalence) and other populations (low/no BD prevalence) will be more significantly associated with BD and will have a larger risk effect on BD. To test this hypothesis, a rank regression analysis was conducted where the allele frequencies of BD associated SNPs in East Asians was regressed on the rank order of the reported *p*-values of these variants from BD GWAS studies with East Asian populations. No statistically significant trend was observed (Supplementary Figure S2B). However, when the same rank regression analysis was conducted regressing allele frequency differences between Africans and East Asians or Europeans and East Asians on the rank order of the reported *p*-values of these variants, significant trends were observed (Figures 3A,B). Overall there was less allele frequency difference between East Asians and Africans or East Asians and Europeans for variants reported to be more statistically significantly associated with BD (having a larger effect on BD risk). This trend was still significant if a regular regression was conducted regressing allele frequency differences between East Asians and Africans or East Asians and Europeans on the actual reported *p*-values of these variants (Supplementary Figure S3). Similarly, a significant rank regression trend was observed between East Asian-African F_st_ and East Asian-European F_st_ estimates, and the rank order of BD associated variant *p*-values. More significant BD associations were observed to have lower F_st_ values between East Asians and Africans or East Asians and Europeans (Figures 3C,D). When similar regression analyses were conducted with reported odds ratios, higher risk variants were observed to have less allele frequency differences and lower population differentiation between East Asians and Europeans, and East Asians and Africans (Supplementary Figure S4).

### 3.3 Population genetic analyses with behçet disease associated genes

#### 3.3.1 Intra-population statistics

Following analyses focusing on the specific BD associated variants, population genetic analyses examining the molecular evolutionary history of BD associated genes were conducted (Supplementary Tables S4─S6). Firstly, the cumulative distribution of summary statistics covering estimates of nucleotide and haplotype diversity, allele frequency spectrum, and population differentiation tests for 114 genes among African, East Asian, and European populations were compared (Table 2). African populations had the highest nucleotide and haplotype diversity followed by East Asian and European populations. Although African populations showed slightly more negative Tajima’s *D* test results, East Asian populations had significantly more negative Fu and Li’s D* and Fu and Li’s F* test results indicating higher number of more recent derived singleton or rare variants (Table 2, Supplementary Figure S5). The Y*, *R*_*2*_, and *ZnS* parameter estimates were similar in East Asian and European populations suggesting similar demographic and evolutionary histories (Table 2). The *ZnS* estimates were larger for East Asian and European populations compared to Africans suggesting larger linkage disequilibrium and extended haplotypes (Table 2).

**TABLE 2:** Comparison of population genetic parameter estimates of 114 BD associated genes among 1,000 Genomes African (AFR), East Asian (EAS), and European (EUR) populations.

Parameter	AFR Median (25%,75%)	EAS Median (25%,75%)	EUR Median (25%,75%)	*p*
**Nucleotide**	**Diversity**			
Total sites	903 (246, 3,695)	903 (246, 3,695)	903 (246, 3,695)	0.99
S	143 (15, 549)	113 (8, 400)	105 (11, 375)	0.05
Eta	553 (144, 1789)	400 (114, 1,074)	375 (106, 1,006)	0.05
Hap	482 (160, 1,056)a	313 (96, 877)b	278 (78, 819)b	0.004
H_d_	0.99 (0.95, 0.99)	0.97 (0.86, 0.99)	0.97 (0.88, 0.92)	0.08
Π	1.1 (0.8, 1.5)a	0.8 (0.6, 1.2)b	0.8 (0.6, 1.3)b	0.0002
θ_K_	43.7 (14.7, 131.1)	35.3 (9.4, 107.2)	35.8 (11.1, 100.5)	0.37
θ_W_	71.3 (18.5, 230.4)	53.4 (15.3, 143.4)	50.1 (14.1, 134.3)	0.08
**Allele Frequency Spectrum**				
Tajima’s *D*	−1.2 (−1.5, −0.9)a	−0.8 (−1.3, −0.3)b	−0.7 (−1.2, −0.1)b	<0.001
Fu and Li’s D*	−6.5 (−7.9, −4.3)a	−8.5 (−10.4, −4.0)b	−7.4 (−9.2, −4.0)a	0.0008
Fu and Li’s F*	−3.7 (−4.5, −2.6)a	−4.5 (−5.6, −2.6)b	−3.9 (−4.8, −2.8)a	0.001
Fu’s *F*_*s*_	−31.4 (−34.4, −30.2)	−30.9 (−32.0, −9.6)	−31.0 (−32.2, −7.9)	0.06
Achaz’s Y*	−0.7 (−1.2, −0.3)a	0.1 (−0.5, 0.7)b	0.2 (−0.4, 0.9)b	<0.001
*R*_*2*_	0.04 (0.03, 0.05)a	0.05 (0.04, 0.06)b	0.05 (0.04, 0.06)b	0.001
*ZnS*	0.02 (0.01, 0.05)a	0.04 (0.02, 0.07)b	0.04 (0.02, 0.07)b	0.0002
**Population Differentiation**				
AFR - F_st_	-	0.12 (0.07, 0.16)	0.09 (0.06, 0.14)	0.03
AFR - D_xy_	-	0.001 (0.0008, 0.002)	0.001 (0.0008, 0.002)	0.90
AFR - H_st_	-	0.009 (0.0003, 0.03)	0.008 (0.0004, 0.02)	0.31
**Effect of Divergence on Nucleotide Diversity**				
*π*/F_st_		0.006 (0.004, 0.01)	0.007 (0.005, 0.02)	0.07
*π*/D_xy_		0.76 (0.64, 0.88)	0.82 (0.71, 0.90)	0.02

Medians and distributions of 114 BD, associated genes are compared by non-parametric Kruskal–Wallis one-way ANOVA, followed by non-parametric Wilcoxon pairwise tests. Small letters ‘a’ and ‘b’ represent significantly different pairwise comparisons between the three populations. S: segregating sites, Eta: total number of mutations, Hap: total number of haplotypes, Hd: Haplotype diversity, π (pi): nucleotide diversity, θ_K_ (ThetaK): average number of nucleotide differences, θ_W_ (ThetaW): watterson theta.

Following comparison of three populations based on pooling of population genetics parameter estimates of all BD associated genes, parameter estimates of all genes were ranked from smallest to largest for each population (East Asian, European, and African). Each parameter (such as nucleotide diversity, haplotype diversity, Tajima’s *D*, etc.) was ranked separately within each population. Genes falling within the lowest (values less than 25th percentile) and highest (values greater than 75th percentile) quartiles for each parameter estimate were identified. This ranking and identifying genes with the lowest and highest quartile values was performed separately for East Asian, African, and European populations. Then, these lists were merged, and genes with the highest and lowest quartile population genetic parameter values in the three populations were compared. Comparison of genes with the highest and lowest quartile population genetic parameter values in the three populations showed overlapping rank orders for most genes but also identified genes with values that are unique to East Asians (Supplementary Tables S7, S8, and S17). For example, HLA genes were among the genes that showed the highest nucleotide diversity, and overabundance of intermediate frequency alleles (high positive Tajima’s, and Fu-Li test results) suggesting balancing selection in all three meta-populations. But some genes reported to be among the most significantly associated with BD showed extreme reduction in nucleotide diversity, and excess of rare variants (such as *DTL, NOD2, FUT2, IL23R*, *SMARCA2*, *STX8*) only in East Asians, suggesting a unique evolutionary history of these genes in East Asians, where highest BD incidence and prevalence is reported.

#### 3.3.2 Population differentiation and divergence

Following intra-population analyses, inter-population statistics is conducted to compare population differentiation between East Asian, African, and European populations (Supplementary Tables S9 and S10). Population differentiation between Africans and East Asians was higher compared to population differentiation between Africans and Europeans based on the F_st_ statistic of all 114 genes (Table 2). Although average net nucleotide divergence (D_xy_) and haplotype divergence (H_st_) between Africans and East Asians, and Africans and Europeans was similar, the effect of divergence on nucleotide diversity was larger leading to more reduction in nucleotide diversity in East Asians (Table 2). Genes with high nucleotide diversity, such as HLA genes, and genes with big differences in rare allele profiles between populations showed the lowest F_st_ values. Comparison of highest and lowest quartile population differentiation and divergence parameters for each gene among the three populations again showed overlaps but also identified genes with divergence patterns unique to East Asians, where *FUT2*, *IL6*, *OSR1*, *IL1A*, *HNF4G*, *HMP19* showed the highest F_st_ between East Asians and Africans (Supplementary Tables S11, S12, and S17).

Next, we compared BD associated SNP F_st_ estimates (detailed above in Section 3.2) with these variants’ respective genes. Because, we are also interested in testing whether BD associated SNPs show higher population differentiation than other SNPs in the same gene between high BD prevalence (East Asian), and very low/no BD prevalence (African) populations. We expect SNPs reported to be significantly associated with BD to show high population differentiation between high BD prevalent and very low/no BD prevalent populations, at least because the BD risk allele frequency should be higher in the high BD prevalent population. We observed that only 39% (85/216) of BD associated SNPs had higher population differentiation (F_st_) estimate than their respective genes’ population differentiation estimate between East Asians and Africans (Supplementary Table S13). The remaining 61% (131/216) of BD SNPs had lower F_st_ values compared to their genes’ F_st_ estimates (Supplementary Table S13). As expected, some of the SNPs reported to show the most significant association with BD, such as the ones in *PSORS1C1*, *POU5F1*, *MUC21*, *HLA-B*, *IL23R,* and *HLA-G*, had F_st_ estimates greater than their respective genes, supporting higher BD risk allele frequency difference and differentiation in these SNPs compared to other variants in their respective genes. However, contrary to expectations, other SNPs again reported to show some of the most significant association with BD, such as the ones in *CCHCR1*, *IL-10*, *ERAP1*, *TLR4*, and *CCR1* had F_st_ estimates less than their respective genes (Supplementary Table S13).

#### 3.3.3 Recent selective sweeps

We conducted cross population extended haplotype homozygosity (XP-EHH), and integrated haplotype score (iHS) tests to detect possible recent selection on BD associated genes. Absolute iHS values greater than 2 are regarded as indicating recent selection as iHS values greater than 2 constitute the top 1% of the empirical distribution of genome-wide iHS values. iHS values between 1.64 ─ 2.0 are regarded as indicating moderate recent selection as values above 1.635 constitute the top 90th percentile among HapMap Phase-2 SNPs (Voight et al., 2006; Hindorff et al., 2009). Unique to EAS populations, we observed recent selection for around twenty genes involved in pattern recognition (i.e., *NOD2*), intracellular processing of peptides (i.e., *DTL*, *UBASH3B*, *GALNT10*, *LYST*), adaptive immune regulation (HLA genes), differentiation of immune cells (i.e., *EBF2*), and other molecular processes (Supplementary Table S14).

#### 3.3.4 Adaptive protein evolution

We investigated possible adaptive protein evolution in BD associated genes in East Asians by utilizing variations of the McDonald and Kreitman (MK) test, comparing proportion of adaptive substitutions in a MK test (alpha value), ratio of ratios in a MK 2x2 table (Neutrality index: NI), and difference between proportion of nonsynonymous divergence and nonsynonymous polymorphism (DoS: Direction of selection). Negative DoS results, and NI values greater than one suggested negative selection for nearly all genes, however, the results were only statistically significant for *ABCB5, ATP8A1, CPVL, HIVEP3, PSORS1C1, SLC22A23* (Supplementary Table S15) indicating abundance of polymorphic but lack of divergent nonsynonymous (replacement) changes. No adaptive protein evolution or positive selection is inferred for any of the BD associated genes in East Asians.

#### 3.3.5 Gene expression profiles

We downloaded expression profiles of BD associated genes for 27 human tissues from ‘NCBI normal tissue RNA-seq’ database (accessed in April 2022) (Suntsova et al., 2019) for gene expression analyses. Highest gene expression was observed in gastrointestinal tissues either considering all BD associated genes (Supplementary Figure S6A) or considering only the genes show recent selection in East Asians (Supplementary Table S16; Supplementary Figure S6B). Interestingly, BD mostly affect gastrointestinal system in East Asians. Expression in immune function related tissues such as bone marrow, spleen, thyroid, and lymph node was lower than gastrointestinal tissues with a wider expression range (Supplementary Figure S6B).

#### 3.3.6 Genes with population genetic parameter and selection estimates unique to east asians

**FIGURE 4:**
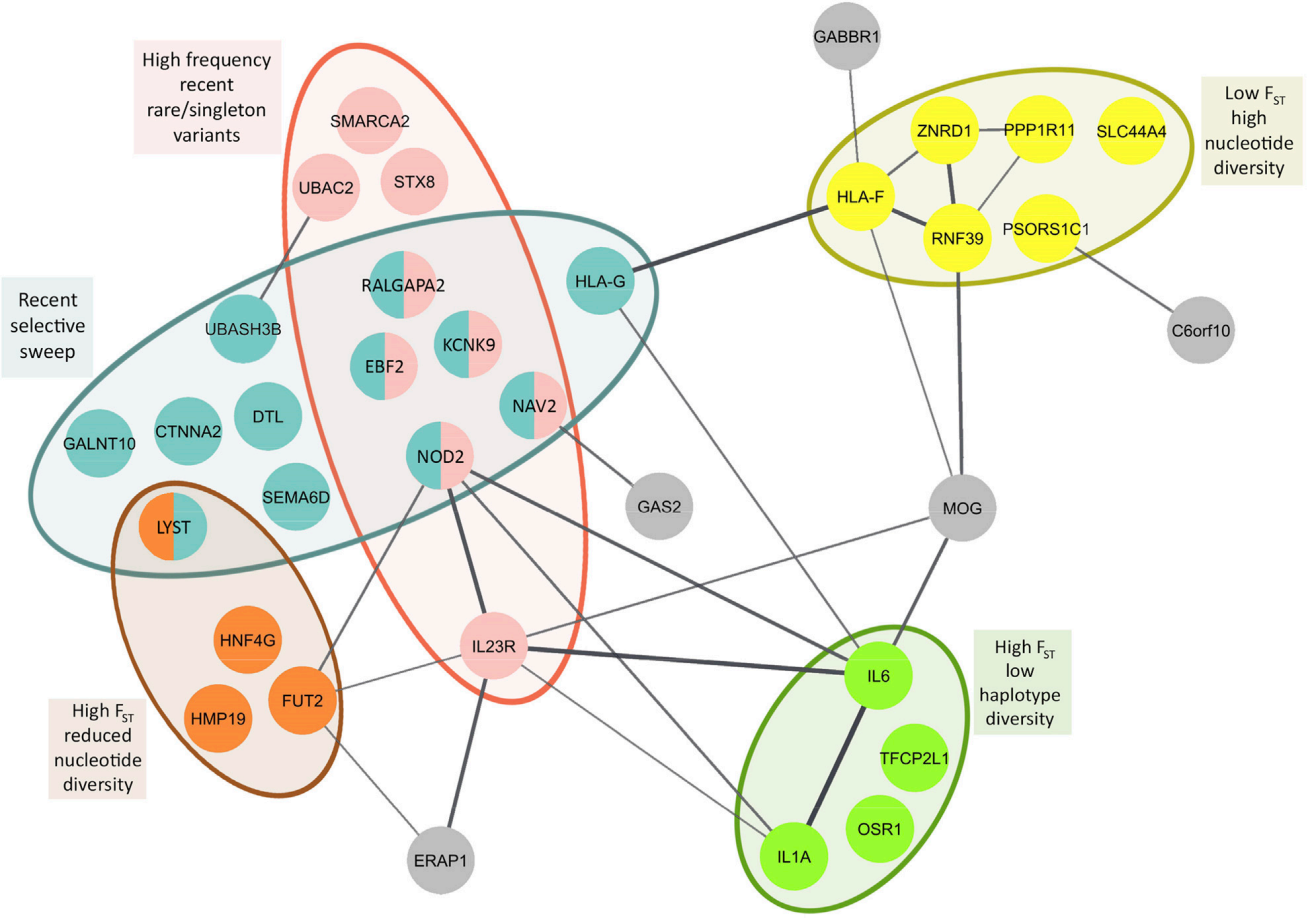
Population genetic parameter and selection estimates superposed on to protein-protein interaction network of BD associated genes with population genetic parameter and selection estimates unique to East Asians. Primary interactions based on functional and physical protein associations only from curated databases and experimentally determined sources are presented. Line thickness of the edges indicates the strength of data support. High and low F_st_ represent population differentiation of East Asians with respect to Africans.

We identified 36 genes with diverse functions and different evolutionary histories unique to East Asians based on differentiation with respect to African populations (F*st*), detection of selective sweep, and abundance of rare/singleton variants. Focusing on iHS values greater than 2, recent soft selective sweep was inferred for *KCNK9*, *NOD2*, *RALGAPA2*, *CTNNA2*, *HLA-G*, *EBF2*, *DTL*, *NAV2*, *GALNT10*, *SEMA6D*, *LYST*, and *UBASH3B*. Among these genes, *KCNK9*, *NOD2*, *RALGAPA2*, *EBF2*, and *NAV2* also showed high frequency of recent rare/singleton variants (Supplementary Table S17, Figure 4). High frequency recent rare/singleton variants were observed for *UBAC2*, *STX8*, *SMARCA2*, and *IL23R* but without a recent selective sweep signal. High F_st_ with respect to African populations and reduced nucleotide diversity was observed in *FUT2*, *HMP19*, *HNF4G*, and *LYST*. On the other hand, high F_st_ and low haplotype diversity was observed in *IL6*, *IL1A*, and *OSR1*. Different from the genes with reduced nucleotide and haplotype diversity, *HLA-F*, *RNF39*, *PSORS1C1*, *PPP1R11*, *ZNRD1*, and *SLC44A4* showed low F_st_ and high nucleotide diversity (Supplementary Table S17, Figure 4).

We performed protein-protein interaction network analysis with these 36 genes and identified two major networks interconnected with each other. HLA-G, HLA-F, RNF39, PPP1R11, ZNRD1, GABBR1, and MOG constituted one of the networks, and interestingly nearly all genes in this network showed the highest nucleotide diversity (Figure 4). All of these genes are found on Chromosome 6 in the extended HLA region with highest expression usually in immune related tissues (Supplementary Tables S16 and S17). The second network includes NOD2, FUT2, ERAP1, IL1A, IL6, and IL23R, the center node of this network. Unlike the first network, the genes in this network show diverse aforementioned population genetic parameter estimates and selection signatures (Figure 4). Highest expression of the genes in this network is observed in the gastrointestinal tissues (Supplementary Tables S16 and S17).

## 4 Discussion

We aimed to understand whether increased BD risk in certain human populations is due to past selection on BD associated genes and their variants in geographic regions where BD prevalence is high, and conducted population genetic and genomic analyses with BD associated genes and their variants in populations with high and low/very low BD prevalence.

In SNP based analyses we observed that both derived and ancestral alleles were associated with increased (or decreased) risk of BD. Moreover, large allele frequency differences and high population differentiation (F_st_) between East Asian and other world populations were observed for both derived and ancestral BD alleles, where population differentiation was even higher for the ancestral BD associated alleles. Comparison of BD associated variants’ allele frequency distribution among East Asians, Europeans, and Africans showed large overlaps and similar allele frequencies. No enrichment or systematic skew with respect to rare or more frequent alleles was observed in any population. These observations indicate BD associated alleles are not unique to East Asians but are also found in other world populations at appreciable frequencies, and argue against selection favoring these variants only in populations with high BD prevalence.

One may expect variants with larger allele frequency difference and high population differentiation between populations with high BD prevalence and populations with none/very low BD cases to confer higher risk, and be more significantly associated with BD. Contrary to expectations, overall, variants showing higher risk to and more significant association with BD had smaller allele frequency differences and showed less population differentiation compared to variants that showed smaller odds ratios (risk) and less significant association with BD. Again, indicating high BD risk variants and genotypes are not only found in populations with high BD occurrence but are also present in populations with none to very low BD cases.

Autoimmune diseases, driven by more active immune system response (Costenbader et al., 2012), can be hypothesized as derived traits in part resulting from selection against pathogens (Fumagalli et al., 2009; Costenbader et al., 2012; Kirino and Remmers, 2015; Takeuchi et al., 2015; Rhodri et al., 2021). BD is considered to have an autoimmune component (Dalvi et al., 2012; Hatemi et al., 2013). Based on this hypothesis, derived alleles with high population differentiation are expected to be the major genetic factors underlying BD susceptibility. However, our results do not support a simple hypothesis that increased BD risk is due to recent positive selection on derived alleles, and that most ancestral alleles are protective against BD. Both derived and ancestral variants with high allele frequency differences and population differentiation between East Asians and other populations that increase BD risk were observed. Therefore, our observations necessitate other alternative selection (or neutral) explanations on BD variants. For example, there can be relaxation of negative selection on BD associated derived alleles due to changes in environmental conditions. Finally, BD associated allele dynamics can be the result of neutral demographic processes independent of any selection. Differentiating these alternative selection processes is not possible by just focusing on allele type and frequency based analyses. Consequently, we conducted population genetic analyses with the genes of the BD associated variants.

Molecular population genetic analyses with BD associated genes in East Asian, European, and African populations showed overlapping parameter estimates suggesting similar evolutionary histories driven by neutral processes for many genes or selection for high nucleotide diversity (possible balancing selection) for HLA genes in all three populations. However, nucleotide diversity in several HLA region genes (such as *HLA-F*, *RNF39*, *ZNRD1*, *PPP1R11*, *PSORS1C1*) was much higher in East Asians compared to other populations suggesting selection for high nucleotide and haplotype diversity in East Asians.

Recognition of infectious organisms’ proteins or human proteins due to molecular mimicry (similarity between pathogen antigens and human peptides) by the innate immune system is suggested to be a trigger in the pathogenesis of BD (Mattioli et al., 2021; Nguyen et al., 2021). Only in East Asian populations, we found signals of recent selective sweep in genes involved in intracellular pattern recognition (*NOD2*), intracellular processing of (foreign) peptides (*DTL*, *UBASH3B*, *GALNT10, LYST*), immune regulation (*HLA-G*), regulation of cellular growth and migration (*CTNNA2*, *NAV2*, *SEMA6D*, *RALGAPA2*), and differentiation of B cells (*EBF2*). Both derived and ancestral alleles in these genes affect BD risk. So, most probably, recent selection is acting on both derived and ancestral alleles. We note that the selective sweep conclusions are based on the strongest selective sweep signal observed on a gene, and selective sweep windows are not necessarily centered on BD associated variants. Therefore the selection can be on other functional variants with unexplored effect on BD.

Other genes involved in microbial recognition (*FUT2*), intracellular processing of peptides (*UBAC2*, *STX8*), triggering of inflammation (*IL6*, *IL1A*), stimulation of Th17 and neutrophils (*IL23R*), cell maturation and differentiation (*SMARCA2*), and transcriptional regulation (*HNF4G*, *TCFP2L1*, *OSR1*) showed the highest population differentiation (Fst between East Asians and Africans), reduced nucleotide and haplotype diversity, and high frequency of recent rare/singleton variants unique to East Asians. These genetic patterns are usually seen in selective sweeps, however no selective sweep is observed for these genes. Even for the genes with a recent selective sweep signal, given their low iHS estimates, the sweep should be considered a soft sweep. Most probably that is why these BD risk genes have not been detected before by genome-wide selection scans. According to soft sweep model, multiple beneficial mutations occur at a locus on different genomic backgrounds, these variants can rise in frequency concurrently, and none of them can reach fixation (Hermisson and Pennings, 2017). BD associated genes have diverse functions involving pathogen detection, self vs non-self recognition, immune response modulation, cellular differentiation, and other unknown functions representing diverse biological pathways. Moreover, these genes influence other inflammatory diseases (Takeuchi et al., 2015). For example, different variants in *IL23R*, that interfere with IL23 signaling, have protective effects not only in BD but also for ankylosing spondylitis (AS), psoriasis, and inflammatory bowel disease (IBD) (Rueda et al., 2008; Momozawa et al., 2011; Kirino et al., 2013b; Tang et al., 2014). IL23R modulates Th17 T-Cell and neutrophil driven inflammation, key factors in immune reaction against pathogens. The ancestral and derived alleles of different variants may confer survival advantage against different pathogens. Therefore, no single variant might have been the main target of selection leading to a selective sweep. Similarly, ancestral and derived genetic variants and haplotypes in *ERAP1* influence several inflammatory diseases in different proportions in European and East Asian populations (Ombrello et al., 2015). In BD, the *ERAP1* effect is limited to individuals with *HLA-B51* ^*^ type, where epistasis/interaction between *ERAP1* and *HLA-B51* ^*^ is suggested (Kirino et al., 2013a). In this study soft selective sweep in *HLA-B* is observed for both East Asians and Europeans. These observations highlight the possible importance of epistasis/interactions shaping the selection acting on BD genes. The soft selective sweep signals observed in this study were population specific. Most signals were only observed in Chinese samples (for example CHB, CHS, KHV populations) but not so much in Japanese samples. This emphasizes the importance of sampling and analyzing local populations rather than analyzing meta-populations to detect soft selective sweep signals. Finally, BD can effect reproductive success, however, disease onset is usually after sexual maturation, and its effect on reproduction is limited (Tiseo et al., 2016; Chan et al., 2021). So, a strong sexual selection against risk alleles driving a hard selective sweep is unlikely. Detecting and understanding the underlying mechanisms of soft sweeps is much harder than hard sweeps, and usually require much larger sample sizes. Future studies generating high density SNP or sequence data from diverse East Asians populations can identify novel sweeps and possible underlying mechanisms.

Gene based analyses also showed genes with population (between high and low/none BD prevalent populations) differentiation estimates higher than their BD associated variants, such as *CCHCR1*, *IL-10*, *ERAP1*, *MEFV*, and *TLR4*, suggesting presence of other variants with higher population differentiation than the reported variants. These unevaluated variants can have possible effect on BD, and may be responsible for the missing heritability in BD. Indeed, targeted sequencing of *ERAP1*, *MEFV*, *TLR4*, and *IL10* in BD patients identified novel population specific functional rare variants strongly associated with BD (Kirino et al., 2013b; Matos et al., 2017). We propose that sequencing of genes with population differentiation estimates higher than their BD associated variants can identify novel BD risk variants, and can contribute to understanding of molecular pathogenesis of BD.

There are several limitations of this study. First, we did not use DNA sequence data from BD patients. Either whole genome or candidate gene resequencing studies with BD patients should test our conclusions. We hypothesize that future analyses with patient sequence data will support our conclusions and find even stronger selection results. For example, the frequency of a recently selected single extended haplotype in HLA-B was three times higher in BD patients compared to the controls (Remmers et al., 2010). Second, we only analyzed East Asian populations. Future studies with high quality genome sequence data from Turkey, Middle East, and Central Asian populations with high BD prevalence should be conducted. Analyses with these populations can not only test our conclusions, but also identify population specific interesting evolutionary histories in BD genes. Third, we only analyzed the coding regions (bounded by well-defined start to end nucleotide positions) but excluded non-coding regions such as extended promoter and down-stream regions of BD genes. As some reported BD associated SNPs are in non-coding regions, future population genetic studies should focus on these non-coding regions.

In conclusion, we presented a comprehensive molecular evolutionary genetic analysis of BD pathogenesis. The evolutionary processes shaping the genetic diversity in BD risk genes are diverse, and certainly elucidating the underlying specific selection mechanisms is complex. We identified a small number of BD risk genes with unique evolutionary histories in East Asians. Future studies with larger sequence data from BD patients and healthy controls sampled from local East Asian populations, and also from other populations with high BD prevalence can uncover the selection mechanisms and their historic reasons in these genes. Several of the genes examined in this study are risk factors for other inflammatory diseases. Thus, our conclusions and propositions are not only limited to BD but may have broader implications for other inflammatory diseases such as rheumatoid arthritis, ankylosing spondylitis, psoriasis, and inflammatory bowel diseases. Similar studies with these diseases may contribute to our understanding of evolution of inflammatory disease susceptibility in humans.

## Data Availability

The original contributions presented in the study are included in the article/Supplementary Material, further inquiries can be directed to the corresponding author.
