# Supplementary material for: Diverse selection pressures shaping the genetic architecture of behçet disease susceptibility
Source: Front Genet. 2022 Sep 30;13:983646. doi: 10.3389/fgene.2022.983646 (PMC9561091; doi:10.3389/fgene.2022.983646)
Supplement: Supplementary file 1 [file DataSheet1.zip › SupplementalTables.docx]

**Supplemental Table 1.** Genes and their variants reported to be associated with Behcet disease.

| **Gene** | **Variant/SNP** | **Type** | **Behcet Allele** | **Effect on Behcet Disease** | **OR** | **P value** | **Study Population** | **Reference** |
| --- | --- | --- | --- | --- | --- | --- | --- | --- |
| *PSORS1C1* | rs4959053 | Intron | A | Susceptible | 3.18 | 1.8x10^-26^ | Japanese | (Mizuki et al., 2010) |
| *POU5F1* | rs9501063 | Exon | G | Susceptible | 2.54 | 1.2x10^-23^ | Japanese | (Mizuki et al., 2010) |
| *CCHCR1* | rs2073716 | Intron | C | Susceptible | 2.57 | 1.7x10^-23^ | Japanese | (Mizuki et al., 2010) |
| *IL-10* | rs1518111 | Intron | A | Susceptible | 1.45 | 3.54x10^-18^ | Turkish | (Zhou et al., 2012; Remmers et al., 2010; Takeuchi et al., 2015) |
| *MUC21* | rs2517446 | Upstream | C | Susceptible | 2.28 | 1.5x10^-17^ | Japanese | (Mizuki et al., 2010) |
| *LOC285830*  *( HLA-F antisense RNA1)* | rs1610637 | Upstream | C | Susceptible | 2.17 | 8.3x10^-17^ | Japanese | (Mizuki et al., 2010) |
| *LOC285830*  *( HLA-F antisense RNA1)* | rs885940 | Upstream | A | Susceptible | 2.13 | 2.4x10^-16^ | Japanese | (Mizuki et al., 2010) |
| *HLA-B* | rs9266409 | Upstream | C | Susceptible | 1.92 | 2.4x10^-16^ | Japanese | (Mizuki et al., 2010) |
| *POU5F1* | rs3130501 | Intron | A | Protective | 0.50 | 2.5x10^-16^ | Japanese | (Mizuki et al., 2010) |
| *HLA-G* | rs2523408 | Upstream | G | Susceptible | 2.10 | 2.7x10^-16^ | Japanese | (Mizuki et al., 2010) |
| *LOC285830*  *( HLA-F antisense RNA1)* | rs1633041 | Upstream | T | Susceptible | 2.15 | 6.2x10^-16^ | Japanese | (Mizuki et al., 2010) |
| *POU5F1* | rs9263804 | Intron | C | Protective | 0.52 | 1.2x10^-15^ | Japanese | (Mizuki et al., 2010) |
| *HLA-G* | rs9258466 | Upstream | G | Susceptible | 2.08 | 1.4x10^-15^ | Japanese | (Mizuki et al., 2010) |
| *HLA-G* | rs1736963 | Upstream | T | Susceptible | 2.08 | 1.5x10^-15^ | Japanese | (Mizuki et al., 2010) |
| *POU5F1* | rs3132524 | Intron | A | Protective | 0.52 | 2x10^-15^ | Japanese | (Mizuki et al., 2010) |
| *HLA-B* | rs9266406 | Upstream | A | Susceptible | 1.86 | 2.1x10^-15^ | Japanese | (Mizuki et al., 2010) |
| *HLA-G* | rs753544 | Upstream | T | Susceptible | 2.08 | 3.3x10^-15^ | Japanese | (Mizuki et al., 2010) |
| *HLA-B* | rs6910516 | Upstream | C | Susceptible | 1.86 | 6x10^-15^ | Japanese | (Mizuki et al., 2010) |
| *IL-10* | rs1800871 | Upstream | T | Susceptible | 1.45 | 1x10^-14^ | Japanese | (Zhou et al., 2012; Mizuki et al., 2010; Takeuchi et al., 2015) |
| *HLA-G* | rs407238 | Downstream | C | Susceptible | 2.01 | 1.6x10^-14^ | Japanese | (Mizuki et al., 2010) |
| *IL-10* | rs1800872 | Upstream | A | Susceptible | 1.45 | 2.1x10^−14^ | Japese, Turkish, Korean | (Zhou et al., 2012; Mizuki et al., 2010) |
| *HLA-G* | rs1633002 | Upstream | A | Susceptible | 2.01 | 1.1x10^-13^ | Japanese | (Mizuki et al., 2010) |
| *HLA-G* | rs1632973 | Upstream | A | Susceptible | 2.00 | 1.6x10^-13^ | Japanese | (Mizuki et al., 2010) |
| *MUC21* | rs2844673 | Downstream | A | Susceptible | 1.96 | 1.7x10^-13^ | Japanese | (Mizuki et al., 2010) |
| *MUC21* | rs2252926 | Downstream | G | Susceptible | 1.97 | 1.8x10^-13^ | Japanese | (Mizuki et al., 2010) |
| *CCR1* | rs7616215 | Downstream | T | Protective | 0.72 | 4.3x10^-13^ | Turkish-Japanese-Han Chinese | (Kirino et al., 2013a) |
| *MUC21* | rs2517411 | Downstream | G | Susceptible | 1.95 | 4.9x10^-13^ | Japanese | (Mizuki et al., 2010) |
| *MEFV* | rs61752717 | Exon | G | Susceptible | 2.65 | 1.79x10^-12^ | Turkish | (Deng et al., 2018; Kirino et al., 2013b) |
| *CCHCR1* | rs2240063 | Intron | A | Protective | 0.55 | 2.1x10^-12^ | Japanese | (Mizuki et al., 2010) |
| *RNF39* | rs9261317 | Exon | A | Susceptible | 2.25 | 3.1x10^-12^ | Japanese | (Mizuki et al., 2010) |
| *MUC21* | rs1632854 | Downstream | T | Susceptible | 1.82 | 3.2x10^-12^ | Japanese | (Mizuki et al., 2010) |
| *MUC21* | rs2523915 | Downstream | T | Susceptible | 1.90 | 7.7x10^-12^ | Japanese | (Mizuki et al., 2010) |
| *TNFAIP3* | rs9494885 | Upstream | T | Protective | 0.50 | 8.26x10^-12^ | Han Chinese | (Li et al., 2013) |
| *HLA-F* | rs3116788 | Upstream | G | Protective | 0.48 | 8.4x10^-12^ | Japanese | (Mizuki et al., 2010) |
| *IFNγ* | UTR-5644 | Downstream | A | Susceptible | 3.53 | 1x10^-11^ | Turkish | (Zhou et al., 2012) |
| *MUC21* | rs1634717 | Downstream | T | Susceptible | 1.80 | 1.6x10^-11^ | Japanese | (Mizuki et al., 2010) |
| *TCF19* | rs2073723 | Intron | T | Protective | 0.57 | 1.9x10^-11^ | Japanese | (Mizuki et al., 2010) |
| *MUC21* | rs2252925 | Downstream | G | Susceptible | 1.87 | 2.1x10^-11^ | Japanese | (Mizuki et al., 2010) |
| *C6orf15 (STG)* | rs1265048 | Upstream | A | Protective | 0.59 | 2.6x10^-11^ | Japanese | (Mizuki et al., 2010) |
| *HLA-C* | rs3905495 | Upstream | C | Protective | 0.59 | 4.7x10^-11^ | Japanese | (Mizuki et al., 2010) |
| *BTNL2* | rs2076530 | Exon | G | Protective | 0.59 | 4.7x10^-11^ | Japanese | (Mizuki et al., 2010) |
| *ERAP1* | rs17482078 | Exon | T | Susceptible | 4.56 | 4.73x10^-11^ | Turkish | (Kirino et al., 2013a; Takeuchi et al., 2015) |
| *HLA-F* | rs1610584 | Upstream | T | Protective | 0.51 | 6.5x10^-11^ | Japanese | (Mizuki et al., 2010) |
| *ZNRD1* | rs9261265 | Upstream | C | Susceptible | 2.04 | 7.3x10^-11^ | Japanese | (Mizuki et al., 2010) |
| *HLA-F* | rs1611388 | Upstream | C | Protective | 0.52 | 7.8x10^-11^ | Japanese | (Mizuki et al., 2010) |
| *HLA-DQA1* | rs9272346 | Upstream | G | Protective | 0.60 | 8.7x10^-11^ | Japanese | (Mizuki et al., 2010) |
| *LOC285830*  *( HLA-F antisense RNA1)* | rs2844845 | Intron | A | Susceptible | 2.07 | 9.5x10^-11^ | Japanese | (Mizuki et al., 2010) |
| *HCG9* | rs9260954 | Downstream | G | Susceptible | 2.06 | 1.2x10^-10^ | Japanese | (Mizuki et al., 2010) |
| *GABBR1* | rs29273 | Upstream | G | Susceptible | 2.03 | 1.4x10^-10^ | Japanese | (Mizuki et al., 2010) |
| *MUC21* | rs2530710 | Upstream | A | Protective | 0.53 | 3x10^-10^ | Japanese | (Mizuki et al., 2010) |
| *HLA-F* | rs1610585 | Upstream | C | Protective | 0.52 | 3.3x10^-10^ | Japanese | (Mizuki et al., 2010) |
| *HCG9* | rs6926792 | Downstream | A | Susceptible | 1.75 | 3.3x10^-10^ | Japanese | (Mizuki et al., 2010) |
| *DHFRP2* | rs7761068 | Exon | T | Protective | 0.48 | 3.5x10^-10^ | Japanese | (Mizuki et al., 2010) |
| *UBD* | rs3025657 | Downstream | G | Protective | 0.48 | 3.8x10^-10^ | Japanese | (Mizuki et al., 2010) |
| *HCG27* | rs3130944 | Downstream | C | Susceptible | 1.70 | 4.3x10^-10^ | Japanese | (Mizuki et al., 2010) |
| *HLA-F* | rs1610593 | Upstream | T | Protective | 0.53 | 4.5x10^-10^ | Japanese | (Mizuki et al., 2010) |
| *MICA* | rs3094584 | Downstream | T | Susceptible | 1.69 | 4.7x10^-10^ | Japanese | (Mizuki et al., 2010) |
| *HLA-F* | rs1611381 | Upstream | T | Protective | 0.53 | 5.6x10^-10^ | Japanese | (Mizuki et al., 2010) |
| *ABCB5* | rs2190411 | Intron | C | Susceptible | 2.51 | 8.77x10^-10^ | Han Chinese | (Hou et al., 2012c) |
| *C6orf10 (TSBP1)* | rs574710 | Intron | G | Susceptible | 1.65 | 9.7x10^-10^ | Japanese | (Mizuki et al., 2010) |
| *C6orf47 (G4)* | rs2242655 | Exon | C | Susceptible | 1.70 | 1.1x10^-09^ | Japanese | (Mizuki et al., 2010) |
| *LOC285830*  *( HLA-F antisense RNA1)* | rs2523386 | Intron | A | Susceptible | 2.02 | 1.2x10^-09^ | Japanese | (Mizuki et al., 2010) |
| *KLRC4* | rs2617170 | Exon | C | Protective | 0.78 | 1.34x10^-09^ | Turkish-Japanese | (Kirino et al., 2013a; Takeuchi et al., 2015) |
| *SGPP2* | rs17562982 | Intron | T | Susceptible | 2.79 | 1.91x10^-09^ | Han Chinese | (Hou et al., 2012c) |
| *TRIM31* | rs9261376 | Downstream | G | Susceptible | 1.67 | 2.2x10^-09^ | Japanese | (Mizuki et al., 2010) |
| *MICA* | rs2523467 | Upstream | A | Protective | 0.59 | 2.3x10^-09^ | Japanese | (Mizuki et al., 2010) |
| *SUSD1* | rs2782932 | Intron | T | Susceptible | 2.41 | 2.47x10^-09^ | Han Chinese | (Hou et al., 2012c) |
| *TRIM31* | rs6923832 | Downstream | A | Susceptible | 2.00 | 2.5x10^-09^ | Japanese | (Mizuki et al., 2010) |
| *HLA-F* | rs1627465 | Upstream | C | Protective | 0.54 | 2.6x10^-09^ | Japanese | (Mizuki et al., 2010) |
| *C6orf10 (TSBP1)* | rs544358 | Intron | C | Susceptible | 1.63 | 2.8x10^-09^ | Japanese | (Mizuki et al., 2010) |
| *RIMBP2* | rs2895135 | Intron | A | Susceptible | 2.55 | 3.35x10^-09^ | Han Chinese | (Hou et al., 2012c) |
| *HLA-F* | rs1611356 | Upstream | G | Protective | 0.55 | 3.4x10^-09^ | Japanese | (Mizuki et al., 2010) |
| *HLA-DQA1* | rs9272723 | Intron | T | Protective | 0.61 | 4.8x10^-09^ | Japanese | (Mizuki et al., 2010) |
| *C6orf10 (TSBP1)* | rs926591 | Intron | T | Susceptible | 1.62 | 5.1x10^-09^ | Japanese | (Mizuki et al., 2010) |
| *BAG6(BAT3)* | rs2077102 | Intron | T | Susceptible | 1.64 | 5.2x10^-09^ | Japanese | (Mizuki et al., 2010) |
| *C6orf10 (TSBP1)* | rs539703 | Intron | C | Susceptible | 1.61 | 5.3x10^-09^ | Japanese | (Mizuki et al., 2010) |
| *FUT2* | rs681343 | Exon | T | Susceptible | 1.30 | 5.9x10^-09^ | Iranian-Turkish | (Takeuchi et al., 2015) |
| *API5 (LINC01499)* | rs16937370 | Upstream | G | Susceptible | 2.46 | 6.01x10^-09^ | Han Chinese | (Hou et al., 2012c) |
| *TRIM31* | rs9261389 | Downstream | G | Susceptible | 1.64 | 6.1x10^-09^ | Japanese | (Mizuki et al., 2010) |
| *SMG6* | rs749240 | Exon | T | Susceptible | 2.49 | 6.43x^-09^ | Han Chinese | (Hou et al., 2012c) |
| *IL23R,IL12RB2* | rs924080 | Down/Up-stream | T | Susceptible | 1.28 | 6.69x10^-09^ | Japanese-Turkish | (Zhou et al., 2012; Remmers et al., 2010) |
| *HLA-G* | rs1077433 | Upstream | A | Susceptible | 1.79 | 6.9x10^-09^ | Japanese | (Mizuki et al., 2010) |
| *C6orf10 (TSBP1)* | rs4959093 | Intron | C | Susceptible | 1.62 | 8.6x10^-09^ | Japanese | (Mizuki et al., 2010) |
| *LOC285830*  *( HLA-F antisense RNA1)* | rs9258205 | Intron | C | Protective | 0.56 | 9.5x10^-09^ | Japanese | (Mizuki et al., 2010) |
| *SLC44A4* | rs11965547 | Intron | A | Susceptible | 1.65 | 1x10^-08^ | Japanese | (Mizuki et al., 2010) |
| *MOG* | rs3129045 | Downstream | T | Susceptible | 1.78 | 1.2x10^-08^ | Japanese | (Mizuki et al., 2010) |
| *ZNRD1* | rs9261189 | Upstream | T | Susceptible | 1.64 | 1.4x10^-08^ | Japanese | (Mizuki et al., 2010) |
| *HLA-F* | rs7741807 | Upstream | G | Protective | 0.54 | 1.5x10^-08^ | Japanese | (Mizuki et al., 2010) |
| *HCG9* | rs6931776 | Downstream | G | Susceptible | 1.63 | 1.7x10^-08^ | Japanese | (Mizuki et al., 2010) |
| *SLC43A3* | rs549630 | Downstream | G | Susceptible | 2.27 | 2.04x10^-08^ | Han Chinese | (Hou et al., 2012c) |
| *HLA-DQB1* | rs6457617 | Upstream | C | Protective | 0.63 | 2.1x10^-08^ | Japanese | (Mizuki et al., 2010) |
| *GALNTL1* | rs12589991 | Intron | A | Susceptible | 2.51 | 2.16x10^-08^ | Han Chinese | (Hou et al., 2012c) |
| *PPP1R11* | rs2074482 | Exon | T | Susceptible | 1.60 | 2.2x10^-08^ | Japanese | (Mizuki et al., 2010) |
| *HLA-G* | rs1736951 | Upstream | A | Susceptible | 1.75 | 2.3x10^-08^ | Japanese | (Mizuki et al., 2010) |
| *LOC285830*  *( HLA-F antisense RNA1)* | rs1615251 | Upstream | T | Susceptible | 1.62 | 2.7x10^-08^ | Japanese | (Mizuki et al., 2010) |
| *IL23R,IL12RB2* | rs12119179 | Down/Up-stream | A | Susceptible | 1.55 | 2.7x10^-08^ | Japanese-Turkish | (Mizuki et al., 2010) |
| *UBD* | rs6933331 | Downstream | A | Protective | 0.55 | 2.8x10^-08^ | Japanese | (Mizuki et al., 2010) |
| *ZNRD1* | rs3869068 | Upstream | A | Susceptible | 1.61 | 3.3x10^-08^ | Japanese | (Mizuki et al., 2010) |
| *SLIT2* | rs13435197 | Intron | A | Susceptible | 2.46 | 3.59x10^-08^ | Han Chinese | (Hou et al., 2012c) |
| *HCG9* | rs6911737 | Downstream | A | Susceptible | 1.60 | 4.1x10^-08^ | Japanese | (Mizuki et al., 2010) |
| *LOC285830*  *( HLA-F antisense RNA1)* | rs1737031 | Upstream | A | Susceptible | 1.59 | 4.4x10^-08^ | Japanese | (Mizuki et al., 2010) |
| *ASB18* | rs7561555 | Intron | C | Susceptible | 2.28 | 4.7x10^-08^ | Han Chinese | (Hou et al., 2012c) |
| *IL23R,IL12RB2* | rs11209033 | Down/Up-stream | C | Susceptible | 1.54 | 5.5x10^-08^ | Japanese-Turkish | (Mizuki et al., 2010) |
| *GIMAP4* | rs1608157 | Upstream | C | Susceptible | 2.53 | 6.01x10^-08^ | Korean | (Lee et al., 2013; Deng et al., 2018) |
| *IL-10* | rs1554286 | Intron | C | Protective | 0.62 | 8x10^-08^ | Chinese | (Mizuki et al., 2010) |
| *IL23R,IL12RB2* | rs12141431 | Down/Up-stream | C | Susceptible | 1.52 | 1.1x10^-07^ | Han Chinese | (Mizuki et al., 2010) |
| *CPLX1* | rs11248047 | Upstream | A | Susceptible | 1.36 | 1.26x10^-07^ | Turkish | (Remmers et al., 2010) |
| *COL12A1* | rs4640857 | Downstream | G | Protective | 0.65 | 1.3x10^-07^ | Japanese | (Mizuki et al., 2010) |
| *GIMAP4* | rs1916012 | Upstream | T | Susceptible | 2.38 | 2.62x10^-07^ | Korean | (Lee et al., 2013) |
| *GIMAP4* | rs1522596 | Upstream | T | Susceptible | 2.38 | 3.47x10^-07^ | Korean | (Lee et al., 2013; Deng et al., 2018) |
| *DNMT3A* | rs1465825 | Intron | C | Protective | 0.49 | 3.83x10^-07^ | Han Chinese | (Hou et al., 2012c) |
| *C10orf11 (LRMDA)* | rs1323076 | Intron | G | Protective | 0.61 | 1.2x10^-06^ | Japanese | (Mizuki et al., 2010) |
| *PAX8* | rs11123169 | Downstream | C | Susceptible | 1.53 | 1.3x10^-06^ | Japanese | (Mizuki et al., 2010) |
| *MSX2* | rs10516130 | Downstream | A | Protective | 0.23 | 2.98x10^-06^ | Han Chinese | (Hou et al., 2012c) |
| *SORBS2* | rs4493590 | Intron | G | Susceptible | 1.86 | 4.88x10^-06^ | Han Chinese | (Hou et al., 2012c) |
| *HIVEP3* | rs4660590 | Intron | A | Protective | 0.69 | 5.7x10^-06^ | Japanese | (Mizuki et al., 2010) |
| *CEP135* | rs2593082 | Intron | T | Susceptible | 1.42 | 6.2x10^-06^ | Japanese | (Mizuki et al., 2010) |
| *UBAC2* | rs3825427 | Upstream | T | Susceptible | 1.50 | 6.9x10^-06^ | Chinese | (Hou et al., 2012a; Deng et al., 2018) |
| *RALGAPA2* | rs6082210 | Upstream | A | Protective | 0.17 | 7.01x10^-06^ | Han Chinese | (Hou et al., 2012c) |
| *GIMAP1* | rs2286900 | Exon | T | Susceptible | 1.81 | 9.22x10^-06^ | Korean | (Lee et al., 2013; Deng et al., 2018) |
| *TTLL7* | rs11163772 | Downstream | A | Susceptible | 1.52 | 9.5x10^-06^ | Japanese | (Mizuki et al., 2010) |
| *HMP19* | rs1909704 | Downstream | A | Susceptible | 1.43 | 9.5x10^-06^ | Japanese | (Mizuki et al., 2010) |
| *TFCP2L1* | rs17006292 | Intron | A | Protective | 0.13 | 1.03x10^-05^ | Han Chinese | (Hou et al., 2012c) |
| *TENM4(ODZ4)* | rs2156215 | Intron | T | Protective | 0.69 | 1.1x10^-05^ | Japanese | (Mizuki et al., 2010) |
| *OSR1* | rs4666492 | Upstream | G | Protective | 0.62 | 1.2x10^-05^ | Japanese | (Mizuki et al., 2010) |
| *KLRK1* | rs2617151 | Intron | A | Protective | 0.63 | 1.2x10^-05^ | Japanese | (Mizuki et al., 2010) |
| *CTNNA2* | rs4852547 | Intron | G | Protective | 0.62 | 1.3x10^-05^ | Japanese | (Mizuki et al., 2010) |
| *IL12A* | rs17810546 | Upstream | A | Susceptible | 1.66 | 1.49x10^-05^ | Turkish-mixed | (Remmers et al., 2010; Takeuchi et al., 2015) |
| *MN1* | rs134006 | Downstream | C | Susceptible | 1.46 | 1.6x10^-05^ | Japanese | (Mizuki et al., 2010) |
| *CEP135* | rs2611826 | Intron | G | Susceptible | 1.40 | 1.6x10^-05^ | Japanese | (Mizuki et al., 2010) |
| *PSMD14* | rs6744214 | Intron | T | Susceptible | 1.76 | 1.67x10^-05^ | Han Chinese | (Hou et al., 2012c) |
| *API5 (LINC01499)* | rs420798 | Intron | C | Susceptible | 1.72 | 1.79x10^-05^ | Han Chinese | (Hou et al., 2012c) |
| *C6orf85(SLC22A23)* | rs12194547 | Intron | C | Protective | 0.16 | 1.91x10^-05^ | Han Chinese | (Hou et al., 2012c) |
| *PSMD14* | rs6733456 | Intron | C | Susceptible | 1.75 | 1.98x10^-05^ | Han Chinese | (Hou et al., 2012c) |
| *LTN1(RNF160)* | rs2832137 | Downstream | T | Protective | 0.64 | 2.1x10^-05^ | Japanese | (Mizuki et al., 2010) |
| *HERPUD2* | rs11763983 | Downstream | T | Protective | 0.66 | 2.1x10^-05^ | Japanese | (Mizuki et al., 2010) |
| *LILRA1* | rs103294 | Upstream | C | Susceptible | 1.76 | 2.19x10^-05^ | Han Chinese | (Hou et al., 2012c) |
| *LILRB1* | rs798887 | Upstream | A | Susceptible | 1.83 | 2.23x10^-05^ | Han Chinese | (Hou et al., 2012c) |
| *SAMD3(TMEM200A)* | rs9483115 | Intron | T | Protective | 0.72 | 2.5x10^-05^ | Japanese | (Mizuki et al., 2010) |
| *GALNT10* | rs574750 | Intron | A | Susceptible | 1.66 | 2.5x10^-05^ | Japanese | (Mizuki et al., 2010) |
| *GIMAP2* | rs10266069 | Upstream | A | Susceptible | 1.83 | 2.57x10^-05^ | Korean | (Lee et al., 2013; Deng et al., 2018) |
| *KLRK1* | rs2733852 | Intron | G | Protective | 0.65 | 2.8x10^-05^ | Japanese | (Mizuki et al., 2010) |
| *DEPDC1* | rs6692084 | Upstream | A | Susceptible | 1.89 | 2.81x10^-05^ | Han Chinese | (Hou et al., 2012c) |
| *GIMAP2* | rs10256482 | Upstream | T | Susceptible | 1.83 | 2.82x10^-5^ | Korean | (Lee et al., 2013; Deng et al., 2018) |
| *UBAC2* | rs9517701 | Intron | G | Susceptible | 1.40 | 2.9x10^-05^ | Chinese | (Hou et al., 2012a) |
| *DEPDC1* | rs12134670 | Upstream | C | Susceptible | 2.15 | 3.13x10^-05^ | Han Chinese | (Hou et al., 2012c) |
| *SEMA6D* | rs470151 | Downstream | T | Susceptible | 1.54 | 3.2x10^-05^ | Japanese | (Mizuki et al., 2010) |
| *CDH26* | rs817277 | Downstream | A | Susceptible | 1.78 | 3.24x10^-05^ | Han Chinese | (Hou et al., 2012c) |
| *PMFBP1* | rs11862324 | Upstream | T | Susceptible | 1.39 | 3.3x10^-05^ | Japanese | (Mizuki et al., 2010) |
| *SAMD3* | rs4897380 | Intron | C | Susceptible | 1.38 | 3.4x10^-05^ | Japanese | (Mizuki et al., 2010) |
| *NAV2* | rs2707110 | Intron | C | Susceptible | 1.43 | 3.5x10^-05^ | Japanese | (Mizuki et al., 2010) |
| *UBAC2* | rs9517668 | Intron | T | Susceptible | 2.62 | 3.61x10^-05^ | Turkish-Italy | (Sawalha et al., 2011) |
| *STK39* | rs2390639 | Intron | A | Susceptible | 1.72 | 3.97x10^-05^ | Han Chinese | (Hou et al., 2012c) |
| *TMEM132B* | rs4435061 | Intron | A | Protective | 0.73 | 4x10^-05^ | Japanese | (Mizuki et al., 2010) |
| *STX8* | rs1549332 | Intron | A | Susceptible | 1.59 | 4x10^-05^ | Japanese | (Mizuki et al., 2010) |
| *SAMD3(TMEM200A)* | rs4141940 | Intron | A | Protective | 0.73 | 4x10^-05^ | Japanese | (Mizuki et al., 2010) |
| *CCDC180* | rs2061634 | Exon | G | Susceptible | 2.04 | 4.2x10^-05^ | Turkish | (Fei et al., 2009; Hou et al., 2012a) |
| *OVCH1* | rs1436321 | Downstream | A | Susceptible | 1.39 | 4.4x10^-05^ | Japanese | (Mizuki et al., 2010) |
| *SLC41A2* | rs2731031 | Intron | A | Susceptible | 1.40 | 4.7x10^-05^ | Japanese | (Mizuki et al., 2010) |
| *NAV2* | rs873764 | Intron | G | Susceptible | 1.42 | 4.7x10^-05^ | Japanese | (Mizuki et al., 2010) |
| *HNF4G* | rs2980221 | Upstream | A | Protective | 0.71 | 4.8x10^-05^ | Japanese | (Mizuki et al., 2010) |
| *IL1 (IL1A)* | rs1800587 | Exon | C | Susceptible | 2.90 | 5x10^-05^ | Turkish | (Zhou et al., 2012) |
| *SMARCA2* | rs7033529 | Intron | A | Protective | 0.73 | 5.1x10^-05^ | Japanese | (Mizuki et al., 2010) |
| *EBF2* | rs4570167 | Intron | C | Susceptible | 1.47 | 5.2x10^-05^ | Japanese | (Mizuki et al., 2010) |
| *GAS2* | rs10833804 | Intron | G | Protective | 0.73 | 5.4x10^-05^ | Japanese | (Mizuki et al., 2010) |
| *PAX8* | rs10864912 | Intron | T | Susceptible | 1.47 | 5.7x10^-05^ | Japanese | (Mizuki et al., 2010) |
| *DTL* | rs1472224 | Downstream | G | Protective | 0.16 | 5.73x10^-05^ | Han Chinese | (Hou et al., 2012c) |
| *STAT4* | rs897200 | Intron | A | Susceptible | 1.45 | 5.88x10^-05^ | Han Chinese | (Hou et al., 2012c; Takeuchi et al., 2015) |
| *LYST/NID1* | rs7354999 | Down/Up-stream | G | Susceptible | 1.38 | 6.1x10^-05^ | Japanese | (Mizuki et al., 2010) |
| *STK39* | rs3769393 | Intron | G | Susceptible | 1.70 | 6.17x10^-05^ | Han Chinese | (Hou et al., 2012c) |
| *TMEM132B* | rs10846917 | Intron | T | Susceptible | 1.39 | 6.3x10^-05^ | Japanese | (Mizuki et al., 2010) |
| *LOC100132252* | rs9469615 | Intergenic | C | Protective | 0.63 | 6.3x10^-05^ | Japanese | (Mizuki et al., 2010) |
| *CDH26* | rs817283 | Downstream | A | Susceptible | 1.74 | 6.42x10^-05^ | Han Chinese | (Hou et al., 2012c) |
| *SAMD3(TMEM200A)* | rs899276 | Intron | A | Protective | 0.73 | 6.5x10^-05^ | Japanese | (Mizuki et al., 2010) |
| *LOC107984355* | rs872837 | Intron | A | Susceptible | 1.46 | 6.5x10^-05^ | Japanese | (Mizuki et al., 2010) |
| *SACM1L* | rs1969624 | Intron | C | Protective | 0.67 | 6.8x10^-05^ | Japanese | (Mizuki et al., 2010) |
| *IL23R* | rs11209026 | Intron | A | Protective | 0.68 | 6.9x10^-05^ | Turkish-Japanese | (Deng et al., 2018) |
| *IL23R* | rs76418789 | Intron | A | Protective | 0.54 | 6.9x10^-05^ | Turkish-Japanese | (Deng et al., 2018) |
| *PLEKHB1* | rs591804 | Intron | G | Protective | 0.72 | 7.4x10^-05^ | Japanese | (Mizuki et al., 2010) |
| *SAMD3(TMEM200A)* | rs7758496 | Intron | G | Protective | 0.73 | 7.7x10^-05^ | Japanese | (Mizuki et al., 2010) |
| *TMEM132B* | rs10846924 | Intron | T | Protective | 0.74 | 8x10^-05^ | Japanese | (Mizuki et al., 2010) |
| *ATP8A1* | rs2100766 | Upstream | T | Protective | 0.62 | 8.2x10^-05^ | Japanese | (Mizuki et al., 2010) |
| *IL23R, IL12RB2* | rs1495965 | Down/Up-stream | G | Susceptible | 1.25 | 8.4x10^-05^ | Japanese-Turkish | (Zhou et al., 2012; Mizuki et al., 2010) |
| *EBF2* | rs4242425 | Intron | T | Susceptible | 1.44 | 8.4x10^-05^ | Japanese | (Mizuki et al., 2010) |
| *UBAC2* | rs9554581 | Intron | T | Susceptible | 2.48 | 8.53x10^-05^ | Turkish-Italy | (Sawalha et al., 2011) |
| *STAT4* | rs7574070 | Intron | A | Susceptible | 1.27 | 8.56x10^-05^ | Turkish-Japanese | (Kirino et al., 2013a; Hou et al., 2012c; Takeuchi et al., 2015) |
| *CCR1* | rs17282391 | Downstream | G | Protective | 0.15 | 8.66x10^-05^ | Han Chinese | (Hou et al., 2012b) |
| *CCR1* | rs13084057 | Downstream | G | Protective | 0.15 | 8.66x10^-05^ | Han Chinese | (Hou et al., 2012b) |
| *CCR1* | rs7631551 | Downstream | A | Protective | 0.15 | 8.66x10^-05^ | Han Chinese | (Hou et al., 2012b) |
| *C10orf11(LRMDA)* | rs17434565 | Intron | G | Protective | 0.64 | 9x10^-05^ | Japanese | (Mizuki et al., 2010) |
| *SAMD3(TMEM200A)* | rs724324 | Intron | G | Protective | 0.74 | 9.5x10^-05^ | Japanese | (Mizuki et al., 2010) |
| *KCNK9* | rs1961261 | Downstream | A | Protective | 0.69 | 9.5x10^-05^ | Japanese | (Mizuki et al., 2010) |
| *STAT4* | rs7572482 | Intron | A | Susceptible | 1.68 | 9.77x10^-05^ | Turkish | (Hou et al., 2012c) |
| *CCR3* | rs13075270 | Intron | C | Protective | 0.13 | 9.9x10^-05^ | Han Chinese | (Hou et al., 2012b) |
| *CCR3* | rs13092160 | Intron | C | Protective | 0.13 | 9.9x10^-05^ | Han Chinese | (Hou et al., 2012b) |
| *CCR3* | rs2373156 | Intron | T | Protective | 0.13 | 9.9x10^-05^ | Han Chinese | (Hou et al., 2012b) |
| *CCR3* | rs1542755 | Intron | A | Protective | 0.13 | 9.9x10^-05^ | Han Chinese | (Hou et al., 2012b) |
| *UBAC2* | rs727263 | Intron | A | Susceptible | 2.45 | 1x10^-04^ | Turkish-Italy | (Sawalha et al., 2011) |
| *CPVL* | rs317711 | Intron | C | Susceptible | 2.26 | 1x10^-04^ | Turkish | (Fei et al., 2009; Hou et al., 2012a) |
| *IL17F-A126G, (Glu126Gly)* | rs2397084 | Exon | T | Protective | 0.06 | 1x10^-04^ | Korean | (Jang et al., 2008) |
| *UBAC2* | rs7332161 | Intron | A | Susceptible | 2.43 | 1.1x10^-04^ | Turkish-Italy | (Sawalha et al., 2011) |
| *IL23R* | rs17375018 | Intron | G | Susceptible | 1.57 | 1.11x10^-04^ | Han Chinese | (Zhou et al., 2012; Jiang et al., 2010) |
| *CCR1* | rs10510749 | Downstream | T | Protective | 0.16 | 1.22x10^-04^ | Han Chinese | (Hou et al., 2012b) |
| *IL23R* | rs11209032 | Downstream | A | Susceptible | 1.48 | 1.58x10^-04^ | Han Chinese | (Zhou et al., 2012; Jiang et al., 2010) |
| *UBAC2* | rs17575643 | Intron | T | Susceptible | 2.91 | 1.8x10^-04^ | Turkish-Italy | (Sawalha et al., 2011) |
| *SUMO4* | rs237024 | Downstream | C | Susceptible | 1.70 | 2x10^-04^ | Han Chinese | (Deng et al., 2018) |
| *IL1* | rs16944 | Upstream | G | Susceptible | 2.19 | 2x10^-04^ | Turkish | (Zhou et al., 2012) |
| *CCR3* | rs13067058 | Intron | A | Protective | 0.14 | 2x10^-04^ | Han Chinese | (Hou et al., 2012b) |
| *UBAC2* | rs2892976 | Downstream | G | Susceptible | 1.96 | 2.3x10^-04^ | Turkish-Italy | (Sawalha et al., 2011) |
| *LOC100129342* | rs11206377 | Intergenic | G | Susceptible | 1.84 | 3x10^-04^ | Turkish | (Fei et al., 2009; Hou et al., 2012a) |
| *UBAC2* | rs7999348 | Intron | G | Susceptible | 1.78 | 5.8x10^-04^ | Turkish-Italy | (Sawalha et al., 2011) |
| *TNFAIP3* | rs10499194 | Upstream | C | Susceptible | 1.92 | 1x10^-03^ | Han Chinese | (Li et al., 2013) |
| *TNFAIP3* | rs7753873 | Upstream | C | Susceptible | 1.49 | 1x10^-03^ | Han Chinese | (Li et al., 2013; Deng et al., 2018) |
| *UBAC2* | rs6491493 | Intron | G | Susceptible | 1.74 | 1.1x10^-03^ | Turkish-Italy | (Sawalha et al., 2011) |
| *UBAC2* | rs9554573 | Intron | A | Susceptible | 1.73 | 1.2x10^-03^ | Turkish-Italy | (Sawalha et al., 2011) |
| *UBAC2* | rs9517644 | Upstream | T | Susceptible | 1.72 | 1.3x10^-03^ | Turkish-Italy | (Sawalha et al., 2011) |
| *CCR3* | rs13092160 | Intron | C | Protective | 0.13 | 1.48x10^-03^ | Han Chinese | (Hou et al., 2012b) |
| *UBASH3B* | rs4936742 | Intron | T | Susceptible | 1.71 | 1.5x10^-03^ | Turkish | (Fei et al., 2009; Hou et al., 2012a) |
| *UBAC2* | rs11069357 | Upstream | A | Susceptible | 1.68 | 2x10^-03^ | Turkish-Italy | (Sawalha et al., 2011) |
| *TLR4* | rs4986790 | Exon | G | Protective | 0.64 | 3x10^-03^ | Turkish-Japanese | (Deng et al., 2018) |
| *TLR4* | rs4986791 | Exon | T | Protective | 0.82 | 3x10^-03^ | Turkish-Japanese | (Deng et al., 2018) |
| *CCR3* | rs9990343 | Downstream | G | Protective | 0.48 | 3.4x10^-03^ | Han Chinese | (Hou et al., 2012b) |
| *CCR3* | rs6803980 | Downstream | A | Protective | 0.48 | 3.4x10^-03^ | Han Chinese | (Hou et al., 2012b) |
| *TNFα* | rs1799724 | Upstream | T | Protective | 0.76 | 4x10^-03^ | Turkish | (Zhou et al., 2012) |
| *IL12* | rs3212227 | Downstream A | | Susceptible | 1.84 | 4x10^-03^ | Turkish | (Zhou et al., 2012) |
| *UBAC2* | rs984477 | Intron | G | Susceptible | 1.65 | 4.3x10^-03^ | Turkish-Italy | (Sawalha et al., 2011) |
| *UBAC2* | rs9513584 | Intron | G | Susceptible | 1.61 | 5.8x10^-03^ | Turkish-China | (Fei et al., 2009; Hou et al., 2012a) |
| *TNFα* | rs361525 | Upstream | A | Susceptible | 1.51 | 6x10^-03^ | Turkish | (Zhou et al., 2012) |
| *TNFα* | rs1799964 | Upstream | C | Susceptible | 1.35 | 7x10^-03^ | Turkish | (Zhou et al., 2012) |
| *UBAC2* | rs912130 | Intron | C | Susceptible | 1.58 | 7.1x10^-03^ | Turkish-Italy | (Sawalha et al., 2011) |
| *CCR3* | rs7651539 | Intron | T | Protective | 0.35 | 8x10^-03^ | Han Chinese | (Hou et al., 2012b) |
| *IL18* | rs1946518 | promoter | C | Susceptible | 1.67 | 1.01x10^-02^ | Korean | (Zhou et al., 2012) |
| *IL1beta* | +3962T/C | Exon | C | Susceptible | 2.60 | 1.5x10^-02^ | Turkish | (Akman et al., 2008) |
| *NOD2* | rs2066844 | Exon | T | Protective | 0.40 | 2x10^-02^ | Turkish-Japanese | (Deng et al., 2018) |
| *NOD2* | rs2066845 | Exon | C | Protective | 0.66 | 2x10^-02^ | Turkish-Japanese | (Deng et al., 2018) |
| *NOD2* | rs2066847 | Exon | ins-C | Protective | 0.38 | 2x10^-02^ | Turkish-Japanese | (Deng et al., 2018) |
| *CCR3* | rs7649764 | Downstream | C | Protective | 0.69 | 2.2x10^-02^ | Han Chinese | (Hou et al., 2012b) |
| *IL1* | rs1143634 | Exon | T | Susceptible | 1.74 | 2.4x10^-02^ | Turkish | (Zhou et al., 2012) |
| *IL23R* | rs1343151 | Intron | T | Protective | 0.50 | 2.9x10^-02^ | Han Chinese | (Jiang et al., 2010) |
| *TNFAIP3* | rs610604 | Intron | A | Protective | 0.80 | 5.5x10^-02^ | Han Chinese | (Li et al., 2013) |
| *IL6* | VNTR | Downstream | C | Susceptible | 3.45 | 4x10^-02^ | Korean | (Chang et al., 2005) |

Notes: Although over seventy publications that reported genetic associations with BD were found in the literature, redundant findings are not included in this final list. For candidate gene studies, only genetic associations that were replicated in at least two independent studies, and the result based on the largest sample size were included the final list. P values are the statistical associations reported in the original publications.

VNTR: Variable number tandem repeat ; ins-C: Insertion of C nucleotide

**References:**

Akman, A., Ekinci, N. C., Kacaroglu, H., Yavuzer, U., Alpsoy, E. & Yegin, O. (2008). Relationship between periodontal findings and specific polymorphisms of interleukin-1alpha and -1beta in Turkish patients with Behcet's disease. *Arch Dermatol Res,* 300**,** 19-26.

Chang, H. K., Jang, W. C., Park, S. B., Han, S. M., Nam, Y. H., Lee, S. S., et al. (2005). Association between interleukin 6 gene polymorphisms and Behcet's disease in Korean people. *Ann Rheum Dis,* 64**,** 339-40.

Deng, Y., Zhu, W. & Zhou, X. (2018). Immune Regulatory Genes Are Major Genetic Factors to Behcet Disease: Systematic Review. *Open Rheumatol J,* 12**,** 70-85.

Fei, Y., Webb, R., Cobb, B. L., Direskeneli, H., Saruhan-Direskeneli, G. & Sawalha, A. H. (2009). Identification of novel genetic susceptibility loci for Behcet's disease using a genome-wide association study. *Arthritis Res Ther,* 11**,** R66.

Hou, S., Shu, Q., Jiang, Z., Chen, Y., Li, F., Chen, F., et al. (2012a). Replication study confirms the association between UBAC2 and Behcet's disease in two independent Chinese sets of patients and controls. *Arthritis Res Ther,* 14**,** R70.

Hou, S., Xiao, X., Li, F., Jiang, Z., Kijlstra, A. & Yang, P. (2012b). Two-stage association study in Chinese Han identifies two independent associations in CCR1/CCR3 locus as candidate for Behcet's disease susceptibility. *Hum Genet,* 131**,** 1841-50.

Hou, S., Yang, Z., Du, L., Jiang, Z., Shu, Q., Chen, Y., et al. (2012c). Identification of a susceptibility locus in STAT4 for Behcet's disease in Han Chinese in a genome-wide association study. *Arthritis Rheum,* 64**,** 4104-13.

Jang, W. C., Nam, Y. H., Ahn, Y. C., Lee, S. H., Park, S. H., Choe, J. Y., et al. (2008). Interleukin-17F gene polymorphisms in Korean patients with Behcet's disease. *Rheumatol Int,* 29**,** 173-8.

Jiang, Z., Yang, P., Hou, S., Du, L., Xie, L., Zhou, H., et al. (2010). IL-23R gene confers susceptibility to Behcet's disease in a Chinese Han population. *Ann Rheum Dis,* 69**,** 1325-8.

Kirino, Y., Bertsias, G., Ishigatsubo, Y., Mizuki, N., Tugal-Tutkun, I., Seyahi, E., et al. (2013a). Genome-wide association analysis identifies new susceptibility loci for Behcet's disease and epistasis between HLA-B*51 and ERAP1. *Nat Genet,* 45**,** 202-7.

Kirino, Y., Zhou, Q., Ishigatsubo, Y., Mizuki, N., Tugal-Tutkun, I., Seyahi, E., et al. (2013b). Targeted resequencing implicates the familial Mediterranean fever gene MEFV and the toll-like receptor 4 gene TLR4 in Behcet disease. *Proc Natl Acad Sci U S A,* 110**,** 8134-9.

Lee, Y. J., Horie, Y., Wallace, G. R., Choi, Y. S., Park, J. A., Choi, J. Y., et al. (2013). Genome-wide association study identifies GIMAP as a novel susceptibility locus for Behcet's disease. *Ann Rheum Dis,* 72**,** 1510-6.

Li, H., Liu, Q., Hou, S., Du, L., Zhou, Q., Zhou, Y., et al. (2013). TNFAIP3 gene polymorphisms confer risk for Behcet's disease in a Chinese Han population. *Hum Genet,* 132**,** 293-300.

Mizuki, N., Meguro, A., Ota, M., Ohno, S., Shiota, T., Kawagoe, T., et al. (2010). Genome-wide association studies identify IL23R-IL12RB2 and IL10 as Behcet's disease susceptibility loci. *Nat Genet,* 42**,** 703-6.

Remmers, E. F., Cosan, F., Kirino, Y., Ombrello, M. J., Abaci, N., Satorius, C., et al. (2010). Genome-wide association study identifies variants in the MHC class I, IL10, and IL23R-IL12RB2 regions associated with Behcet's disease. *Nat Genet,* 42**,** 698-702.

Sawalha, A. H., Hughes, T., Nadig, A., Yilmaz, V., Aksu, K., Keser, G., et al. (2011). A putative functional variant within the UBAC2 gene is associated with increased risk of Behcet's disease. *Arthritis Rheum,* 63**,** 3607-12.

Takeuchi, M., Kastner, D. L. & Remmers, E. F. (2015). The immunogenetics of Behcet's disease: A comprehensive review. *J Autoimmun,* 64**,** 137-48.

Zhou, Z. Y., Chen, S. L., Shen, N. & Lu, Y. (2012). Cytokines and Behcet's disease. *Autoimmun Rev,* 11**,** 699-704.

**Supplemental Table 2**. Gene ontology analyses of Behcet disease associated genes

| **Gene** | **Family Name** | **GO-Slim Molecular Function** | **GO-Slim Biological Process** | **GO-Slim Cellular Component** | **Panther Protein Class** |
| --- | --- | --- | --- | --- | --- |
| ABCB5 | ATP-BINDING CASSETTE SUB-FAMILY B MEMBER 5 (PTHR24221:SF217) | ATPase activity(GO:0016887);phospholipid transporter activity(GO:0005548);ATPase-coupled intramembrane lipid transporter activity(GO:0140326);active transmembrane transporter activity(GO:0022804) | transmembrane transport(GO:0055085);lipid translocation(GO:0034204);amide transport(GO:0042886) | integral component of membrane(GO:0016021) | ATP-binding cassette (ABC) transporter(PC00003) |
| API5 | APOPTOSIS INHIBITOR 5 (PTHR12758:SF23) | RNA binding(GO:0003723) | negative regulation of apoptotic process(GO:0043066);apoptotic process(GO:0006915) | nucleus(GO:0005634) |  |
| ASB18 | ANKYRIN REPEAT AND SOCS BOX PROTEIN 18 (PTHR24118:SF76) |  |  |  | membrane traffic protein(PC00150) |
| ATP8A1 | PHOSPHOLIPID-TRANSPORTING ATPASE IA (PTHR24092:SF150) | ATPase activity(GO:0016887);ATPase-coupled intramembrane lipid transporter activity(GO:0140326) | phospholipid translocation(GO:0045332) | plasma membrane(GO:0005886) | primary active transporter(PC00068) |
| BAG6 | LARGE PROLINE-RICH PROTEIN BAG6 (PTHR15204:SF0) | polyubiquitin modification-dependent protein binding(GO:0031593) | ubiquitin-dependent ERAD pathway(GO:0030433) | cytosol(GO:0005829);protein-containing complex(GO:0032991) | chaperone(PC00072) |
| BTNL2 | BTNL2-RELATED (PTHR24100:SF105) | signaling receptor binding(GO:0005102) | immune response(GO:0006955);cytokine production(GO:0001816);regulation of cytokine production(GO:0001817);T cell receptor signaling pathway(GO:0050852) | leaflet of membrane bilayer(GO:0097478);external side of plasma membrane(GO:0009897) | immunoglobulin receptor superfamily(PC00124) |
| C10orf11 (LRMDA) | LEUCINE-RICH MELANOCYTE DIFFERENTIATION-ASSOCIATED PROTEIN (PTHR46282:SF2) |  |  |  |  |
| C6orf10 (TSBP1) | TESTIS-EXPRESSED BASIC PROTEIN 1 | nucleus (GO:0005634), integral component of membrane (GO:0016021) |  |  |  |
| C6orf15 | MUCIN 18B, ISOFORM A (PTHR15817:SF2) |  |  |  |  |
| C6orf47 | SI:CH73-25F10.6 (PTHR14307:SF0) |  |  |  |  |
| CCDC180 | COILED-COIL DOMAIN-CONTAINING PROTEIN 180 (PTHR21444:SF14) |  |  |  |  |
| CCHCR1 | COILED-COIL ALPHA-HELICAL ROD PROTEIN 1 (PTHR46822:SF1) |  | protein export from nucleus(GO:0006611) | centriole(GO:0005814) |  |
| CCR1 | C-C CHEMOKINE RECEPTOR TYPE 1 (PTHR10489:SF711) | C-C chemokine binding(GO:0019957) | inflammatory response(GO:0006954);immune response(GO:0006955) | cytoplasm(GO:0005737) |  |
| CCR3 | C-C CHEMOKINE RECEPTOR TYPE 3 (PTHR10489:SF649) |  | inflammatory response(GO:0006954);immune response(GO:0006955) | cytoplasm(GO:0005737) |  |
| CDH26 | CADHERIN-LIKE PROTEIN 26 (PTHR24027:SF78) | cadherin binding(GO:0045296);calcium ion binding(GO:0005509) | cell-cell junction assembly(GO:0007043);multicellular organism development(GO:0007275);cell morphogenesis(GO:0000902);cell-cell adhesion via plasma-membrane adhesion molecules(GO:0098742) | adherens junction(GO:0005912);plasma membrane protein complex(GO:0098797);extrinsic component of plasma membrane(GO:0019897) | cadherin(PC00057) |
| CEP135 | CENTROSOMAL PROTEIN OF 135 KDA (PTHR23159:SF18) |  |  |  | chromatin/chromatin-binding, or -regulatory protein(PC00077) |
| COL12A1 | COLLAGEN ALPHA-1(XII) CHAIN (PTHR24020:SF17) |  | cell differentiation(GO:0030154);ectoderm formation(GO:0001705);endoderm formation(GO:0001706);mesoderm formation(GO:0001707) | collagen-containing extracellular matrix(GO:0062023) | extracellular matrix structural protein(PC00103) |
| CTLA4 | CYTOTOXIC T-LYMPHOCYTE PROTEIN 4 (PTHR11494:SF8) |  | T cell differentiation (GO:0030217), regulation of hemopoiesis (GO:1903706), immune response (GO:0006955), regulation of cell differentiation (GO:0045595), negative regulation of multicellular organismal process (GO:0051241), negative regulation of T cell activation (GO:0050868), T cell receptor signaling pathway (GO:0050852), leukocyte cell-cell adhesion (GO:0007159), B cell receptor signaling pathway (GO:0050853) | leaflet of membrane bilayer (GO:0097478), external side of plasma membrane (GO:0009897) | immunoglobulin receptor superfamily (PC00124) |
| CPLX1 | COMPLEXIN-1 (PTHR16705:SF6) | syntaxin-1 binding(GO:0017075) | regulation of neurotransmitter secretion(GO:0046928);vesicle fusion to plasma membrane(GO:0099500);synaptic vesicle exocytosis(GO:0016079) | terminal bouton(GO:0043195);SNARE complex(GO:0031201);plasma membrane region(GO:0098590) |  |
| CPVL | SERINE CARBOXYPEPTIDASE CPVL-RELATED (PTHR11802:SF98) | carboxypeptidase activity(GO:0004180);serine-type peptidase activity(GO:0008236) |  |  | serine protease(PC00203) |
| CTNNA2 | CATENIN ALPHA-2 (PTHR18914:SF23) |  |  |  | non-motor actin binding protein(PC00165) |
| DEPDC1 | DEP DOMAIN-CONTAINING PROTEIN 1A (PTHR16206:SF12) |  |  |  | scaffold/adaptor protein(PC00226) |
| DHFRP2 | dihydrofolate reductase pseudogene 2 |  |  |  |  |
| DNMT3A | DNA (CYTOSINE-5)-METHYLTRANSFERASE 3A (PTHR23068:SF10) |  |  |  | DNA methyltransferase(PC00013) |
| DTL | DENTICLELESS PROTEIN HOMOLOG (PTHR22852:SF0) |  |  |  |  |
| EBF2 | TRANSCRIPTION FACTOR COE2 (PTHR10747:SF33) | RNA polymerase II cis-regulatory region sequence-specific DNA binding(GO:0000978);DNA-binding transcription factor activity, RNA polymerase II-specific(GO:0000981) | transcription by RNA polymerase II(GO:0006366);regulation of transcription by RNA polymerase II(GO:0006357) | nuclear chromatin(GO:0000790) | DNA-binding transcription factor(PC00218) |
| ERAP1 | ENDOPLASMIC RETICULUM AMINOPEPTIDASE 1 (PTHR11533:SF156) | zinc ion binding(GO:0008270);peptide binding(GO:0042277);metallopeptidase activity(GO:0008237) | peptide metabolic process(GO:0006518);proteolysis(GO:0006508);organonitrogen compound catabolic process(GO:1901565);cellular catabolic process(GO:0044248) | cytoplasm(GO:0005737) | metalloprotease(PC00153) |
| FUT2 | GALACTOSIDE ALPHA-(1,2)-FUCOSYLTRANSFERASE 2 (PTHR11927:SF2) | fucosyltransferase activity(GO:0008417) | protein glycosylation(GO:0006486) |  | glycosyltransferase(PC00111) |
| GABBR1 | GAMMA-AMINOBUTYRIC ACID TYPE B RECEPTOR SUBUNIT 1 (PTHR10519:SF73) | G protein-coupled receptor activity(GO:0004930) | G protein-coupled receptor signaling pathway(GO:0007186) | integral component of plasma membrane(GO:0005887);plasma membrane protein complex(GO:0098797);receptor complex(GO:0043235) | G-protein coupled receptor(PC00021) |
| GALNT1 | POLYPEPTIDE N-ACETYLGALACTOSAMINYLTRANSFERASE 1 (PTHR11675:SF123) |  |  | Golgi apparatus(GO:0005794);vacuole(GO:0005773);plasma membrane(GO:0005886) | glycosyltransferase(PC00111) |
| GALNT10 | POLYPEPTIDE N-ACETYLGALACTOSAMINYLTRANSFERASE 10 (PTHR11675:SF41) |  |  | Golgi apparatus(GO:0005794);vacuole(GO:0005773);plasma membrane(GO:0005886) | glycosyltransferase(PC00111) |
| GAS2 | GROWTH ARREST-SPECIFIC PROTEIN 2 (PTHR46756:SF9) | actin filament binding(GO:0051015);protein-macromolecule adaptor activity(GO:0030674);microtubule binding(GO:0008017) | actin filament organization(GO:0007015);microtubule cytoskeleton organization(GO:0000226) | actin filament(GO:0005884);microtubule(GO:0005874) |  |
| GIMAP1 | GTPASE IMAP FAMILY MEMBER 1 (PTHR10903:SF74) |  |  | endoplasmic reticulum(GO:0005783);vacuole(GO:0005773);plasma membrane(GO:0005886) | small GTPase(PC00208) |
| GIMAP2 | GTPASE IMAP FAMILY MEMBER 2 (PTHR10903:SF7) |  |  | endoplasmic reticulum(GO:0005783);vacuole(GO:0005773);plasma membrane(GO:0005886) | small GTPase(PC00208) |
| GIMAP4 | GTPASE IMAP FAMILY MEMBER 4 (PTHR10903:SF62) |  |  |  | small GTPase(PC00208) |
| HCG27 |  |  |  |  |  |
| HCG9 |  |  |  |  |  |
| HERPUD2 | HOMOCYSTEINE-RESPONSIVE ENDOPLASMIC RETICULUM-RESIDENT UBIQUITIN-LIKE DOMAIN MEMBER 2 PROTEIN (PTHR12943:SF5) |  | endoplasmic reticulum unfolded protein response(GO:0030968) |  |  |
| HIVEP3 | TRANSCRIPTION FACTOR HIVEP3 (PTHR45944:SF5) | RNA polymerase II cis-regulatory region sequence-specific DNA binding(GO:0000978);DNA-binding transcription factor activity, RNA polymerase II-specific(GO:0000981) | transcription by RNA polymerase II(GO:0006366);regulation of transcription by RNA polymerase II(GO:0006357) | nucleus(GO:0005634) |  |
| HLA-B | HLA CLASS I HISTOCOMPATIBILITY ANTIGEN, B ALPHA CHAIN (PTHR16675:SF251) |  | T cell mediated immunity(GO:0002456);positive regulation of adaptive immune response(GO:0002821);positive regulation of cellular process(GO:0048522);antigen processing and presentation(GO:0019882);positive regulation of lymphocyte mediated immunity(GO:0002708);cellular process(GO:0009987) | extracellular space(GO:0005615);leaflet of membrane bilayer(GO:0097478);external side of plasma membrane(GO:0009897) | major histocompatibility complex protein(PC00149) |
| HLA-C | HLA CLASS I HISTOCOMPATIBILITY ANTIGEN, C ALPHA CHAIN (PTHR16675:SF252) | peptide binding(GO:0042277);signaling receptor binding(GO:0005102) | T cell mediated immunity(GO:0002456);positive regulation of adaptive immune response(GO:0002821);positive regulation of cellular process(GO:0048522);antigen processing and presentation(GO:0019882);positive regulation of lymphocyte mediated immunity(GO:0002708);cellular process(GO:0009987) | extracellular space(GO:0005615);leaflet of membrane bilayer(GO:0097478);external side of plasma membrane(GO:0009897) | major histocompatibility complex protein(PC00149) |
| HLA-DQ |  | antigen processing and presentation of peptide or polysaccharide antigen via MHC class II(GO:0002504);cellular defense response(GO:0006968) | major histocompatibility complex antigen(PC00149) | T cell activation->MHC-antigen;; |  |
| HLA-DQA1 | HLA CLASS II HISTOCOMPATIBILITY ANTIGEN, DQ ALPHA 1 CHAIN (PTHR19944:SF59) |  |  |  | major histocompatibility complex protein(PC00149) |
| HLA-DQA2 | HLA CLASS II HISTOCOMPATIBILITY ANTIGEN, DQ ALPHA 2 CHAIN (PTHR19944:SF94) |  |  |  | major histocompatibility complex protein(PC00149) |
| HLA-DQB1 |  | antigen processing and presentation(GO:0019882) | immunoglobulin receptor superfamily(PC00124);major histocompatibility complex antigen(PC00149) |  |  |
| HLA-F | HLA CLASS I HISTOCOMPATIBILITY ANTIGEN, ALPHA CHAIN F (PTHR16675:SF187) | peptide binding(GO:0042277);signaling receptor binding(GO:0005102) | T cell mediated immunity(GO:0002456);positive regulation of adaptive immune response(GO:0002821);positive regulation of cellular process(GO:0048522);antigen processing and presentation(GO:0019882);positive regulation of lymphocyte mediated immunity(GO:0002708);cellular process(GO:0009987) | extracellular space(GO:0005615);leaflet of membrane bilayer(GO:0097478);external side of plasma membrane(GO:0009897) | major histocompatibility complex protein(PC00149) |
| HLA-G | HLA CLASS I HISTOCOMPATIBILITY ANTIGEN, ALPHA CHAIN G (PTHR16675:SF169) | peptide binding(GO:0042277);signaling receptor binding(GO:0005102) | T cell mediated immunity(GO:0002456);positive regulation of adaptive immune response(GO:0002821);positive regulation of cellular process(GO:0048522);antigen processing and presentation(GO:0019882);positive regulation of lymphocyte mediated immunity(GO:0002708);cellular process(GO:0009987) | extracellular space(GO:0005615);leaflet of membrane bilayer(GO:0097478);external side of plasma membrane(GO:0009897) | major histocompatibility complex protein(PC00149) |
| HMP19 | NEURONAL VESICLE TRAFFICKING-ASSOCIATED PROTEIN 2 (PTHR28546:SF2) | clathrin binding(GO:0030276) | cellular protein-containing complex assembly(GO:0034622);endosomal transport(GO:0016197) | endosome(GO:0005768);integral component of membrane(GO:0016021);vacuole(GO:0005773);plasma membrane(GO:0005886) |  |
| HNF4G | HEPATOCYTE NUCLEAR FACTOR 4-GAMMA (PTHR24083:SF42) | RNA polymerase II cis-regulatory region sequence-specific DNA binding(GO:0000978);DNA-binding transcription factor activity, RNA polymerase II-specific(GO:0000981) | anatomical structure development(GO:0048856);cell differentiation(GO:0030154);transcription by RNA polymerase II(GO:0006366);regulation of transcription by RNA polymerase II(GO:0006357) | nuclear chromatin(GO:0000790) | C4 zinc finger nuclear receptor(PC00169) |
| IFNG | INTERFERON GAMMA (PTHR11419:SF0) |  |  |  | interferon superfamily(PC00127) |
| IL10 | Interkulin-10 |  | interleukin superfamily(PC00128) | Interleukin signaling pathway->Interleukin;; |  |
| IL12A | Interkulin-12 |  | interleukin superfamily(PC00128) | Interleukin signaling pathway->Interleukin;; |  |
| IL17A | Interkulin-17 |  | interleukin superfamily(PC00128) | Interleukin signaling pathway->Interleukin;; |  |
| IL1A | INTERLEUKIN-1 ALPHA (PTHR10078:SF33) |  | T cell differentiation(GO:0030217);inflammatory response(GO:0006954);cellular response to lipopolysaccharide(GO:0071222);cytokine-mediated signaling pathway(GO:0019221);positive regulation of T cell differentiation(GO:0045582);positive regulation of gene expression(GO:0010628);T cell proliferation(GO:0042098);regulation of catalytic activity(GO:0050790);gene expression(GO:0010467);oxidation-reduction process(GO:0055114);positive regulation of T cell proliferation(GO:0042102);leukocyte cell-cell adhesion(GO:0007159) | extracellular space(GO:0005615) | interleukin superfamily(PC00128) |
| IL23R | INTERLEUKIN-23 RECEPTOR (PTHR23036:SF112) | growth factor receptor binding(GO:0070851);cytokine binding(GO:0019955);cytokine receptor activity(GO:0004896);cytokine receptor binding(GO:0005126) | cytokine-mediated signaling pathway(GO:0019221) | integral component of plasma membrane(GO:0005887);leaflet of membrane bilayer(GO:0097478);plasma membrane protein complex(GO:0098797);external side of plasma membrane(GO:0009897);receptor complex(GO:0043235) | transmembrane signal receptor(PC00197) |
| IL6 | INTERLEUKIN-6 (PTHR10511:SF3) |  |  |  |  |
| KCNK9 | POTASSIUM CHANNEL SUBFAMILY K MEMBER 9 (PTHR11003:SF75) | potassium channel activity(GO:0005267) | potassium ion transmembrane transport(GO:0071805);regulation of membrane potential(GO:0042391) | integral component of plasma membrane(GO:0005887) | ion channel(PC00133) |
| KLRC4 | NKG2-F TYPE II INTEGRAL MEMBRANE PROTEIN (PTHR22800:SF185) |  |  |  |  |
| KLRK1 | NKG2-D TYPE II INTEGRAL MEMBRANE PROTEIN (PTHR47494:SF1) |  |  |  |  |
| LILRA1 | LEUKOCYTE IMMUNOGLOBULIN-LIKE RECEPTOR SUBFAMILY A MEMBER 1-RELATED (PTHR11738:SF165) |  |  |  | immunoglobulin receptor superfamily(PC00124) |
| LILRB1 | LEUKOCYTE IMMUNOGLOBULIN-LIKE RECEPTOR SUBFAMILY A MEMBER 1-RELATED (PTHR11738:SF165) |  |  |  | immunoglobulin receptor superfamily(PC00124) |
| LOC100129342 |  |  |  |  |  |
| LOC100132252 |  |  |  |  |  |
| LOC107984355 |  |  |  |  |  |
| LOC285830 |  |  |  |  |  |
| LTN1 | E3 UBIQUITIN-PROTEIN LIGASE LISTERIN (PTHR12389:SF0) | ubiquitin protein ligase activity(GO:0061630);ribonucleoprotein complex binding(GO:0043021) | regulation of translation(GO:0006417);proteasome-mediated ubiquitin-dependent protein catabolic process(GO:0043161);translational elongation(GO:0006414) | cytosol(GO:0005829);protein-containing complex(GO:0032991) | ubiquitin-protein ligase(PC00234) |
| LYST | LYSOSOMAL-TRAFFICKING REGULATOR (PTHR13743:SF86) | protein kinase binding(GO:0019901) | protein localization(GO:0008104) | membrane(GO:0016020);cytosol(GO:0005829) |  |
| MEFV | PYRIN (PTHR24103:SF606) | ubiquitin protein ligase activity(GO:0061630) | protein ubiquitination(GO:0016567) | cytoplasm(GO:0005737) | ubiquitin-protein ligase(PC00234) |
| MICA | MHC CLASS I POLYPEPTIDE-RELATED SEQUENCE A-RELATED |  |  |  |  |
| MN1 | TRANSCRIPTIONAL ACTIVATOR MN1 (PTHR15821:SF0) |  |  |  |  |
| MOG | MYELIN-OLIGODENDROCYTE GLYCOPROTEIN (PTHR24100:SF71) | signaling receptor binding(GO:0005102) | immune response(GO:0006955);cytokine production(GO:0001816);regulation of cytokine production(GO:0001817);T cell receptor signaling pathway(GO:0050852) | leaflet of membrane bilayer(GO:0097478);external side of plasma membrane(GO:0009897) | immunoglobulin receptor superfamily(PC00124) |
| MSX2 | HOMEOBOX PROTEIN MSX-2 (PTHR24338:SF10) | RNA polymerase II transcription regulatory region sequence-specific DNA binding(GO:0000977);DNA-binding transcription factor activity, RNA polymerase II-specific(GO:0000981) | embryonic morphogenesis(GO:0048598) | nucleus(GO:0005634) | homeodomain transcription factor(PC00119) |
| MUC21 | MUCIN-21 (PTHR39408:SF3) |  |  |  |  |
| NAV2 | NEURON NAVIGATOR 2 (PTHR12784:SF6) |  | nervous system development(GO:0007399) |  |  |
| NOD2 | NUCLEOTIDE-BINDING OLIGOMERIZATION DOMAIN-CONTAINING PROTEIN 2 (PTHR24106:SF64) |  | transcription, DNA-templated(GO:0006351);positive regulation of I-kappaB kinase/NF-kappaB signaling(GO:0043123);response to peptide(GO:1901652);I-kappaB kinase/NF-kappaB signaling(GO:0007249);defense response to bacterium(GO:0042742);positive regulation of NF-kappaB transcription factor activity(GO:0051092) | cytosol(GO:0005829) | scaffold/adaptor protein(PC00226) |
| OSR1 | PROTEIN ODD-SKIPPED-RELATED 1 (PTHR14196:SF5) | RNA polymerase II transcription regulatory region sequence-specific DNA binding(GO:0000977);DNA-binding transcription factor activity, RNA polymerase II-specific(GO:0000981) | embryo development(GO:0009790);urogenital system development(GO:0001655);transcription by RNA polymerase II(GO:0006366);regulation of transcription by RNA polymerase II(GO:0006357) | nucleus(GO:0005634) | zinc finger transcription factor(PC00244) |
| OVCH1 | OVOCHYMASE-1 (PTHR24251:SF21) |  |  |  | serine protease(PC00203) |
| PAX8 | PAIRED BOX PROTEIN PAX-8 (PTHR45636:SF6) | RNA polymerase II cis-regulatory region sequence-specific DNA binding(GO:0000978);DNA-binding transcription factor activity, RNA polymerase II-specific(GO:0000981) | anatomical structure development(GO:0048856);transcription by RNA polymerase II(GO:0006366);regulation of transcription by RNA polymerase II(GO:0006357) | nuclear chromatin(GO:0000790) |  |
| PLEKHB1 | PLECKSTRIN HOMOLOGY DOMAIN-CONTAINING FAMILY B MEMBER 1 (PTHR14309:SF7) |  | regulation of cell differentiation(GO:0045595);cell differentiation(GO:0030154) | integral component of membrane(GO:0016021) |  |
| PMFBP1 | POLYAMINE-MODULATED FACTOR 1-BINDING PROTEIN 1 (PTHR18881:SF2) |  |  |  |  |
| POU5F1 | POU DOMAIN, CLASS 5, TRANSCRIPTION FACTOR 1 (PTHR11636:SF86) | RNA polymerase II cis-regulatory region sequence-specific DNA binding(GO:0000978);DNA-binding transcription factor activity, RNA polymerase II-specific(GO:0000981) | transcription by RNA polymerase II(GO:0006366);regulation of transcription by RNA polymerase II(GO:0006357) | nuclear chromatin(GO:0000790) |  |
| PPP1R11 | E3 UBIQUITIN-PROTEIN LIGASE PPP1R11 (PTHR20835:SF4) | phosphoprotein phosphatase activity(GO:0004721);protein phosphatase inhibitor activity(GO:0004864);protein phosphatase 1 binding(GO:0008157) | negative regulation of phosphatase activity(GO:0010923);protein dephosphorylation(GO:0006470);negative regulation of protein dephosphorylation(GO:0035308);regulation of phosphoprotein phosphatase activity(GO:0043666) | nucleus(GO:0005634) | ubiquitin-protein ligase(PC00234) |
| PSMD14 | 26S PROTEASOME NON-ATPASE REGULATORY SUBUNIT 14 (PTHR10410:SF5) | thiol-dependent ubiquitin-specific protease activity(GO:0004843);proteasome binding(GO:0070628);metallopeptidase activity(GO:0008237) | protein deubiquitination(GO:0016579);proteasome-mediated ubiquitin-dependent protein catabolic process(GO:0043161) | proteasome regulatory particle, lid subcomplex(GO:0008541) | translation initiation factor(PC00224) |
| PSORS1C1 | Psoriasis susceptibility 1 candidate gene 1 protein;PSORS1C1;ortholog |  |  |  |  |
| PTPN22 | TYROSINE-PROTEIN PHOSPHATASE NON-RECEPTOR TYPE 22 (PTHR45983:SF1) | protein tyrosine phosphatase activity (GO:0004725) | peptidyl-tyrosine dephosphorylation (GO:0035335) | Nucleus cytoplasm (GO:0005737) |  |
| RALGAPA2 | RAL GTPASE-ACTIVATING PROTEIN SUBUNIT ALPHA-2 (PTHR10063:SF2) | GTPase activity(GO:0003924);GTPase activator activity(GO:0005096) |  | cytoplasm(GO:0005737) | GTPase-activating protein(PC00257) |
| RIMBP2 | RIMS-BINDING PROTEIN 2 (PTHR14234:SF18) |  | neuromuscular synaptic transmission(GO:0007274) |  |  |
| RNF39 | RING FINGER PROTEIN 39 (PTHR24103:SF646) | ubiquitin protein ligase activity(GO:0061630) | innate immune response(GO:0045087);regulation of gene expression(GO:0010468);gene expression(GO:0010467);protein ubiquitination(GO:0016567) | cytoplasm(GO:0005737) | ubiquitin-protein ligase(PC00234) |
| SACM1L | PHOSPHATIDYLINOSITOL-3-PHOSPHATASE SAC1 (PTHR45662:SF2) | phosphatase activity(GO:0016791) | phospholipid dephosphorylation(GO:0046839);phosphatidylinositol metabolic process(GO:0046488) | endoplasmic reticulum(GO:0005783);vacuole(GO:0005773);plasma membrane(GO:0005886) | phosphatase(PC00181) |
| SAMD3 | STERILE ALPHA MOTIF DOMAIN-CONTAINING PROTEIN 3 (PTHR47302:SF1) |  |  |  |  |
| SEMA6D | SEMAPHORIN-6D (PTHR11036:SF65) | receptor ligand activity(GO:0048018) | negative regulation of locomotion(GO:0040013);neural crest cell development(GO:0014032);negative regulation of cellular component movement(GO:0051271);negative regulation of neuron differentiation(GO:0045665);axon guidance(GO:0007411);cell surface receptor signaling pathway(GO:0007166);axon extension(GO:0048675);regulation of axonogenesis(GO:0050770);regulation of cell growth(GO:0001558);negative regulation of response to external stimulus(GO:0032102);negative regulation of cellular component organization(GO:0051129);regulation of cellular component size(GO:0032535);regulation of chemotaxis(GO:0050920);cell migration(GO:0016477);positive regulation of cell migration(GO:0030335) | integral component of plasma membrane(GO:0005887);extracellular space(GO:0005615) | membrane-bound signaling molecule(PC00152) |
| SGPP2 | SPHINGOSINE-1-PHOSPHATE PHOSPHATASE 2 (PTHR14969:SF14) | phosphatase activity(GO:0016791) | phospholipid dephosphorylation(GO:0046839);alcohol metabolic process(GO:0006066);sphingolipid metabolic process(GO:0006665) | endoplasmic reticulum membrane(GO:0005789);endoplasmic reticulum(GO:0005783);vacuole(GO:0005773);plasma membrane(GO:0005886) | phosphatase(PC00181) |
| SLC22A23 | SOLUTE CARRIER FAMILY 22 MEMBER 23 (PTHR24064:SF192) |  |  |  | secondary carrier transporter(PC00258) |
| SLC41A2 | SOLUTE CARRIER FAMILY 41 MEMBER 2 (PTHR16228:SF25) |  |  | plasma membrane(GO:0005886) | secondary carrier transporter(PC00258) |
| SLC43A3 | SOLUTE CARRIER FAMILY 43 MEMBER 3 (PTHR20765:SF1) |  |  |  | amino acid transporter(PC00046) |
| SLC44A4 | CHOLINE TRANSPORTER-LIKE PROTEIN 4 (PTHR12385:SF37) | organic cation transmembrane transporter activity(GO:0015101);organophosphate ester transmembrane transporter activity(GO:0015605);organic anion transmembrane transporter activity(GO:0008514) | choline transport(GO:0015871);anion transmembrane transport(GO:0098656);cation transmembrane transport(GO:0098655);organophosphate ester transport(GO:0015748);organic anion transport(GO:0015711) | plasma membrane(GO:0005886) | secondary carrier transporter(PC00258) |
| SLIT2 | SLIT HOMOLOG 2 PROTEIN | heparin binding(GO:0008201);signaling receptor binding(GO:0005102) |  |  |  |
| SMARCA2 | GLOBAL TRANSCRIPTION ACTIVATOR SNF2L2-RELATED (PTHR10799:SF541) | DNA binding(GO:0003677);DNA-dependent ATPase activity(GO:0008094);transcription factor binding(GO:0008134) | ATP-dependent chromatin remodeling(GO:0043044);transcription by RNA polymerase II(GO:0006366);positive regulation of transcription by RNA polymerase II(GO:0045944) | nucleus(GO:0005634) | DNA helicase(PC00011) |
| SMG6 | TELOMERASE-BINDING PROTEIN EST1A (PTHR15696:SF0) | telomerase RNA binding(GO:0070034);telomeric DNA binding(GO:0042162) | nuclear-transcribed mRNA catabolic process, nonsense-mediated decay(GO:0000184);gene expression(GO:0010467) | telomerase holoenzyme complex(GO:0005697) | RNA metabolism protein(PC00031) |
| SORBS2 | SORBIN AND SH3 DOMAIN-CONTAINING PROTEIN 2 (PTHR14167:SF56) |  |  |  |  |
| STAT4 | SIGNAL TRANSDUCER AND ACTIVATOR OF TRANSCRIPTION 4 (PTHR11801:SF19) | RNA polymerase II cis-regulatory region sequence-specific DNA binding(GO:0000978);DNA-binding transcription factor activity, RNA polymerase II-specific(GO:0000981) | cell population proliferation(GO:0008283);cytokine-mediated signaling pathway(GO:0019221);transcription by RNA polymerase II(GO:0006366);defense response(GO:0006952);response to peptide hormone(GO:0043434);regulation of cell population proliferation(GO:0042127);receptor signaling pathway via JAK-STAT(GO:0007259);regulation of transcription by RNA polymerase II(GO:0006357) | nuclear chromatin(GO:0000790) | DNA-binding transcription factor(PC00218) |
| STK39 | STE20/SPS1-RELATED PROLINE-ALANINE-RICH PROTEIN KINASE (PTHR48012:SF14) |  |  |  |  |
| STX8 | SYNTAXIN-8 (PTHR19957:SF322) | SNARE binding(GO:0000149);protein-macromolecule adaptor activity(GO:0030674) | organelle localization(GO:0051640);vesicle fusion(GO:0006906);intracellular protein transport(GO:0006886) | integral component of membrane(GO:0016021);SNARE complex(GO:0031201);endomembrane system(GO:0012505);vacuole(GO:0005773);plasma membrane(GO:0005886) | SNARE protein(PC00034) |
| SUMO4 | SMALL UBIQUITIN-RELATED MODIFIER 3-RELATED (PTHR10562:SF79) | ubiquitin-like protein ligase binding(GO:0044389) | protein sumoylation(GO:0016925) | nucleus(GO:0005634) |  |
| SUSD1 | SUSHI DOMAIN-CONTAINING PROTEIN 1 (PTHR24051:SF5) |  |  |  | extracellular matrix glycoprotein(PC00100) |
| TCF19 | TRANSCRIPTION FACTOR 19 (PTHR15464:SF1) |  | regulation of gene expression(GO:0010468);gene expression(GO:0010467) | nucleus(GO:0005634) | DNA-binding transcription factor(PC00218) |
| TENM4 | TENEURIN-4 (PTHR11219:SF9) | protein homodimerization activity(GO:0042803);cell adhesion molecule binding(GO:0050839) | cell-cell adhesion via plasma-membrane adhesion molecules(GO:0098742);neuron development(GO:0048666) | plasma membrane region(GO:0098590);neuron projection(GO:0043005) |  |
| TFCP2L1 | TRANSCRIPTION FACTOR CP2-LIKE PROTEIN 1 (PTHR11037:SF18) | RNA polymerase II cis-regulatory region sequence-specific DNA binding(GO:0000978);DNA-binding transcription activator activity, RNA polymerase II-specific(GO:0001228) | transcription by RNA polymerase II(GO:0006366);regulation of transcription by RNA polymerase II(GO:0006357) | nucleus(GO:0005634) | DNA-binding transcription factor(PC00218) |
| TLR4 | TOLL-LIKE RECEPTOR 4 (PTHR24365:SF521) | signaling receptor activity(GO:0038023);lipopolysaccharide binding(GO:0001530) | toll-like receptor signaling pathway(GO:0002224);inflammatory response(GO:0006954);defense response to Gram-negative bacterium(GO:0050829) | integral component of plasma membrane(GO:0005887) |  |
| TMEM132B | TRANSMEMBRANE PROTEIN 132B (PTHR13388:SF12) |  |  |  |  |
| TNF | TUMOR NECROSIS FACTOR (PTHR11471:SF23) | tumor necrosis factor receptor binding(GO:0005164);cytokine activity(GO:0005125) | tumor necrosis factor-mediated signaling pathway(GO:0033209);transcription by RNA polymerase II(GO:0006366);extrinsic apoptotic signaling pathway via death domain receptors(GO:0008625);positive regulation of transcription by RNA polymerase II(GO:0045944);positive regulation of NF-kappaB transcription factor activity(GO:0051092) | extracellular space(GO:0005615);cell surface(GO:0009986) |  |
| TNFAIP3 | TUMOR NECROSIS FACTOR ALPHA-INDUCED PROTEIN 3 (PTHR13367:SF3) | thiol-dependent ubiquitin-specific protease activity(GO:0004843);K63-linked polyubiquitin modification-dependent protein binding(GO:0070530) | protein deubiquitination(GO:0016579);cytoskeleton organization(GO:0007010);Wnt signaling pathway(GO:0016055);positive regulation of Wnt signaling pathway(GO:0030177);cell migration(GO:0016477);ubiquitin-dependent protein catabolic process(GO:0006511) | nucleus(GO:0005634);cytoplasm(GO:0005737) | cysteine protease(PC00081) |
| TRIM31 | E3 UBIQUITIN-PROTEIN LIGASE TRIM31 (PTHR24103:SF87) | ubiquitin protein ligase activity(GO:0061630) | innate immune response(GO:0045087);regulation of gene expression(GO:0010468);gene expression(GO:0010467);protein ubiquitination(GO:0016567) | cytoplasm(GO:0005737) | ubiquitin-protein ligase(PC00234) |
| TTLL7 | TUBULIN POLYGLUTAMYLASE TTLL7 (PTHR12241:SF147) | tubulin binding(GO:0015631);catalytic activity, acting on a protein(GO:0140096);ligase activity(GO:0016874) | peptidyl-amino acid modification(GO:0018193);microtubule cytoskeleton organization(GO:0000226) | cilium(GO:0005929);intraciliary transport particle(GO:0030990);plasma membrane region(GO:0098590) | microtubule or microtubule-binding cytoskeletal protein(PC00157) |
| UBAC2 | Ubiquitin-associated domain-containing protein 2;UBAC2;ortholog |  |  |  |  |
| UBASH3B | UBIQUITIN-ASSOCIATED AND SH3 DOMAIN-CONTAINING PROTEIN B (PTHR16469:SF29) | protein tyrosine phosphatase activity(GO:0004725) | transmembrane receptor protein tyrosine kinase signaling pathway(GO:0007169);regulation of bone resorption(GO:0045124);protein phosphorylation(GO:0006468);negative regulation of protein kinase activity(GO:0006469);osteoclast differentiation(GO:0030316);regulation of release of sequestered calcium ion into cytosol(GO:0051279);regulation of hemopoiesis(GO:1903706);negative regulation of signal transduction(GO:0009968);regulation of cell differentiation(GO:0045595);bone resorption(GO:0045453);negative regulation of multicellular organismal process(GO:0051241);sequestering of calcium ion(GO:0051208);release of sequestered calcium ion into cytosol(GO:0051209);peptidyl-tyrosine dephosphorylation(GO:0035335);platelet aggregation(GO:0070527) | cytoplasm(GO:0005737) |  |
| UBD | UBIQUITIN D (PTHR47731:SF1) | proteasome binding(GO:0070628) | transcription, DNA-templated(GO:0006351);innate immune response(GO:0045087);apoptotic process(GO:0006915);positive regulation of apoptotic process(GO:0043065);positive regulation of I-kappaB kinase/NF-kappaB signaling(GO:0043123);I-kappaB kinase/NF-kappaB signaling(GO:0007249);positive regulation of NF-kappaB transcription factor activity(GO:0051092);protein ubiquitination(GO:0016567);response to tumor necrosis factor(GO:0034612);ubiquitin-dependent protein catabolic process(GO:0006511) | nucleus(GO:0005634) |  |
| ZNRD1 | DNA-DIRECTED RNA POLYMERASE I SUBUNIT RPA12 (PTHR11239:SF14) | DNA-directed 5'-3' RNA polymerase activity(GO:0003899) | transcription by RNA polymerase I(GO:0006360) | RNA polymerase I complex(GO:0005736) | DNA-directed RNA polymerase(PC00019) |

**Supplemental Table 3.** Distribution of Behcet disease associated variants among 1000 Genomes population samples

1. Abbreviations of the 1000 Genomes populations

| **AFR** | **African** |
| --- | --- |
| ACB | African Caribbeans in Barbados |
| ASW | Americans of African Ancestry in SW USA |
| ESN | Esan in Nigeria |
| GWD | Gambian in Western Divisions in the Gambia |
| LWK | Luhya in Webuye, Kenya |
| MSL | Mende in Sierra Leone |
| YRI | Yoruba in Ibadan, Nigeria |
| **AMR** | **Ad Mixed American** |
| CLM | Colombians from Medellin, Colombia |
| MXL | Mexican Ancestry from Los Angeles USA |
| PEL | Peruvians from Lima, Peru |
| PUR | Puerto Ricans from Puerto Rico |
| **EAS** | **East Asian** |
| CDX | Chinese Dai in Xishuangbanna, China |
| CHB | Han Chinese in Bejing, China |
| CHS | Southern Han Chinese |
| JPT | Japanese in Tokyo, Japan |
| KHV | Kinh in Ho Chi Minh City, Vietnam |
| **EUR** | **European** |
| CEU | Utah Residents (CEPH) with Northern and Western European Ancestry |
| FIN | Finnish in Finland |
| GBR | British in England and Scotland |
| IBS | Iberian Population in Spain |
| TSI | Toscani in Italia |
| **SAS** | **South Asian** |
| BEB | Bengali from Bangladesh |
| GIH | Gujarati Indian from Houston, Texas |
| ITU | Indian Telugu from the UK |
| PJL | Punjabi from Lahore, Pakistan |
| STU | Sri Lankan Tamil from the UK |

1. 1000 Genomes African population samples

| **Gene** | **Variant/SNP** | **Behcet Allele** | **ALL** | **AFR** | ACB | ASW | ESN | GWD | LWK | MSL | YRI |
| --- | --- | --- | --- | --- | --- | --- | --- | --- | --- | --- | --- |
| *IL-10* | rs1518111 | A | 0.427 | 0.434 | 0.385 | 0.377 | 0.449 | 0.473 | 0.394 | 0.471 | 0.463 |
| *IL-10* | rs1800871 | T | 0.435 | 0.436 | 0.391 | 0.377 | 0.449 | 0.473 | 0.389 | 0.476 | 0.468 |
| *IL-10* | rs1800872 | A | 0.435 | 0.436 | 0.391 | 0.377 | 0.449 | 0.473 | 0.394 | 0.476 | 0.468 |
| *IL-10* | rs1554286 | C | 0.592 | 0.569 | 0.625 | 0.623 | 0.556 | 0.527 | 0.606 | 0.529 | 0.542 |
| *IL23R,IL12RB2* | rs1495965 | G | 0.466 | 0.422 | 0.422 | 0.484 | 0.409 | 0.363 | 0.419 | 0.471 | 0.426 |
| *IL23R,IL12RB2* | rs924080 | T | 0.604 | 0.495 | 0.484 | 0.541 | 0.530 | 0.403 | 0.515 | 0.547 | 0.481 |
| *IL23R,IL12RB2* | rs12119179 | A | 0.644 | 0.801 | 0.797 | 0.746 | 0.788 | 0.876 | 0.778 | 0.806 | 0.787 |
| *IL23R,IL12RB2* | rs11209033 | C | 0.644 | 0.797 | 0.792 | 0.746 | 0.783 | 0.876 | 0.773 | 0.806 | 0.778 |
| *IL23R,IL12RB2* | rs12141431 | C | 0.294 | 0.023 | 0.036 | 0.131 | 0.005 | 0.013 | 0.010 | 0 | 0.005 |
| *TNFAIP3* | rs9494885 | T | 0.794 | 0.477 | 0.536 | 0.639 | 0.505 | 0.363 | 0.551 | 0.447 | 0.384 |
| *TNFAIP3* | rs10499194 | C | 0.809 | 0.870 | 0.833 | 0.811 | 0.838 | 0.876 | 0.929 | 0.841 | 0.926 |
| *TNFAIP3* | rs610604 | A | 0.613 | 0.318 | 0.354 | 0.369 | 0.384 | 0.292 | 0.278 | 0.247 | 0.315 |
| *TNFAIP3* | rs7753873 | C | 0.191 | 0.463 | 0.458 | 0.295 | 0.449 | 0.522 | 0.409 | 0.441 | 0.579 |
| *STAT4* | rs7574070 | A | 0.494 | 0.734 | 0.688 | 0.623 | 0.828 | 0.704 | 0.753 | 0.759 | 0.750 |
| *STAT4* | rs897200 | A | 0.499 | 0.734 | 0.688 | 0.615 | 0.828 | 0.704 | 0.753 | 0.765 | 0.750 |
| *STAT4* | rs7572482 | A | 0.484 | 0.697 | 0.635 | 0.607 | 0.763 | 0.699 | 0.702 | 0.735 | 0.704 |
| *CCR1* | rs17282391 | G | 0.093 | 0.003 | 0.010 | 0.016 | 0 | 0 | 0 | 0 | 0 |
| *CCR1* | rs10510749 | T | 0.122 | 0.111 | 0.052 | 0.082 | 0.056 | 0.173 | 0.141 | 0.141 | 0.116 |
| *CCR1* | rs13084057 | G | 0.122 | 0.111 | 0.052 | 0.082 | 0.056 | 0.173 | 0.141 | 0.141 | 0.116 |
| *CCR1* | rs7631551 | A | 0.180 | 0.307 | 0.198 | 0.262 | 0.232 | 0.336 | 0.449 | 0.365 | 0.292 |
| *CCR1* | rs7616215 | T | 0.652 | 0.523 | 0.609 | 0.574 | 0.566 | 0.513 | 0.369 | 0.512 | 0.537 |
| *CCR3* | rs7649764 | C | 0.634 | 0.722 | 0.609 | 0.656 | 0.722 | 0.792 | 0.662 | 0.776 | 0.801 |
| *CCR3* | rs9990343 | G | 0.409 | 0.523 | 0.432 | 0.402 | 0.470 | 0.673 | 0.424 | 0.606 | 0.593 |
| *CCR3* | rs6803980 | A | 0.418 | 0.559 | 0.458 | 0.467 | 0.500 | 0.704 | 0.465 | 0.624 | 0.639 |
| *CCR3* | rs13075270 | C | 0.156 | 0.220 | 0.255 | 0.230 | 0.217 | 0.168 | 0.227 | 0.235 | 0.222 |
| *CCR3* | rs13092160 | C | 0.125 | 0.124 | 0.130 | 0.164 | 0.076 | 0.097 | 0.146 | 0.141 | 0.134 |
| *CCR3* | rs2373156 | T | 0.195 | 0.374 | 0.344 | 0.311 | 0.359 | 0.381 | 0.399 | 0.441 | 0.370 |
| *CCR3* | rs7651539 | T | 0.195 | 0.374 | 0.344 | 0.311 | 0.359 | 0.381 | 0.399 | 0.441 | 0.370 |
| *CCR3* | rs1542755 | A | 0.084 | 0.003 | 0.010 | 0.016 | 0 | 0 | 0 | 0 | 0 |
| *CCR3* | rs13067058 | A | 0.079 | 0.003 | 0.010 | 0.016 | 0 | 0 | 0 | 0 | 0 |
| *CCR3* | rs13092160 | C | 0.125 | 0.124 | 0.130 | 0.164 | 0.076 | 0.097 | 0.146 | 0.141 | 0.134 |
| *KLRC4* | rs2617170 | C | 0.557 | 0.433 | 0.474 | 0.516 | 0.434 | 0.341 | 0.455 | 0.365 | 0.477 |
| *MEFV* | rs61752717 | G | 0.0002 | 0 | 0 | 0 | 0 | 0 | 0 | 0 | 0 |
| *ERAP1* | rs17482078 | T | 0.101 | 0.054 | 0.073 | 0.074 | 0.035 | 0.031 | 0.040 | 0.071 | 0.065 |
| *FUT2* | rs681343 | T | 0.322 | 0.491 | 0.516 | 0.508 | 0.566 | 0.469 | 0.439 | 0.388 | 0.542 |
| *IL12A* | rs17810546 | A | 0.960 | 0.998 | 0.995 | 0.992 | 1.000 | 1.000 | 1.000 | 1.000 | 1.000 |
| *IL23R* | rs11209026 | A | 0.023 | 0.003 | 0 | 0.016 | 0 | 0 | 0.005 | 0 | 0.005 |
| *IL23R* | rs76418789 | A | 0.0112 | 0.0008 | 0 | 0.0082 | 0 | 0 | 0 | 0 | 0 |
| *IL23R* | rs17375018 | G | 0.694 | 0.794 | 0.812 | 0.795 | 0.854 | 0.752 | 0.692 | 0.818 | 0.843 |
| *IL23R* | rs11209032 | A | 0.354 | 0.198 | 0.198 | 0.246 | 0.222 | 0.124 | 0.212 | 0.194 | 0.218 |
| *IL23R* | rs1343151 | T | 0.338 | 0.728 | 0.703 | 0.598 | 0.788 | 0.730 | 0.712 | 0.771 | 0.750 |
| *TLR4* | rs4986790 | G | 0.060 | 0.071 | 0.042 | 0.057 | 0.051 | 0.128 | 0.096 | 0.076 | 0.037 |
| *TLR4* | rs4986791 | T | 0.041 | 0.005 | 0 | 0 | 0.010 | 0.018 | 0 | 0.006 | 0 |
| *NOD2* | rs2066844 | T | 0.014 | 0.002 | 0.005 | 0.016 | 0 | 0 | 0 | 0 | 0 |
| *NOD2* | rs2066845 | C | 0.005 | 0 | 0 | 0 | 0 | 0 | 0 | 0 | 0 |
| *NOD2* | rs2066847 | ins-C | 0.006 | 0.004 | 0.005 | 0.033 | 0 | 0 | 0 | 0 | 0 |
| *IL1* | rs1800587 | C | 0.721 | 0.596 | 0.568 | 0.664 | 0.641 | 0.531 | 0.606 | 0.641 | 0.565 |
| *IL1* | rs1143634 | T | 0.133 | 0.123 | 0.130 | 0.123 | 0.126 | 0.168 | 0.111 | 0.088 | 0.102 |
| *IL1* | rs16944 | G | 0.491 | 0.427 | 0.464 | 0.443 | 0.434 | 0.478 | 0.359 | 0.400 | 0.412 |
| *TNFα* | rs1799964 | C | 0.219 | 0.149 | 0.130 | 0.156 | 0.076 | 0.164 | 0.207 | 0.188 | 0.130 |
| *TNFα* | rs361525 | A | 0.061 | 0.038 | 0.016 | 0.041 | 0.010 | 0.084 | 0.061 | 0.047 | 0.005 |
| *TNFα* | rs1799724 | T | 0.099 | 0.024 | 0.031 | 0.049 | 0.025 | 0.022 | 0.010 | 0.024 | 0.019 |
| *IL12* | rs3212227 | A | 0.641 | 0.648 | 0.682 | 0.713 | 0.616 | 0.659 | 0.591 | 0.647 | 0.648 |
| *IL18* | rs1946518 | C | 0.592 | 0.650 | 0.661 | 0.672 | 0.662 | 0.606 | 0.657 | 0.635 | 0.667 |
| *IL17F-A126G* | rs2397084 | T | 0.967 | 0.997 | 0.995 | 0.992 | 1.000 | 1.000 | 0.990 | 1.000 | 1.000 |
| *LOC100129342* | rs11206377 | G | 0.540 | 0.274 | 0.271 | 0.320 | 0.308 | 0.319 | 0.212 | 0.271 | 0.231 |
| *CCDC180* | rs2061634 | G | 0.278 | 0.410 | 0.411 | 0.402 | 0.434 | 0.420 | 0.394 | 0.371 | 0.426 |
| *CPVL* | rs317711 | C | 0.173 | 0.203 | 0.177 | 0.246 | 0.212 | 0.146 | 0.222 | 0.247 | 0.199 |
| *UBASH3B* | rs4936742 | T | 0.389 | 0.163 | 0.203 | 0.230 | 0.136 | 0.124 | 0.182 | 0.159 | 0.144 |
| *UBAC2* | rs9513584 | G | 0.532 | 0.728 | 0.667 | 0.656 | 0.763 | 0.770 | 0.697 | 0.771 | 0.741 |
| *UBAC2* | rs9517644 | T | 0.526 | 0.705 | 0.641 | 0.631 | 0.758 | 0.743 | 0.677 | 0.753 | 0.704 |
| *UBAC2* | rs11069357 | A | 0.526 | 0.707 | 0.641 | 0.639 | 0.758 | 0.748 | 0.677 | 0.753 | 0.704 |
| *UBAC2* | rs984477 | G | 0.546 | 0.712 | 0.641 | 0.648 | 0.753 | 0.757 | 0.682 | 0.759 | 0.718 |
| *UBAC2* | rs9554573 | A | 0.559 | 0.809 | 0.745 | 0.746 | 0.874 | 0.810 | 0.773 | 0.788 | 0.894 |
| *UBAC2* | rs6491493 | G | 0.533 | 0.726 | 0.656 | 0.656 | 0.768 | 0.770 | 0.697 | 0.771 | 0.736 |
| *UBAC2* | rs9517668 | T | 0.266 | 0.404 | 0.385 | 0.369 | 0.545 | 0.319 | 0.369 | 0.424 | 0.417 |
| *UBAC2* | rs7999348 | G | 0.555 | 0.741 | 0.672 | 0.680 | 0.823 | 0.717 | 0.747 | 0.735 | 0.782 |
| *UBAC2* | rs9554581 | T | 0.192 | 0.135 | 0.099 | 0.139 | 0.167 | 0.159 | 0.111 | 0.171 | 0.102 |
| *UBAC2* | rs17575643 | T | 0.096 | 0.048 | 0.031 | 0.082 | 0.056 | 0.044 | 0.040 | 0.059 | 0.042 |
| *UBAC2* | rs727263 | A | 0.188 | 0.121 | 0.099 | 0.115 | 0.136 | 0.146 | 0.131 | 0.147 | 0.074 |
| *UBAC2* | rs7332161 | A | 0.189 | 0.121 | 0.104 | 0.115 | 0.131 | 0.146 | 0.131 | 0.147 | 0.074 |
| *UBAC2* | rs912130 | C | 0.527 | 0.705 | 0.635 | 0.631 | 0.717 | 0.765 | 0.712 | 0.729 | 0.708 |
| *UBAC2* | rs2892976 | G | 0.344 | 0.467 | 0.490 | 0.443 | 0.490 | 0.376 | 0.556 | 0.465 | 0.454 |
| *UBAC2* | rs3825427 | T | 0.194 | 0.135 | 0.104 | 0.139 | 0.167 | 0.159 | 0.121 | 0.159 | 0.102 |
| *UBAC2* | rs9517701 | G | 0.190 | 0.121 | 0.104 | 0.115 | 0.131 | 0.146 | 0.131 | 0.147 | 0.074 |
| *GIMAP4* | rs1916012 | T | 0.506 | 0.452 | 0.521 | 0.410 | 0.480 | 0.434 | 0.530 | 0.388 | 0.389 |
| *GIMAP4* | rs1522596 | T | 0.530 | 0.541 | 0.536 | 0.459 | 0.631 | 0.588 | 0.581 | 0.488 | 0.463 |
| *GIMAP4* | rs1608157 | C | 0.505 | 0.452 | 0.516 | 0.410 | 0.480 | 0.434 | 0.530 | 0.388 | 0.389 |
| *GIMAP2* | rs10266069 | A | 0.412 | 0.327 | 0.396 | 0.352 | 0.313 | 0.270 | 0.359 | 0.347 | 0.278 |
| *GIMAP2* | rs10256482 | T | 0.501 | 0.455 | 0.484 | 0.418 | 0.480 | 0.447 | 0.470 | 0.471 | 0.407 |
| *GIMAP1* | rs2286900 | T | 0.123 | 0.118 | 0.115 | 0.066 | 0.146 | 0.150 | 0.126 | 0.076 | 0.116 |
| *CPLX1* | rs11248047 | A | 0.440 | 0.424 | 0.438 | 0.434 | 0.485 | 0.394 | 0.343 | 0.359 | 0.505 |
| *DEPDC1* | rs6692084 | A | 0.293 | 0.495 | 0.432 | 0.500 | 0.515 | 0.544 | 0.480 | 0.535 | 0.463 |
| *DEPDC1* | rs12134670 | C | 0.068 | 0.012 | 0.026 | 0.008 | 0.005 | 0.009 | 0.015 | 0.012 | 0.009 |
| *DTL* | rs1472224 | G | 0.452 | 0.592 | 0.552 | 0.557 | 0.662 | 0.606 | 0.551 | 0.553 | 0.634 |
| *DNMT3A* | rs1465825 | C | 0.364 | 0.405 | 0.411 | 0.385 | 0.308 | 0.478 | 0.318 | 0.500 | 0.426 |
| *TFCP2L1* | rs17006292 | A | 0.048 | 0.118 | 0.156 | 0.090 | 0.101 | 0.093 | 0.172 | 0.106 | 0.102 |
| *PSMD14* | rs6744214 | T | 0.374 | 0.368 | 0.302 | 0.393 | 0.414 | 0.332 | 0.379 | 0.412 | 0.366 |
| *PSMD14* | rs6733456 | C | 0.428 | 0.582 | 0.542 | 0.557 | 0.576 | 0.593 | 0.510 | 0.676 | 0.616 |
| *STK39* | rs2390639 | A | 0.639 | 0.632 | 0.661 | 0.598 | 0.657 | 0.522 | 0.712 | 0.671 | 0.616 |
| *STK39* | rs3769393 | G | 0.697 | 0.720 | 0.734 | 0.689 | 0.758 | 0.664 | 0.803 | 0.682 | 0.704 |
| *SGPP2* | rs17562982 | T | 0.368 | 0.288 | 0.260 | 0.320 | 0.298 | 0.270 | 0.359 | 0.282 | 0.245 |
| *ASB18* | rs7561555 | C | 0.437 | 0.822 | 0.781 | 0.639 | 0.828 | 0.863 | 0.833 | 0.859 | 0.875 |
| *SLIT2* | rs13435197 | A | 0.311 | 0.389 | 0.417 | 0.385 | 0.389 | 0.442 | 0.369 | 0.365 | 0.347 |
| *SORBS2* | rs4493590 | G | 0.174 | 0.038 | 0.031 | 0.057 | 0.030 | 0.062 | 0.025 | 0.041 | 0.023 |
| *MSX2* | rs10516130 | A | 0.268 | 0.486 | 0.438 | 0.467 | 0.495 | 0.496 | 0.510 | 0.535 | 0.463 |
| *C6orf85(LOC100507336)* | rs12194547 | C | 0.087 | 0.115 | 0.115 | 0.082 | 0.146 | 0.106 | 0.066 | 0.141 | 0.139 |
| *ABCB5* | rs2190411 | C | 0.235 | 0.260 | 0.250 | 0.303 | 0.278 | 0.248 | 0.116 | 0.329 | 0.319 |
| *SUSD1* | rs2782932 | T | 0.140 | 0.009 | 0.036 | 0.033 | 0 | 0 | 0 | 0 | 0.005 |
| *LINC01499(API5)* | rs420798 | C | 0.795 | 0.913 | 0.906 | 0.926 | 0.929 | 0.907 | 0.874 | 0.912 | 0.940 |
| *API5* | rs16937370 | G | 0.039 | 0 | 0 | 0 | 0 | 0 | 0 | 0 | 0 |
| *SLC43A3* | rs549630 | G | 0.364 | 0.579 | 0.573 | 0.516 | 0.611 | 0.500 | 0.667 | 0.624 | 0.560 |
| *RIMBP2* | rs2895135 | A | 0.174 | 0.039 | 0.052 | 0.115 | 0.010 | 0.058 | 0.015 | 0.018 | 0.028 |
| *GALNTL1* | rs12589991 | A | 0.109 | 0.089 | 0.109 | 0.115 | 0.056 | 0.049 | 0.121 | 0.065 | 0.120 |
| *SMG6* | rs749240 | T | 0.426 | 0.691 | 0.641 | 0.607 | 0.697 | 0.699 | 0.667 | 0.729 | 0.764 |
| *LILRB1* | rs798887 | A | 0.695 | 0.825 | 0.891 | 0.803 | 0.788 | 0.823 | 0.798 | 0.835 | 0.833 |
| *LILRA1* | rs103294 | C | 0.782 | 0.926 | 0.938 | 0.885 | 0.975 | 0.867 | 0.934 | 0.918 | 0.954 |
| *RALGAPA2* | rs6082210 | A | 0.100 | 0.165 | 0.125 | 0.156 | 0.182 | 0.181 | 0.177 | 0.106 | 0.208 |
| *CDH26* | rs817277 | A | 0.447 | 0.692 | 0.661 | 0.623 | 0.722 | 0.743 | 0.707 | 0.753 | 0.616 |
| *CDH26* | rs817283 | A | 0.441 | 0.669 | 0.620 | 0.590 | 0.687 | 0.726 | 0.707 | 0.729 | 0.597 |
| *UBD* | rs6933331 | A | 0.079 | 0.123 | 0.099 | 0.082 | 0.121 | 0.150 | 0.141 | 0.129 | 0.120 |
| *UBD* | rs3025657 | G | 0.079 | 0.123 | 0.099 | 0.074 | 0.116 | 0.146 | 0.152 | 0.147 | 0.111 |
| *GABBR1* | rs29273 | G | 0.863 | 0.864 | 0.870 | 0.844 | 0.859 | 0.854 | 0.894 | 0.871 | 0.852 |
| *MOG* | rs3129045 | T | 0.386 | 0.580 | 0.630 | 0.525 | 0.525 | 0.558 | 0.556 | 0.606 | 0.644 |
| *HLA-F* | rs3116788 | G | 0.310 | 0.312 | 0.292 | 0.270 | 0.338 | 0.323 | 0.359 | 0.329 | 0.259 |
| *HLA-F* | rs1610584 | T | 0.310 | 0.312 | 0.292 | 0.270 | 0.338 | 0.323 | 0.359 | 0.329 | 0.259 |
| *HLA-F* | rs1610585 | C | 0.310 | 0.312 | 0.292 | 0.270 | 0.338 | 0.323 | 0.359 | 0.329 | 0.259 |
| *HLA-F* | rs1610593 | T | 0.311 | 0.312 | 0.292 | 0.270 | 0.338 | 0.323 | 0.359 | 0.329 | 0.264 |
| *HLA-F* | rs1611356 | G | 0.689 | 0.688 | 0.708 | 0.730 | 0.662 | 0.677 | 0.641 | 0.671 | 0.736 |
| *HLA-F* | rs1611381 | T | 0.311 | 0.312 | 0.292 | 0.270 | 0.338 | 0.323 | 0.359 | 0.329 | 0.264 |
| *HLA-F* | rs7741807 | G | 0.939 | 0.934 | 0.958 | 0.934 | 0.955 | 0.867 | 0.960 | 0.947 | 0.931 |
| *HLA-F* | rs1611388 | C | 0.310 | 0.312 | 0.292 | 0.270 | 0.338 | 0.323 | 0.354 | 0.329 | 0.264 |
| *HLA-F* | rs1627465 | C | 0.311 | 0.312 | 0.292 | 0.270 | 0.338 | 0.323 | 0.359 | 0.329 | 0.264 |
| *LOC285830 ( HLA-F antisense RNA1)* | rs9258205 | C | 0.217 | 0.223 | 0.229 | 0.164 | 0.268 | 0.204 | 0.247 | 0.212 | 0.218 |
| *LOC285830 ( HLA-F antisense RNA1)* | rs2523386 | A | 0.088 | 0.044 | 0.026 | 0.082 | 0.035 | 0.080 | 0.051 | 0.012 | 0.028 |
| *LOC285830 ( HLA-F antisense RNA1)* | rs2844845 | A | 0.094 | 0.063 | 0.052 | 0.082 | 0.056 | 0.097 | 0.106 | 0.012 | 0.032 |
| *LOC285830 ( HLA-F antisense RNA1)* | rs1633041 | T | 0.232 | 0.251 | 0.240 | 0.320 | 0.227 | 0.265 | 0.278 | 0.194 | 0.250 |
| *LOC285830 ( HLA-F antisense RNA1)* | rs1737031 | A | 0.329 | 0.486 | 0.505 | 0.500 | 0.480 | 0.518 | 0.404 | 0.412 | 0.565 |
| *LOC285830 ( HLA-F antisense RNA1)* | rs885940 | A | 0.232 | 0.250 | 0.240 | 0.320 | 0.222 | 0.265 | 0.278 | 0.194 | 0.250 |
| *LOC285830 ( HLA-F antisense RNA1)* | rs1610637 | C | 0.232 | 0.250 | 0.240 | 0.320 | 0.222 | 0.265 | 0.278 | 0.194 | 0.250 |
| *LOC285830 ( HLA-F antisense RNA1)* | rs1615251 | T | 0.616 | 0.402 | 0.370 | 0.393 | 0.439 | 0.327 | 0.510 | 0.429 | 0.356 |
| *HLA-G* | rs1633002 | A | 0.771 | 0.757 | 0.781 | 0.689 | 0.778 | 0.735 | 0.742 | 0.806 | 0.755 |
| *HLA-G* | rs1632973 | A | 0.232 | 0.250 | 0.240 | 0.320 | 0.222 | 0.261 | 0.278 | 0.194 | 0.250 |
| *HLA-G* | rs1736963 | T | 0.232 | 0.250 | 0.240 | 0.320 | 0.222 | 0.265 | 0.278 | 0.194 | 0.250 |
| *HLA-G* | rs2523408 | G | 0.001 | 0 | 0 | 0 | 0 | 0 | 0 | 0 | 0 |
| *HLA-G* | rs1611172 | G | 0.232 | 0.250 | 0.240 | 0.320 | 0.222 | 0.265 | 0.278 | 0.194 | 0.250 |
| *HLA-G* | rs753544 | T | 0.232 | 0.250 | 0.240 | 0.320 | 0.222 | 0.265 | 0.278 | 0.194 | 0.250 |
| *HLA-G* | rs1077433 | A | 0.232 | 0.250 | 0.240 | 0.320 | 0.222 | 0.265 | 0.278 | 0.194 | 0.250 |
| *HLA-G* | rs1736951 | A | 0.302 | 0.374 | 0.375 | 0.418 | 0.318 | 0.438 | 0.389 | 0.353 | 0.333 |
| *HLA-G* | rs407238 | C | 0.260 | 0.241 | 0.260 | 0.213 | 0.222 | 0.212 | 0.278 | 0.206 | 0.278 |
| *HCG9* | rs9260954 | G | 0.041 | 0.050 | 0.052 | 0.041 | 0.051 | 0.080 | 0.061 | 0.006 | 0.046 |
| *HCG9* | rs6911737 | A | 0.192 | 0.300 | 0.286 | 0.270 | 0.253 | 0.345 | 0.288 | 0.324 | 0.315 |
| *HCG9* | rs6926792 | A | 0.191 | 0.299 | 0.286 | 0.270 | 0.253 | 0.345 | 0.278 | 0.324 | 0.319 |
| *HCG9* | rs6931776 | G | 0.191 | 0.299 | 0.286 | 0.270 | 0.253 | 0.345 | 0.278 | 0.324 | 0.319 |
| *ZNRD1* | rs9261189 | T | 0.192 | 0.300 | 0.286 | 0.270 | 0.253 | 0.350 | 0.278 | 0.324 | 0.319 |
| *ZNRD1* | rs3869068 | A | 0.191 | 0.299 | 0.286 | 0.270 | 0.253 | 0.345 | 0.278 | 0.324 | 0.319 |
| *ZNRD1* | rs9261265 | C | 0.041 | 0.048 | 0.052 | 0.041 | 0.051 | 0.071 | 0.061 | 0.006 | 0.046 |
| *PPP1R11* | rs2074482 | T | 0.191 | 0.299 | 0.286 | 0.270 | 0.253 | 0.345 | 0.278 | 0.324 | 0.319 |
| *RNF39* | rs9261317 | A | 0.958 | 0.950 | 0.948 | 0.959 | 0.949 | 0.920 | 0.939 | 0.994 | 0.954 |
| *TRIM31* | rs9261376 | G | 0.317 | 0.507 | 0.490 | 0.434 | 0.465 | 0.580 | 0.449 | 0.600 | 0.505 |
| *TRIM31* | rs9261389 | G | 0.315 | 0.503 | 0.490 | 0.434 | 0.460 | 0.580 | 0.449 | 0.600 | 0.486 |
| *TRIM31* | rs6923832 | A | 0.041 | 0.050 | 0.052 | 0.041 | 0.051 | 0.080 | 0.061 | 0.006 | 0.046 |
| *MUC21* | rs2530710 | A | 0.139 | 0.021 | 0.026 | 0.057 | 0.010 | 0.009 | 0.030 | 0.035 | 0 |
| *MUC21* | rs2517446 | C | 0.154 | 0.154 | 0.172 | 0.156 | 0.116 | 0.146 | 0.111 | 0.229 | 0.157 |
| *MUC21* | rs2517411 | G | 0.155 | 0.154 | 0.172 | 0.156 | 0.116 | 0.146 | 0.111 | 0.229 | 0.157 |
| *MUC21* | rs2844673 | A | 0.173 | 0.154 | 0.172 | 0.156 | 0.116 | 0.146 | 0.111 | 0.229 | 0.157 |
| *MUC21* | rs2252925 | G | 0.155 | 0.154 | 0.172 | 0.156 | 0.116 | 0.146 | 0.111 | 0.229 | 0.157 |
| *MUC21* | rs2252926 | G | 0.155 | 0.154 | 0.172 | 0.156 | 0.116 | 0.146 | 0.111 | 0.229 | 0.157 |
| *MUC21* | rs1634717 | T | 0.305 | 0.273 | 0.286 | 0.246 | 0.253 | 0.279 | 0.268 | 0.324 | 0.255 |
| *MUC21* | rs2523915 | T | 0.845 | 0.846 | 0.828 | 0.844 | 0.884 | 0.854 | 0.889 | 0.771 | 0.843 |
| *MUC21* | rs1632854 | T | 0.695 | 0.727 | 0.714 | 0.754 | 0.747 | 0.721 | 0.732 | 0.676 | 0.745 |
| *C6orf15* | rs1265048 | A | 0.606 | 0.705 | 0.661 | 0.689 | 0.732 | 0.642 | 0.793 | 0.735 | 0.690 |
| *PSORS1C1* | rs4959053 | A | 0.093 | 0.012 | 0.031 | 0.041 | 0.005 | 0 | 0 | 0.012 | 0.009 |
| *CCHCR1* | rs2240063 | A | 0.427 | 0.438 | 0.469 | 0.475 | 0.500 | 0.420 | 0.455 | 0.347 | 0.407 |
| *CCHCR1* | rs2073716 | C | 0.895 | 0.849 | 0.854 | 0.852 | 0.884 | 0.854 | 0.879 | 0.794 | 0.819 |
| *TCF19* | rs2073723 | T | 0.231 | 0.131 | 0.120 | 0.189 | 0.106 | 0.155 | 0.101 | 0.129 | 0.134 |
| *POU5F1* | rs9501063 | G | 0.891 | 0.811 | 0.844 | 0.811 | 0.813 | 0.810 | 0.838 | 0.771 | 0.787 |
| *POU5F1* | rs9263804 | C | 0.237 | 0.130 | 0.120 | 0.189 | 0.101 | 0.155 | 0.101 | 0.129 | 0.134 |
| *POU5F1* | rs3130501 | A | 0.231 | 0.130 | 0.120 | 0.189 | 0.101 | 0.155 | 0.101 | 0.129 | 0.134 |
| *POU5F1* | rs3132524 | A | 0.237 | 0.130 | 0.120 | 0.189 | 0.101 | 0.155 | 0.101 | 0.129 | 0.134 |
| *HCG27* | rs3130944 | C | 0.779 | 0.862 | 0.901 | 0.811 | 0.879 | 0.836 | 0.778 | 0.912 | 0.907 |
| *HLA-C* | rs3905495 | C | 0.555 | 0.508 | 0.432 | 0.615 | 0.500 | 0.544 | 0.586 | 0.441 | 0.468 |
| *DHFRP2* | rs7761068 | T | 0.358 | 0.402 | 0.401 | 0.451 | 0.348 | 0.491 | 0.399 | 0.412 | 0.329 |
| *HLA-B* | rs9266406 | A | 0.296 | 0.238 | 0.255 | 0.148 | 0.182 | 0.248 | 0.207 | 0.259 | 0.329 |
| *HLA-B* | rs9266409 | C | 0.297 | 0.240 | 0.266 | 0.148 | 0.182 | 0.248 | 0.207 | 0.259 | 0.329 |
| *HLA-B* | rs6910516 | C | 0.297 | 0.240 | 0.266 | 0.148 | 0.182 | 0.248 | 0.207 | 0.259 | 0.329 |
| *MICA* | rs2523467 | A | 0.436 | 0.554 | 0.536 | 0.566 | 0.667 | 0.447 | 0.606 | 0.565 | 0.514 |
| *MICA* | rs3094584 | T | 0.204 | 0.253 | 0.276 | 0.230 | 0.177 | 0.283 | 0.162 | 0.265 | 0.361 |
| *BAG6(BAT3)* | rs2077102 | T | 0.127 | 0.076 | 0.057 | 0.131 | 0.040 | 0.159 | 0.045 | 0.076 | 0.032 |
| *C6orf47* | rs2242655 | C | 0.873 | 0.924 | 0.943 | 0.869 | 0.960 | 0.841 | 0.955 | 0.924 | 0.968 |
| *SLC44A4* | rs11965547 | A | 0.127 | 0.077 | 0.052 | 0.123 | 0.040 | 0.133 | 0.040 | 0.082 | 0.079 |
| *C6orf10* | rs544358 | C | 0.327 | 0.135 | 0.146 | 0.164 | 0.081 | 0.235 | 0.111 | 0.135 | 0.079 |
| *C6orf10* | rs574710 | G | 0.337 | 0.135 | 0.146 | 0.164 | 0.081 | 0.235 | 0.111 | 0.135 | 0.079 |
| *C6orf10* | rs539703 | C | 0.327 | 0.135 | 0.146 | 0.164 | 0.081 | 0.235 | 0.111 | 0.135 | 0.079 |
| *C6orf10* | rs926591 | T | 0.326 | 0.135 | 0.151 | 0.164 | 0.086 | 0.235 | 0.101 | 0.135 | 0.079 |
| *C6orf10* | rs4959093 | C | 0.327 | 0.135 | 0.151 | 0.164 | 0.086 | 0.235 | 0.101 | 0.135 | 0.079 |
| *BTNL2* | rs2076530 | G | 0.387 | 0.328 | 0.312 | 0.311 | 0.182 | 0.522 | 0.192 | 0.459 | 0.301 |
| *HLA-DQA1* | rs9272346 | G | 0.475 | 0.533 | 0.630 | 0.598 | 0.530 | 0.394 | 0.682 | 0.329 | 0.579 |
| *HLA-DQB1* | rs6457617 | C | 0.465 | 0.459 | 0.568 | 0.459 | 0.586 | 0.274 | 0.449 | 0.329 | 0.551 |
| *COL12A1* | rs4640857 | G | 0.318 | 0.056 | 0.099 | 0.156 | 0.035 | 0.018 | 0.061 | 0.012 | 0.051 |
| *C10orf11* | rs1323076 | G | 0.365 | 0.348 | 0.417 | 0.393 | 0.318 | 0.319 | 0.409 | 0.288 | 0.310 |
| *C10orf11* | rs17434565 | G | 0.167 | 0.023 | 0.057 | 0.057 | 0.015 | 0.004 | 0.025 | 0.012 | 0.005 |
| *PAX8* | rs11123169 | C | 0.322 | 0.378 | 0.422 | 0.328 | 0.303 | 0.367 | 0.414 | 0.459 | 0.352 |
| *PAX8* | rs10864912 | T | 0.384 | 0.433 | 0.484 | 0.434 | 0.485 | 0.358 | 0.409 | 0.365 | 0.495 |
| *HIVEP3* | rs4660590 | A | 0.536 | 0.716 | 0.677 | 0.672 | 0.717 | 0.765 | 0.692 | 0.729 | 0.731 |
| *CEP135* | rs2593082 | T | 0.542 | 0.686 | 0.693 | 0.623 | 0.687 | 0.655 | 0.687 | 0.718 | 0.722 |
| *CEP135* | rs2611826 | G | 0.460 | 0.357 | 0.354 | 0.410 | 0.348 | 0.394 | 0.374 | 0.329 | 0.306 |
| *HMP19* | rs1909704 | A | 0.538 | 0.509 | 0.526 | 0.557 | 0.475 | 0.473 | 0.535 | 0.512 | 0.509 |
| *TTLL7* | rs11163772 | A | 0.220 | 0.354 | 0.349 | 0.328 | 0.359 | 0.288 | 0.379 | 0.400 | 0.380 |
| *TENM4(ODZ4)* | rs2156215 | T | 0.233 | 0.248 | 0.240 | 0.238 | 0.308 | 0.195 | 0.258 | 0.235 | 0.264 |
| *KLRK1* | rs2617151 | A | 0.176 | 0.231 | 0.229 | 0.221 | 0.192 | 0.336 | 0.172 | 0.294 | 0.167 |
| *KLRK1* | rs2733852 | G | 0.315 | 0.540 | 0.531 | 0.475 | 0.500 | 0.628 | 0.601 | 0.588 | 0.435 |
| *OSR1* | rs4666492 | G | 0.312 | 0.248 | 0.281 | 0.303 | 0.182 | 0.279 | 0.268 | 0.212 | 0.227 |
| *CTNNA2* | rs4852547 | G | 0.384 | 0.492 | 0.531 | 0.385 | 0.500 | 0.460 | 0.475 | 0.465 | 0.579 |
| *MN1* | rs134006 | C | 0.213 | 0.315 | 0.255 | 0.254 | 0.409 | 0.292 | 0.313 | 0.365 | 0.301 |
| *LTN1(RNF160)* | rs2832137 | T | 0.340 | 0.111 | 0.135 | 0.164 | 0.076 | 0.102 | 0.136 | 0.100 | 0.088 |
| *HERPUD2* | rs11763983 | T | 0.294 | 0.086 | 0.146 | 0.131 | 0.045 | 0.084 | 0.096 | 0.065 | 0.056 |
| *GALNT10* | rs574750 | A | 0.313 | 0.460 | 0.396 | 0.451 | 0.540 | 0.456 | 0.470 | 0.476 | 0.431 |
| *SAMD3(TMEM200A)* | rs9483115 | T | 0.544 | 0.878 | 0.849 | 0.738 | 0.889 | 0.881 | 0.924 | 0.912 | 0.903 |
| *SAMD3(TMEM200A)* | rs4141940 | A | 0.518 | 0.790 | 0.781 | 0.680 | 0.788 | 0.783 | 0.818 | 0.829 | 0.815 |
| *SAMD3(TMEM200A)* | rs899276 | A | 0.514 | 0.772 | 0.776 | 0.672 | 0.753 | 0.779 | 0.808 | 0.829 | 0.759 |
| *SAMD3(TMEM200A)* | rs7758496 | G | 0.557 | 0.937 | 0.880 | 0.795 | 0.955 | 0.978 | 0.960 | 0.976 | 0.958 |
| *SAMD3(TMEM200A)* | rs724324 | G | 0.544 | 0.877 | 0.854 | 0.738 | 0.909 | 0.903 | 0.879 | 0.906 | 0.894 |
| *SAMD3* | rs4897380 | C | 0.567 | 0.937 | 0.865 | 0.803 | 0.960 | 0.978 | 0.965 | 0.971 | 0.963 |
| *SEMA6D* | rs470151 | T | 0.197 | 0.257 | 0.224 | 0.295 | 0.303 | 0.235 | 0.247 | 0.247 | 0.264 |
| *PMFBP1* | rs11862324 | T | 0.381 | 0.542 | 0.458 | 0.492 | 0.561 | 0.504 | 0.662 | 0.500 | 0.588 |
| *NAV2* | rs2707110 | C | 0.433 | 0.571 | 0.615 | 0.557 | 0.571 | 0.588 | 0.571 | 0.576 | 0.519 |
| *NAV2* | rs873764 | G | 0.518 | 0.615 | 0.651 | 0.648 | 0.606 | 0.615 | 0.591 | 0.582 | 0.620 |
| *TMEM132B* | rs4435061 | A | 0.444 | 0.689 | 0.688 | 0.541 | 0.727 | 0.735 | 0.672 | 0.682 | 0.713 |
| *TMEM132B* | rs10846917 | T | 0.472 | 0.269 | 0.286 | 0.336 | 0.202 | 0.239 | 0.268 | 0.306 | 0.282 |
| *TMEM132B* | rs10846924 | T | 0.330 | 0.355 | 0.396 | 0.328 | 0.364 | 0.358 | 0.338 | 0.335 | 0.352 |
| *STX8* | rs1549332 | A | 0.135 | 0.216 | 0.229 | 0.172 | 0.207 | 0.204 | 0.308 | 0.206 | 0.171 |
| *OVCH1* | rs1436321 | A | 0.489 | 0.747 | 0.677 | 0.648 | 0.813 | 0.810 | 0.763 | 0.782 | 0.699 |
| *SLC41A2* | rs2731031 | A | 0.373 | 0.238 | 0.203 | 0.287 | 0.192 | 0.257 | 0.273 | 0.253 | 0.218 |
| *HNF4G* | rs2980221 | A | 0.479 | 0.483 | 0.469 | 0.410 | 0.520 | 0.558 | 0.495 | 0.441 | 0.449 |
| *SMARCA2* | rs7033529 | A | 0.570 | 0.152 | 0.193 | 0.262 | 0.126 | 0.133 | 0.141 | 0.141 | 0.116 |
| *EBF2* | rs4570167 | C | 0.493 | 0.825 | 0.828 | 0.656 | 0.838 | 0.876 | 0.864 | 0.794 | 0.838 |
| *EBF2* | rs4242425 | T | 0.495 | 0.832 | 0.839 | 0.656 | 0.864 | 0.885 | 0.864 | 0.794 | 0.843 |
| *GAS2* | rs10833804 | G | 0.603 | 0.598 | 0.615 | 0.631 | 0.601 | 0.650 | 0.449 | 0.612 | 0.634 |
| *LYST/NID1* | rs7354999 | G | 0.738 | 0.762 | 0.760 | 0.803 | 0.798 | 0.721 | 0.823 | 0.682 | 0.755 |
| *LOC100132252* | rs9469615 | C | 0.097 | 0.129 | 0.083 | 0.115 | 0.136 | 0.195 | 0.146 | 0.129 | 0.083 |
| *LOC107984355* | rs872837 | A | 0.293 | 0.275 | 0.297 | 0.246 | 0.308 | 0.221 | 0.263 | 0.288 | 0.301 |
| *SACM1L* | rs1969624 | C | 0.403 | 0.421 | 0.411 | 0.426 | 0.414 | 0.540 | 0.303 | 0.447 | 0.394 |
| *PLEKHB1* | rs591804 | G | 0.383 | 0.489 | 0.438 | 0.492 | 0.535 | 0.429 | 0.525 | 0.524 | 0.495 |
| *ATP8A1* | rs2100766 | T | 0.145 | 0.294 | 0.302 | 0.230 | 0.354 | 0.243 | 0.278 | 0.294 | 0.338 |
| *KCNK9* | rs1961261 | A | 0.209 | 0.129 | 0.078 | 0.164 | 0.126 | 0.119 | 0.121 | 0.200 | 0.116 |
| *SUMO4* | rs237024 | C | 0.702 | 0.970 | 0.948 | 0.926 | 0.990 | 0.973 | 0.975 | 0.982 | 0.981 |

1. 1000 Genomes Ad-mixed American and East Asian populations population samples

| **Gene** | **Variant/SNP** | **Behcet Allele** | **AMR** | CLM | MXL | PEL | PUR | **EAS** | CDX | CHB | CHS | JPT | KHV |
| --- | --- | --- | --- | --- | --- | --- | --- | --- | --- | --- | --- | --- | --- |
| *IL-10* | rs1518111 | A | 0.329 | 0.287 | 0.422 | 0.371 | 0.274 | 0.676 | 0.651 | 0.743 | 0.686 | 0.644 | 0.652 |
| *IL-10* | rs1800871 | T | 0.333 | 0.298 | 0.422 | 0.371 | 0.279 | 0.676 | 0.651 | 0.743 | 0.686 | 0.639 | 0.657 |
| *IL-10* | rs1800872 | A | 0.333 | 0.298 | 0.422 | 0.371 | 0.279 | 0.676 | 0.651 | 0.743 | 0.686 | 0.639 | 0.657 |
| *IL-10* | rs1554286 | C | 0.693 | 0.729 | 0.594 | 0.647 | 0.760 | 0.339 | 0.360 | 0.267 | 0.338 | 0.365 | 0.369 |
| *IL23R,IL12RB2* | rs1495965 | G | 0.354 | 0.378 | 0.289 | 0.371 | 0.361 | 0.517 | 0.565 | 0.510 | 0.529 | 0.433 | 0.556 |
| *IL23R,IL12RB2* | rs924080 | T | 0.581 | 0.521 | 0.523 | 0.765 | 0.519 | 0.759 | 0.769 | 0.791 | 0.800 | 0.707 | 0.727 |
| *IL23R,IL12RB2* | rs12119179 | A | 0.769 | 0.718 | 0.773 | 0.806 | 0.784 | 0.494 | 0.430 | 0.505 | 0.495 | 0.587 | 0.444 |
| *IL23R,IL12RB2* | rs11209033 | C | 0.769 | 0.718 | 0.773 | 0.806 | 0.784 | 0.499 | 0.435 | 0.515 | 0.500 | 0.591 | 0.444 |
| *IL23R,IL12RB2* | rs12141431 | C | 0.215 | 0.277 | 0.195 | 0.176 | 0.202 | 0.506 | 0.575 | 0.490 | 0.505 | 0.413 | 0.556 |
| *TNFAIP3* | rs9494885 | T | 0.863 | 0.846 | 0.914 | 0.900 | 0.817 | 0.938 | 0.973 | 0.922 | 0.952 | 0.875 | 0.975 |
| *TNFAIP3* | rs10499194 | C | 0.722 | 0.750 | 0.688 | 0.806 | 0.649 | 0.967 | 0.973 | 0.981 | 0.976 | 0.933 | 0.975 |
| *TNFAIP3* | rs610604 | A | 0.605 | 0.580 | 0.617 | 0.629 | 0.601 | 0.900 | 0.935 | 0.883 | 0.890 | 0.947 | 0.843 |
| *TNFAIP3* | rs7753873 | C | 0.131 | 0.144 | 0.086 | 0.100 | 0.173 | 0.066 | 0.032 | 0.078 | 0.057 | 0.135 | 0.025 |
| *STAT4* | rs7574070 | A | 0.341 | 0.319 | 0.336 | 0.300 | 0.399 | 0.612 | 0.737 | 0.612 | 0.605 | 0.500 | 0.621 |
| *STAT4* | rs897200 | A | 0.344 | 0.330 | 0.328 | 0.300 | 0.404 | 0.614 | 0.737 | 0.617 | 0.605 | 0.500 | 0.626 |
| *STAT4* | rs7572482 | A | 0.334 | 0.319 | 0.320 | 0.282 | 0.399 | 0.614 | 0.737 | 0.617 | 0.605 | 0.500 | 0.626 |
| *CCR1* | rs17282391 | G | 0.049 | 0.064 | 0.031 | 0.053 | 0.043 | 0.044 | 0.027 | 0.068 | 0.043 | 0.048 | 0.030 |
| *CCR1* | rs10510749 | T | 0.059 | 0.069 | 0.039 | 0.065 | 0.058 | 0.043 | 0.027 | 0.068 | 0.043 | 0.043 | 0.030 |
| *CCR1* | rs13084057 | G | 0.059 | 0.069 | 0.039 | 0.065 | 0.058 | 0.043 | 0.027 | 0.068 | 0.043 | 0.043 | 0.030 |
| *CCR1* | rs7631551 | A | 0.095 | 0.085 | 0.055 | 0.076 | 0.144 | 0.043 | 0.027 | 0.068 | 0.043 | 0.043 | 0.030 |
| *CCR1* | rs7616215 | T | 0.736 | 0.718 | 0.789 | 0.818 | 0.654 | 0.873 | 0.914 | 0.850 | 0.876 | 0.822 | 0.909 |
| *CCR3* | rs7649764 | C | 0.666 | 0.676 | 0.711 | 0.571 | 0.707 | 0.403 | 0.317 | 0.369 | 0.424 | 0.486 | 0.409 |
| *CCR3* | rs9990343 | G | 0.378 | 0.372 | 0.391 | 0.318 | 0.423 | 0.122 | 0.075 | 0.141 | 0.110 | 0.202 | 0.076 |
| *CCR3* | rs6803980 | A | 0.382 | 0.378 | 0.398 | 0.318 | 0.428 | 0.122 | 0.075 | 0.141 | 0.110 | 0.202 | 0.076 |
| *CCR3* | rs13075270 | C | 0.091 | 0.101 | 0.062 | 0.059 | 0.125 | 0.038 | 0.016 | 0.083 | 0.033 | 0.029 | 0.025 |
| *CCR3* | rs13092160 | C | 0.058 | 0.074 | 0.031 | 0.059 | 0.058 | 0.038 | 0.016 | 0.083 | 0.033 | 0.029 | 0.025 |
| *CCR3* | rs2373156 | T | 0.121 | 0.133 | 0.086 | 0.059 | 0.183 | 0.038 | 0.016 | 0.083 | 0.033 | 0.029 | 0.025 |
| *CCR3* | rs7651539 | T | 0.121 | 0.133 | 0.086 | 0.059 | 0.183 | 0.038 | 0.016 | 0.083 | 0.033 | 0.029 | 0.025 |
| *CCR3* | rs1542755 | A | 0.048 | 0.064 | 0.039 | 0.041 | 0.043 | 0.038 | 0.016 | 0.083 | 0.033 | 0.029 | 0.025 |
| *CCR3* | rs13067058 | A | 0.046 | 0.059 | 0.039 | 0.041 | 0.043 | 0.036 | 0.011 | 0.083 | 0.033 | 0.029 | 0.020 |
| *CCR3* | rs13092160 | C | 0.058 | 0.074 | 0.031 | 0.059 | 0.058 | 0.038 | 0.016 | 0.083 | 0.033 | 0.029 | 0.025 |
| *KLRC4* | rs2617170 | C | 0.667 | 0.707 | 0.727 | 0.682 | 0.582 | 0.550 | 0.500 | 0.549 | 0.610 | 0.601 | 0.480 |
| *MEFV* | rs61752717 | G | 0.001 | 0 | 0 | 0.006 | 0 | 0 | 0 | 0 | 0 | 0 | 0 |
| *ERAP1* | rs17482078 | T | 0.124 | 0.170 | 0.070 | 0.053 | 0.173 | 0.058 | 0.054 | 0.063 | 0.057 | 0.072 | 0.040 |
| *FUT2* | rs681343 | T | 0.343 | 0.388 | 0.320 | 0.124 | 0.495 | 0.004 | 0 | 0.015 | 0 | 0 | 0.005 |
| *IL12A* | rs17810546 | A | 0.899 | 0.899 | 0.875 | 0.929 | 0.889 | 1.000 | 1.000 | 1.000 | 1.000 | 1.000 | 1.000 |
| *IL23R* | rs11209026 | A | 0.052 | 0.053 | 0.062 | 0.012 | 0.077 | 0 | 0 | 0 | 0 | 0 | 0 |
| *IL23R* | rs76418789 | A | 0 | 0 | 0 | 0 | 0 | 0.0526 | 0.0376 | 0.0631 | 0.0429 | 0.10 | 0.0152 |
| *IL23R* | rs17375018 | G | 0.504 | 0.569 | 0.516 | 0.265 | 0.635 | 0.680 | 0.715 | 0.689 | 0.695 | 0.644 | 0.657 |
| *IL23R* | rs11209032 | A | 0.229 | 0.282 | 0.227 | 0.188 | 0.216 | 0.497 | 0.570 | 0.471 | 0.495 | 0.409 | 0.551 |
| *IL23R* | rs1343151 | T | 0.277 | 0.324 | 0.234 | 0.082 | 0.418 | 0.053 | 0.038 | 0.024 | 0.038 | 0.101 | 0.061 |
| *TLR4* | rs4986790 | G | 0.037 | 0.053 | 0.031 | 0.006 | 0.053 | 0 | 0 | 0 | 0 | 0 | 0 |
| *TLR4* | rs4986791 | T | 0.036 | 0.064 | 0.031 | 0 | 0.043 | 0 | 0 | 0 | 0 | 0 | 0 |
| *NOD2* | rs2066844 | T | 0.024 | 0.053 | 0 | 0.006 | 0.029 | 0 | 0 | 0 | 0 | 0 | 0 |
| *NOD2* | rs2066845 | C | 0.013 | 0.021 | 0.023 | 0 | 0.010 | 0 | 0 | 0 | 0 | 0 | 0 |
| *NOD2* | rs2066847 | ins-C | 0.016 | 0.016 | 0.008 | 0.006 | 0.029 | 0 | 0 | 0 | 0 | 0 | 0 |
| *IL1* | rs1800587 | C | 0.725 | 0.691 | 0.766 | 0.724 | 0.731 | 0.928 | 0.930 | 0.937 | 0.948 | 0.865 | 0.960 |
| *IL1* | rs1143634 | T | 0.125 | 0.170 | 0.086 | 0.053 | 0.168 | 0.023 | 0.011 | 0.019 | 0.010 | 0.062 | 0.010 |
| *IL1* | rs16944 | G | 0.450 | 0.548 | 0.469 | 0.300 | 0.471 | 0.531 | 0.500 | 0.549 | 0.543 | 0.534 | 0.525 |
| *TNFα* | rs1799964 | C | 0.219 | 0.181 | 0.258 | 0.182 | 0.260 | 0.195 | 0.210 | 0.218 | 0.190 | 0.135 | 0.227 |
| *TNFα* | rs361525 | A | 0.082 | 0.059 | 0.102 | 0.118 | 0.062 | 0.031 | 0.011 | 0.034 | 0.038 | 0.014 | 0.056 |
| *TNFα* | rs1799724 | T | 0.183 | 0.170 | 0.203 | 0.282 | 0.101 | 0.125 | 0.129 | 0.141 | 0.071 | 0.163 | 0.121 |
| *IL12* | rs3212227 | A | 0.659 | 0.755 | 0.602 | 0.541 | 0.702 | 0.499 | 0.457 | 0.568 | 0.486 | 0.466 | 0.515 |
| *IL18* | rs1946518 | C | 0.509 | 0.511 | 0.508 | 0.471 | 0.538 | 0.473 | 0.489 | 0.408 | 0.514 | 0.394 | 0.566 |
| *IL17F-A126G* | rs2397084 | T | 0.952 | 0.931 | 0.945 | 0.994 | 0.942 | 0.996 | 0.995 | 0.995 | 0.995 | 1.000 | 0.995 |
| *LOC100129342* | rs11206377 | G | 0.633 | 0.670 | 0.633 | 0.753 | 0.500 | 0.568 | 0.575 | 0.500 | 0.519 | 0.688 | 0.561 |
| *CCDC180* | rs2061634 | G | 0.304 | 0.314 | 0.266 | 0.394 | 0.245 | 0.184 | 0.194 | 0.131 | 0.248 | 0.154 | 0.192 |
| *CPVL* | rs317711 | C | 0.147 | 0.176 | 0.156 | 0.059 | 0.188 | 0.152 | 0.134 | 0.170 | 0.138 | 0.082 | 0.237 |
| *UBASH3B* | rs4936742 | T | 0.418 | 0.452 | 0.438 | 0.394 | 0.394 | 0.569 | 0.618 | 0.539 | 0.629 | 0.428 | 0.641 |
| *UBAC2* | rs9513584 | G | 0.504 | 0.346 | 0.547 | 0.700 | 0.462 | 0.494 | 0.602 | 0.413 | 0.448 | 0.538 | 0.480 |
| *UBAC2* | rs9517644 | T | 0.499 | 0.340 | 0.539 | 0.694 | 0.457 | 0.494 | 0.602 | 0.413 | 0.448 | 0.538 | 0.480 |
| *UBAC2* | rs11069357 | A | 0.499 | 0.340 | 0.539 | 0.694 | 0.457 | 0.492 | 0.602 | 0.408 | 0.448 | 0.538 | 0.475 |
| *UBAC2* | rs984477 | G | 0.526 | 0.372 | 0.570 | 0.700 | 0.495 | 0.494 | 0.602 | 0.413 | 0.448 | 0.538 | 0.480 |
| *UBAC2* | rs9554573 | A | 0.509 | 0.351 | 0.547 | 0.706 | 0.466 | 0.507 | 0.618 | 0.417 | 0.462 | 0.548 | 0.500 |
| *UBAC2* | rs6491493 | G | 0.504 | 0.346 | 0.547 | 0.700 | 0.462 | 0.493 | 0.597 | 0.413 | 0.448 | 0.538 | 0.480 |
| *UBAC2* | rs9517668 | T | 0.287 | 0.170 | 0.398 | 0.441 | 0.197 | 0.317 | 0.398 | 0.267 | 0.267 | 0.356 | 0.308 |
| *UBAC2* | rs7999348 | G | 0.507 | 0.351 | 0.539 | 0.712 | 0.462 | 0.495 | 0.597 | 0.413 | 0.448 | 0.538 | 0.490 |
| *UBAC2* | rs9554581 | T | 0.267 | 0.149 | 0.383 | 0.418 | 0.178 | 0.320 | 0.403 | 0.272 | 0.267 | 0.356 | 0.313 |
| *UBAC2* | rs17575643 | T | 0.219 | 0.144 | 0.234 | 0.376 | 0.149 | 0.057 | 0.038 | 0.102 | 0.071 | 0.034 | 0.035 |
| *UBAC2* | rs727263 | A | 0.267 | 0.154 | 0.375 | 0.418 | 0.178 | 0.318 | 0.403 | 0.277 | 0.262 | 0.341 | 0.318 |
| *UBAC2* | rs7332161 | A | 0.268 | 0.154 | 0.375 | 0.424 | 0.178 | 0.319 | 0.403 | 0.277 | 0.262 | 0.346 | 0.318 |
| *UBAC2* | rs912130 | C | 0.504 | 0.346 | 0.555 | 0.694 | 0.462 | 0.495 | 0.602 | 0.422 | 0.443 | 0.534 | 0.485 |
| *UBAC2* | rs2892976 | G | 0.346 | 0.229 | 0.414 | 0.488 | 0.293 | 0.349 | 0.344 | 0.291 | 0.319 | 0.452 | 0.338 |
| *UBAC2* | rs3825427 | T | 0.264 | 0.149 | 0.367 | 0.418 | 0.178 | 0.315 | 0.398 | 0.267 | 0.262 | 0.351 | 0.308 |
| *UBAC2* | rs9517701 | G | 0.271 | 0.154 | 0.383 | 0.429 | 0.178 | 0.322 | 0.409 | 0.282 | 0.262 | 0.351 | 0.318 |
| *GIMAP4* | rs1916012 | T | 0.463 | 0.537 | 0.461 | 0.335 | 0.500 | 0.513 | 0.570 | 0.485 | 0.529 | 0.490 | 0.495 |
| *GIMAP4* | rs1522596 | T | 0.473 | 0.548 | 0.461 | 0.335 | 0.524 | 0.509 | 0.554 | 0.485 | 0.524 | 0.490 | 0.495 |
| *GIMAP4* | rs1608157 | C | 0.463 | 0.537 | 0.461 | 0.335 | 0.500 | 0.513 | 0.570 | 0.485 | 0.529 | 0.490 | 0.495 |
| *GIMAP2* | rs10266069 | A | 0.408 | 0.447 | 0.438 | 0.271 | 0.466 | 0.501 | 0.548 | 0.500 | 0.476 | 0.457 | 0.530 |
| *GIMAP2* | rs10256482 | T | 0.484 | 0.553 | 0.477 | 0.335 | 0.548 | 0.516 | 0.565 | 0.515 | 0.500 | 0.462 | 0.545 |
| *GIMAP1* | rs2286900 | T | 0.091 | 0.106 | 0.109 | 0.071 | 0.082 | 0.213 | 0.247 | 0.189 | 0.205 | 0.212 | 0.217 |
| *CPLX1* | rs11248047 | A | 0.555 | 0.516 | 0.617 | 0.624 | 0.495 | 0.438 | 0.371 | 0.461 | 0.429 | 0.500 | 0.419 |
| *DEPDC1* | rs6692084 | A | 0.336 | 0.282 | 0.305 | 0.371 | 0.375 | 0.138 | 0.134 | 0.146 | 0.190 | 0.115 | 0.101 |
| *DEPDC1* | rs12134670 | C | 0.032 | 0.032 | 0.023 | 0.012 | 0.053 | 0.099 | 0.097 | 0.107 | 0.124 | 0.091 | 0.076 |
| *DTL* | rs1472224 | G | 0.403 | 0.431 | 0.422 | 0.188 | 0.543 | 0.135 | 0.086 | 0.141 | 0.129 | 0.202 | 0.111 |
| *DNMT3A* | rs1465825 | C | 0.285 | 0.282 | 0.297 | 0.271 | 0.293 | 0.381 | 0.360 | 0.422 | 0.376 | 0.409 | 0.333 |
| *TFCP2L1* | rs17006292 | A | 0.010 | 0.005 | 0 | 0 | 0.029 | 0.032 | 0.022 | 0.044 | 0.014 | 0.058 | 0.020 |
| *PSMD14* | rs6744214 | T | 0.251 | 0.202 | 0.273 | 0.265 | 0.269 | 0.488 | 0.452 | 0.476 | 0.514 | 0.438 | 0.561 |
| *PSMD14* | rs6733456 | C | 0.275 | 0.245 | 0.273 | 0.259 | 0.317 | 0.462 | 0.441 | 0.461 | 0.500 | 0.361 | 0.551 |
| *STK39* | rs2390639 | A | 0.693 | 0.691 | 0.695 | 0.694 | 0.692 | 0.420 | 0.446 | 0.388 | 0.386 | 0.500 | 0.379 |
| *STK39* | rs3769393 | G | 0.723 | 0.755 | 0.711 | 0.694 | 0.726 | 0.492 | 0.543 | 0.447 | 0.495 | 0.534 | 0.444 |
| *SGPP2* | rs17562982 | T | 0.530 | 0.473 | 0.617 | 0.706 | 0.385 | 0.200 | 0.226 | 0.214 | 0.186 | 0.125 | 0.258 |
| *ASB18* | rs7561555 | C | 0.256 | 0.282 | 0.211 | 0.194 | 0.312 | 0.242 | 0.226 | 0.277 | 0.257 | 0.216 | 0.232 |
| *SLIT2* | rs13435197 | A | 0.316 | 0.335 | 0.281 | 0.294 | 0.337 | 0.133 | 0.140 | 0.121 | 0.124 | 0.183 | 0.096 |
| *SORBS2* | rs4493590 | G | 0.137 | 0.176 | 0.164 | 0.071 | 0.139 | 0.246 | 0.237 | 0.262 | 0.262 | 0.250 | 0.217 |
| *MSX2* | rs10516130 | A | 0.274 | 0.229 | 0.281 | 0.312 | 0.279 | 0.165 | 0.215 | 0.189 | 0.167 | 0.101 | 0.157 |
| *C6orf85(LOC100507336)* | rs12194547 | C | 0.035 | 0.021 | 0.078 | 0.012 | 0.038 | 0.104 | 0.048 | 0.136 | 0.095 | 0.188 | 0.045 |
| *ABCB5* | rs2190411 | C | 0.252 | 0.298 | 0.211 | 0.212 | 0.269 | 0.192 | 0.172 | 0.199 | 0.152 | 0.255 | 0.182 |
| *SUSD1* | rs2782932 | T | 0.174 | 0.207 | 0.133 | 0.147 | 0.192 | 0.231 | 0.220 | 0.252 | 0.252 | 0.240 | 0.187 |
| *LINC01499(API5)* | rs420798 | C | 0.703 | 0.846 | 0.633 | 0.506 | 0.779 | 0.490 | 0.473 | 0.471 | 0.462 | 0.505 | 0.540 |
| *API5* | rs16937370 | G | 0 | 0 | 0 | 0 | 0 | 0.168 | 0.124 | 0.136 | 0.200 | 0.202 | 0.172 |
| *SLC43A3* | rs549630 | G | 0.255 | 0.309 | 0.289 | 0.059 | 0.346 | 0.276 | 0.306 | 0.248 | 0.233 | 0.332 | 0.263 |
| *RIMBP2* | rs2895135 | A | 0.197 | 0.261 | 0.156 | 0.088 | 0.255 | 0.182 | 0.204 | 0.155 | 0.152 | 0.183 | 0.217 |
| *GALNTL1* | rs12589991 | A | 0.052 | 0.064 | 0.039 | 0.006 | 0.087 | 0.162 | 0.183 | 0.131 | 0.186 | 0.178 | 0.131 |
| *SMG6* | rs749240 | T | 0.357 | 0.441 | 0.281 | 0.229 | 0.433 | 0.237 | 0.242 | 0.223 | 0.243 | 0.216 | 0.263 |
| *LILRB1* | rs798887 | A | 0.571 | 0.665 | 0.516 | 0.353 | 0.697 | 0.343 | 0.618 | 0.189 | 0.305 | 0.188 | 0.449 |
| *LILRA1* | rs103294 | C | 0.756 | 0.846 | 0.641 | 0.712 | 0.784 | 0.487 | 0.855 | 0.243 | 0.452 | 0.260 | 0.672 |
| *RALGAPA2* | rs6082210 | A | 0.049 | 0.048 | 0.047 | 0.059 | 0.043 | 0.128 | 0.086 | 0.155 | 0.148 | 0.139 | 0.106 |
| *CDH26* | rs817277 | A | 0.298 | 0.277 | 0.234 | 0.212 | 0.428 | 0.289 | 0.409 | 0.204 | 0.229 | 0.260 | 0.359 |
| *CDH26* | rs817283 | A | 0.298 | 0.277 | 0.234 | 0.206 | 0.433 | 0.286 | 0.409 | 0.194 | 0.229 | 0.231 | 0.384 |
| *UBD* | rs6933331 | A | 0.042 | 0.043 | 0.031 | 0.024 | 0.062 | 0.129 | 0.070 | 0.107 | 0.057 | 0.216 | 0.192 |
| *UBD* | rs3025657 | G | 0.042 | 0.043 | 0.031 | 0.024 | 0.062 | 0.129 | 0.070 | 0.107 | 0.057 | 0.216 | 0.192 |
| *GABBR1* | rs29273 | G | 0.800 | 0.782 | 0.734 | 0.871 | 0.798 | 0.954 | 0.984 | 0.956 | 0.976 | 0.894 | 0.965 |
| *MOG* | rs3129045 | T | 0.375 | 0.351 | 0.500 | 0.194 | 0.466 | 0.231 | 0.247 | 0.282 | 0.252 | 0.159 | 0.217 |
| *HLA-F* | rs3116788 | G | 0.265 | 0.287 | 0.250 | 0.176 | 0.327 | 0.308 | 0.306 | 0.330 | 0.290 | 0.250 | 0.364 |
| *HLA-F* | rs1610584 | T | 0.265 | 0.287 | 0.250 | 0.176 | 0.327 | 0.309 | 0.306 | 0.330 | 0.290 | 0.255 | 0.364 |
| *HLA-F* | rs1610585 | C | 0.265 | 0.287 | 0.250 | 0.176 | 0.327 | 0.309 | 0.306 | 0.330 | 0.290 | 0.255 | 0.364 |
| *HLA-F* | rs1610593 | T | 0.265 | 0.287 | 0.250 | 0.176 | 0.327 | 0.309 | 0.306 | 0.330 | 0.290 | 0.255 | 0.364 |
| *HLA-F* | rs1611356 | G | 0.735 | 0.713 | 0.750 | 0.824 | 0.673 | 0.691 | 0.694 | 0.670 | 0.710 | 0.745 | 0.636 |
| *HLA-F* | rs1611381 | T | 0.265 | 0.287 | 0.250 | 0.176 | 0.327 | 0.309 | 0.306 | 0.330 | 0.290 | 0.255 | 0.364 |
| *HLA-F* | rs7741807 | G | 0.981 | 0.979 | 0.992 | 0.988 | 0.971 | 0.895 | 0.930 | 0.903 | 0.948 | 0.784 | 0.914 |
| *HLA-F* | rs1611388 | C | 0.265 | 0.287 | 0.250 | 0.176 | 0.327 | 0.309 | 0.306 | 0.330 | 0.290 | 0.255 | 0.364 |
| *HLA-F* | rs1627465 | C | 0.265 | 0.287 | 0.250 | 0.176 | 0.327 | 0.309 | 0.306 | 0.330 | 0.290 | 0.255 | 0.364 |
| *LOC285830 ( HLA-F antisense RNA1)* | rs9258205 | C | 0.146 | 0.106 | 0.156 | 0.141 | 0.178 | 0.291 | 0.301 | 0.291 | 0.271 | 0.250 | 0.343 |
| *LOC285830 ( HLA-F antisense RNA1)* | rs2523386 | A | 0.125 | 0.154 | 0.156 | 0.059 | 0.135 | 0.041 | 0.011 | 0.039 | 0.014 | 0.106 | 0.030 |
| *LOC285830 ( HLA-F antisense RNA1)* | rs2844845 | A | 0.128 | 0.160 | 0.156 | 0.059 | 0.139 | 0.041 | 0.011 | 0.039 | 0.014 | 0.106 | 0.030 |
| *LOC285830 ( HLA-F antisense RNA1)* | rs1633041 | T | 0.245 | 0.223 | 0.281 | 0.341 | 0.163 | 0.109 | 0.011 | 0.160 | 0.057 | 0.202 | 0.106 |
| *LOC285830 ( HLA-F antisense RNA1)* | rs1737031 | A | 0.280 | 0.271 | 0.289 | 0.341 | 0.231 | 0.187 | 0.081 | 0.238 | 0.110 | 0.303 | 0.192 |
| *LOC285830 ( HLA-F antisense RNA1)* | rs885940 | A | 0.245 | 0.223 | 0.281 | 0.341 | 0.163 | 0.109 | 0.011 | 0.160 | 0.057 | 0.202 | 0.106 |
| *LOC285830 ( HLA-F antisense RNA1)* | rs1610637 | C | 0.245 | 0.223 | 0.281 | 0.341 | 0.163 | 0.109 | 0.011 | 0.160 | 0.057 | 0.202 | 0.106 |
| *LOC285830 ( HLA-F antisense RNA1)* | rs1615251 | T | 0.630 | 0.644 | 0.609 | 0.612 | 0.644 | 0.805 | 0.919 | 0.733 | 0.890 | 0.683 | 0.808 |
| *HLA-G* | rs1633002 | A | 0.759 | 0.777 | 0.734 | 0.665 | 0.837 | 0.893 | 0.989 | 0.850 | 0.943 | 0.798 | 0.894 |
| *HLA-G* | rs1632973 | A | 0.245 | 0.223 | 0.281 | 0.341 | 0.163 | 0.109 | 0.011 | 0.160 | 0.057 | 0.202 | 0.106 |
| *HLA-G* | rs1736963 | T | 0.245 | 0.223 | 0.281 | 0.341 | 0.163 | 0.109 | 0.011 | 0.160 | 0.057 | 0.202 | 0.106 |
| *HLA-G* | rs2523408 | G | 0 | 0 | 0 | 0 | 0 | 0.001 | 0 | 0 | 0.005 | 0 | 0 |
| *HLA-G* | rs1611172 | G | 0.245 | 0.223 | 0.281 | 0.341 | 0.163 | 0.109 | 0.011 | 0.160 | 0.057 | 0.202 | 0.106 |
| *HLA-G* | rs753544 | T | 0.245 | 0.223 | 0.281 | 0.341 | 0.163 | 0.109 | 0.011 | 0.160 | 0.057 | 0.202 | 0.106 |
| *HLA-G* | rs1077433 | A | 0.245 | 0.223 | 0.281 | 0.341 | 0.163 | 0.109 | 0.011 | 0.160 | 0.057 | 0.202 | 0.106 |
| *HLA-G* | rs1736951 | A | 0.346 | 0.319 | 0.383 | 0.406 | 0.298 | 0.120 | 0.011 | 0.199 | 0.057 | 0.216 | 0.106 |
| *HLA-G* | rs407238 | C | 0.252 | 0.293 | 0.234 | 0.282 | 0.202 | 0.127 | 0.011 | 0.175 | 0.076 | 0.207 | 0.157 |
| *HCG9* | rs9260954 | G | 0.035 | 0.037 | 0.062 | 0.012 | 0.034 | 0.036 | 0.011 | 0.029 | 0.010 | 0.101 | 0.025 |
| *HCG9* | rs6911737 | A | 0.174 | 0.165 | 0.266 | 0.100 | 0.188 | 0.190 | 0.091 | 0.194 | 0.095 | 0.303 | 0.263 |
| *HCG9* | rs6926792 | A | 0.174 | 0.165 | 0.266 | 0.100 | 0.188 | 0.188 | 0.086 | 0.194 | 0.095 | 0.303 | 0.258 |
| *HCG9* | rs6931776 | G | 0.174 | 0.165 | 0.266 | 0.100 | 0.188 | 0.190 | 0.091 | 0.194 | 0.095 | 0.303 | 0.263 |
| *ZNRD1* | rs9261189 | T | 0.174 | 0.165 | 0.266 | 0.100 | 0.188 | 0.190 | 0.091 | 0.194 | 0.095 | 0.303 | 0.263 |
| *ZNRD1* | rs3869068 | A | 0.174 | 0.165 | 0.266 | 0.100 | 0.188 | 0.190 | 0.091 | 0.194 | 0.095 | 0.303 | 0.263 |
| *ZNRD1* | rs9261265 | C | 0.040 | 0.048 | 0.062 | 0.018 | 0.038 | 0.036 | 0.011 | 0.029 | 0.010 | 0.101 | 0.025 |
| *PPP1R11* | rs2074482 | T | 0.174 | 0.165 | 0.266 | 0.100 | 0.188 | 0.190 | 0.091 | 0.194 | 0.095 | 0.303 | 0.263 |
| *RNF39* | rs9261317 | A | 0.960 | 0.952 | 0.938 | 0.982 | 0.962 | 0.964 | 0.989 | 0.971 | 0.990 | 0.899 | 0.975 |
| *TRIM31* | rs9261376 | G | 0.256 | 0.266 | 0.336 | 0.141 | 0.293 | 0.208 | 0.091 | 0.223 | 0.110 | 0.303 | 0.308 |
| *TRIM31* | rs9261389 | G | 0.256 | 0.266 | 0.336 | 0.141 | 0.293 | 0.208 | 0.091 | 0.223 | 0.110 | 0.303 | 0.308 |
| *TRIM31* | rs6923832 | A | 0.040 | 0.048 | 0.062 | 0.018 | 0.038 | 0.036 | 0.011 | 0.029 | 0.010 | 0.101 | 0.025 |
| *MUC21* | rs2530710 | A | 0.125 | 0.112 | 0.125 | 0.100 | 0.159 | 0.163 | 0.156 | 0.126 | 0.100 | 0.221 | 0.212 |
| *MUC21* | rs2517446 | C | 0.261 | 0.250 | 0.258 | 0.371 | 0.183 | 0.119 | 0.075 | 0.155 | 0.110 | 0.159 | 0.091 |
| *MUC21* | rs2517411 | G | 0.262 | 0.250 | 0.266 | 0.371 | 0.183 | 0.118 | 0.075 | 0.155 | 0.110 | 0.154 | 0.091 |
| *MUC21* | rs2844673 | A | 0.262 | 0.250 | 0.266 | 0.371 | 0.183 | 0.208 | 0.317 | 0.189 | 0.214 | 0.154 | 0.177 |
| *MUC21* | rs2252925 | G | 0.261 | 0.245 | 0.266 | 0.371 | 0.183 | 0.118 | 0.075 | 0.155 | 0.110 | 0.154 | 0.091 |
| *MUC21* | rs2252926 | G | 0.261 | 0.245 | 0.266 | 0.371 | 0.183 | 0.118 | 0.075 | 0.155 | 0.110 | 0.154 | 0.091 |
| *MUC21* | rs1634717 | T | 0.535 | 0.500 | 0.602 | 0.571 | 0.495 | 0.161 | 0.075 | 0.233 | 0.152 | 0.188 | 0.146 |
| *MUC21* | rs2523915 | T | 0.739 | 0.755 | 0.734 | 0.629 | 0.817 | 0.882 | 0.925 | 0.845 | 0.890 | 0.846 | 0.909 |
| *MUC21* | rs1632854 | T | 0.465 | 0.500 | 0.398 | 0.429 | 0.505 | 0.839 | 0.925 | 0.767 | 0.848 | 0.812 | 0.854 |
| *C6orf15* | rs1265048 | A | 0.520 | 0.516 | 0.477 | 0.382 | 0.663 | 0.475 | 0.263 | 0.510 | 0.457 | 0.606 | 0.520 |
| *PSORS1C1* | rs4959053 | A | 0.091 | 0.080 | 0.070 | 0.147 | 0.067 | 0.128 | 0.172 | 0.107 | 0.095 | 0.082 | 0.192 |
| *CCHCR1* | rs2240063 | A | 0.367 | 0.367 | 0.352 | 0.318 | 0.418 | 0.399 | 0.274 | 0.437 | 0.410 | 0.481 | 0.379 |
| *CCHCR1* | rs2073716 | C | 0.970 | 0.968 | 0.961 | 0.982 | 0.966 | 0.817 | 0.796 | 0.854 | 0.814 | 0.803 | 0.818 |
| *TCF19* | rs2073723 | T | 0.186 | 0.181 | 0.195 | 0.212 | 0.163 | 0.323 | 0.199 | 0.320 | 0.338 | 0.457 | 0.288 |
| *POU5F1* | rs9501063 | G | 0.968 | 0.952 | 0.969 | 0.982 | 0.971 | 0.817 | 0.796 | 0.854 | 0.814 | 0.803 | 0.818 |
| *POU5F1* | rs9263804 | C | 0.192 | 0.181 | 0.203 | 0.218 | 0.173 | 0.323 | 0.199 | 0.320 | 0.338 | 0.457 | 0.288 |
| *POU5F1* | rs3130501 | A | 0.186 | 0.181 | 0.195 | 0.218 | 0.159 | 0.323 | 0.199 | 0.320 | 0.338 | 0.457 | 0.288 |
| *POU5F1* | rs3132524 | A | 0.192 | 0.181 | 0.203 | 0.218 | 0.173 | 0.323 | 0.199 | 0.320 | 0.338 | 0.457 | 0.288 |
| *HCG27* | rs3130944 | C | 0.780 | 0.782 | 0.797 | 0.718 | 0.817 | 0.698 | 0.597 | 0.728 | 0.724 | 0.707 | 0.727 |
| *HLA-C* | rs3905495 | C | 0.546 | 0.590 | 0.594 | 0.506 | 0.510 | 0.594 | 0.715 | 0.612 | 0.629 | 0.404 | 0.626 |
| *DHFRP2* | rs7761068 | T | 0.304 | 0.319 | 0.242 | 0.171 | 0.438 | 0.273 | 0.242 | 0.277 | 0.357 | 0.216 | 0.268 |
| *HLA-B* | rs9266406 | A | 0.223 | 0.186 | 0.273 | 0.229 | 0.221 | 0.391 | 0.457 | 0.354 | 0.357 | 0.361 | 0.434 |
| *HLA-B* | rs9266409 | C | 0.225 | 0.191 | 0.273 | 0.229 | 0.221 | 0.391 | 0.457 | 0.354 | 0.357 | 0.361 | 0.434 |
| *HLA-B* | rs6910516 | C | 0.225 | 0.191 | 0.273 | 0.229 | 0.221 | 0.391 | 0.457 | 0.354 | 0.357 | 0.361 | 0.434 |
| *MICA* | rs2523467 | A | 0.520 | 0.590 | 0.453 | 0.494 | 0.519 | 0.324 | 0.360 | 0.301 | 0.295 | 0.337 | 0.333 |
| *MICA* | rs3094584 | T | 0.187 | 0.191 | 0.164 | 0.100 | 0.269 | 0.119 | 0.032 | 0.131 | 0.048 | 0.303 | 0.071 |
| *BAG6(BAT3)* | rs2077102 | T | 0.187 | 0.181 | 0.148 | 0.276 | 0.144 | 0.130 | 0.065 | 0.165 | 0.105 | 0.264 | 0.040 |
| *C6orf47* | rs2242655 | C | 0.813 | 0.819 | 0.852 | 0.724 | 0.856 | 0.869 | 0.935 | 0.830 | 0.895 | 0.736 | 0.960 |
| *SLC44A4* | rs11965547 | A | 0.183 | 0.197 | 0.180 | 0.265 | 0.106 | 0.148 | 0.070 | 0.209 | 0.100 | 0.284 | 0.066 |
| *C6orf10* | rs544358 | C | 0.501 | 0.415 | 0.539 | 0.718 | 0.380 | 0.361 | 0.306 | 0.398 | 0.410 | 0.365 | 0.318 |
| *C6orf10* | rs574710 | G | 0.517 | 0.431 | 0.570 | 0.735 | 0.385 | 0.382 | 0.344 | 0.408 | 0.433 | 0.370 | 0.348 |
| *C6orf10* | rs539703 | C | 0.500 | 0.415 | 0.531 | 0.718 | 0.380 | 0.361 | 0.306 | 0.398 | 0.410 | 0.365 | 0.318 |
| *C6orf10* | rs926591 | T | 0.496 | 0.399 | 0.531 | 0.718 | 0.380 | 0.360 | 0.301 | 0.398 | 0.410 | 0.365 | 0.318 |
| *C6orf10* | rs4959093 | C | 0.496 | 0.399 | 0.531 | 0.718 | 0.380 | 0.361 | 0.301 | 0.398 | 0.410 | 0.370 | 0.318 |
| *BTNL2* | rs2076530 | G | 0.484 | 0.532 | 0.531 | 0.435 | 0.452 | 0.292 | 0.231 | 0.335 | 0.181 | 0.519 | 0.182 |
| *HLA-DQA1* | rs9272346 | G | 0.321 | 0.447 | 0.289 | 0.171 | 0.351 | 0.492 | 0.640 | 0.422 | 0.452 | 0.510 | 0.449 |
| *HLA-DQB1* | rs6457617 | C | 0.318 | 0.372 | 0.266 | 0.194 | 0.404 | 0.443 | 0.312 | 0.476 | 0.462 | 0.404 | 0.556 |
| *COL12A1* | rs4640857 | G | 0.313 | 0.346 | 0.328 | 0.259 | 0.317 | 0.390 | 0.376 | 0.393 | 0.390 | 0.365 | 0.424 |
| *C10orf11* | rs1323076 | G | 0.460 | 0.532 | 0.367 | 0.441 | 0.466 | 0.188 | 0.156 | 0.204 | 0.176 | 0.216 | 0.182 |
| *C10orf11* | rs17434565 | G | 0.229 | 0.250 | 0.211 | 0.335 | 0.135 | 0.171 | 0.156 | 0.175 | 0.176 | 0.183 | 0.162 |
| *PAX8* | rs11123169 | C | 0.316 | 0.426 | 0.312 | 0.253 | 0.269 | 0.220 | 0.194 | 0.214 | 0.238 | 0.212 | 0.242 |
| *PAX8* | rs10864912 | T | 0.353 | 0.452 | 0.367 | 0.276 | 0.317 | 0.231 | 0.247 | 0.228 | 0.238 | 0.159 | 0.288 |
| *HIVEP3* | rs4660590 | A | 0.693 | 0.707 | 0.680 | 0.800 | 0.601 | 0.311 | 0.269 | 0.296 | 0.290 | 0.385 | 0.308 |
| *CEP135* | rs2593082 | T | 0.640 | 0.601 | 0.648 | 0.718 | 0.606 | 0.385 | 0.360 | 0.374 | 0.405 | 0.447 | 0.333 |
| *CEP135* | rs2611826 | G | 0.362 | 0.394 | 0.352 | 0.282 | 0.404 | 0.567 | 0.597 | 0.597 | 0.529 | 0.505 | 0.616 |
| *HMP19* | rs1909704 | A | 0.595 | 0.644 | 0.570 | 0.600 | 0.562 | 0.440 | 0.457 | 0.447 | 0.429 | 0.409 | 0.465 |
| *TTLL7* | rs11163772 | A | 0.146 | 0.160 | 0.117 | 0.076 | 0.207 | 0.272 | 0.263 | 0.248 | 0.267 | 0.255 | 0.328 |
| *TENM4(ODZ4)* | rs2156215 | T | 0.278 | 0.223 | 0.289 | 0.453 | 0.178 | 0.376 | 0.414 | 0.364 | 0.390 | 0.274 | 0.444 |
| *KLRK1* | rs2617151 | A | 0.125 | 0.138 | 0.109 | 0.041 | 0.192 | 0.214 | 0.269 | 0.155 | 0.190 | 0.207 | 0.258 |
| *KLRK1* | rs2733852 | G | 0.228 | 0.245 | 0.133 | 0.071 | 0.399 | 0.262 | 0.344 | 0.209 | 0.219 | 0.226 | 0.323 |
| *OSR1* | rs4666492 | G | 0.343 | 0.394 | 0.320 | 0.224 | 0.409 | 0.197 | 0.215 | 0.160 | 0.186 | 0.183 | 0.247 |
| *CTNNA2* | rs4852547 | G | 0.441 | 0.457 | 0.500 | 0.329 | 0.481 | 0.154 | 0.145 | 0.194 | 0.143 | 0.135 | 0.152 |
| *MN1* | rs134006 | C | 0.190 | 0.160 | 0.219 | 0.288 | 0.120 | 0.279 | 0.349 | 0.214 | 0.290 | 0.260 | 0.288 |
| *LTN1(RNF160)* | rs2832137 | T | 0.418 | 0.367 | 0.453 | 0.476 | 0.394 | 0.172 | 0.151 | 0.189 | 0.138 | 0.202 | 0.177 |
| *HERPUD2* | rs11763983 | T | 0.427 | 0.463 | 0.383 | 0.424 | 0.423 | 0.234 | 0.220 | 0.272 | 0.210 | 0.250 | 0.217 |
| *GALNT10* | rs574750 | A | 0.215 | 0.261 | 0.195 | 0.088 | 0.288 | 0.144 | 0.156 | 0.131 | 0.129 | 0.159 | 0.146 |
| *SAMD3(TMEM200A)* | rs9483115 | T | 0.445 | 0.415 | 0.492 | 0.476 | 0.418 | 0.473 | 0.538 | 0.364 | 0.443 | 0.534 | 0.495 |
| *SAMD3(TMEM200A)* | rs4141940 | A | 0.435 | 0.399 | 0.500 | 0.465 | 0.404 | 0.473 | 0.538 | 0.364 | 0.443 | 0.534 | 0.495 |
| *SAMD3(TMEM200A)* | rs899276 | A | 0.434 | 0.394 | 0.500 | 0.465 | 0.404 | 0.474 | 0.543 | 0.364 | 0.443 | 0.534 | 0.495 |
| *SAMD3(TMEM200A)* | rs7758496 | G | 0.44 | 0.41 | 0.508 | 0.476 | 0.42 | 0.47 | 0.538 | 0.36 | 0.44 | 0.534 | 0.48 |
| *SAMD3(TMEM200A)* | rs724324 | G | 0.445 | 0.410 | 0.500 | 0.476 | 0.418 | 0.473 | 0.538 | 0.364 | 0.443 | 0.534 | 0.495 |
| *SAMD3* | rs4897380 | C | 0.45 | 0.43 | 0.523 | 0.471 | 0.41 | 0.48 | 0.522 | 0.40 | 0.45 | 0.577 | 0.46 |
| *SEMA6D* | rs470151 | T | 0.159 | 0.133 | 0.172 | 0.259 | 0.091 | 0.259 | 0.269 | 0.248 | 0.281 | 0.144 | 0.359 |
| *PMFBP1* | rs11862324 | T | 0.476 | 0.367 | 0.484 | 0.735 | 0.356 | 0.455 | 0.376 | 0.505 | 0.529 | 0.495 | 0.359 |
| *NAV2* | rs2707110 | C | 0.354 | 0.335 | 0.336 | 0.382 | 0.361 | 0.338 | 0.301 | 0.335 | 0.329 | 0.332 | 0.394 |
| *NAV2* | rs873764 | G | 0.500 | 0.457 | 0.469 | 0.624 | 0.457 | 0.408 | 0.376 | 0.432 | 0.405 | 0.394 | 0.429 |
| *TMEM132B* | rs4435061 | A | 0.341 | 0.372 | 0.352 | 0.329 | 0.317 | 0.476 | 0.468 | 0.490 | 0.486 | 0.438 | 0.500 |
| *TMEM132B* | rs10846917 | T | 0.588 | 0.574 | 0.570 | 0.571 | 0.625 | 0.376 | 0.430 | 0.364 | 0.357 | 0.346 | 0.389 |
| *TMEM132B* | rs10846924 | T | 0.290 | 0.314 | 0.328 | 0.329 | 0.212 | 0.476 | 0.468 | 0.485 | 0.486 | 0.442 | 0.500 |
| *STX8* | rs1549332 | A | 0.104 | 0.128 | 0.117 | 0.035 | 0.130 | 0.091 | 0.070 | 0.078 | 0.086 | 0.149 | 0.071 |
| *OVCH1* | rs1436321 | A | 0.274 | 0.266 | 0.188 | 0.194 | 0.399 | 0.465 | 0.516 | 0.456 | 0.467 | 0.365 | 0.530 |
| *SLC41A2* | rs2731031 | A | 0.284 | 0.261 | 0.312 | 0.259 | 0.308 | 0.390 | 0.414 | 0.422 | 0.381 | 0.322 | 0.414 |
| *HNF4G* | rs2980221 | A | 0.431 | 0.521 | 0.391 | 0.259 | 0.514 | 0.343 | 0.323 | 0.350 | 0.338 | 0.346 | 0.359 |
| *SMARCA2* | rs7033529 | A | 0.761 | 0.729 | 0.742 | 0.700 | 0.851 | 0.538 | 0.527 | 0.539 | 0.557 | 0.510 | 0.556 |
| *EBF2* | rs4570167 | C | 0.363 | 0.335 | 0.359 | 0.253 | 0.481 | 0.250 | 0.290 | 0.228 | 0.238 | 0.202 | 0.298 |
| *EBF2* | rs4242425 | T | 0.362 | 0.330 | 0.359 | 0.253 | 0.481 | 0.250 | 0.290 | 0.228 | 0.238 | 0.202 | 0.298 |
| *GAS2* | rs10833804 | G | 0.715 | 0.777 | 0.727 | 0.618 | 0.731 | 0.400 | 0.387 | 0.325 | 0.371 | 0.486 | 0.429 |
| *LYST/NID1* | rs7354999 | G | 0.885 | 0.910 | 0.891 | 0.824 | 0.909 | 0.364 | 0.371 | 0.320 | 0.357 | 0.404 | 0.369 |
| *LOC100132252* | rs9469615 | C | 0.086 | 0.069 | 0.078 | 0.053 | 0.135 | 0.058 | 0.005 | 0.053 | 0.019 | 0.173 | 0.030 |
| *LOC107984355* | rs872837 | A | 0.370 | 0.335 | 0.352 | 0.441 | 0.356 | 0.240 | 0.290 | 0.233 | 0.243 | 0.197 | 0.242 |
| *SACM1L* | rs1969624 | C | 0.454 | 0.410 | 0.445 | 0.382 | 0.558 | 0.265 | 0.317 | 0.204 | 0.224 | 0.240 | 0.348 |
| *PLEKHB1* | rs591804 | G | 0.329 | 0.319 | 0.328 | 0.306 | 0.356 | 0.310 | 0.306 | 0.350 | 0.290 | 0.317 | 0.283 |
| *ATP8A1* | rs2100766 | T | 0.082 | 0.085 | 0.047 | 0.094 | 0.091 | 0.156 | 0.113 | 0.170 | 0.167 | 0.188 | 0.136 |
| *KCNK9* | rs1961261 | A | 0.226 | 0.207 | 0.250 | 0.218 | 0.236 | 0.233 | 0.253 | 0.282 | 0.148 | 0.216 | 0.273 |
| *SUMO4* | rs237024 | C | 0.615 | 0.532 | 0.594 | 0.700 | 0.635 | 0.736 | 0.753 | 0.728 | 0.714 | 0.726 | 0.763 |

D. 1000 Genomes European and South Asian populations

| **Gene** | **Variant/SNP** | **Behcet Allele** | **EUR** | CEU | FIN | GBR | IBS | TSI | **SAS** | BEB | GIH | ITU | PJL | STU |
| --- | --- | --- | --- | --- | --- | --- | --- | --- | --- | --- | --- | --- | --- | --- |
| *IL-10* | rs1518111 | A | 0.221 | 0.202 | 0.207 | 0.176 | 0.234 | 0.276 | 0.443 | 0.459 | 0.403 | 0.436 | 0.443 | 0.475 |
| *IL-10* | rs1800871 | T | 0.24 | 0.207 | 0.237 | 0.187 | 0.262 | 0.294 | 0.458 | 0.494 | 0.408 | 0.456 | 0.448 | 0.49 |
| *IL-10* | rs1800872 | A | 0.240 | 0.207 | 0.237 | 0.187 | 0.262 | 0.294 | 0.458 | 0.494 | 0.408 | 0.456 | 0.448 | 0.490 |
| *IL-10* | rs1554286 | C | 0.815 | 0.833 | 0.813 | 0.841 | 0.818 | 0.776 | 0.582 | 0.547 | 0.621 | 0.574 | 0.620 | 0.544 |
| *IL23R,IL12RB2* | rs1495965 | G | 0.480 | 0.424 | 0.646 | 0.445 | 0.439 | 0.449 | 0.537 | 0.599 | 0.529 | 0.505 | 0.484 | 0.574 |
| *IL23R,IL12RB2* | rs924080 | T | 0.553 | 0.515 | 0.697 | 0.555 | 0.505 | 0.500 | 0.661 | 0.703 | 0.655 | 0.598 | 0.677 | 0.676 |
| *IL23R,IL12RB2* | rs12119179 | A | 0.665 | 0.697 | 0.611 | 0.698 | 0.664 | 0.659 | 0.475 | 0.407 | 0.481 | 0.510 | 0.536 | 0.436 |
| *IL23R,IL12RB2* | rs11209033 | C | 0.665 | 0.697 | 0.611 | 0.698 | 0.664 | 0.659 | 0.478 | 0.407 | 0.485 | 0.510 | 0.542 | 0.436 |
| *IL23R,IL12RB2* | rs12141431 | C | 0.304 | 0.288 | 0.323 | 0.275 | 0.313 | 0.318 | 0.488 | 0.547 | 0.461 | 0.471 | 0.453 | 0.515 |
| *TNFAIP3* | rs9494885 | T | 0.913 | 0.904 | 0.960 | 0.907 | 0.916 | 0.879 | 0.902 | 0.924 | 0.917 | 0.922 | 0.875 | 0.873 |
| *TNFAIP3* | rs10499194 | C | 0.720 | 0.727 | 0.763 | 0.720 | 0.654 | 0.738 | 0.715 | 0.797 | 0.636 | 0.725 | 0.682 | 0.745 |
| *TNFAIP3* | rs610604 | A | 0.662 | 0.601 | 0.707 | 0.654 | 0.650 | 0.696 | 0.674 | 0.715 | 0.689 | 0.637 | 0.661 | 0.672 |
| *TNFAIP3* | rs7753873 | C | 0.087 | 0.096 | 0.040 | 0.093 | 0.084 | 0.121 | 0.101 | 0.081 | 0.083 | 0.078 | 0.125 | 0.137 |
| *STAT4* | rs7574070 | A | 0.342 | 0.343 | 0.313 | 0.379 | 0.318 | 0.360 | 0.314 | 0.366 | 0.291 | 0.343 | 0.297 | 0.279 |
| *STAT4* | rs897200 | A | 0.357 | 0.364 | 0.313 | 0.401 | 0.341 | 0.369 | 0.319 | 0.372 | 0.301 | 0.343 | 0.292 | 0.294 |
| *STAT4* | rs7572482 | A | 0.343 | 0.343 | 0.313 | 0.385 | 0.318 | 0.360 | 0.315 | 0.360 | 0.296 | 0.343 | 0.292 | 0.289 |
| *CCR1* | rs17282391 | G | 0.094 | 0.101 | 0.141 | 0.099 | 0.061 | 0.075 | 0.293 | 0.209 | 0.325 | 0.275 | 0.359 | 0.289 |
| *CCR1* | rs10510749 | T | 0.094 | 0.101 | 0.141 | 0.099 | 0.061 | 0.075 | 0.292 | 0.209 | 0.325 | 0.275 | 0.359 | 0.284 |
| *CCR1* | rs13084057 | G | 0.094 | 0.101 | 0.141 | 0.099 | 0.061 | 0.075 | 0.293 | 0.209 | 0.325 | 0.275 | 0.359 | 0.289 |
| *CCR1* | rs7631551 | A | 0.100 | 0.101 | 0.141 | 0.099 | 0.079 | 0.084 | 0.293 | 0.209 | 0.325 | 0.275 | 0.365 | 0.284 |
| *CCR1* | rs7616215 | T | 0.643 | 0.616 | 0.616 | 0.648 | 0.631 | 0.701 | 0.549 | 0.640 | 0.519 | 0.525 | 0.500 | 0.574 |
| *CCR3* | rs7649764 | C | 0.735 | 0.753 | 0.697 | 0.769 | 0.785 | 0.673 | 0.628 | 0.570 | 0.675 | 0.603 | 0.656 | 0.627 |
| *CCR3* | rs9990343 | G | 0.507 | 0.510 | 0.566 | 0.571 | 0.509 | 0.393 | 0.472 | 0.378 | 0.515 | 0.490 | 0.516 | 0.451 |
| *CCR3* | rs6803980 | A | 0.506 | 0.510 | 0.566 | 0.571 | 0.509 | 0.388 | 0.466 | 0.384 | 0.515 | 0.480 | 0.495 | 0.446 |
| *CCR3* | rs13075270 | C | 0.098 | 0.101 | 0.131 | 0.099 | 0.070 | 0.093 | 0.299 | 0.221 | 0.354 | 0.265 | 0.370 | 0.275 |
| *CCR3* | rs13092160 | C | 0.092 | 0.101 | 0.131 | 0.099 | 0.061 | 0.075 | 0.299 | 0.221 | 0.354 | 0.265 | 0.370 | 0.275 |
| *CCR3* | rs2373156 | T | 0.089 | 0.091 | 0.106 | 0.077 | 0.075 | 0.098 | 0.276 | 0.203 | 0.316 | 0.240 | 0.349 | 0.265 |
| *CCR3* | rs7651539 | T | 0.089 | 0.091 | 0.106 | 0.077 | 0.075 | 0.098 | 0.276 | 0.203 | 0.316 | 0.240 | 0.349 | 0.265 |
| *CCR3* | rs1542755 | A | 0.080 | 0.091 | 0.106 | 0.077 | 0.051 | 0.075 | 0.272 | 0.215 | 0.311 | 0.245 | 0.312 | 0.270 |
| *CCR3* | rs13067058 | A | 0.078 | 0.096 | 0.111 | 0.071 | 0.047 | 0.065 | 0.253 | 0.198 | 0.301 | 0.211 | 0.297 | 0.250 |
| *CCR3* | rs13092160 | C | 0.092 | 0.101 | 0.131 | 0.099 | 0.061 | 0.075 | 0.299 | 0.221 | 0.354 | 0.265 | 0.370 | 0.275 |
| *KLRC4* | rs2617170 | C | 0.663 | 0.687 | 0.591 | 0.720 | 0.696 | 0.626 | 0.544 | 0.552 | 0.515 | 0.515 | 0.589 | 0.554 |
| *ERAP1* | rs17482078 | T | 0.224 | 0.263 | 0.227 | 0.253 | 0.182 | 0.201 | 0.065 | 0.058 | 0.058 | 0.078 | 0.068 | 0.064 |
| *FUT2* | rs681343 | T | 0.440 | 0.535 | 0.298 | 0.473 | 0.425 | 0.472 | 0.283 | 0.238 | 0.252 | 0.240 | 0.427 | 0.260 |
| *IL12A* | rs17810546 | A | 0.906 | 0.909 | 0.894 | 0.885 | 0.916 | 0.921 | 0.967 | 0.983 | 0.942 | 0.966 | 0.958 | 0.990 |
| *IL23R* | rs11209026 | A | 0.062 | 0.051 | 0.030 | 0.071 | 0.065 | 0.089 | 0.012 | 0.006 | 0.010 | 0.015 | 0.016 | 0.015 |
| *IL23R* | rs76418789 | A | 0.002 | 0 | 0 | 0.0055 | 0.0047 | 0 | 0 | 0 | 0 | 0 | 0 | 0 |
| *IL23R* | rs17375018 | G | 0.714 | 0.717 | 0.697 | 0.747 | 0.664 | 0.748 | 0.686 | 0.733 | 0.660 | 0.667 | 0.714 | 0.667 |
| *IL23R* | rs11209032 | A | 0.334 | 0.303 | 0.389 | 0.297 | 0.336 | 0.341 | 0.525 | 0.593 | 0.515 | 0.495 | 0.458 | 0.569 |
| *IL23R* | rs1343151 | T | 0.322 | 0.343 | 0.207 | 0.363 | 0.336 | 0.360 | 0.164 | 0.122 | 0.155 | 0.167 | 0.203 | 0.167 |
| *TLR4* | rs4986790 | G | 0.057 | 0.040 | 0.116 | 0.044 | 0.037 | 0.047 | 0.126 | 0.134 | 0.102 | 0.142 | 0.109 | 0.142 |
| *TLR4* | rs4986791 | T | 0.058 | 0.040 | 0.116 | 0.038 | 0.047 | 0.047 | 0.117 | 0.093 | 0.092 | 0.147 | 0.078 | 0.167 |
| *NOD2* | rs2066844 | T | 0.051 | 0.071 | 0.030 | 0.049 | 0.056 | 0.047 | 0.001 | 0.006 | 0 | 0 | 0 | 0 |
| *NOD2* | rs2066845 | C | 0.010 | 0.020 | 0 | 0.005 | 0.009 | 0.014 | 0.004 | 0 | 0 | 0.005 | 0.016 | 0 |
| *NOD2* | rs2066847 | ins-C | 0.014 | 0.030 | 0 | 0.022 | 0.014 | 0.005 | 0 | 0 | 0 | 0 | 0 | 0 |
| *IL1* | rs1800587 | C | 0.713 | 0.747 | 0.722 | 0.637 | 0.743 | 0.706 | 0.685 | 0.715 | 0.704 | 0.642 | 0.693 | 0.676 |
| *IL1* | rs1143634 | T | 0.248 | 0.232 | 0.237 | 0.313 | 0.196 | 0.266 | 0.147 | 0.122 | 0.194 | 0.132 | 0.146 | 0.137 |
| *IL1* | rs16944 | G | 0.650 | 0.652 | 0.626 | 0.681 | 0.673 | 0.621 | 0.400 | 0.401 | 0.403 | 0.382 | 0.438 | 0.377 |
| *TNFα* | rs1799964 | C | 0.210 | 0.212 | 0.202 | 0.220 | 0.178 | 0.238 | 0.348 | 0.436 | 0.383 | 0.373 | 0.286 | 0.270 |
| *TNFα* | rs361525 | A | 0.064 | 0.066 | 0.040 | 0.077 | 0.051 | 0.084 | 0.105 | 0.105 | 0.170 | 0.132 | 0.047 | 0.069 |
| *TNFα* | rs1799724 | T | 0.094 | 0.056 | 0.066 | 0.077 | 0.131 | 0.136 | 0.119 | 0.076 | 0.107 | 0.118 | 0.151 | 0.137 |
| *IL12* | rs3212227 | A | 0.777 | 0.808 | 0.813 | 0.753 | 0.780 | 0.734 | 0.626 | 0.674 | 0.655 | 0.583 | 0.667 | 0.559 |
| *IL18* | rs1946518 | C | 0.577 | 0.636 | 0.545 | 0.621 | 0.579 | 0.509 | 0.712 | 0.750 | 0.772 | 0.691 | 0.646 | 0.701 |
| *IL17F-A126G* | rs2397084 | T | 0.921 | 0.924 | 0.934 | 0.907 | 0.935 | 0.907 | 0.953 | 0.953 | 0.961 | 0.956 | 0.938 | 0.956 |
| *LOC100129342* | rs11206377 | G | 0.542 | 0.596 | 0.530 | 0.533 | 0.500 | 0.551 | 0.802 | 0.756 | 0.859 | 0.789 | 0.766 | 0.828 |
| *CCDC180* | rs2061634 | G | 0.200 | 0.237 | 0.121 | 0.209 | 0.215 | 0.215 | 0.261 | 0.227 | 0.223 | 0.275 | 0.328 | 0.250 |
| *CPVL* | rs317711 | C | 0.249 | 0.247 | 0.247 | 0.236 | 0.234 | 0.276 | 0.095 | 0.087 | 0.034 | 0.098 | 0.120 | 0.137 |
| *UBASH3B* | rs4936742 | T | 0.419 | 0.369 | 0.328 | 0.451 | 0.439 | 0.505 | 0.458 | 0.465 | 0.485 | 0.475 | 0.396 | 0.466 |
| *UBAC2* | rs9513584 | G | 0.273 | 0.202 | 0.359 | 0.258 | 0.234 | 0.313 | 0.593 | 0.558 | 0.597 | 0.627 | 0.536 | 0.637 |
| *UBAC2* | rs9517644 | T | 0.276 | 0.202 | 0.359 | 0.264 | 0.238 | 0.318 | 0.594 | 0.552 | 0.602 | 0.623 | 0.552 | 0.632 |
| *UBAC2* | rs11069357 | A | 0.277 | 0.202 | 0.359 | 0.264 | 0.238 | 0.322 | 0.594 | 0.552 | 0.602 | 0.623 | 0.552 | 0.632 |
| *UBAC2* | rs984477 | G | 0.341 | 0.308 | 0.455 | 0.319 | 0.290 | 0.336 | 0.599 | 0.570 | 0.602 | 0.627 | 0.547 | 0.642 |
| *UBAC2* | rs9554573 | A | 0.275 | 0.202 | 0.364 | 0.264 | 0.234 | 0.313 | 0.603 | 0.576 | 0.597 | 0.642 | 0.547 | 0.647 |
| *UBAC2* | rs6491493 | G | 0.273 | 0.202 | 0.359 | 0.258 | 0.234 | 0.313 | 0.599 | 0.576 | 0.607 | 0.627 | 0.542 | 0.637 |
| *UBAC2* | rs9517668 | T | 0.115 | 0.096 | 0.146 | 0.137 | 0.075 | 0.126 | 0.168 | 0.174 | 0.165 | 0.176 | 0.151 | 0.172 |
| *UBAC2* | rs7999348 | G | 0.285 | 0.202 | 0.364 | 0.286 | 0.243 | 0.332 | 0.677 | 0.651 | 0.650 | 0.725 | 0.604 | 0.745 |
| *UBAC2* | rs9554581 | T | 0.114 | 0.096 | 0.146 | 0.137 | 0.075 | 0.121 | 0.166 | 0.169 | 0.165 | 0.176 | 0.146 | 0.172 |
| *UBAC2* | rs17575643 | T | 0.100 | 0.091 | 0.131 | 0.126 | 0.070 | 0.089 | 0.109 | 0.110 | 0.121 | 0.118 | 0.062 | 0.132 |
| *UBAC2* | rs727263 | A | 0.114 | 0.096 | 0.146 | 0.137 | 0.075 | 0.121 | 0.163 | 0.169 | 0.155 | 0.172 | 0.146 | 0.172 |
| *UBAC2* | rs7332161 | A | 0.114 | 0.096 | 0.146 | 0.137 | 0.075 | 0.121 | 0.167 | 0.174 | 0.165 | 0.176 | 0.146 | 0.172 |
| *UBAC2* | rs912130 | C | 0.274 | 0.207 | 0.364 | 0.258 | 0.234 | 0.308 | 0.595 | 0.570 | 0.592 | 0.627 | 0.542 | 0.637 |
| *UBAC2* | rs2892976 | G | 0.202 | 0.162 | 0.247 | 0.203 | 0.150 | 0.248 | 0.317 | 0.250 | 0.306 | 0.338 | 0.292 | 0.387 |
| *UBAC2* | rs3825427 | T | 0.116 | 0.096 | 0.146 | 0.143 | 0.075 | 0.126 | 0.178 | 0.186 | 0.175 | 0.181 | 0.167 | 0.181 |
| *UBAC2* | rs9517701 | G | 0.115 | 0.096 | 0.146 | 0.137 | 0.079 | 0.121 | 0.167 | 0.174 | 0.165 | 0.176 | 0.146 | 0.172 |
| *GIMAP4* | rs1916012 | T | 0.554 | 0.525 | 0.475 | 0.637 | 0.584 | 0.551 | 0.551 | 0.465 | 0.587 | 0.529 | 0.573 | 0.588 |
| *GIMAP4* | rs1522596 | T | 0.555 | 0.525 | 0.475 | 0.637 | 0.589 | 0.551 | 0.551 | 0.465 | 0.587 | 0.529 | 0.573 | 0.588 |
| *GIMAP4* | rs1608157 | C | 0.554 | 0.525 | 0.475 | 0.637 | 0.584 | 0.551 | 0.551 | 0.465 | 0.587 | 0.529 | 0.573 | 0.588 |
| *GIMAP2* | rs10266069 | A | 0.494 | 0.485 | 0.455 | 0.489 | 0.514 | 0.523 | 0.356 | 0.366 | 0.393 | 0.324 | 0.349 | 0.348 |
| *GIMAP2* | rs10256482 | T | 0.597 | 0.556 | 0.571 | 0.654 | 0.603 | 0.607 | 0.463 | 0.430 | 0.505 | 0.412 | 0.500 | 0.466 |
| *GIMAP1* | rs2286900 | T | 0.092 | 0.096 | 0.126 | 0.082 | 0.070 | 0.089 | 0.090 | 0.064 | 0.083 | 0.127 | 0.099 | 0.074 |
| *CPLX1* | rs11248047 | A | 0.466 | 0.480 | 0.500 | 0.505 | 0.453 | 0.402 | 0.359 | 0.355 | 0.427 | 0.363 | 0.328 | 0.319 |
| *DEPDC1* | rs6692084 | A | 0.232 | 0.242 | 0.308 | 0.187 | 0.210 | 0.210 | 0.214 | 0.198 | 0.214 | 0.186 | 0.245 | 0.225 |
| *DEPDC1* | rs12134670 | C | 0.082 | 0.076 | 0.126 | 0.066 | 0.065 | 0.075 | 0.122 | 0.087 | 0.112 | 0.137 | 0.141 | 0.127 |
| *DTL* | rs1472224 | G | 0.597 | 0.616 | 0.465 | 0.670 | 0.621 | 0.617 | 0.474 | 0.384 | 0.481 | 0.495 | 0.495 | 0.505 |
| *DNMT3A* | rs1465825 | C | 0.262 | 0.258 | 0.227 | 0.236 | 0.322 | 0.262 | 0.451 | 0.465 | 0.413 | 0.485 | 0.432 | 0.461 |
| *TFCP2L1* | rs17006292 | A | 0.002 | 0 | 0 | 0 | 0.005 | 0.005 | 0.043 | 0.070 | 0.053 | 0.054 | 0.010 | 0.029 |
| *PSMD14* | rs6744214 | T | 0.313 | 0.227 | 0.414 | 0.330 | 0.304 | 0.294 | 0.415 | 0.453 | 0.403 | 0.412 | 0.396 | 0.417 |
| *PSMD14* | rs6733456 | C | 0.317 | 0.232 | 0.419 | 0.330 | 0.304 | 0.304 | 0.406 | 0.442 | 0.374 | 0.412 | 0.391 | 0.417 |
| *STK39* | rs2390639 | A | 0.790 | 0.833 | 0.753 | 0.808 | 0.808 | 0.752 | 0.681 | 0.645 | 0.646 | 0.740 | 0.703 | 0.667 |
| *STK39* | rs3769393 | G | 0.818 | 0.843 | 0.808 | 0.808 | 0.841 | 0.790 | 0.734 | 0.686 | 0.728 | 0.765 | 0.750 | 0.735 |
| *SGPP2* | rs17562982 | T | 0.402 | 0.384 | 0.333 | 0.396 | 0.388 | 0.500 | 0.500 | 0.442 | 0.529 | 0.529 | 0.443 | 0.544 |
| *ASB18* | rs7561555 | C | 0.304 | 0.343 | 0.354 | 0.247 | 0.285 | 0.290 | 0.383 | 0.419 | 0.393 | 0.348 | 0.380 | 0.382 |
| *SLIT2* | rs13435197 | A | 0.397 | 0.419 | 0.379 | 0.396 | 0.355 | 0.435 | 0.297 | 0.262 | 0.257 | 0.333 | 0.354 | 0.275 |
| *SORBS2* | rs4493590 | G | 0.280 | 0.343 | 0.323 | 0.269 | 0.271 | 0.201 | 0.199 | 0.256 | 0.218 | 0.137 | 0.182 | 0.211 |
| *MSX2* | rs10516130 | A | 0.141 | 0.126 | 0.141 | 0.176 | 0.131 | 0.136 | 0.203 | 0.215 | 0.194 | 0.255 | 0.198 | 0.157 |
| *C6orf85(LOC100507336)* | rs12194547 | C | 0.062 | 0.066 | 0.081 | 0.082 | 0.065 | 0.019 | 0.096 | 0.116 | 0.068 | 0.064 | 0.120 | 0.118 |
| *ABCB5* | rs2190411 | C | 0.273 | 0.283 | 0.187 | 0.269 | 0.262 | 0.360 | 0.192 | 0.169 | 0.194 | 0.181 | 0.219 | 0.196 |
| *SUSD1* | rs2782932 | T | 0.181 | 0.157 | 0.192 | 0.165 | 0.196 | 0.192 | 0.158 | 0.134 | 0.146 | 0.142 | 0.161 | 0.206 |
| *LINC01499(API5)* | rs420798 | C | 0.925 | 0.934 | 0.924 | 0.923 | 0.911 | 0.935 | 0.882 | 0.831 | 0.898 | 0.892 | 0.896 | 0.887 |
| *API5* | rs16937370 | G | 0 | 0 | 0 | 0 | 0 | 0 | 0.026 | 0.047 | 0.010 | 0.020 | 0.036 | 0.020 |
| *SLC43A3* | rs549630 | G | 0.380 | 0.449 | 0.253 | 0.407 | 0.393 | 0.397 | 0.223 | 0.186 | 0.228 | 0.206 | 0.229 | 0.260 |
| *RIMBP2* | rs2895135 | A | 0.324 | 0.303 | 0.384 | 0.302 | 0.374 | 0.257 | 0.178 | 0.215 | 0.136 | 0.162 | 0.219 | 0.167 |
| *GALNTL1* | rs12589991 | A | 0.142 | 0.091 | 0.202 | 0.143 | 0.164 | 0.112 | 0.087 | 0.099 | 0.117 | 0.069 | 0.083 | 0.069 |
| *SMG6* | rs749240 | T | 0.362 | 0.328 | 0.338 | 0.352 | 0.369 | 0.416 | 0.378 | 0.384 | 0.413 | 0.368 | 0.312 | 0.412 |
| *LILRB1* | rs798887 | A | 0.810 | 0.823 | 0.682 | 0.802 | 0.836 | 0.897 | 0.852 | 0.767 | 0.898 | 0.902 | 0.818 | 0.858 |
| *LILRA1* | rs103294 | C | 0.820 | 0.833 | 0.702 | 0.824 | 0.836 | 0.897 | 0.872 | 0.820 | 0.908 | 0.917 | 0.828 | 0.877 |
| *RALGAPA2* | rs6082210 | A | 0.046 | 0.025 | 0.141 | 0.038 | 0.009 | 0.019 | 0.077 | 0.058 | 0.092 | 0.078 | 0.083 | 0.069 |
| *CDH26* | rs817277 | A | 0.431 | 0.500 | 0.409 | 0.407 | 0.416 | 0.425 | 0.399 | 0.401 | 0.383 | 0.373 | 0.411 | 0.426 |
| *CDH26* | rs817283 | A | 0.433 | 0.500 | 0.424 | 0.401 | 0.421 | 0.421 | 0.403 | 0.424 | 0.388 | 0.373 | 0.411 | 0.422 |
| *UBD* | rs6933331 | A | 0.012 | 0.010 | 0.005 | 0 | 0.023 | 0.019 | 0.063 | 0.093 | 0.058 | 0.044 | 0.057 | 0.069 |
| *UBD* | rs3025657 | G | 0.012 | 0.010 | 0.005 | 0 | 0.023 | 0.019 | 0.063 | 0.093 | 0.058 | 0.044 | 0.057 | 0.069 |
| *GABBR1* | rs29273 | G | 0.813 | 0.889 | 0.894 | 0.786 | 0.724 | 0.780 | 0.863 | 0.901 | 0.850 | 0.907 | 0.792 | 0.868 |
| *MOG* | rs3129045 | T | 0.347 | 0.247 | 0.212 | 0.385 | 0.472 | 0.407 | 0.333 | 0.413 | 0.286 | 0.294 | 0.354 | 0.333 |
| *HLA-F* | rs3116788 | G | 0.304 | 0.298 | 0.429 | 0.291 | 0.234 | 0.276 | 0.349 | 0.390 | 0.320 | 0.328 | 0.354 | 0.358 |
| *HLA-F* | rs1610584 | T | 0.304 | 0.298 | 0.429 | 0.291 | 0.234 | 0.276 | 0.349 | 0.390 | 0.320 | 0.328 | 0.354 | 0.358 |
| *HLA-F* | rs1610585 | C | 0.304 | 0.298 | 0.429 | 0.291 | 0.234 | 0.276 | 0.349 | 0.390 | 0.320 | 0.328 | 0.354 | 0.358 |
| *HLA-F* | rs1610593 | T | 0.304 | 0.298 | 0.429 | 0.291 | 0.234 | 0.276 | 0.350 | 0.390 | 0.320 | 0.333 | 0.354 | 0.358 |
| *HLA-F* | rs1611356 | G | 0.696 | 0.702 | 0.571 | 0.709 | 0.766 | 0.724 | 0.650 | 0.610 | 0.680 | 0.667 | 0.646 | 0.642 |
| *HLA-F* | rs1611381 | T | 0.304 | 0.298 | 0.429 | 0.291 | 0.234 | 0.276 | 0.350 | 0.390 | 0.320 | 0.333 | 0.354 | 0.358 |
| *HLA-F* | rs7741807 | G | 0.978 | 0.980 | 0.975 | 0.978 | 0.991 | 0.967 | 0.921 | 0.942 | 0.927 | 0.931 | 0.891 | 0.917 |
| *HLA-F* | rs1611388 | C | 0.302 | 0.298 | 0.419 | 0.291 | 0.234 | 0.276 | 0.350 | 0.390 | 0.320 | 0.333 | 0.354 | 0.358 |
| *HLA-F* | rs1627465 | C | 0.304 | 0.298 | 0.429 | 0.291 | 0.234 | 0.276 | 0.353 | 0.407 | 0.320 | 0.333 | 0.354 | 0.358 |
| *LOC285830 ( HLA-F antisense RNA1)* | rs9258205 | C | 0.115 | 0.121 | 0.111 | 0.159 | 0.079 | 0.112 | 0.287 | 0.343 | 0.262 | 0.279 | 0.302 | 0.260 |
| *LOC285830 ( HLA-F antisense RNA1)* | rs2523386 | A | 0.174 | 0.096 | 0.126 | 0.187 | 0.234 | 0.220 | 0.081 | 0.041 | 0.117 | 0.039 | 0.130 | 0.074 |
| *LOC285830 ( HLA-F antisense RNA1)* | rs2844845 | A | 0.179 | 0.096 | 0.126 | 0.187 | 0.248 | 0.229 | 0.082 | 0.041 | 0.117 | 0.044 | 0.130 | 0.074 |
| *LOC285830 ( HLA-F antisense RNA1)* | rs1633041 | T | 0.255 | 0.232 | 0.202 | 0.275 | 0.280 | 0.285 | 0.299 | 0.209 | 0.417 | 0.304 | 0.328 | 0.221 |
| *LOC285830 ( HLA-F antisense RNA1)* | rs1737031 | A | 0.280 | 0.258 | 0.207 | 0.286 | 0.313 | 0.332 | 0.350 | 0.262 | 0.451 | 0.333 | 0.401 | 0.289 |
| *LOC285830 ( HLA-F antisense RNA1)* | rs885940 | A | 0.255 | 0.232 | 0.202 | 0.275 | 0.280 | 0.285 | 0.301 | 0.209 | 0.417 | 0.314 | 0.328 | 0.221 |
| *LOC285830 ( HLA-F antisense RNA1)* | rs1610637 | C | 0.255 | 0.232 | 0.202 | 0.275 | 0.280 | 0.285 | 0.303 | 0.215 | 0.417 | 0.314 | 0.333 | 0.221 |
| *LOC285830 ( HLA-F antisense RNA1)* | rs1615251 | T | 0.690 | 0.712 | 0.783 | 0.681 | 0.650 | 0.631 | 0.626 | 0.727 | 0.524 | 0.623 | 0.573 | 0.696 |
| *HLA-G* | rs1633002 | A | 0.745 | 0.768 | 0.798 | 0.725 | 0.720 | 0.715 | 0.700 | 0.791 | 0.587 | 0.686 | 0.672 | 0.779 |
| *HLA-G* | rs1632973 | A | 0.254 | 0.232 | 0.202 | 0.269 | 0.280 | 0.285 | 0.302 | 0.209 | 0.417 | 0.314 | 0.333 | 0.221 |
| *HLA-G* | rs1736963 | T | 0.256 | 0.232 | 0.202 | 0.280 | 0.280 | 0.285 | 0.302 | 0.209 | 0.417 | 0.314 | 0.333 | 0.221 |
| *HLA-G* | rs2523408 | G | 0 | 0 | 0 | 0 | 0 | 0 | 0.005 | 0.017 | 0.005 | 0.005 | 0 | 0 |
| *HLA-G* | rs1611172 | G | 0.256 | 0.237 | 0.202 | 0.275 | 0.280 | 0.285 | 0.301 | 0.209 | 0.417 | 0.314 | 0.328 | 0.221 |
| *HLA-G* | rs753544 | T | 0.255 | 0.232 | 0.202 | 0.275 | 0.280 | 0.285 | 0.303 | 0.215 | 0.417 | 0.314 | 0.328 | 0.225 |
| *HLA-G* | rs1077433 | A | 0.255 | 0.232 | 0.202 | 0.275 | 0.280 | 0.285 | 0.302 | 0.209 | 0.417 | 0.314 | 0.333 | 0.221 |
| *HLA-G* | rs1736951 | A | 0.303 | 0.283 | 0.237 | 0.324 | 0.322 | 0.346 | 0.362 | 0.227 | 0.476 | 0.431 | 0.411 | 0.245 |
| *HLA-G* | rs407238 | C | 0.348 | 0.333 | 0.424 | 0.335 | 0.276 | 0.374 | 0.340 | 0.297 | 0.417 | 0.299 | 0.328 | 0.353 |
| *HCG9* | rs9260954 | G | 0.051 | 0.030 | 0.010 | 0.060 | 0.070 | 0.079 | 0.028 | 0.017 | 0.010 | 0.039 | 0.016 | 0.054 |
| *HCG9* | rs6911737 | A | 0.095 | 0.061 | 0.030 | 0.082 | 0.126 | 0.168 | 0.158 | 0.238 | 0.121 | 0.123 | 0.104 | 0.216 |
| *HCG9* | rs6926792 | A | 0.095 | 0.061 | 0.030 | 0.082 | 0.126 | 0.168 | 0.157 | 0.238 | 0.121 | 0.123 | 0.099 | 0.216 |
| *HCG9* | rs6931776 | G | 0.096 | 0.061 | 0.030 | 0.088 | 0.126 | 0.168 | 0.157 | 0.238 | 0.121 | 0.123 | 0.099 | 0.216 |
| *ZNRD1* | rs9261189 | T | 0.095 | 0.061 | 0.030 | 0.082 | 0.126 | 0.168 | 0.158 | 0.238 | 0.121 | 0.123 | 0.099 | 0.221 |
| *ZNRD1* | rs3869068 | A | 0.095 | 0.061 | 0.030 | 0.082 | 0.126 | 0.168 | 0.156 | 0.238 | 0.121 | 0.123 | 0.099 | 0.211 |
| *ZNRD1* | rs9261265 | C | 0.051 | 0.030 | 0.010 | 0.060 | 0.070 | 0.079 | 0.027 | 0.017 | 0.010 | 0.039 | 0.016 | 0.049 |
| *PPP1R11* | rs2074482 | T | 0.095 | 0.061 | 0.030 | 0.082 | 0.126 | 0.168 | 0.157 | 0.238 | 0.121 | 0.123 | 0.099 | 0.216 |
| *RNF39* | rs9261317 | A | 0.949 | 0.970 | 0.990 | 0.940 | 0.930 | 0.921 | 0.972 | 0.983 | 0.990 | 0.961 | 0.979 | 0.951 |
| *TRIM31* | rs9261376 | G | 0.189 | 0.146 | 0.061 | 0.187 | 0.248 | 0.290 | 0.346 | 0.372 | 0.398 | 0.333 | 0.302 | 0.324 |
| *TRIM31* | rs9261389 | G | 0.189 | 0.146 | 0.061 | 0.187 | 0.248 | 0.290 | 0.345 | 0.372 | 0.398 | 0.333 | 0.297 | 0.324 |
| *TRIM31* | rs6923832 | A | 0.051 | 0.030 | 0.010 | 0.060 | 0.070 | 0.079 | 0.027 | 0.017 | 0.010 | 0.039 | 0.016 | 0.049 |
| *MUC21* | rs2530710 | A | 0.182 | 0.212 | 0.232 | 0.176 | 0.150 | 0.145 | 0.237 | 0.209 | 0.199 | 0.240 | 0.255 | 0.279 |
| *MUC21* | rs2517446 | C | 0.143 | 0.101 | 0.086 | 0.121 | 0.224 | 0.173 | 0.125 | 0.145 | 0.073 | 0.152 | 0.130 | 0.127 |
| *MUC21* | rs2517411 | G | 0.146 | 0.101 | 0.096 | 0.121 | 0.224 | 0.178 | 0.127 | 0.151 | 0.078 | 0.152 | 0.130 | 0.127 |
| *MUC21* | rs2844673 | A | 0.146 | 0.101 | 0.096 | 0.121 | 0.224 | 0.178 | 0.127 | 0.151 | 0.078 | 0.152 | 0.130 | 0.127 |
| *MUC21* | rs2252925 | G | 0.146 | 0.101 | 0.096 | 0.121 | 0.224 | 0.178 | 0.127 | 0.151 | 0.078 | 0.152 | 0.130 | 0.127 |
| *MUC21* | rs2252926 | G | 0.146 | 0.101 | 0.096 | 0.121 | 0.224 | 0.178 | 0.127 | 0.151 | 0.078 | 0.152 | 0.130 | 0.127 |
| *MUC21* | rs1634717 | T | 0.405 | 0.354 | 0.293 | 0.423 | 0.477 | 0.467 | 0.232 | 0.227 | 0.180 | 0.289 | 0.266 | 0.201 |
| *MUC21* | rs2523915 | T | 0.854 | 0.899 | 0.904 | 0.879 | 0.776 | 0.822 | 0.873 | 0.849 | 0.922 | 0.848 | 0.870 | 0.873 |
| *MUC21* | rs1632854 | T | 0.595 | 0.646 | 0.707 | 0.577 | 0.523 | 0.533 | 0.768 | 0.773 | 0.820 | 0.711 | 0.734 | 0.799 |
| *C6orf15* | rs1265048 | A | 0.603 | 0.682 | 0.510 | 0.621 | 0.579 | 0.626 | 0.673 | 0.744 | 0.684 | 0.632 | 0.609 | 0.701 |
| *PSORS1C1* | rs4959053 | A | 0.082 | 0.086 | 0.076 | 0.082 | 0.079 | 0.084 | 0.178 | 0.221 | 0.146 | 0.157 | 0.250 | 0.127 |
| *CCHCR1* | rs2240063 | A | 0.437 | 0.439 | 0.359 | 0.423 | 0.495 | 0.463 | 0.474 | 0.535 | 0.466 | 0.451 | 0.458 | 0.471 |
| *CCHCR1* | rs2073716 | C | 0.957 | 0.965 | 0.955 | 0.962 | 0.939 | 0.967 | 0.922 | 0.953 | 0.942 | 0.931 | 0.901 | 0.887 |
| *TCF19* | rs2073723 | T | 0.216 | 0.247 | 0.232 | 0.253 | 0.173 | 0.182 | 0.317 | 0.407 | 0.243 | 0.284 | 0.339 | 0.328 |
| *POU5F1* | rs9501063 | G | 0.957 | 0.965 | 0.955 | 0.956 | 0.949 | 0.963 | 0.950 | 0.977 | 0.971 | 0.961 | 0.943 | 0.902 |
| *POU5F1* | rs9263804 | C | 0.219 | 0.247 | 0.232 | 0.258 | 0.182 | 0.182 | 0.344 | 0.424 | 0.272 | 0.314 | 0.380 | 0.343 |
| *POU5F1* | rs3130501 | A | 0.217 | 0.253 | 0.232 | 0.253 | 0.173 | 0.182 | 0.318 | 0.407 | 0.243 | 0.289 | 0.339 | 0.328 |
| *POU5F1* | rs3132524 | A | 0.220 | 0.247 | 0.232 | 0.258 | 0.182 | 0.187 | 0.344 | 0.424 | 0.272 | 0.314 | 0.380 | 0.343 |
| *HCG27* | rs3130944 | C | 0.763 | 0.727 | 0.808 | 0.731 | 0.794 | 0.752 | 0.767 | 0.826 | 0.733 | 0.735 | 0.760 | 0.789 |
| *HLA-C* | rs3905495 | C | 0.607 | 0.641 | 0.510 | 0.648 | 0.626 | 0.612 | 0.532 | 0.651 | 0.578 | 0.475 | 0.490 | 0.480 |
| *DHFRP2* | rs7761068 | T | 0.512 | 0.641 | 0.434 | 0.555 | 0.542 | 0.397 | 0.268 | 0.250 | 0.282 | 0.221 | 0.271 | 0.314 |
| *HLA-B* | rs9266406 | A | 0.215 | 0.182 | 0.202 | 0.148 | 0.224 | 0.304 | 0.412 | 0.483 | 0.393 | 0.436 | 0.375 | 0.382 |
| *HLA-B* | rs9266409 | C | 0.215 | 0.182 | 0.202 | 0.148 | 0.224 | 0.304 | 0.412 | 0.483 | 0.393 | 0.436 | 0.375 | 0.382 |
| *HLA-B* | rs6910516 | C | 0.215 | 0.182 | 0.202 | 0.148 | 0.224 | 0.304 | 0.412 | 0.483 | 0.393 | 0.436 | 0.375 | 0.382 |
| *MICA* | rs2523467 | A | 0.388 | 0.293 | 0.369 | 0.418 | 0.444 | 0.411 | 0.381 | 0.419 | 0.456 | 0.382 | 0.271 | 0.377 |
| *MICA* | rs3094584 | T | 0.179 | 0.121 | 0.051 | 0.132 | 0.318 | 0.252 | 0.261 | 0.302 | 0.223 | 0.275 | 0.255 | 0.255 |
| *BAG6(BAT3)* | rs2077102 | T | 0.163 | 0.146 | 0.328 | 0.165 | 0.093 | 0.093 | 0.115 | 0.110 | 0.107 | 0.093 | 0.099 | 0.162 |
| *C6orf47* | rs2242655 | C | 0.837 | 0.854 | 0.672 | 0.835 | 0.907 | 0.907 | 0.888 | 0.890 | 0.898 | 0.907 | 0.901 | 0.843 |
| *SLC44A4* | rs11965547 | A | 0.116 | 0.101 | 0.212 | 0.104 | 0.056 | 0.112 | 0.144 | 0.128 | 0.126 | 0.142 | 0.130 | 0.191 |
| *C6orf10* | rs544358 | C | 0.372 | 0.414 | 0.313 | 0.368 | 0.350 | 0.411 | 0.383 | 0.355 | 0.437 | 0.338 | 0.401 | 0.382 |
| *C6orf10* | rs574710 | G | 0.379 | 0.414 | 0.333 | 0.368 | 0.360 | 0.416 | 0.395 | 0.349 | 0.442 | 0.343 | 0.417 | 0.417 |
| *C6orf10* | rs539703 | C | 0.372 | 0.414 | 0.313 | 0.368 | 0.350 | 0.411 | 0.381 | 0.337 | 0.437 | 0.343 | 0.401 | 0.382 |
| *C6orf10* | rs926591 | T | 0.374 | 0.414 | 0.313 | 0.368 | 0.350 | 0.421 | 0.381 | 0.343 | 0.437 | 0.338 | 0.401 | 0.382 |
| *C6orf10* | rs4959093 | C | 0.374 | 0.414 | 0.313 | 0.368 | 0.350 | 0.421 | 0.381 | 0.343 | 0.437 | 0.338 | 0.401 | 0.382 |
| *BTNL2* | rs2076530 | G | 0.447 | 0.475 | 0.444 | 0.478 | 0.477 | 0.369 | 0.435 | 0.494 | 0.388 | 0.471 | 0.385 | 0.441 |
| *HLA-DQA1* | rs9272346 | G | 0.451 | 0.434 | 0.535 | 0.407 | 0.444 | 0.435 | 0.511 | 0.541 | 0.505 | 0.520 | 0.495 | 0.500 |
| *HLA-DQB1* | rs6457617 | C | 0.455 | 0.480 | 0.369 | 0.467 | 0.491 | 0.467 | 0.608 | 0.610 | 0.549 | 0.569 | 0.729 | 0.593 |
| *COL12A1* | rs4640857 | G | 0.414 | 0.404 | 0.465 | 0.379 | 0.421 | 0.397 | 0.505 | 0.547 | 0.500 | 0.554 | 0.505 | 0.426 |
| *C10orf11* | rs1323076 | G | 0.500 | 0.510 | 0.444 | 0.484 | 0.500 | 0.556 | 0.367 | 0.401 | 0.311 | 0.382 | 0.385 | 0.363 |
| *C10orf11* | rs17434565 | G | 0.214 | 0.192 | 0.298 | 0.181 | 0.206 | 0.192 | 0.265 | 0.331 | 0.199 | 0.270 | 0.271 | 0.265 |
| *PAX8* | rs11123169 | C | 0.329 | 0.374 | 0.359 | 0.253 | 0.322 | 0.332 | 0.349 | 0.401 | 0.383 | 0.353 | 0.323 | 0.289 |
| *PAX8* | rs10864912 | T | 0.456 | 0.470 | 0.510 | 0.456 | 0.416 | 0.435 | 0.420 | 0.436 | 0.456 | 0.387 | 0.422 | 0.402 |
| *HIVEP3* | rs4660590 | A | 0.472 | 0.444 | 0.535 | 0.533 | 0.411 | 0.449 | 0.481 | 0.401 | 0.408 | 0.554 | 0.495 | 0.534 |
| *CEP135* | rs2593082 | T | 0.508 | 0.455 | 0.515 | 0.484 | 0.547 | 0.533 | 0.476 | 0.477 | 0.471 | 0.436 | 0.464 | 0.534 |
| *CEP135* | rs2611826 | G | 0.490 | 0.545 | 0.485 | 0.516 | 0.444 | 0.467 | 0.527 | 0.535 | 0.544 | 0.574 | 0.531 | 0.451 |
| *HMP19* | rs1909704 | A | 0.670 | 0.702 | 0.636 | 0.665 | 0.645 | 0.701 | 0.501 | 0.471 | 0.500 | 0.510 | 0.495 | 0.525 |
| *TTLL7* | rs11163772 | A | 0.159 | 0.187 | 0.116 | 0.143 | 0.168 | 0.178 | 0.102 | 0.122 | 0.073 | 0.103 | 0.083 | 0.132 |
| *TENM4(ODZ4)* | rs2156215 | T | 0.033 | 0.005 | 0.086 | 0.027 | 0.033 | 0.014 | 0.237 | 0.262 | 0.214 | 0.304 | 0.203 | 0.206 |
| *KLRK1* | rs2617151 | A | 0.183 | 0.172 | 0.222 | 0.137 | 0.164 | 0.215 | 0.090 | 0.110 | 0.083 | 0.078 | 0.089 | 0.093 |
| *KLRK1* | rs2733852 | G | 0.294 | 0.293 | 0.308 | 0.264 | 0.280 | 0.322 | 0.150 | 0.157 | 0.150 | 0.127 | 0.161 | 0.157 |
| *OSR1* | rs4666492 | G | 0.418 | 0.404 | 0.470 | 0.385 | 0.402 | 0.430 | 0.385 | 0.343 | 0.388 | 0.392 | 0.396 | 0.402 |
| *CTNNA2* | rs4852547 | G | 0.484 | 0.455 | 0.525 | 0.495 | 0.467 | 0.481 | 0.333 | 0.390 | 0.272 | 0.333 | 0.359 | 0.324 |
| *MN1* | rs134006 | C | 0.058 | 0.040 | 0.076 | 0.055 | 0.042 | 0.075 | 0.185 | 0.186 | 0.155 | 0.250 | 0.161 | 0.172 |
| *LTN1(RNF160)* | rs2832137 | T | 0.562 | 0.571 | 0.611 | 0.632 | 0.486 | 0.523 | 0.538 | 0.523 | 0.539 | 0.549 | 0.557 | 0.520 |
| *HERPUD2* | rs11763983 | T | 0.571 | 0.601 | 0.636 | 0.588 | 0.533 | 0.505 | 0.259 | 0.262 | 0.296 | 0.245 | 0.240 | 0.250 |
| *GALNT10* | rs574750 | A | 0.293 | 0.308 | 0.258 | 0.302 | 0.304 | 0.294 | 0.379 | 0.419 | 0.403 | 0.314 | 0.401 | 0.368 |
| *SAMD3(TMEM200A)* | rs9483115 | T | 0.348 | 0.354 | 0.348 | 0.363 | 0.318 | 0.360 | 0.438 | 0.471 | 0.413 | 0.436 | 0.370 | 0.500 |
| *SAMD3(TMEM200A)* | rs4141940 | A | 0.343 | 0.343 | 0.348 | 0.357 | 0.313 | 0.355 | 0.435 | 0.471 | 0.413 | 0.431 | 0.370 | 0.490 |
| *SAMD3(TMEM200A)* | rs899276 | A | 0.343 | 0.343 | 0.348 | 0.357 | 0.313 | 0.355 | 0.438 | 0.471 | 0.413 | 0.436 | 0.370 | 0.500 |
| *SAMD3(TMEM200A)* | rs7758496 | G | 0.34 | 0.34 | 0.36 | 0.35 | 0.31 | 0.36 | 0.42 | 0.471 | 0.40 | 0.43 | 0.35 | 0.45 |
| *SAMD3(TMEM200A)* | rs724324 | G | 0.349 | 0.354 | 0.348 | 0.363 | 0.322 | 0.360 | 0.439 | 0.477 | 0.413 | 0.436 | 0.370 | 0.500 |
| *SAMD3* | rs4897380 | C | 0.36 | 0.37 | 0.38 | 0.36 | 0.33 | 0.37 | 0.44 | 0.471 | 0.42 | 0.49 | 0.37 | 0.45 |
| *SEMA6D* | rs470151 | T | 0.043 | 0.066 | 0.035 | 0.055 | 0.019 | 0.042 | 0.237 | 0.314 | 0.165 | 0.240 | 0.135 | 0.338 |
| *PMFBP1* | rs11862324 | T | 0.170 | 0.182 | 0.232 | 0.181 | 0.126 | 0.136 | 0.237 | 0.279 | 0.218 | 0.186 | 0.260 | 0.250 |
| *NAV2* | rs2707110 | C | 0.300 | 0.348 | 0.217 | 0.308 | 0.304 | 0.322 | 0.534 | 0.541 | 0.592 | 0.529 | 0.448 | 0.554 |
| *NAV2* | rs873764 | G | 0.430 | 0.470 | 0.460 | 0.462 | 0.360 | 0.411 | 0.601 | 0.599 | 0.655 | 0.613 | 0.526 | 0.608 |
| *TMEM132B* | rs4435061 | A | 0.286 | 0.263 | 0.328 | 0.308 | 0.257 | 0.280 | 0.317 | 0.337 | 0.345 | 0.289 | 0.297 | 0.319 |
| *TMEM132B* | rs10846917 | T | 0.669 | 0.662 | 0.631 | 0.621 | 0.720 | 0.701 | 0.562 | 0.541 | 0.544 | 0.588 | 0.620 | 0.520 |
| *TMEM132B* | rs10846924 | T | 0.230 | 0.227 | 0.288 | 0.264 | 0.192 | 0.187 | 0.279 | 0.314 | 0.296 | 0.260 | 0.240 | 0.289 |
| *STX8* | rs1549332 | A | 0.124 | 0.126 | 0.071 | 0.104 | 0.168 | 0.145 | 0.103 | 0.134 | 0.087 | 0.083 | 0.120 | 0.098 |
| *OVCH1* | rs1436321 | A | 0.308 | 0.283 | 0.374 | 0.286 | 0.290 | 0.308 | 0.505 | 0.552 | 0.529 | 0.451 | 0.505 | 0.495 |
| *SLC41A2* | rs2731031 | A | 0.372 | 0.374 | 0.455 | 0.352 | 0.350 | 0.332 | 0.603 | 0.541 | 0.607 | 0.603 | 0.589 | 0.667 |
| *HNF4G* | rs2980221 | A | 0.586 | 0.611 | 0.545 | 0.566 | 0.570 | 0.636 | 0.537 | 0.442 | 0.573 | 0.510 | 0.547 | 0.598 |
| *SMARCA2* | rs7033529 | A | 0.849 | 0.843 | 0.793 | 0.896 | 0.822 | 0.893 | 0.747 | 0.738 | 0.767 | 0.750 | 0.724 | 0.755 |
| *EBF2* | rs4570167 | C | 0.344 | 0.313 | 0.343 | 0.297 | 0.350 | 0.407 | 0.542 | 0.453 | 0.495 | 0.627 | 0.589 | 0.534 |
| *EBF2* | rs4242425 | T | 0.344 | 0.313 | 0.343 | 0.297 | 0.350 | 0.407 | 0.542 | 0.453 | 0.495 | 0.627 | 0.589 | 0.534 |
| *GAS2* | rs10833804 | G | 0.664 | 0.657 | 0.646 | 0.687 | 0.696 | 0.636 | 0.677 | 0.756 | 0.665 | 0.647 | 0.661 | 0.667 |
| *LYST/NID1* | rs7354999 | G | 0.989 | 1.000 | 0.960 | 0.995 | 0.995 | 0.995 | 0.731 | 0.680 | 0.791 | 0.667 | 0.776 | 0.735 |
| *LOC100132252* | rs9469615 | C | 0.143 | 0.126 | 0.136 | 0.143 | 0.117 | 0.192 | 0.057 | 0.041 | 0.068 | 0.039 | 0.094 | 0.044 |
| *LOC107984355* | rs872837 | A | 0.363 | 0.394 | 0.449 | 0.324 | 0.322 | 0.327 | 0.244 | 0.256 | 0.277 | 0.172 | 0.307 | 0.216 |
| *SACM1L* | rs1969624 | C | 0.564 | 0.520 | 0.540 | 0.516 | 0.584 | 0.645 | 0.319 | 0.250 | 0.291 | 0.363 | 0.391 | 0.294 |
| *PLEKHB1* | rs591804 | G | 0.354 | 0.354 | 0.318 | 0.352 | 0.355 | 0.388 | 0.383 | 0.331 | 0.403 | 0.392 | 0.396 | 0.387 |
| *ATP8A1* | rs2100766 | T | 0.034 | 0.025 | 0.061 | 0.060 | 0.009 | 0.019 | 0.093 | 0.081 | 0.107 | 0.083 | 0.094 | 0.098 |
| *KCNK9* | rs1961261 | A | 0.230 | 0.232 | 0.263 | 0.264 | 0.201 | 0.196 | 0.261 | 0.326 | 0.252 | 0.230 | 0.260 | 0.245 |
| *SUMO4* | rs237024 | C | 0.483 | 0.490 | 0.480 | 0.522 | 0.481 | 0.449 | 0.593 | 0.628 | 0.553 | 0.613 | 0.562 | 0.613 |

**Supplemental Table 4.** Population genetic parameter estimates of Behcet disease associated genes in the 1000 Genomes Asian populations

| **Gene** | **Total**  **Sites** | **Eta** | **Segregating**  **Sites** | **Haplotypes** | **Haplotype**  **Diversity** | **ThetaK** | **Pi** | **Theta**  **(Watterson)** | **Tajima’s**  **D** | **FuLi’s**  **D** | **FuLi’s**  **F** | **Achaz Y** | **R2** |
| --- | --- | --- | --- | --- | --- | --- | --- | --- | --- | --- | --- | --- | --- |
| *ABCB5* | 4810 | 1406 | 1404 | 959 | 1.00 | 108.64 | 0.0008 | 187.66 | -1.24 | -9.90 | -5.16 | -0.46 | 0.04 |
| *API5* | 788 | 204 | 203 | 163 | 0.92 | 7.17 | 0.0002 | 27.23 | -2.11 | -10.59 | -6.77 | -1.59 | 0.02 |
| *ASB18* | 1984 | 616 | 616 | 452 | 0.98 | 62.04 | 0.0009 | 82.22 | -0.72 | -9.44 | -4.78 | 0.36 | 0.05 |
| *ATP8A1* | 7037 | 1920 | 1915 | 902 | 1.00 | 176.94 | 0.0007 | 256.26 | -0.91 | -10.73 | -5.26 | 0.14 | 0.05 |
| *BAG6* | 382 | 144 | 143 | 157 | 0.95 | 10.73 | 0.0008 | 19.22 | -1.25 | -5.57 | -3.81 | -0.82 | 0.04 |
| *BTNL2* | 642 | 454 | 450 | 240 | 0.97 | 94.45 | 0.0077 | 60.59 | 1.63 | 1.91 | 2.01 | 1.43 | 0.10 |
| *C10ORF11 (LRMDA)* | 30401 | 8102 | 8093 | 1008 | 1.00 | 651.76 | 0.0006 | 1081.36 | -1.17 | -12.52 | -6.10 | -0.05 | 0.04 |
| *C6ORF10 (TSBP1)* | 2086 | 1083 | 1081 | 445 | 0.99 | 215.55 | 0.0027 | 144.55 | 1.44 | -0.19 | 0.93 | 1.57 | 0.10 |
| *C6ORF15* | 56 | 24 | 24 | 17 | 0.69 | 4.85 | 0.0038 | 3.20 | 1.23 | -2.13 | -0.84 | 2.08 | 0.10 |
| *C6ORF47* | 49 | 13 | 13 | 15 | 0.72 | 0.97 | 0.0004 | 1.74 | -0.94 | -2.57 | -2.34 | -0.45 | 0.04 |
| *C6ORF85 (SLC22A23)* | 5961 | 1763 | 1762 | 998 | 1.00 | 233.92 | 0.0012 | 235.30 | -0.02 | -9.54 | -4.13 | 1.34 | 0.07 |
| *CCDC180* | 2020 | 578 | 578 | 373 | 0.98 | 62.87 | 0.0009 | 77.14 | -0.54 | -10.11 | -4.98 | 0.78 | 0.05 |
| *CCHCR1* | 492 | 250 | 250 | 132 | 0.94 | 38.36 | 0.0024 | 33.37 | 0.43 | -1.46 | -0.44 | 0.67 | 0.08 |
| *CCR1* | 157 | 39 | 39 | 33 | 0.28 | 0.99 | 0.0002 | 5.21 | -2.09 | -5.83 | -4.98 | -1.74 | 0.01 |
| *CCR3* | 702 | 206 | 206 | 160 | 0.86 | 19.57 | 0.0008 | 27.49 | -0.83 | -7.92 | -4.56 | 0.15 | 0.05 |
| *CDH26* | 1524 | 399 | 399 | 440 | 0.98 | 18.42 | 0.0003 | 53.25 | -1.90 | -10.83 | -6.39 | -1.32 | 0.02 |
| *CEP135* | 2132 | 531 | 530 | 362 | 0.98 | 35.47 | 0.0004 | 70.87 | -1.46 | -10.60 | -5.86 | -0.64 | 0.03 |
| *COL12A1* | 3133 | 811 | 810 | 878 | 1.00 | 57.55 | 0.0005 | 108.24 | -1.37 | -12.75 | -6.63 | -0.22 | 0.03 |
| *CPLX1* | 1427 | 438 | 437 | 527 | 0.99 | 48.92 | 0.0012 | 58.46 | -0.48 | -8.87 | -4.46 | 0.71 | 0.06 |
| *CPVL* | 5291 | 1550 | 1545 | 988 | 1.00 | 154.73 | 0.0008 | 206.88 | -0.74 | -9.78 | -4.75 | 0.29 | 0.05 |
| *CTLA4* | 145 | 48 | 48 | 48 | 0.67 | 1.47 | 0.0002 | 6.41 | -2.03 | -7.68 | -6.03 | -1.43 | 0.02 |
| *CTNNA2* | 34316 | 9730 | 9718 | 1008 | 1.00 | 955.57 | 0.0008 | 1298.64 | -0.78 | -9.70 | -4.63 | 0.16 | 0.05 |
| *DEPDC1* | 532 | 145 | 145 | 103 | 0.74 | 11.13 | 0.0005 | 19.35 | -1.20 | -6.30 | -4.14 | -0.60 | 0.04 |
| *DHFRP2* | 150 | 110 | 109 | 35 | 0.81 | 22.25 | 0.0064 | 14.68 | 1.44 | 0.53 | 1.23 | 1.45 | 0.10 |
| *DNMT3A* | 2863 | 707 | 706 | 774 | 1.00 | 47.29 | 0.0004 | 94.36 | -1.46 | -13.07 | -6.88 | -0.31 | 0.03 |
| *DTL* | 1831 | 485 | 485 | 342 | 0.97 | 30.97 | 0.0004 | 64.73 | -1.52 | -9.65 | -5.50 | -0.85 | 0.03 |
| *EBF2* | 6081 | 1629 | 1625 | 953 | 1.00 | 135.43 | 0.0007 | 217.42 | -1.11 | -10.45 | -5.30 | -0.20 | 0.04 |
| *ERAP1* | 1453 | 435 | 435 | 317 | 0.97 | 57.39 | 0.0011 | 58.06 | -0.03 | -5.62 | -2.64 | 0.73 | 0.07 |
| *FUT2* | 356 | 103 | 103 | 116 | 0.88 | 4.26 | 0.0004 | 13.75 | -1.93 | -6.39 | -4.80 | -1.59 | 0.02 |
| *GABBR1* | 704 | 273 | 273 | 147 | 0.94 | 25.63 | 0.0008 | 36.44 | -0.86 | -4.06 | -2.56 | -0.51 | 0.05 |
| *GALNT1* | 2769 | 781 | 781 | 780 | 1.00 | 81.82 | 0.0009 | 104.24 | -0.63 | -9.17 | -4.53 | 0.42 | 0.05 |
| *GALT10* | 6235 | 1875 | 1873 | 971 | 1.00 | 162.96 | 0.0007 | 250.25 | -1.03 | -9.56 | -4.83 | -0.19 | 0.04 |
| *GAS2* | 4319 | 1159 | 1159 | 882 | 1.00 | 129.32 | 0.0009 | 154.69 | -0.48 | -9.70 | -4.59 | 0.70 | 0.06 |
| *GIMAP1* | 226 | 62 | 62 | 55 | 0.72 | 5.30 | 0.0007 | 8.28 | -0.97 | -7.21 | -4.97 | 0.27 | 0.04 |
| *GIMAP2* | 238 | 63 | 63 | 77 | 0.86 | 5.93 | 0.0007 | 8.41 | -0.80 | -4.90 | -3.45 | -0.05 | 0.05 |
| *GIMAP4* | 197 | 60 | 60 | 51 | 0.84 | 6.17 | 0.0009 | 8.01 | -0.62 | -6.15 | -4.14 | 0.56 | 0.05 |
| *HCG27* | 212 | 121 | 120 | 98 | 0.95 | 25.94 | 0.0043 | 16.15 | 1.71 | -1.45 | 0.32 | 2.21 | 0.11 |
| *HCG9* | 158 | 117 | 116 | 79 | 0.82 | 20.11 | 0.0062 | 15.62 | 0.81 | 0.70 | 0.87 | 0.69 | 0.09 |
| *HERPUD2* | 212 | 121 | 120 | 98 | 0.95 | 25.94 | 0.0043 | 16.15 | 1.71 | -1.45 | 0.32 | 2.21 | 0.11 |
| *HIVEP3* | 14415 | 3684 | 3679 | 1004 | 1.00 | 299.71 | 0.0006 | 491.70 | -1.15 | -11.35 | -5.63 | -0.17 | 0.04 |
| *HLA_B* | 297 | 270 | 248 | 690 | 0.99 | 54.89 | 0.0166 | 36.04 | 1.51 | -0.94 | 0.52 | 1.76 | 0.11 |
| *HLA_DQ* | 836 | 798 | 783 | 578 | 0.98 | 241.87 | 0.0347 | 106.51 | 3.72 | 4.08 | 4.43 | 3.13 | 0.15 |
| *HLA_F* | 128 | 52 | 51 | 33 | 0.81 | 8.47 | 0.0022 | 6.94 | 0.59 | -2.66 | -1.41 | 1.12 | 0.08 |
| *HLA_G* | 185 | 99 | 98 | 41 | 0.80 | 22.60 | 0.0055 | 13.21 | 1.98 | -1.66 | 0.34 | 2.63 | 0.12 |
| *HLA_C* | 319 | 233 | 222 | 532 | 0.98 | 55.02 | 0.0163 | 31.10 | 2.21 | -0.35 | 1.26 | 2.39 | 0.12 |
| *HLADQB1* | 905 | 861 | 861 | 646 | 0.99 | 278.42 | 0.0386 | 114.92 | 4.17 | 4.36 | 4.82 | 3.45 | 0.16 |
| *HMP19* | 1774 | 452 | 452 | 319 | 0.98 | 9.99 | 0.0002 | 60.33 | -2.43 | -10.29 | -6.44 | -2.27 | 0.01 |
| *HNF4G* | 808 | 234 | 234 | 154 | 0.88 | 25.21 | 0.0009 | 31.23 | -0.55 | -9.89 | -5.30 | 0.99 | 0.05 |
| *IFNG* | 118 | 28 | 28 | 28 | 0.62 | 1.45 | 0.0003 | 3.74 | -1.51 | -7.84 | -6.16 | 0.05 | 0.03 |
| *IL10* | 142 | 38 | 38 | 33 | 0.73 | 2.87 | 0.0006 | 5.07 | -1.12 | -5.98 | -4.54 | -0.08 | 0.04 |
| *IL12A* | 178 | 43 | 43 | 37 | 0.59 | 3.75 | 0.0005 | 5.74 | -0.90 | -5.29 | -3.92 | 0.02 | 0.04 |
| *IL17A* | 224 | 62 | 61 | 52 | 0.87 | 5.89 | 0.0008 | 8.28 | -0.78 | -6.48 | -4.44 | 0.35 | 0.05 |
| *IL1A* | 269 | 72 | 72 | 47 | 0.57 | 8.09 | 0.0007 | 9.61 | -0.43 | -6.45 | -4.10 | 0.84 | 0.06 |
| *IL23R* | 3296 | 853 | 852 | 680 | 1.00 | 74.90 | 0.0006 | 113.85 | -1.00 | -11.43 | -5.77 | 0.20 | 0.04 |
| *IL6* | 145 | 52 | 52 | 42 | 0.50 | 1.53 | 0.0003 | 6.94 | -2.07 | -4.63 | -4.10 | -1.88 | 0.01 |
| *KCNK9* | 3439 | 929 | 928 | 885 | 1.00 | 82.86 | 0.0008 | 123.99 | -0.97 | -12.62 | -6.26 | 0.46 | 0.04 |
| *KLRC4* | 473 | 144 | 144 | 119 | 0.93 | 17.22 | 0.0008 | 19.22 | -0.30 | -6.36 | -3.58 | 0.74 | 0.06 |
| *KLRK1* | 429 | 116 | 116 | 103 | 0.79 | 15.81 | 0.0009 | 15.48 | 0.06 | -6.76 | -3.69 | 1.46 | 0.07 |
| *LILRA1* | 461 | 171 | 171 | 149 | 0.91 | 8.89 | 0.0010 | 22.82 | -1.74 | -6.12 | -4.32 | -1.42 | 0.03 |
| *LILRB1* | 901 | 307 | 306 | 248 | 0.94 | 24.16 | 0.0012 | 40.97 | -1.19 | -6.14 | -3.76 | -0.70 | 0.04 |
| *LOC100129342* | 207 | 53 | 53 | 44 | 0.65 | 4.90 | 0.0007 | 7.07 | -0.82 | -5.58 | -3.96 | 0.14 | 0.05 |
| *LOC100132252* | 1039 | 385 | 384 | 428 | 0.99 | 38.60 | 0.0013 | 51.39 | -0.72 | -6.34 | -3.49 | -0.07 | 0.05 |
| *LOC107984355* | 617 | 179 | 179 | 309 | 0.98 | 11.38 | 0.0007 | 23.89 | -1.50 | -9.53 | -5.90 | -0.61 | 0.03 |
| *LOC285830* | 655 | 347 | 346 | 117 | 0.90 | 71.36 | 0.0032 | 46.31 | 1.57 | -1.06 | 0.55 | 1.85 | 0.10 |
| *LTN1* | 1567 | 430 | 430 | 337 | 0.98 | 33.70 | 0.0005 | 57.39 | -1.20 | -11.92 | -6.38 | 0.08 | 0.04 |
| *LYST* | 5588 | 1257 | 1257 | 828 | 1.00 | 38.18 | 0.0002 | 167.77 | -2.27 | -13.74 | -7.58 | -1.84 | 0.01 |
| *MEFV* | 584 | 169 | 169 | 259 | 0.96 | 18.87 | 0.0013 | 22.56 | -0.47 | -8.59 | -4.78 | 0.95 | 0.06 |
| *MICA* | 686 | 418 | 413 | 149 | 0.95 | 64.73 | 0.0042 | 55.79 | 0.47 | -3.67 | -1.40 | 1.05 | 0.08 |
| *MN1* | 1555 | 459 | 458 | 873 | 1.00 | 35.53 | 0.0007 | 61.26 | -1.22 | -8.90 | -4.97 | -0.47 | 0.04 |
| *MOG* | 427 | 185 | 183 | 88 | 0.86 | 9.16 | 0.0006 | 24.69 | -1.80 | -8.51 | -5.57 | -1.28 | 0.03 |
| *MSX2* | 199 | 51 | 51 | 43 | 0.65 | 1.94 | 0.0003 | 6.81 | -1.90 | -4.02 | -3.62 | -1.70 | 0.02 |
| *MUC21* | 209 | 114 | 112 | 164 | 0.86 | 8.35 | 0.0014 | 15.22 | -1.27 | -3.31 | -2.62 | -0.99 | 0.04 |
| *NAV2* | 24008 | 6560 | 6550 | 1008 | 1.00 | 664.45 | 0.0009 | 875.55 | -0.71 | -10.54 | -4.95 | 0.39 | 0.05 |
| *NOD2* | 1185 | 321 | 320 | 282 | 0.88 | 12.18 | 0.0003 | 42.84 | -2.07 | -11.73 | -7.04 | -1.50 | 0.02 |
| *OSR1* | 178 | 33 | 33 | 32 | 0.44 | 0.48 | 0.0001 | 4.40 | -2.25 | -9.61 | -7.66 | -1.33 | 0.01 |
| *OVCH1* | 1882 | 556 | 556 | 515 | 0.99 | 52.31 | 0.0007 | 74.21 | -0.86 | -8.17 | -4.32 | -0.05 | 0.05 |
| *PAX8* | 1700 | 507 | 506 | 448 | 0.98 | 50.28 | 0.0008 | 67.67 | -0.75 | -7.92 | -4.16 | 0.09 | 0.05 |
| *PLEKHB1* | 459 | 119 | 119 | 111 | 0.84 | 13.49 | 0.0008 | 15.88 | -0.42 | -5.74 | -3.42 | 0.47 | 0.06 |
| *PMFBP1* | 1461 | 401 | 401 | 340 | 0.98 | 29.81 | 0.0006 | 53.52 | -1.29 | -11.16 | -6.12 | -0.20 | 0.04 |
| *POU5F1* | 190 | 103 | 103 | 64 | 0.87 | 17.20 | 0.0027 | 13.75 | 0.70 | -2.77 | -1.12 | 1.31 | 0.08 |
| *PPP1R11* | 73 | 33 | 32 | 21 | 0.62 | 2.16 | 0.0007 | 4.40 | -1.29 | -1.38 | -1.65 | -1.24 | 0.03 |
| *PSMD14* | 2268 | 531 | 530 | 424 | 0.99 | 35.12 | 0.0003 | 70.87 | -1.47 | -13.68 | -7.28 | -0.16 | 0.03 |
| *PSORS1C1* | 920 | 567 | 561 | 272 | 0.97 | 101.02 | 0.0040 | 75.68 | 0.98 | -0.76 | 0.33 | 1.16 | 0.09 |
| *PTPN22* | 1249 | 313 | 313 | 243 | 0.91 | 24.69 | 0.0004 | 41.78 | -1.18 | -9.58 | -5.42 | -0.23 | 0,04 |
| *RALGAPA2* | 8018 | 1953 | 1950 | 958 | 1.00 | 113.86 | 0.0004 | 260.66 | -1.66 | -13.06 | -6.78 | -0.79 | 0.03 |
| *RIMBP2* | 11477 | 3395 | 3385 | 1008 | 1.00 | 412.77 | 0.0013 | 453.12 | -0.26 | -9.19 | -4.09 | 0.87 | 0.06 |
| *RNF39* | 173 | 89 | 89 | 49 | 0.84 | 10.65 | 0.0019 | 11.88 | -0.29 | -1.53 | -1.06 | -0.08 | 0.06 |
| *SACM1L* | 1481 | 447 | 446 | 275 | 0.94 | 64.48 | 0.0011 | 59.66 | 0.24 | -7.77 | -3.46 | 1.53 | 0.07 |
| *SAMD3* | 6653 | 2044 | 2037 | 962 | 1.00 | 308.60 | 0.0014 | 272.81 | 0.39 | -9.19 | -3.68 | 1.87 | 0.08 |
| *SEMA6D* | 15970 | 4670 | 4662 | 1003 | 1.00 | 471.63 | 0.0008 | 623.29 | -0.72 | -10.41 | -4.91 | 0.37 | 0.05 |
| *SGPP2* | 3802 | 1099 | 1096 | 974 | 1.00 | 80.10 | 0.0006 | 146.68 | -1.33 | -9.81 | -5.23 | -0.61 | 0.04 |
| *SLC41A2* | 3845 | 1111 | 1110 | 650 | 1.00 | 120.88 | 0.0008 | 148.28 | -0.54 | -10.17 | -4.84 | 0.69 | 0.05 |
| *SLC43A3* | 603 | 162 | 162 | 164 | 0.89 | 6.28 | 0.0003 | 21.62 | -2.02 | -10.16 | -6.64 | -1.43 | 0.02 |
| *SLC44A4* | 406 | 156 | 156 | 141 | 0.94 | 16.72 | 0.0011 | 20.82 | -0.56 | -4.38 | -2.67 | -0.02 | 0.05 |
| *SLIT2* | 10545 | 2845 | 2840 | 1004 | 1.00 | 205.09 | 0.0006 | 379.72 | -1.35 | -11.29 | -5.77 | -0.52 | 0.04 |
| *SMARCA2* | 6721 | 1809 | 1808 | 1001 | 1.00 | 177.36 | 0.0010 | 241.44 | -0.78 | -11.90 | -5.68 | 0.58 | 0.05 |
| *SMG6* | 6965 | 1975 | 1974 | 920 | 1.00 | 173.24 | 0.0007 | 263.60 | -1.01 | -12.11 | -5.91 | 0.22 | 0.04 |
| *SORBS2* | 11721 | 3518 | 3501 | 1008 | 1.00 | 434.77 | 0.0012 | 469.54 | -0.22 | -9.06 | -4.00 | 0.92 | 0.06 |
| *STAT4* | 3720 | 989 | 989 | 894 | 1.00 | 94.74 | 0.0007 | 132.00 | -0.83 | -11.46 | -5.63 | 0.48 | 0.05 |
| *STK39* | 8547 | 2341 | 2339 | 966 | 1.00 | 321.08 | 0.0011 | 312.45 | 0.08 | -9.04 | -3.82 | 1.37 | 0.07 |
| *STX8* | 10346 | 2820 | 2815 | 1008 | 1.00 | 217.81 | 0.0007 | 376.38 | -1.24 | -11.57 | -5.81 | -0.29 | 0.04 |
| *SUMO4* | 36 | 8 | 8 | 8 | 0.40 | 0.79 | 0.0010 | 1.07 | -0.48 | -2.98 | -2.49 | 0.46 | 0.05 |
| *SUSD1* | 3622 | 1048 | 1048 | 974 | 1.00 | 102.97 | 0.0008 | 139.87 | -0.77 | -10.86 | -5.31 | 0.45 | 0.05 |
| *TCF19* | 190 | 92 | 91 | 52 | 0.88 | 12.50 | 0.0016 | 12.28 | 0.05 | -3.47 | -1.99 | 0.63 | 0.07 |
| *TENM4* | 23487 | 6209 | 6203 | 1008 | 0.30 | 558.50 | 0.0007 | 828.70 | -0.96 | -11.78 | -5.65 | 0.20 | 0.04 |
| *TFCP2L1* | 2031 | 532 | 531 | 679 | 0.33 | 49.23 | 0.0007 | 71.00 | -0.89 | -10.39 | -5.38 | 0.28 | 0.05 |
| *TLR4* | 338 | 80 | 80 | 95 | 0.85 | 3.69 | 0.0003 | 10.68 | -1.80 | -9.49 | -6.65 | -0.85 | 0.02 |
| *TMEM132B* | 9972 | 2766 | 2764 | 1007 | 1.00 | 256.79 | 0.0008 | 369.17 | -0.90 | -10.43 | -5.08 | 0.11 | 0.05 |
| *TNF* | 61 | 13 | 13 | 13 | 0.33 | 0.47 | 0.0002 | 1.74 | -1.55 | -2.57 | -2.64 | -1.29 | 0.02 |
| *TNFAIP3* | 381 | 116 | 116 | 110 | 0.69 | 3.72 | 0.0002 | 15.48 | -2.13 | -8.63 | -6.11 | -1.73 | 0.02 |
| *TRIM31* | 338 | 146 | 146 | 166 | 0.95 | 14.92 | 0.0015 | 19.49 | -0.67 | -2.90 | -1.97 | -0.36 | 0.05 |
| *TTLL7* | 3060 | 874 | 873 | 605 | 0.99 | 56.74 | 0.0004 | 116.65 | -1.51 | -9.93 | -5.45 | -0.85 | 0.03 |
| *UBAC2* | 5101 | 1406 | 1405 | 808 | 1.00 | 110.54 | 0.0006 | 187.66 | -1.21 | -10.66 | -5.47 | -0.31 | 0.04 |
| *UBASH3B* | 4485 | 1324 | 1324 | 985 | 1.00 | 141.49 | 0.0009 | 176.71 | -0.59 | -8.72 | -4.21 | 0.36 | 0.05 |
| *UBD* | 120 | 48 | 48 | 30 | 0.79 | 6.17 | 0.0015 | 6.41 | -0.10 | -2.08 | -1.38 | 0.30 | 0.06 |
| *ZNRD1* | 85 | 53 | 53 | 38 | 0.81 | 4.43 | 0.0012 | 7.07 | -1.00 | -0.67 | -1.00 | -0.97 | 0.04 |

**Supplemental Table 5.** Population genetic parameter estimates of Behcet disease associated genes in the 1000 Genomes African populations

| **Gene** | **Total**  **Sites** | **Eta** | **Segregating**  **Sites** | **Haplotypes** | **Haplotype**  **Diversity** | **ThetaK** | **Pi** | **Theta**  **(Watterson)** | **Tajima’s**  **D** | **FuLi’s**  **D** | **FuLi’s**  **F** | **Achaz Y** | **R2** |
| --- | --- | --- | --- | --- | --- | --- | --- | --- | --- | --- | --- | --- | --- |
| *ABCB5* | 4810 | 2530 | 2527 | 1249 | 1.00 | 204.23 | 0.0014 | 325.87 | -1.09 | -7.03 | -3.64 | -0.65 | 0.04 |
| *API5* | 788 | 328 | 327 | 247 | 0.97 | 11.20 | 0.0003 | 42.25 | -2.10 | -8.57 | -5.45 | -1.87 | 0.02 |
| *ASB18* | 1984 | 1028 | 1025 | 697 | 1.00 | 104.25 | 0.0015 | 132.41 | -0.62 | -5.14 | -2.61 | -0.20 | 0.05 |
| *ATP8A1* | 7037 | 3530 | 3511 | 1143 | 1.00 | 238.82 | 0.0010 | 454.68 | -1.38 | -7.55 | -4.05 | -1.02 | 0.03 |
| *BAG6* | 382 | 180 | 180 | 206 | 0.96 | 11.07 | 0.0008 | 23.18 | -1.47 | -6.64 | -4.35 | -1.03 | 0.03 |
| *BTNL2* | 642 | 526 | 520 | 160 | 0.96 | 43.65 | 0.0035 | 67.75 | -1.03 | -4.52 | -2.73 | -0.76 | 0.04 |
| *C10ORF11 (LRMDA)* | 30401 | 14818 | 14778 | 1322 | 1.00 | 872.90 | 0.0008 | 1908.62 | -1.58 | -9.29 | -4.84 | -1.18 | 0.03 |
| *C6ORF10 (TSBP1)* | 2086 | 1267 | 1264 | 591 | 0.99 | 203.66 | 0.0026 | 163.19 | 0.72 | -2.76 | -0.64 | 1.10 | 0.08 |
| *C6ORF15* | 56 | 38 | 38 | 37 | 0.74 | 3.58 | 0.0028 | 4.89 | -0.68 | -2.70 | -2.15 | -0.27 | 0.05 |
| *C6ORF47* | 49 | 24 | 24 | 24 | 0.75 | 1.11 | 0.0005 | 3.09 | -1.52 | -3.39 | -3.16 | -1.18 | 0.02 |
| *C6ORF85 (SLC22A23)* | 5961 | 3108 | 3098 | 1285 | 1.00 | 277.29 | 0.0015 | 400.32 | -0.89 | -6.62 | -3.33 | -0.44 | 0.04 |
| *CCDC180* | 2020 | 907 | 905 | 481 | 0.99 | 67.38 | 0.0010 | 116.83 | -1.23 | -8.53 | -4.50 | -0.67 | 0.04 |
| *CCHCR1* | 492 | 293 | 293 | 228 | 0.97 | 43.68 | 0.0028 | 37.74 | 0.45 | -2.42 | -0.84 | 0.80 | 0.07 |
| *CCR1* | 157 | 74 | 74 | 69 | 0.76 | 2.61 | 0.0004 | 9.53 | -1.96 | -5.43 | -4.40 | -1.71 | 0.02 |
| *CCR3* | 702 | 366 | 366 | 285 | 0.95 | 24.89 | 0.0010 | 47.14 | -1.35 | -7.69 | -4.48 | -0.85 | 0.03 |
| *CDH26* | 1524 | 720 | 720 | 606 | 0.99 | 43.21 | 0.0008 | 92.74 | -1.54 | -8.99 | -4.97 | -1.07 | 0.03 |
| *CEP135* | 2132 | 1052 | 1051 | 567 | 0.99 | 46.61 | 0.0006 | 135.50 | -1.90 | -7.39 | -4.46 | -1.70 | 0.02 |
| *COL12A1* | 3133 | 1501 | 1495 | 1052 | 1.00 | 82.26 | 0.0007 | 193.33 | -1.67 | -9.44 | -5.11 | -1.25 | 0.03 |
| *CTLA4* | 145 | 60 | 60 | 56 | 0.82 | 2.44 | 0.0004 | 7.73 | -1.82 | -6.47 | -5.04 | -1.35 | 0.02 |
| *CPLX1* | 1427 | 712 | 711 | 683 | 1.00 | 59.49 | 0.0015 | 91.71 | -1.02 | -7.79 | -4.08 | -0.43 | 0.04 |
| *CPVL* | 5291 | 2705 | 2695 | 1232 | 1.00 | 199.51 | 0.0011 | 348.41 | -1.24 | -6.51 | -3.54 | -0.91 | 0.04 |
| *CTNNA2* | 34316 | 17541 | 17485 | 1322 | 1.00 | 1314.89 | 0.0012 | 2259.35 | -1.22 | -8.04 | -4.08 | -0.76 | 0.04 |
| *DEPDC1* | 532 | 253 | 253 | 199 | 0.94 | 20.03 | 0.0009 | 32.59 | -1.10 | -6.28 | -3.78 | -0.60 | 0.04 |
| *DHFRP2* | 150 | 126 | 125 | 69 | 0.83 | 23.84 | 0.0069 | 16.23 | 1.30 | -0.84 | 0.40 | 1.55 | 0.10 |
| *DNMT3A* | 2863 | 1278 | 1274 | 936 | 1.00 | 65.59 | 0.0006 | 164.61 | -1.75 | -9.60 | -5.25 | -1.34 | 0.03 |
| *DTL* | 1831 | 921 | 920 | 486 | 0.99 | 44.44 | 0.0006 | 118.63 | -1.81 | -7.66 | -4.54 | -1.56 | 0.02 |
| *EBF2* | 6081 | 3026 | 3007 | 1187 | 1.00 | 197.84 | 0.0010 | 389.76 | -1.43 | -8.20 | -4.36 | -1.04 | 0.03 |
| *ERAP1* | 1453 | 762 | 760 | 538 | 0.99 | 66.40 | 0.0012 | 98.15 | -0.94 | -6.15 | -3.31 | -0.49 | 0.04 |
| *FUT2* | 356 | 178 | 178 | 227 | 0.93 | 19.94 | 0.0020 | 22.93 | -0.37 | -6.09 | -3.34 | 0.44 | 0.06 |
| *GABBR1* | 704 | 371 | 370 | 228 | 0.96 | 19.66 | 0.0006 | 47.79 | -1.69 | -4.81 | -3.38 | -1.52 | 0.03 |
| *GALNT1* | 2769 | 1380 | 1380 | 1027 | 1.00 | 101.99 | 0.0011 | 177.75 | -1.24 | -7.29 | -3.92 | -0.82 | 0.04 |
| *GALT10* | 6235 | 3215 | 3206 | 1189 | 1.00 | 292.02 | 0.0013 | 414.10 | -0.86 | -6.92 | -3.42 | -0.37 | 0.05 |
| *GAS2* | 4319 | 2263 | 2253 | 1052 | 1.00 | 192.97 | 0.0013 | 291.48 | -0.98 | -6.70 | -3.44 | -0.54 | 0.04 |
| *GIMAP1* | 226 | 114 | 113 | 113 | 0.95 | 6.67 | 0.0009 | 14.68 | -1.51 | -2.80 | -2.49 | -1.36 | 0.03 |
| *GIMAP2* | 238 | 132 | 132 | 187 | 0.95 | 6.45 | 0.0008 | 17.00 | -1.73 | -6.56 | -4.63 | -1.36 | 0.02 |
| *GIMAP4* | 197 | 86 | 86 | 82 | 0.89 | 7.37 | 0.0011 | 11.08 | -0.91 | -2.91 | -2.24 | -0.63 | 0.04 |
| *HCG27* | 212 | 143 | 143 | 130 | 0.95 | 22.84 | 0.0038 | 18.42 | 0.67 | -0.48 | 0.18 | 0.78 | 0.08 |
| *HCG9* | 158 | 127 | 124 | 186 | 0.96 | 25.05 | 0.0077 | 16.36 | 1.48 | 0.55 | 1.26 | 1.46 | 0.10 |
| *HERPUD2* | 212 | 143 | 143 | 130 | 0.95 | 22.84 | 0.0038 | 18.42 | 0.67 | -0.48 | 0.18 | 0.78 | 0.08 |
| *HIVEP3* | 14415 | 7323 | 7298 | 1312 | 1.00 | 519.53 | 0.0010 | 943.23 | -1.31 | -8.02 | -4.16 | -0.89 | 0.04 |
| *HLA_B* | 297 | 282 | 261 | 975 | 1.00 | 50.66 | 0.0153 | 36.32 | 1.13 | 2.56 | 1.93 | 0.72 | 0.10 |
| *HLA_DQ* | 836 | 799 | 784 | 761 | 0.97 | 236.97 | 0.0340 | 102.91 | 3.77 | 3.96 | 4.37 | 3.23 | 0.15 |
| *HLA_F* | 128 | 74 | 73 | 58 | 0.91 | 10.41 | 0.0027 | 9.53 | 0.25 | -5.00 | -2.88 | 1.18 | 0.07 |
| *HLA_G* | 185 | 125 | 124 | 69 | 0.90 | 25.63 | 0.0062 | 16.10 | 1.64 | -1.91 | 0.04 | 2.17 | 0.10 |
| *HLAC* | 319 | 284 | 270 | 805 | 1.00 | 63.54 | 0.0188 | 36.58 | 2.10 | 1.95 | 2.36 | 1.86 | 0.12 |
| *HLADQB1* | 905 | 866 | 865 | 794 | 0.98 | 244.95 | 0.0340 | 111.54 | 3.46 | -0.10 | 2.39 | 3.66 | 0.14 |
| *HMP19* | 1774 | 842 | 838 | 483 | 0.99 | 49.72 | 0.0008 | 108.45 | -1.57 | -8.50 | -4.75 | -1.16 | 0.03 |
| *HNF4G* | 808 | 421 | 419 | 301 | 0.97 | 23.39 | 0.0009 | 54.23 | -1.63 | -7.01 | -4.33 | -1.34 | 0.03 |
| *IFNG* | 118 | 59 | 59 | 51 | 0.85 | 2.26 | 0.0005 | 7.60 | -1.86 | -4.54 | -3.89 | -1.62 | 0.02 |
| *IL10* | 142 | 58 | 58 | 59 | 0.85 | 4.59 | 0.0010 | 7.47 | -1.02 | -5.32 | -3.88 | -0.32 | 0.04 |
| *IL12A* | 178 | 82 | 82 | 75 | 0.90 | 5.98 | 0.0009 | 10.56 | -1.18 | -4.79 | -3.51 | -0.71 | 0.04 |
| *IL17A* | 224 | 110 | 110 | 102 | 0.93 | 6.17 | 0.0008 | 14.17 | -1.56 | -6.51 | -4.58 | -1.09 | 0.03 |
| *IL1A* | 269 | 139 | 139 | 108 | 0.94 | 10.77 | 0.0009 | 17.90 | -1.11 | -4.62 | -3.17 | -0.73 | 0.04 |
| *IL23R* | 3296 | 1664 | 1659 | 806 | 1.00 | 118.55 | 0.0010 | 214.33 | -1.30 | -7.46 | -4.01 | -0.89 | 0.04 |
| *IL6* | 145 | 66 | 65 | 77 | 0.92 | 4.51 | 0.0010 | 8.50 | -1.26 | -3.47 | -2.83 | -0.91 | 0.03 |
| *KCNK9* | 3439 | 1758 | 1751 | 1214 | 1.00 | 124.64 | 0.0012 | 226.44 | -1.31 | -7.11 | -3.87 | -0.95 | 0.04 |
| *KLRC4* | 473 | 240 | 240 | 160 | 0.95 | 24.78 | 0.0012 | 30.91 | -0.56 | -5.74 | -3.18 | 0.06 | 0.05 |
| *KLRK1* | 429 | 199 | 199 | 155 | 0.95 | 26.10 | 0.0015 | 25.63 | 0.05 | -4.32 | -2.13 | 0.66 | 0.07 |
| *LILRA1* | 461 | 239 | 239 | 240 | 0.97 | 18.64 | 0.0021 | 30.78 | -1.12 | -5.65 | -3.51 | -0.70 | 0.04 |
| *LILRB1* | 901 | 508 | 505 | 638 | 0.99 | 43.66 | 0.0021 | 65.43 | -0.96 | -5.15 | -2.95 | -0.58 | 0.04 |
| *LOC100129342* | 207 | 97 | 97 | 79 | 0.92 | 3.99 | 0.0006 | 12.49 | -1.87 | -5.04 | -4.02 | -1.65 | 0.02 |
| *LOC100132252* | 1039 | 574 | 570 | 560 | 0.99 | 53.55 | 0.0018 | 73.93 | -0.80 | -3.94 | -2.29 | -0.51 | 0.05 |
| *LOC107984355* | 617 | 276 | 274 | 642 | 0.99 | 17.80 | 0.0010 | 35.55 | -1.42 | -8.77 | -5.20 | -0.88 | 0.03 |
| *LOC285830* | 655 | 443 | 442 | 257 | 0.98 | 77.78 | 0.0035 | 57.06 | 1.04 | -3.19 | -0.72 | 1.58 | 0.09 |
| *LTN1* | 1567 | 743 | 742 | 471 | 0.99 | 40.87 | 0.0006 | 95.70 | -1.66 | -9.26 | -5.17 | -1.22 | 0.03 |
| *LYST* | 5588 | 2713 | 2702 | 1066 | 1.00 | 183.39 | 0.0008 | 349.45 | -1.38 | -8.61 | -4.49 | -0.93 | 0.03 |
| *MEFV* | 584 | 278 | 277 | 481 | 0.99 | 22.98 | 0.0016 | 35.81 | -1.02 | -5.18 | -3.16 | -0.61 | 0.04 |
| *MICA* | 686 | 533 | 526 | 260 | 0.98 | 67.66 | 0.0044 | 68.65 | -0.04 | 0.26 | 0.09 | -0.07 | 0.06 |
| *MN1* | 1555 | 743 | 739 | 1062 | 1.00 | 40.16 | 0.0008 | 95.70 | -1.68 | -7.98 | -4.63 | -1.36 | 0.03 |
| *MOG* | 427 | 259 | 257 | 169 | 0.96 | 23.05 | 0.0015 | 33.36 | -0.88 | -6.94 | -3.95 | -0.22 | 0.04 |
| *MSX2* | 199 | 94 | 94 | 104 | 0.94 | 8.05 | 0.0013 | 12.11 | -0.92 | -5.00 | -3.40 | -0.36 | 0.04 |
| *MUC21* | 209 | 146 | 141 | 233 | 0.96 | 9.74 | 0.0016 | 18.81 | -1.35 | -0.68 | -1.26 | -1.41 | 0.03 |
| *NAV2* | 24008 | 12463 | 12414 | 1322 | 1.00 | 988.67 | 0.0013 | 1605.28 | -1.12 | -7.20 | -3.68 | -0.70 | 0.04 |
| *NOD2* | 1185 | 530 | 529 | 420 | 0.99 | 26.67 | 0.0007 | 68.27 | -1.76 | -8.55 | -5.03 | -1.40 | 0.02 |
| *OSR1* | 178 | 88 | 88 | 103 | 0.79 | 2.87 | 0.0004 | 11.33 | -2.04 | -7.26 | -5.43 | -1.69 | 0.02 |
| *OVCH1* | 1882 | 953 | 950 | 692 | 0.99 | 80.03 | 0.0011 | 122.75 | -1.01 | -6.88 | -3.64 | -0.54 | 0.04 |
| *PAX8* | 1700 | 850 | 848 | 606 | 0.99 | 66.97 | 0.0011 | 109.48 | -1.12 | -7.23 | -3.89 | -0.66 | 0.04 |
| *PLEKHB1* | 459 | 256 | 254 | 251 | 0.96 | 24.15 | 0.0015 | 32.97 | -0.76 | -5.46 | -3.14 | -0.22 | 0.05 |
| *PMFBP1* | 1461 | 635 | 633 | 486 | 0.99 | 37.36 | 0.0007 | 81.79 | -1.57 | -10.36 | -5.64 | -1.01 | 0.03 |
| *POU5F1* | 190 | 107 | 107 | 67 | 0.92 | 14.46 | 0.0023 | 13.78 | 0.13 | -2.35 | -1.21 | 0.51 | 0.07 |
| *PPP1R11* | 73 | 35 | 33 | 30 | 0.85 | 3.02 | 0.0010 | 4.51 | -0.83 | -2.49 | -2.18 | -0.58 | 0.05 |
| *PSMD14* | 2268 | 998 | 996 | 608 | 0.99 | 30.15 | 0.0003 | 128.55 | -2.22 | -9.73 | -5.69 | -2.03 | 0.01 |
| *PSORS1C1* | 920 | 582 | 577 | 366 | 0.97 | 94.74 | 0.0038 | 74.96 | 0.76 | -3.23 | -0.90 | 1.24 | 0.08 |
| *PTPN22* | 1249 | 541 | 541 | 375 | 0.97 | 22.73 | 0.0004 | 69.68 | -1.94 | -8.07 | -4.93 | -1.69 | 0.02 |
| *RALGAPA2* | 8018 | 3639 | 3634 | 1224 | 1.00 | 137.17 | 0.0004 | 468.72 | -2.06 | -9.72 | -5.41 | -1.83 | 0.02 |
| *RIMBP2* | 11477 | 6072 | 6045 | 1322 | 1.00 | 506.77 | 0.0016 | 782.10 | -1.03 | -6.49 | -3.34 | -0.63 | 0.04 |
| *RNF39* | 173 | 94 | 94 | 70 | 0.92 | 12.41 | 0.0022 | 12.11 | 0.07 | -2.48 | -1.36 | 0.47 | 0.07 |
| *SACM1L* | 1481 | 749 | 749 | 441 | 0.99 | 77.14 | 0.0014 | 96.47 | -0.58 | -6.50 | -3.21 | 0.01 | 0.05 |
| *SAMD3* | 6653 | 3684 | 3669 | 1205 | 1.00 | 319.27 | 0.0014 | 474.51 | -0.95 | -5.16 | -2.78 | -0.65 | 0.04 |
| *SEMA6D* | 15970 | 8188 | 8160 | 1317 | 1.00 | 556.11 | 0.0009 | 1054.65 | -1.38 | -9.12 | -4.65 | -0.90 | 0.03 |
| *SGPP2* | 3802 | 1907 | 1903 | 1165 | 1.00 | 133.19 | 0.0010 | 245.63 | -1.33 | -8.36 | -4.40 | -0.88 | 0.03 |
| *SLC41A2* | 3845 | 1894 | 1891 | 817 | 1.00 | 124.14 | 0.0008 | 243.95 | -1.43 | -6.37 | -3.64 | -1.15 | 0.03 |
| *SLC43A3* | 603 | 264 | 264 | 260 | 0.98 | 15.57 | 0.0008 | 34.00 | -1.54 | -6.38 | -4.11 | -1.21 | 0.03 |
| *SLC44A4* | 406 | 211 | 211 | 217 | 0.97 | 18.01 | 0.0011 | 27.18 | -0.96 | -5.18 | -3.21 | -0.52 | 0.04 |
| *SLIT2* | 10545 | 5088 | 5069 | 1298 | 1.00 | 278.11 | 0.0008 | 655.35 | -1.68 | -9.28 | -4.94 | -1.31 | 0.03 |
| *SMARCA2* | 6721 | 3437 | 3427 | 1299 | 1.00 | 243.11 | 0.0014 | 442.70 | -1.31 | -8.31 | -4.31 | -0.86 | 0.04 |
| *SMG6* | 6965 | 3153 | 3142 | 1159 | 1.00 | 225.72 | 0.0009 | 406.12 | -1.29 | -10.61 | -5.23 | -0.61 | 0.04 |
| *SORBS2* | 11721 | 6134 | 6122 | 1321 | 1.00 | 561.34 | 0.0015 | 790.08 | -0.84 | -6.80 | -3.34 | -0.36 | 0.05 |
| *STAT4* | 3720 | 1762 | 1757 | 1057 | 1.00 | 116.54 | 0.0008 | 226.95 | -1.41 | -8.69 | -4.59 | -0.96 | 0.03 |
| *STK39* | 8547 | 4545 | 4536 | 1218 | 1.00 | 405.22 | 0.0014 | 585.41 | -0.90 | -7.29 | -3.59 | -0.39 | 0.04 |
| *STX8* | 10346 | 5399 | 5381 | 1319 | 1.00 | 437.06 | 0.0013 | 695.41 | -1.08 | -7.72 | -3.88 | -0.60 | 0.04 |
| *SUMO4* | 36 | 15 | 15 | 14 | 0.54 | 0.69 | 0.0009 | 1.93 | -1.39 | -0.05 | -0.71 | -1.45 | 0.02 |
| *SUSD1* | 3622 | 1798 | 1794 | 1195 | 1.00 | 120.52 | 0.0009 | 231.59 | -1.39 | -8.23 | -4.38 | -0.96 | 0.03 |
| *TCF19* | 190 | 100 | 99 | 69 | 0.90 | 11.74 | 0.0015 | 12.88 | -0.24 | -4.24 | -2.54 | 0.40 | 0.06 |
| *TENM4* | 23487 | 11678 | 11642 | 1320 | 1.00 | 838.79 | 0.0011 | 1504.17 | -1.29 | -8.38 | -4.27 | -0.83 | 0.04 |
| *TFCP2L1* | 2031 | 955 | 953 | 862 | 1.00 | 66.90 | 0.0010 | 123.01 | -1.32 | -8.25 | -4.44 | -0.83 | 0.03 |
| *TLR4* | 338 | 167 | 167 | 211 | 0.97 | 9.55 | 0.0007 | 21.51 | -1.56 | -5.62 | -3.92 | -1.25 | 0.03 |
| *TMEM132B* | 9972 | 5241 | 5227 | 1305 | 1.00 | 370.74 | 0.0011 | 675.06 | -1.31 | -7.77 | -4.07 | -0.91 | 0.04 |
| *TNF* | 61 | 27 | 27 | 28 | 0.42 | 0.75 | 0.0003 | 3.48 | -1.89 | -6.20 | -5.27 | -1.31 | 0.01 |
| *TNFAIP3* | 381 | 167 | 165 | 179 | 0.93 | 12.19 | 0.0008 | 21.51 | -1.22 | -7.52 | -4.66 | -0.48 | 0.04 |
| *TRIM31* | 338 | 191 | 191 | 275 | 0.98 | 20.99 | 0.0021 | 24.60 | -0.41 | -2.49 | -1.53 | -0.16 | 0.05 |
| *TTLL7* | 3060 | 1380 | 1379 | 748 | 1.00 | 77.70 | 0.0006 | 177.75 | -1.63 | -8.33 | -4.63 | -1.28 | 0.03 |
| *UBAC2* | 5101 | 2372 | 2367 | 960 | 1.00 | 110.87 | 0.0006 | 305.52 | -1.85 | -9.22 | -5.10 | -1.56 | 0.02 |
| *UBASH3B* | 4485 | 2372 | 2363 | 1215 | 1.00 | 182.56 | 0.0012 | 305.52 | -1.17 | -6.95 | -3.67 | -0.77 | 0.04 |
| *UBD* | 120 | 67 | 66 | 53 | 0.81 | 9.70 | 0.0023 | 8.63 | 0.33 | -3.39 | -1.79 | 1.16 | 0.07 |
| *ZNRD1* | 85 | 39 | 39 | 32 | 0.80 | 5.38 | 0.0015 | 5.02 | 0.18 | -0.86 | -0.46 | 0.36 | 0.07 |

**Supplemental Table 6.** Population genetic parameter estimates of Behcet disease associated genes in the 1000 Genomes European populations

| **Gene** | **Total**  **Sites** | **Eta** | **Segregating**  **Sites** | **Haplotypes** | **Haplotype**  **Diversity** | **ThetaK** | **Pi** | **Theta**  **(Watterson)** | **Tajima’s**  **D** | **FuLi’s**  **D** | **FuLi’s**  **F** | **Achaz Y** | **R2** |
| --- | --- | --- | --- | --- | --- | --- | --- | --- | --- | --- | --- | --- | --- |
| *ABCB5* | 4810 | 1331 | 1330 | 937 | 1.00 | 154.70 | 0.0011 | 177.69 | -0.38 | -9.64 | -4.47 | 0.83 | 0.06 |
| *API5* | 788 | 197 | 196 | 159 | 0.93 | 11.00 | 0.0003 | 26.30 | -1.67 | -10.45 | -6.44 | -0.81 | 0.03 |
| *ASB18* | 1984 | 674 | 674 | 443 | 0.98 | 67.32 | 0.0010 | 89.98 | -0.74 | -8.56 | -4.37 | 0.17 | 0.05 |
| *ATP8A1* | 7037 | 1794 | 1790 | 842 | 1.00 | 224.55 | 0.0009 | 239.50 | -0.18 | -8.66 | -3.87 | 0.91 | 0.06 |
| *BAG6* | 382 | 132 | 131 | 123 | 0.96 | 10.40 | 0.0007 | 17.62 | -1.16 | -7.78 | -4.94 | -0.21 | 0.04 |
| *BTNL2* | 642 | 438 | 434 | 98 | 0.94 | 38.91 | 0.0032 | 58.47 | -0.97 | 2.77 | 0.61 | -1.24 | 0.04 |
| *C10ORF11 (LRMDA)* | 30401 | 7855 | 7846 | 1005 | 1.00 | 618.55 | 0.0005 | 1048.67 | -1.21 | -11.28 | -5.61 | -0.30 | 0.04 |
| *C6ORF10 (TSBP1)* | 2086 | 1171 | 1166 | 432 | 0.98 | 214.66 | 0.0027 | 156.33 | 1.10 | -0.44 | 0.58 | 1.23 | 0.09 |
| *C6ORF15* | 56 | 27 | 27 | 25 | 0.73 | 4.58 | 0.0036 | 3.60 | 0.66 | -1.25 | -0.51 | 1.05 | 0.08 |
| *C6ORF47* | 49 | 15 | 15 | 15 | 0.68 | 0.87 | 0.0004 | 2.00 | -1.24 | -3.63 | -3.26 | -0.62 | 0.03 |
| *C6ORF85 (SLC22A23)* | 5961 | 1749 | 1747 | 965 | 1.00 | 210.03 | 0.0011 | 233.50 | -0.30 | -7.99 | -3.66 | 0.65 | 0.06 |
| *CCDC180* | 2020 | 526 | 525 | 329 | 0.98 | 50.52 | 0.0007 | 70.22 | -0.82 | -9.19 | -4.78 | 0.19 | 0.05 |
| *CCHCR1* | 492 | 255 | 254 | 146 | 0.96 | 45.27 | 0.0029 | 34.04 | 0.95 | -1.40 | -0.05 | 1.25 | 0.09 |
| *CCR1* | 157 | 48 | 48 | 43 | 0.47 | 2.10 | 0.0003 | 6.41 | -1.77 | -8.05 | -6.12 | -0.85 | 0.02 |
| *CCR3* | 702 | 178 | 178 | 116 | 0.93 | 23.77 | 0.0010 | 23.76 | 0.00 | -5.94 | -3.08 | 1.00 | 0.07 |
| *CDH26* | 1524 | 442 | 440 | 380 | 0.99 | 15.04 | 0.0003 | 59.01 | -2.17 | -10.92 | -6.57 | -1.79 | 0.02 |
| *CEP135* | 2132 | 536 | 536 | 348 | 0.98 | 42.06 | 0.0005 | 71.56 | -1.20 | -10.11 | -5.46 | -0.28 | 0.04 |
| *COL12A1* | 3133 | 786 | 785 | 818 | 1.00 | 28.39 | 0.0002 | 104.93 | -2.14 | -11.40 | -6.57 | -1.74 | 0.02 |
| *CTLA4* | 145 | 26 | 26 | 24 | 0.66 | 1.37 | 0.0002 | 3.47 | -1.48 | -6.18 | -5.04 | -0.47 | 0.03 |
| *CPLX1* | 1427 | 426 | 425 | 522 | 1.00 | 55.09 | 0.0013 | 56.87 | -0.09 | -7.04 | -3.36 | 0.90 | 0.06 |
| *CPVL* | 5291 | 1639 | 1634 | 950 | 1.00 | 177.83 | 0.0009 | 218.81 | -0.55 | -7.94 | -3.82 | 0.28 | 0.05 |
| *CTNNA2* | 34316 | 9396 | 9386 | 1006 | 1.00 | 861.27 | 0.0008 | 1254.40 | -0.92 | -10.11 | -4.91 | -0.01 | 0.05 |
| *DEPDC1* | 532 | 140 | 140 | 98 | 0.83 | 14.99 | 0.0007 | 18.69 | -0.56 | -7.31 | -4.28 | 0.57 | 0.05 |
| *DHFRP2* | 150 | 92 | 92 | 35 | 0.90 | 24.58 | 0.0071 | 12.28 | 2.78 | 1.05 | 2.32 | 2.64 | 0.13 |
| *DNMT3A* | 2863 | 726 | 725 | 695 | 1.00 | 57.53 | 0.0005 | 96.92 | -1.19 | -10.87 | -5.70 | -0.18 | 0.04 |
| *DTL* | 1831 | 504 | 504 | 302 | 0.97 | 38.64 | 0.0006 | 67.29 | -1.24 | -7.87 | -4.48 | -0.64 | 0.04 |
| *EBF2* | 6081 | 1738 | 1727 | 878 | 1.00 | 170.09 | 0.0008 | 232.03 | -0.79 | -9.23 | -4.54 | 0.12 | 0.05 |
| *ERAP1* | 1453 | 517 | 514 | 371 | 0.99 | 77.47 | 0.0015 | 69.02 | 0.36 | -6.06 | -2.53 | 1.33 | 0.07 |
| *FUT2* | 356 | 103 | 103 | 126 | 0.85 | 15.32 | 0.0015 | 13.75 | 0.32 | -5.93 | -3.13 | 1.65 | 0.07 |
| *GABBR1* | 704 | 305 | 304 | 151 | 0.91 | 18.32 | 0.0006 | 40.72 | -1.59 | -7.39 | -4.64 | -1.13 | 0.03 |
| *GALNT1* | 2769 | 754 | 754 | 714 | 1.00 | 89.96 | 0.0010 | 100.66 | -0.31 | -8.15 | -3.86 | 0.72 | 0.06 |
| *GALT10* | 6235 | 1806 | 1803 | 929 | 1.00 | 247.65 | 0.0011 | 241.11 | 0.08 | -8.27 | -3.51 | 1.24 | 0.07 |
| *GAS2* | 4319 | 1189 | 1186 | 831 | 1.00 | 127.31 | 0.0009 | 158.74 | -0.58 | -10.62 | -5.06 | 0.70 | 0.05 |
| *GIMAP1* | 226 | 48 | 48 | 45 | 0.72 | 4.58 | 0.0006 | 6.41 | -0.75 | -4.69 | -3.41 | 0.05 | 0.05 |
| *GIMAP2* | 238 | 53 | 53 | 54 | 0.81 | 5.78 | 0.0007 | 7.08 | -0.49 | -4.53 | -3.11 | 0.36 | 0.05 |
| *GIMAP4* | 197 | 52 | 52 | 41 | 0.81 | 5.15 | 0.0008 | 6.94 | -0.69 | -5.34 | -3.74 | 0.29 | 0.05 |
| *HCG27* | 212 | 122 | 121 | 115 | 0.95 | 24.85 | 0.0041 | 16.29 | 1.48 | -1.01 | 0.40 | 1.80 | 0.10 |
| *HCG9* | 158 | 115 | 113 | 72 | 0.87 | 21.00 | 0.0065 | 15.35 | 1.03 | 0.18 | 0.73 | 1.03 | 0.09 |
| *HERPUD2* | 212 | 122 | 121 | 115 | 0.95 | 24.85 | 0.0041 | 16.29 | 1.48 | -1.01 | 0.40 | 1.80 | 0.10 |
| *HIVEP3* | 14415 | 3943 | 3934 | 1003 | 1.00 | 356.88 | 0.0007 | 526.40 | -0.95 | -9.31 | -4.62 | -0.14 | 0.04 |
| *HLA_B* | 297 | 246 | 226 | 710 | 0.99 | 54.42 | 0.0165 | 32.84 | 1.89 | 1.99 | 2.24 | 1.60 | 0.12 |
| *HLA_DQ* | 836 | 793 | 778 | 595 | 0.98 | 259.20 | 0.0372 | 105.87 | 4.24 | 4.03 | 4.76 | 3.61 | 0.17 |
| *HLA_F* | 128 | 51 | 50 | 32 | 0.84 | 8.12 | 0.0021 | 6.81 | 0.51 | -2.37 | -1.27 | 0.97 | 0.08 |
| *HLA_G* | 185 | 95 | 94 | 38 | 0.83 | 24.09 | 0.0058 | 12.68 | 2.50 | 0.35 | 1.77 | 2.59 | 0.13 |
| *HLAC* | 319 | 268 | 254 | 602 | 0.99 | 62.61 | 0.0185 | 35.78 | 2.17 | 2.30 | 2.59 | 1.86 | 0.12 |
| *HLADQB1* | 905 | 860 | 860 | 624 | 0.98 | 257.90 | 0.0358 | 114.81 | 3.65 | 4.36 | 4.46 | 2.97 | 0.15 |
| *HMP19* | 1774 | 478 | 477 | 293 | 0.97 | 23.06 | 0.0004 | 63.81 | -1.86 | -9.07 | -5.47 | -1.43 | 0.02 |
| *HNF4G* | 808 | 252 | 250 | 163 | 0.95 | 30.33 | 0.0011 | 33.64 | -0.28 | -5.41 | -2.88 | 0.41 | 0.06 |
| *IFNG* | 118 | 25 | 25 | 22 | 0.74 | 1.96 | 0.0004 | 3.34 | -1.00 | -5.84 | -4.57 | 0.32 | 0.04 |
| *IL10* | 142 | 53 | 53 | 51 | 0.84 | 5.15 | 0.0011 | 7.08 | -0.72 | -4.88 | -3.47 | 0.10 | 0.05 |
| *IL12A* | 178 | 46 | 46 | 40 | 0.81 | 5.50 | 0.0008 | 6.14 | -0.28 | -3.00 | -2.07 | 0.30 | 0.06 |
| *IL17A* | 224 | 60 | 59 | 53 | 0.77 | 5.79 | 0.0007 | 8.01 | -0.75 | -5.71 | -3.94 | 0.30 | 0.05 |
| *IL1A* | 269 | 67 | 66 | 45 | 0.81 | 11.49 | 0.0010 | 8.94 | 0.77 | -5.45 | -2.82 | 2.38 | 0.09 |
| *IL23R* | 3296 | 884 | 883 | 603 | 1.00 | 97.96 | 0.0008 | 118.02 | -0.50 | -9.41 | -4.53 | 0.64 | 0.06 |
| *IL6* | 145 | 44 | 44 | 40 | 0.75 | 4.20 | 0.0009 | 5.87 | -0.75 | -4.76 | -3.48 | 0.11 | 0.05 |
| *KCNK9* | 3439 | 964 | 959 | 888 | 1.00 | 85.38 | 0.0008 | 128.70 | -0.99 | -10.46 | -5.31 | 0.03 | 0.04 |
| *KLRC4* | 473 | 114 | 114 | 74 | 0.88 | 15.18 | 0.0007 | 15.22 | -0.01 | -5.63 | -3.12 | 1.06 | 0.07 |
| *KLRK1* | 429 | 127 | 127 | 90 | 0.87 | 13.87 | 0.0008 | 16.95 | -0.51 | -6.82 | -4.04 | 0.57 | 0.05 |
| *LILRA1* | 461 | 144 | 143 | 107 | 0.89 | 9.28 | 0.0011 | 19.22 | -1.47 | -5.57 | -3.91 | -1.04 | 0.03 |
| *LILRB1* | 901 | 283 | 282 | 256 | 0.95 | 33.40 | 0.0016 | 37.78 | -0.33 | -3.85 | -2.09 | 0.13 | 0.06 |
| *LOC100129342* | 207 | 67 | 67 | 52 | 0.83 | 4.73 | 0.0007 | 8.94 | -1.28 | -7.26 | -5.14 | -0.29 | 0.03 |
| *LOC100132252* | 1039 | 444 | 442 | 413 | 0.99 | 41.13 | 0.0014 | 59.28 | -0.89 | -5.44 | -3.13 | -0.42 | 0.05 |
| *LOC107984355* | 617 | 173 | 172 | 252 | 0.97 | 10.83 | 0.0006 | 23.10 | -1.52 | -9.40 | -5.90 | -0.71 | 0.03 |
| *LOC285830* | 655 | 365 | 364 | 151 | 0.94 | 70.75 | 0.0032 | 48.73 | 1.31 | -0.81 | 0.51 | 1.53 | 0.10 |
| *LTN1* | 1567 | 368 | 368 | 281 | 0.98 | 32.87 | 0.0005 | 49.13 | -0.96 | -9.34 | -5.08 | 0.05 | 0.04 |
| *LYST* | 5588 | 1265 | 1261 | 789 | 1.00 | 100.14 | 0.0004 | 168.88 | -1.20 | -14.32 | -7.08 | 0.34 | 0.04 |
| *MEFV* | 584 | 150 | 149 | 190 | 0.94 | 20.70 | 0.0014 | 20.03 | 0.10 | -7.88 | -4.10 | 1.78 | 0.07 |
| *MICA* | 686 | 343 | 340 | 165 | 0.96 | 62.33 | 0.0040 | 45.79 | 1.05 | 0.00 | 0.71 | 1.10 | 0.09 |
| *MN1* | 1555 | 388 | 386 | 819 | 1.00 | 30.54 | 0.0006 | 51.80 | -1.19 | -8.75 | -4.93 | -0.39 | 0.04 |
| *MOG* | 427 | 214 | 212 | 106 | 0.92 | 14.15 | 0.0009 | 28.57 | -1.45 | -3.59 | -2.77 | -1.27 | 0.03 |
| *MSX2* | 199 | 60 | 60 | 50 | 0.75 | 3.77 | 0.0006 | 8.01 | -1.43 | -5.83 | -4.41 | -0.79 | 0.03 |
| *MUC21* | 209 | 113 | 111 | 198 | 0.94 | 9.84 | 0.0016 | 15.09 | -0.98 | -0.09 | -0.66 | -1.02 | 0.04 |
| *NAV2* | 24008 | 6660 | 6649 | 1006 | 1.00 | 642.34 | 0.0008 | 889.13 | -0.82 | -9.11 | -4.42 | 0.02 | 0.05 |
| *NOD2* | 1185 | 303 | 303 | 257 | 0.96 | 24.00 | 0.0006 | 40.45 | -1.18 | -9.67 | -5.47 | -0.20 | 0.04 |
| *OSR1* | 178 | 47 | 47 | 51 | 0.71 | 1.39 | 0.0002 | 6.27 | -2.05 | -4.05 | -3.75 | -1.89 | 0.01 |
| *OVCH1* | 1882 | 522 | 521 | 455 | 0.99 | 60.17 | 0.0009 | 69.69 | -0.40 | -8.77 | -4.31 | 0.74 | 0.06 |
| *PAX8* | 1700 | 472 | 471 | 415 | 0.98 | 62.38 | 0.0010 | 63.01 | -0.03 | -7.72 | -3.60 | 1.11 | 0.07 |
| *PLEKHB1* | 459 | 141 | 141 | 95 | 0.88 | 13.85 | 0.0008 | 18.82 | -0.75 | -4.90 | -3.11 | -0.17 | 0.05 |
| *PMFBP1* | 1461 | 383 | 381 | 306 | 0.98 | 38.15 | 0.0007 | 51.13 | -0.74 | -8.90 | -4.70 | 0.33 | 0.05 |
| *POU5F1* | 190 | 88 | 88 | 47 | 0.92 | 16.43 | 0.0026 | 11.75 | 1.10 | -1.32 | -0.08 | 1.46 | 0.09 |
| *PPP1R11* | 73 | 41 | 40 | 35 | 0.72 | 2.16 | 0.0007 | 5.47 | -1.57 | -3.72 | -3.31 | -1.26 | 0.03 |
| *PSMD14* | 2268 | 550 | 549 | 423 | 0.99 | 33.03 | 0.0003 | 73.43 | -1.61 | -12.09 | -6.63 | -0.68 | 0.03 |
| *PSORS1C1* | 920 | 491 | 487 | 258 | 0.98 | 100.68 | 0.0040 | 65.55 | 1.56 | -2.10 | 0.10 | 2.04 | 0.10 |
| *PTPN22* | 1249 | 323 | 323 | 257 | 0.97 | 31.68 | 0.0005 | 43.12 | -0.77 | -9.48 | -5.08 | 0.42 | 0.05 |
| *RALGAPA2* | 8018 | 1879 | 1879 | 898 | 1.00 | 64.85 | 0.0002 | 250.85 | -2.18 | -13.97 | -7.54 | -1.67 | 0.02 |
| *RIMBP2* | 11477 | 3617 | 3603 | 1006 | 1.00 | 403.01 | 0.0013 | 482.88 | -0.49 | -8.65 | -4.02 | 0.45 | 0.06 |
| *RNF39* | 173 | 77 | 77 | 48 | 0.89 | 7.65 | 0.0014 | 10.28 | -0.70 | -2.12 | -1.68 | -0.46 | 0.05 |
| *SACM1L* | 1481 | 459 | 459 | 275 | 0.97 | 73.72 | 0.0013 | 61.28 | 0.59 | -8.06 | -3.34 | 2.11 | 0.08 |
| *SAMD3* | 6653 | 2122 | 2116 | 878 | 1.00 | 290.04 | 0.0013 | 283.29 | 0.07 | -5.75 | -2.42 | 0.79 | 0.07 |
| *SEMA6D* | 15970 | 4321 | 4316 | 997 | 1.00 | 478.33 | 0.0008 | 576.87 | -0.50 | -9.10 | -4.21 | 0.49 | 0.06 |
| *SGPP2* | 3802 | 1020 | 1015 | 882 | 1.00 | 94.38 | 0.0007 | 136.17 | -0.90 | -8.79 | -4.51 | -0.09 | 0.05 |
| *SLC41A2* | 3845 | 1127 | 1126 | 550 | 0.99 | 89.81 | 0.0006 | 150.46 | -1.18 | -10.05 | -5.23 | -0.34 | 0.04 |
| *SLC43A3* | 603 | 164 | 164 | 170 | 0.95 | 8.76 | 0.0004 | 21.89 | -1.71 | -10.50 | -6.61 | -0.80 | 0.03 |
| *SLC44A4* | 406 | 138 | 138 | 136 | 0.94 | 17.17 | 0.0011 | 18.42 | -0.19 | -4.87 | -2.74 | 0.57 | 0.06 |
| *SLIT2* | 10545 | 2767 | 2761 | 991 | 1.00 | 246.09 | 0.0007 | 369.40 | -0.98 | -9.51 | -4.76 | -0.17 | 0.04 |
| *SMARCA2* | 6721 | 1835 | 1824 | 966 | 1.00 | 141.14 | 0.0008 | 244.98 | -1.25 | -9.18 | -4.83 | -0.57 | 0.04 |
| *SMG6* | 6965 | 1925 | 1923 | 859 | 1.00 | 206.73 | 0.0008 | 256.99 | -0.58 | -11.25 | -5.24 | 0.80 | 0.05 |
| *SORBS2* | 11721 | 3862 | 3857 | 1003 | 1.00 | 483.58 | 0.0013 | 515.59 | -0.18 | -8.12 | -3.58 | 0.81 | 0.06 |
| *STAT4* | 3720 | 946 | 943 | 833 | 1.00 | 87.81 | 0.0006 | 126.29 | -0.89 | -8.99 | -4.60 | -0.03 | 0.05 |
| *STK39* | 8547 | 2574 | 2572 | 915 | 1.00 | 286.69 | 0.0010 | 343.64 | -0.49 | -9.89 | -4.57 | 0.67 | 0.06 |
| *STX8* | 10346 | 3118 | 3112 | 1004 | 1.00 | 288.84 | 0.0009 | 416.26 | -0.90 | -10.07 | -4.93 | 0.04 | 0.05 |
| *SUMO4* | 36 | 11 | 11 | 11 | 0.51 | 1.02 | 0.0013 | 1.47 | -0.62 | -5.61 | -4.49 | 1.74 | 0.05 |
| *SUSD1* | 3622 | 960 | 959 | 891 | 1.00 | 102.66 | 0.0008 | 128.16 | -0.58 | -9.97 | -4.81 | 0.61 | 0.05 |
| *TCF19* | 190 | 80 | 79 | 42 | 0.92 | 14.48 | 0.0019 | 10.68 | 0.98 | -0.98 | 0.02 | 1.25 | 0.09 |
| *TENM4* | 23487 | 6583 | 6569 | 1004 | 1.00 | 607.65 | 0.0008 | 878.85 | -0.91 | -10.63 | -5.12 | 0.10 | 0.05 |
| *TFCP2L1* | 2031 | 536 | 535 | 575 | 0.99 | 42.00 | 0.0006 | 71.56 | -1.21 | -8.82 | -4.87 | -0.46 | 0.04 |
| *TLR4* | 338 | 75 | 75 | 73 | 0.87 | 5.31 | 0.0004 | 10.01 | -1.29 | -4.76 | -3.60 | -0.81 | 0.04 |
| *TMEM132B* | 9972 | 2823 | 2820 | 995 | 1.00 | 282.41 | 0.0008 | 376.88 | -0.74 | -7.41 | -3.68 | -0.07 | 0.05 |
| *TNF* | 61 | 19 | 19 | 21 | 0.36 | 0.64 | 0.0002 | 2.54 | -1.72 | -6.05 | -5.18 | -0.84 | 0.02 |
| *TNFAIP3* | 381 | 96 | 96 | 89 | 0.78 | 6.07 | 0.0004 | 12.82 | -1.46 | -6.69 | -4.71 | -0.83 | 0.03 |
| *TRIM31* | 338 | 163 | 163 | 194 | 0.96 | 19.32 | 0.0019 | 21.76 | -0.32 | -3.76 | -2.18 | 0.18 | 0.06 |
| *TTLL7* | 3060 | 835 | 835 | 537 | 0.99 | 76.29 | 0.0006 | 111.48 | -0.92 | -8.28 | -4.33 | -0.17 | 0.05 |
| *UBAC2* | 5101 | 1355 | 1353 | 722 | 1.00 | 111.15 | 0.0006 | 180.90 | -1.13 | -9.02 | -4.72 | -0.42 | 0.04 |
| *UBASH3B* | 4485 | 1274 | 1272 | 934 | 1.00 | 151.43 | 0.0010 | 170.08 | -0.32 | -8.16 | -3.79 | 0.66 | 0.06 |
| *UBD* | 120 | 50 | 49 | 29 | 0.77 | 4.12 | 0.0010 | 6.68 | -1.01 | -3.95 | -3.06 | -0.43 | 0.04 |
| *ZNRD1* | 85 | 33 | 33 | 29 | 0.82 | 3.28 | 0.0009 | 4.41 | -0.65 | 0.19 | -0.22 | -0.71 | 0.05 |

**Supplemental Table 7.** Genes with highest quartile population genetic parameter values in East Asian, African, and European 1K Genomes populations. Only the highest quartile values are presented for clarity.

|  | **East Asians** | | | | | | |  | **Africans** | | | | | | |  | **Europeans** | | | | | | |
| --- | --- | --- | --- | --- | --- | --- | --- | --- | --- | --- | --- | --- | --- | --- | --- | --- | --- | --- | --- | --- | --- | --- | --- |
| **Gene** | **HD** | **θ_k_** | **Pi** | **θ_W_** | **TD** | **FLD** | **FLF** |  | **HD** | **θ_k_** | **Pi** | **θ_W_** | **TD** | **FLD** | **FLF** |  | **HD** | **θ_k_** | **Pi** | **θ_W_** | **TD** | **FLD** | **FLF** |
| *ABCB5* | 1.00 |  |  |  |  |  |  |  | 1.00 |  |  |  |  |  |  |  | 1.00 |  |  |  |  |  |  |
| *ASB18* |  |  |  |  |  |  |  |  | 1.00 |  |  |  |  |  |  |  |  |  |  |  |  |  |  |
| *ATP8A1* | 1.00 |  |  |  |  |  |  |  | 1.00 |  |  | 454.68 |  |  |  |  | 1.00 |  |  |  |  |  |  |
| *BTNL2* |  |  | 0.00770 |  | 1.63 | 1.91 | 2.01 |  |  |  | 0.0035 |  |  |  |  |  |  |  | 0.0032 |  |  | 2.77 | 0.61 |
| *C10ORF11 (LRMDA)* | 1.00 | 651.76 |  | 1081.36 |  |  |  |  | 1.00 | 872.90 |  | 1908.62 |  |  |  |  | 1.00 | 618.55 |  | 1048.67 |  |  |  |
| *C6ORF10 (TSBP1)* |  |  | 0.00270 |  | 1.44 | -0.19 | 0.93 |  |  |  |  |  | 0.72 |  | -0.64 |  |  |  |  |  | 1.1 | -0.44 | 0.58 |
| *C6ORF15* |  |  | 0.00380 |  | 1.23 |  | -0.84 |  |  |  | 0.0028 |  |  |  |  |  |  |  | 0.0036 |  |  |  |  |
| *C6ORF85 (SLC22A23)* | 1.00 | 233.92 |  |  |  |  |  |  | 1.00 | 277.29 |  |  |  |  |  |  | 1.00 |  |  |  |  |  |  |
| *CCHCR1* |  |  |  |  |  | -1.46 | -0.44 |  |  |  | 0.0028 |  | 0.45 | -2.42 | -0.84 |  |  |  | 0.0029 |  |  |  |  |
| *COL12A1* | 1.00 |  |  |  |  |  |  |  | 1.00 |  |  |  |  |  |  |  | 1.00 |  |  |  |  |  |  |
| *CPLX1* |  |  |  |  |  |  |  |  | 1.00 |  |  |  |  |  |  |  | 1.00 |  |  |  |  |  |  |
| *CPVL* | 1.00 |  |  |  |  |  |  |  | 1.00 |  |  |  |  |  |  |  | 1.00 |  |  |  |  |  |  |
| *CTNNA2* | 1.00 | 955.57 |  | 1298.64 |  |  |  |  | 1.00 | 1314.89 |  | 2259.35 |  |  |  |  | 1.00 | 861.27 |  | 1254.40 |  |  |  |
| *DHFRP2* |  |  | 0.00640 |  | 1.44 | 0.53 | 1.23 |  |  |  | 0.0069 |  | 1.30 | -0.84 | 0.40 |  |  |  | 0.0071 |  | 2.78 | 1.05 | 2.32 |
| *DNMT3A* | 1.00 |  |  |  |  |  |  |  | 1.00 |  |  |  |  |  |  |  | 1.00 |  |  |  |  |  |  |
| *EBF2* | 1.00 |  |  |  |  |  |  |  | 1.00 |  |  |  |  |  |  |  | 1.00 |  |  |  |  |  |  |
| *GALNT1* | 1.00 |  |  |  |  |  |  |  | 1.00 |  |  |  |  |  |  |  | 1.00 |  |  |  |  |  |  |
| *GALT10* | 1.00 |  |  |  |  |  |  |  | 1.00 | 292.02 |  |  |  |  |  |  | 1.00 | 247.65 |  |  |  |  |  |
| *GAS2* | 1.00 |  |  |  |  |  |  |  | 1.00 |  |  |  |  |  |  |  | 1.00 |  |  |  |  |  |  |
| *HCG27* |  |  | 0.00430 |  | 1.71 | -1.45 | 0.32 |  |  |  | 0.0038 |  | 0.67 | -0.48 | 0.18 |  |  |  | 0.0041 |  | 1.48 | -1.01 | 0.40 |
| *HCG9* |  |  | 0.00620 |  | 0.81 | 0.70 | 0.87 |  |  |  | 0.0077 |  | 1.48 | 0.55 | 1.26 |  |  |  | 0.0065 |  | 1.03 | 0.18 | 0.73 |
| *HERPUD2* |  |  | 0.00430 |  | 1.71 | -1.45 | 0.32 |  |  |  | 0.0038 |  | 0.67 | -0.48 | 0.18 |  |  |  | 0.0041 |  | 1.48 | -1.01 | 0.40 |
| *HIVEP3* | 1.00 | 299.71 |  | 491.70 |  |  |  |  | 1.00 | 519.53 |  |  |  |  |  |  | 1.00 | 356.88 |  | 526.40 |  |  |  |
| *HLA_B* |  |  | 0.01660 |  | 1.51 | -0.94 | 0.52 |  | 1.00 |  | 0.0153 |  | 1.13 | 2.56 | 1.93 |  |  |  | 0.0165 |  | 1.89 | 1.990 | 2.24 |
| *HLA_DQ* |  | 241.87 | 0.03470 |  | 3.72 | 4.08 | 4.43 |  |  |  | 0.0340 |  | 3.77 | 3.96 | 4.37 |  |  | 259.20 | 0.0372 |  | 4.24 | 4.030 | 4.76 |
| *HLA_F* |  |  |  |  |  |  |  |  |  |  |  |  | 0.25 |  |  |  |  |  |  |  |  |  |  |
| *HLA_G* |  |  | 0.00550 |  | 1.98 |  | 0.34 |  |  |  | 0.0062 |  | 1.64 | -1.91 | 0.04 |  |  |  | 0.0058 |  | 2.5 | 0.350 | 1.77 |
| *HLAC* |  |  | 0.01630 |  | 2.21 | -0.35 | 1.26 |  | 1.00 |  | 0.0188 |  | 2.10 | 1.95 | 2.36 |  |  |  | 0.0185 |  | 2.17 | 2.300 | 2.59 |
| *HLADQB1* |  | 278.42 | 0.03860 |  | 4.17 | 4.36 | 4.82 |  |  |  | 0.0340 |  | 3.46 | -0.10 | 2.39 |  |  | 257.90 | 0.0358 |  | 3.65 | 4.360 | 4.46 |
| *IL23R* | 1.00 |  |  |  |  |  |  |  | 1.00 |  |  |  |  |  |  |  | 1.00 |  |  |  |  |  |  |
| *KCNK9* | 1.00 |  |  |  |  |  |  |  | 1.00 |  |  |  |  |  |  |  | 1.00 |  |  |  |  |  |  |
| *LOC285830* |  |  | 0.00320 |  | 1.57 | -1.06 | 0.55 |  |  |  | 0.0035 |  | 1.04 |  | -0.72 |  |  |  | 0.0032 |  | 1.31 | -0.810 | 0.51 |
| *LYST* | 1.00 |  |  |  |  |  |  |  | 1.00 |  |  |  |  |  |  |  | 1.00 |  |  |  |  |  |  |
| *MICA* |  |  | 0.00420 |  |  |  |  |  |  |  | 0.0044 |  |  | 0.26 | 0.09 |  |  |  | 0.0040 |  | 1.05 | 0.000 | 0.71 |
| *MN1* | 1.00 |  |  |  |  |  |  |  | 1.00 |  |  |  |  |  |  |  | 1.00 |  |  |  |  |  |  |
| *MUC21* |  |  |  |  |  |  |  |  |  |  |  |  |  | -0.68 |  |  |  |  |  |  |  | -0.090 |  |
| *NAV2* | 1.00 | 664.45 |  | 875.55 |  |  |  |  | 1.00 | 988.67 |  | 1605.28 |  |  |  |  | 1.00 | 642.34 |  | 889.13 |  |  |  |
| *POU5F1* |  |  |  |  | 0.70 |  |  |  |  |  |  |  |  | -2.35 |  |  |  |  |  |  | 1.1 |  |  |
| *PPP1R11* |  |  |  |  |  | -1.38 |  |  |  |  |  |  |  |  |  |  |  |  |  |  |  |  |  |
| *PSORS1C1* |  |  | 0.00400 |  | 0.98 | -0.76 | 0.33 |  |  |  | 0.0038 |  | 0.76 |  |  |  |  |  | 0.0040 |  | 1.56 |  | 0.10 |
| *RALGAPA2* | 1.00 |  |  | 260.66 |  |  |  |  | 1.00 |  |  | 468.72 |  |  |  |  | 1.00 |  |  | 250.85 |  |  |  |
| *RIMBP2* | 1.00 | 412.77 |  | 453.12 |  |  |  |  | 1.00 | 506.77 |  | 782.10 |  |  |  |  | 1.00 | 403.01 |  | 482.88 |  |  |  |
| *SAMD3* | 1.00 | 308.60 |  | 272.81 |  |  |  |  | 1.00 | 319.27 |  | 474.51 |  |  |  |  | 1.00 | 290.04 |  | 283.29 |  |  |  |
| *SEMA6D* | 1.00 | 471.63 |  | 623.29 |  |  |  |  | 1.00 | 556.11 |  | 1054.65 |  |  |  |  | 1.00 | 478.33 |  | 576.87 |  |  |  |
| *SGPP2* | 1.00 |  |  |  |  |  |  |  | 1.00 |  |  |  |  |  |  |  | 1.00 |  |  |  |  |  |  |
| *SLC41A2* | 1.00 |  |  |  |  |  |  |  | 1.00 |  |  |  |  |  |  |  |  |  |  |  |  |  |  |
| *SLIT2* | 1.00 |  |  | 379.72 |  |  |  |  | 1.00 | 278.11 |  | 655.35 |  |  |  |  | 1.00 |  |  | 369.40 |  |  |  |
| *SMARCA2* | 1.00 |  |  |  |  |  |  |  | 1.00 |  |  |  |  |  |  |  | 1.00 |  |  |  |  |  |  |
| *SMG6* | 1.00 |  |  | 263.60 |  |  |  |  | 1.00 |  |  |  |  |  |  |  | 1.00 |  |  | 256.99 |  |  |  |
| *SORBS2* | 1.00 | 434.77 |  | 469.54 |  |  |  |  | 1.00 | 561.34 |  | 790.08 |  |  |  |  | 1.00 | 483.58 |  | 515.59 |  |  |  |
| *STAT4* | 1.00 |  |  |  |  |  |  |  | 1.00 |  |  |  |  |  |  |  | 1.00 |  |  |  |  |  |  |
| *STK39* | 1.00 | 321.08 |  | 312.45 |  |  |  |  | 1.00 | 405.22 |  | 585.41 |  |  |  |  | 1.00 | 286.69 |  | 343.64 |  |  |  |
| *STX8* | 1.00 | 217.81 |  | 376.38 |  |  |  |  | 1.00 | 437.06 |  | 695.41 |  |  |  |  | 1.00 | 288.84 |  | 416.26 |  |  |  |
| *SUMO4* |  |  |  |  |  |  |  |  |  |  |  |  |  | -0.05 | -0.71 |  |  |  |  |  |  |  |  |
| *SUSD1* | 1.00 |  |  |  |  |  |  |  | 1.00 |  |  |  |  |  |  |  | 1.00 |  |  |  |  |  |  |
| *TCF19* |  |  |  |  |  |  |  |  |  |  |  |  |  |  |  |  |  |  |  |  | 0.98 | -0.98 | 0.02 |
| *TENM4* |  | 558.50 |  | 828.70 |  |  |  |  | 1.00 | 838.79 |  | 1504.17 |  |  |  |  | 1.00 | 607.65 |  | 878.85 |  |  |  |
| *TFCP2L1* |  |  |  |  |  |  |  |  | 1.00 |  |  |  |  |  |  |  |  |  |  |  |  |  |  |
| *TMEM132B* | 1.00 | 256.79 |  | 369.17 |  |  |  |  | 1.00 | 370.74 |  | 675.06 |  |  |  |  | 1.00 | 282.41 |  | 376.88 |  |  |  |
| *TTLL7* |  |  |  |  |  |  |  |  | 1.00 |  |  |  |  |  |  |  |  |  |  |  |  |  |  |
| *UBAC2* | 1.00 |  |  |  |  |  |  |  | 1.00 |  |  |  |  |  |  |  | 1.00 |  |  |  |  |  |  |
| *UBASH3B* | 1.00 |  |  |  |  |  |  |  | 1.00 |  |  |  |  |  |  |  | 1.00 |  |  |  |  |  |  |
| *UBD* |  |  |  |  |  |  |  |  |  |  |  |  | 0.33 |  |  |  |  |  |  |  |  |  |  |
| *ZNRD1* |  |  |  |  |  | -0.67 |  |  |  |  |  |  |  | -0.86 | -0.46 |  |  |  |  |  |  | 0.19 |  |

HD: Haplotype diversity; θ_k_:Theta-K; θ_W_:Theta-Watterson; TD:Tajima's D; FLD: Fu-Li’s D; FLF: Fu-Li’s F

**Supplemental Table 8.** Genes with lowest quartile population genetic parameter values in East Asian, African, and European 1K Genomes populations. Only the lowest quartile values are presented for clarity.

|  | **East Asians** | | | | | | |  | **Africans** | | | | | | |  | **Europeans** | | | | | | |
| --- | --- | --- | --- | --- | --- | --- | --- | --- | --- | --- | --- | --- | --- | --- | --- | --- | --- | --- | --- | --- | --- | --- | --- |
| **Gene** | **HD** | **θ_k_** | **Pi** | **θ_W_** | **TD** | **FLD** | **FLF** |  | **HD** | **θ_k_** | **Pi** | **θ_W_** | **TD** | **FLD** | **FLF** |  | **HD** | **θ_k_** | **Pi** | **θ_W_** | **TD** | **FLD** | **FLF** |
| *API5* |  |  | 0.0002 |  | -2.11 |  | -6.77 |  |  |  | 0.0003 |  | -2.1 |  | -5.45 |  |  |  | 0.0003 |  | -1.67 | -10.45 | -6.44 |
| *C10ORF11 (LRMDA)* |  |  |  |  |  | -12.52 |  |  |  |  |  |  |  | -9.29 |  |  |  |  |  |  |  | -11.28 | -5.61 |
| *C6ORF15* |  |  |  | 3.2 |  |  |  |  | 0.74 | 3.58 |  | 4.89 |  |  |  |  | 0.73 | 4.58 |  | 3.6 |  |  |  |
| *C6ORF47* |  | 0.97 |  | 1.74 |  |  |  |  | 0.75 | 1.11 | 0.0005 | 3.09 |  |  |  |  | 0.68 | 0.87 |  | 2 |  |  |  |
| *CCR1* | 0.28 | 0.99 | 0.0002 | 5.21 | -2.09 |  |  |  | 0.76 | 2.61 | 0.0004 | 9.53 | -1.96 |  |  |  | 0.47 | 2.1 | 0.0003 | 6.41 | -1.77 |  | -6.12 |
| *CDH26* |  |  |  |  | -1.9 |  | -6.39 |  |  |  |  |  |  | -8.99 | -4.97 |  |  |  | 0.0003 |  | -2.17 | -10.92 | -6.57 |
| *CEP135* |  |  |  |  |  |  |  |  |  |  |  |  | -1.9 |  |  |  |  |  |  |  |  | -10.11 | -5.46 |
| *COL12A1* |  |  |  |  |  | -12.75 | -6.63 |  |  |  |  |  |  | -9.44 | -5.11 |  |  |  | 0.0002 |  | -2.14 | -11.4 | -6.57 |
| *CTLA4* | 0.67 | 1.47 | 0.00024 | 6.41 | -2.03 |  |  |  | 0.82 | 2.44 | 0.0004 | 7.73 | -1.82 |  | -5.04 |  | 0.66 | 1.37 | 0.00023 | 3.47 | -1.48 |  |  |
| *CTNNA2* |  |  |  |  |  |  |  |  |  |  |  |  |  |  |  |  |  |  |  |  |  | -10.11 |  |
| *DHFRP2* |  |  |  |  |  |  |  |  | 0.83 |  |  |  |  |  |  |  |  |  |  |  |  |  |  |
| *DNMT3A* |  |  |  |  |  | -13.07 | -6.88 |  |  |  |  |  | -1.75 | -9.6 | -5.25 |  |  |  |  |  |  | -10.87 | -5.7 |
| *DTL* |  |  |  |  |  |  |  |  |  |  |  |  | -1.81 |  |  |  |  |  |  |  |  |  |  |
| *FUT2* |  | 4.26 |  |  | -1.93 |  |  |  |  |  |  |  |  |  |  |  |  |  |  |  |  |  |  |
| *GABBR1* |  |  |  |  |  |  |  |  |  |  | 0.0006 |  |  |  |  |  |  |  |  |  | -1.59 |  |  |
| *GAS2* |  |  |  |  |  |  |  |  |  |  |  |  |  |  |  |  |  |  |  |  |  | -10.62 |  |
| *GIMAP1* |  |  |  |  |  |  |  |  |  |  |  |  |  |  |  |  | 0.72 | 4.58 |  | 6.41 |  |  |  |
| *GIMAP4* |  |  |  |  |  |  |  |  | 0.89 |  |  | 11.08 |  |  |  |  |  |  |  |  |  |  |  |
| *HLA_F* |  |  |  | 6.94 |  |  |  |  |  |  |  | 9.53 |  |  |  |  |  |  |  | 6.81 |  |  |  |
| *HMP19* |  |  | 0.0002 |  | -2.43 |  | -6.44 |  |  |  |  |  |  |  |  |  |  |  | 0.0004 |  | -1.86 |  | -5.47 |
| *IFNG* | 0.62 | 1.45 | 0.0003 | 3.74 |  |  | -6.16 |  | 0.85 |  | 0.0005 | 7.6 | -1.86 |  |  |  | 0.74 | 1.96 | 0.0004 | 3.34 |  |  |  |
| *IL10* |  | 2.87 |  | 5.07 |  |  |  |  | 0.85 | 4.59 |  | 7.47 |  |  |  |  |  |  |  |  |  |  |  |
| *IL12A* | 0.59 | 3.75 |  | 5.74 |  |  |  |  |  | 5.98 |  | 10.56 |  |  |  |  |  |  |  | 6.14 |  |  |  |
| *IL17A* |  |  |  |  |  |  |  |  |  | 6.17 |  |  |  |  |  |  | 0.77 |  |  |  |  |  |  |
| *IL1A* | 0.57 |  |  |  |  |  |  |  |  |  |  |  |  |  |  |  |  |  |  |  |  |  |  |
| *IL23R* |  |  |  |  |  | -11.43 |  |  |  |  |  |  |  |  |  |  |  |  |  |  |  |  |  |
| *IL6* | 0.5 | 1.53 | 0.0003 | 6.94 | -2.07 |  |  |  |  | 4.51 |  | 8.5 |  |  |  |  | 0.75 | 4.2 |  | 5.87 |  |  |  |
| *KCNK9* |  |  |  |  |  | -12.62 | -6.26 |  |  |  |  |  |  |  |  |  |  |  |  |  |  | -10.46 | -5.31 |
| *LILRA1* |  |  |  |  |  |  |  |  |  |  |  |  |  |  |  |  |  |  |  |  | -1.47 |  |  |
| *LOC100129342* | 0.65 |  |  |  |  |  |  |  |  | 3.99 | 0.0006 |  | -1.87 |  |  |  |  | 4.73 |  |  |  |  |  |
| *LOC107984355* |  |  |  |  |  |  |  |  |  |  |  |  |  | -8.77 | -5.2 |  |  |  |  |  | -1.52 |  | -5.9 |
| *LTN1* |  |  |  |  |  | -11.92 | -6.38 |  |  |  | 0.0006 |  |  | -9.26 | -5.17 |  |  |  |  |  |  |  |  |
| *LYST* |  |  | 0.0002 |  | -2.27 | -13.74 | -7.58 |  |  |  |  |  |  | -8.61 |  |  |  |  | 0.0004 |  |  | -14.32 | -7.08 |
| *MOG* |  |  |  |  | -1.8 |  |  |  |  |  |  |  |  |  |  |  |  |  |  |  |  |  |  |
| *MSX2* | 0.65 | 1.94 | 0.0003 | 6.81 | -1.9 |  |  |  |  |  |  |  |  |  |  |  | 0.75 | 3.77 |  |  |  |  |  |
| *NOD2* |  |  | 0.0003 |  | -2.07 | -11.73 | -7.04 |  |  |  |  |  | -1.76 |  | -5.03 |  |  |  |  |  |  |  | -5.47 |
| *OSR1* | 0.44 | 0.48 | 0.0001 | 4.4 | -2.25 |  | -7.66 |  | 0.79 | 2.87 | 0.0004 |  | -2.04 |  | -5.43 |  | 0.71 | 1.39 | 0.0002 | 6.27 | -2.05 |  |  |
| *PMFBP1* |  |  |  |  |  |  |  |  |  |  |  |  |  | -10.36 | -5.64 |  |  |  |  |  |  |  |  |
| *PPP1R11* |  | 2.16 |  | 4.4 |  |  |  |  | 0.85 | 3.02 |  | 4.51 |  |  |  |  | 0.72 | 2.16 |  | 5.47 | -1.57 |  |  |
| *PTPN22* |  |  |  |  |  |  |  |  |  |  | 0.00039 |  | -1.94 |  |  |  |  |  |  |  |  |  |  |
| *PSMD14* |  |  | 0.0003 |  |  | -13.68 | -7.28 |  |  |  | 0.0003 |  | -2.22 | -9.73 | -5.69 |  |  |  | 0.0003 |  | -1.61 | -12.09 | -6.63 |
| *RALGAPA2* |  |  |  |  |  | -13.06 | -6.78 |  |  |  | 0.0004 |  | -2.06 | -9.72 | -5.41 |  |  |  | 0.0002 |  | -2.18 | -13.97 | -7.54 |
| *SEMA6D* |  |  |  |  |  |  |  |  |  |  |  |  |  | -9.12 |  |  |  |  |  |  |  |  |  |
| *SLC43A3* |  |  | 0.0003 |  | -2.02 |  | -6.64 |  |  |  |  |  |  |  |  |  |  |  | 0.0004 |  | -1.71 | -10.5 | -6.61 |
| *SLIT2* |  |  |  |  |  |  |  |  |  |  |  |  |  | -9.28 |  |  |  |  |  |  |  |  |  |
| *SMARCA2* |  |  |  |  |  | -11.9 |  |  |  |  |  |  |  |  |  |  |  |  |  |  |  |  |  |
| *SMG6* |  |  |  |  |  | -12.11 |  |  |  |  |  |  |  | -10.61 | -5.23 |  |  |  |  |  |  | -11.25 |  |
| *STAT4* |  |  |  |  |  | -11.46 |  |  |  |  |  |  |  | -8.69 |  |  |  |  |  |  |  |  |  |
| *STX8* |  |  |  |  |  | -11.57 |  |  |  |  |  |  |  |  |  |  |  |  |  |  |  |  |  |
| *SUMO4* | 0.4 | 0.79 |  | 1.07 |  |  |  |  | 0.54 | 0.69 |  | 1.93 |  |  |  |  | 0.51 | 1.02 |  | 1.47 |  |  |  |
| *TCF19* |  |  |  |  |  |  |  |  | 0.9 |  |  |  |  |  |  |  |  |  |  |  |  |  |  |
| *TENM4* | 0.3 |  |  |  |  | -11.78 |  |  |  |  |  |  |  |  |  |  |  |  |  |  |  | -10.63 |  |
| *TFCP2L1* | 0.33 |  |  |  |  |  |  |  |  |  |  |  |  |  |  |  |  |  |  |  |  |  |  |
| *TLR4* |  | 3.69 | 0.0003 |  | -1.8 |  | -6.65 |  |  |  |  |  |  |  |  |  |  |  | 0.0004 |  |  |  |  |
| *TNF* | 0.33 | 0.47 | 0.0002 | 1.74 |  |  |  |  | 0.42 | 0.75 | 0.0003 | 3.48 | -1.89 |  | -5.27 |  | 0.36 | 0.64 | 0.0002 | 2.54 | -1.72 |  |  |
| *TNFAIP3* | 0.69 | 3.72 | 0.0002 |  | -2.13 |  |  |  |  |  |  |  |  |  |  |  | 0.78 |  | 0.0004 |  |  |  |  |
| *TTLL7* |  |  |  |  |  |  |  |  |  |  | 0.0006 |  |  |  |  |  |  |  |  |  |  |  |  |
| *UBAC2* |  |  |  |  |  |  |  |  |  |  | 0.0006 |  | -1.85 | -9.22 | -5.1 |  |  |  |  |  |  |  |  |
| *UBD* |  |  |  | 6.41 |  |  |  |  | 0.81 |  |  | 8.63 |  |  |  |  | 0.77 | 4.12 |  | 6.68 |  |  |  |
| *ZNRD1* |  |  |  |  |  |  |  |  | 0.8 | 5.38 |  | 5.02 |  |  |  |  |  | 3.28 |  | 4.41 |  |  |  |

HD: Haplotype diversity; θ_k_:Theta-K; θ_W_:Theta-Watterson; TD:Tajima's D; FLD: Fu-Li’s D; FLF: Fu-Li’s F

**Supplemental Table 9.** Genetic differentiation parameter estimates of East Asians (EAS) with respect to Africans (AFR), and its effect on nucleotide diversity in East Asians

| **Gene** | **AFR_EAS_Dxy** | **AFR_EAS_Hst** | **AFR_EAS_Fst** | **EAS_Pi/AFR_EAS_Fst** | **EAS_Pi/AFR_EAS_Dxy** |
| --- | --- | --- | --- | --- | --- |
| *ABC5* | 0.0012805 | 5.7482E-05 | 0.13561221 | 0.005899174 | 0.624756645 |
| *API5* | 0.00038164 | 0.02132157 | 0.25981622 | 0.000769775 | 0.52405105 |
| *ASB18* | 0.00156873 | 0.00630468 | 0.23580367 | 0.003816734 | 0.57371188 |
| *ATP8A1* | 0.00097434 | 0.00018203 | 0.14205202 | 0.004927772 | 0.718438701 |
| *BAG6* | 0.00086794 | 0.01234207 | 0.10969875 | 0.007292699 | 0.921726246 |
| *BTNL2* | 0.00592129 | 0.01035004 | 0.05537937 | 0.139040939 | 1.300392174 |
| *C10ORF11* | 0.00079738 | 0 | 0.15079451 | 0.003978925 | 0.752466944 |
| *C6ORF10* | 0.00283765 | 0.00432596 | 0.06756536 | 0.039961305 | 0.951489924 |
| *C6ORF15* | 0.00386485 | 0.03733819 | 0.14358921 | 0.026464383 | 0.983220968 |
| *C6ORF47* | 0.00048594 | 0.04189384 | 0.12416654 | 0.00322148 | 0.823152535 |
| *C6ORF85* | 0.0015689 | 1.7216E-05 | 0.13048273 | 0.009196619 | 0.764866461 |
| *CCDC180* | 0.00102377 | 0.00623006 | 0.08668254 | 0.010382714 | 0.879107419 |
| *CCHCR1* | 0.0027174 | 0.01776057 | 0.04246738 | 0.056513964 | 0.88319623 |
| *CCR1* | 0.00033504 | 0.06951883 | 0.16353413 | 0.001222986 | 0.596951819 |
| *CCR3* | 0.00099889 | 0.04256231 | 0.08311031 | 0.009625761 | 0.800890712 |
| *CDH26* | 0.00072376 | 0.00565823 | 0.22993393 | 0.001304723 | 0.414504764 |
| *CEP135* | 0.00053182 | 0.00671425 | 0.08494813 | 0.004708756 | 0.75212784 |
| *COL12A1* | 0.00062252 | 0.00027683 | 0.07571757 | 0.006603487 | 0.803191889 |
| *CPLX1* | 0.00155238 | 0.00269676 | 0.14803493 | 0.008106195 | 0.773005484 |
| *CPVL* | 0.00113394 | 5.8396E-05 | 0.17756689 | 0.004505344 | 0.705503304 |
| *CTNNA2* | 0.00115792 | 0 | 0.13686432 | 0.005845205 | 0.690893376 |
| *DEPDC1* | 0.00079559 | 0.04686008 | 0.14581231 | 0.003429066 | 0.628467084 |
| *DHFRP2* | 0.00693959 | 0.05326073 | 0.04487538 | 0.142617171 | 0.922245075 |
| *DNMT3A* | 0.00052854 | 0.00061307 | 0.06197857 | 0.006453844 | 0.756797469 |
| *DTL* | 0.00059926 | 0.00990656 | 0.09099666 | 0.004395766 | 0.667486599 |
| *EBF2* | 0.00104801 | 0.00012816 | 0.21797327 | 0.003211403 | 0.667932773 |
| *ERAP1* | 0.00121127 | 0.00582362 | 0.03866646 | 0.028448425 | 0.908136337 |
| *FUT2* | 0.00190189 | 0.04106919 | 0.35903835 | 0.001114087 | 0.210317018 |
| *GABBR1* | 0.00075628 | 0.01310144 | 0.02821912 | 0.028349576 | 1.057803914 |
| *GALNT1* | 0.00114819 | 0.00054958 | 0.15271414 | 0.005893364 | 0.783845176 |
| *GALT10* | 0.00137402 | 9.6625E-05 | 0.28090408 | 0.002491954 | 0.509455183 |
| *GAS2* | 0.00136807 | 0.00036999 | 0.19504514 | 0.004614316 | 0.657859544 |
| *GIMAP1* | 0.00087205 | 0.07078605 | 0.1044166 | 0.006703915 | 0.802709041 |
| *GIMAP2* | 0.00087793 | 0.03306419 | 0.12058733 | 0.005804922 | 0.797329212 |
| *GIMAP4* | 0.00112319 | 0.01932429 | 0.07458924 | 0.012066083 | 0.801291022 |
| *HCG27* | 0.00430921 | 0.01785309 | 0.06183716 | 0.069537481 | 0.997862876 |
| *HCG9* | 0.0075162 | 0.03944051 | 0.07293072 | 0.085012184 | 0.824884685 |
| *HERPUD2* | 0.00430921 | 0.01785309 | 0.06183716 | 0.069537481 | 0.997862876 |
| *HIVEP3* | 0.00095579 | 5.2317E-06 | 0.19064344 | 0.003147237 | 0.627756078 |
| *HLA_B* | 0.01658694 | 0.00149874 | 0.03703837 | 0.448183816 | 1.000787179 |
| *HLA_DQ* | 0.0350457 | 0.00541328 | 0.02026803 | 1.712056034 | 0.990135681 |
| *HLA_F* | 0.00253558 | 0.03020028 | 0.04248372 | 0.051784543 | 0.867651627 |
| *HLA_G* | 0.00601565 | 0.04240733 | 0.0319909 | 0.171923902 | 0.914281449 |
| *HLAC* | 0.01841791 | 0.00267751 | 0.0474322 | 0.343648389 | 0.885008135 |
| *HLADQB1* | 0.03806229 | 0.00315063 | 0.04591025 | 0.840770787 | 1.014127159 |
| *HMP19* | 0.00066317 | 0.00705015 | 0.29147223 | 0.000686172 | 0.30158349 |
| *HNF4G* | 0.00135791 | 0.03520107 | 0.32921853 | 0.002733746 | 0.662784924 |
| *IFNG* | 0.00041096 | 0.03768679 | 0.06903799 | 0.004345433 | 0.730001401 |
| *IL10* | 0.00093886 | 0.06975995 | 0.16665197 | 0.003600317 | 0.639074899 |
| *IL12A* | 0.00085772 | 0.08349891 | 0.18491592 | 0.002703932 | 0.582943132 |
| *IL17AAFR* | 0.00086359 | 0.02754838 | 0.10323376 | 0.007749403 | 0.92636176 |
| *IL1A* | 0.00122824 | 0.11040593 | 0.33105719 | 0.002114438 | 0.569922383 |
| *IL23R* | 0.00094427 | 0.00106876 | 0.15359628 | 0.003906345 | 0.635414347 |
| *IL6* | 0.00093007 | 0.12486276 | 0.31535736 | 0.000951302 | 0.32255781 |
| *KCNK9* | 0.00116044 | 0.00019911 | 0.12503356 | 0.006398282 | 0.68939099 |
| *KLRC4* | 0.00109544 | 0.02193595 | 0.07486845 | 0.010685409 | 0.730302964 |
| *KLRK1* | 0.00139076 | 0.05874657 | 0.14554428 | 0.006183685 | 0.647130337 |
| *LILRA1* | 0.00194443 | 0.01924533 | 0.1929125 | 0.005183697 | 0.514290003 |
| *LILRB1* | 0.00184951 | 0.01527878 | 0.10957954 | 0.01095095 | 0.648819171 |
| *LOC100129342* | 0.00071645 | 0.06571282 | 0.14274365 | 0.004903896 | 0.977036941 |
| *LOC100132252* | 0.00171863 | 0.0038551 | 0.10534126 | 0.012340844 | 0.75641656 |
| *LOC107984355* | 0.00103659 | 0.00698551 | 0.18986785 | 0.003686775 | 0.675291826 |
| *LOC285830* | 0.00347845 | 0.02443881 | 0.04205956 | 0.076082587 | 0.91994973 |
| *LTN1* | 0.00062583 | 0.00685825 | 0.07963906 | 0.006278326 | 0.798941191 |
| *LYST* | 0.00083235 | 0.00057678 | 0.4019478 | 0.000497577 | 0.240282246 |
| *MEFV* | 0.00161378 | 0.01214822 | 0.10975776 | 0.011844265 | 0.805560302 |
| *MICA* | 0.00460319 | 0.01547815 | 0.07197141 | 0.058356508 | 0.912409979 |
| *MN1* | 0.00076324 | 0.00030085 | 0.0669336 | 0.010458126 | 0.917139604 |
| *MOGAFR* | 0.00118343 | 0.02867243 | 0.11134111 | 0.005388845 | 0.507002463 |
| *MSX2* | 0.00131581 | 0.08216862 | 0.39264893 | 0.000764041 | 0.227997021 |
| *MUC21* | 0.00154524 | 0.03987265 | 0.04844684 | 0.028897653 | 0.906006414 |
| *NAV2* | 0.00127905 | 0 | 0.16165851 | 0.005567291 | 0.703646463 |
| *NOD2* | 0.0005754 | 0.03120405 | 0.14295169 | 0.002098611 | 0.52137946 |
| *OSR1* | 0.0003497 | 0.17034855 | 0.31979811 | 0.000312697 | 0.285959471 |
| *OVCH1* | 0.00107319 | 0.00300444 | 0.12001921 | 0.005832399 | 0.65226093 |
| *PAX8* | 0.00107414 | 0.00706631 | 0.13199385 | 0.006060888 | 0.744784568 |
| *PLEKHB1* | 0.00143004 | 0.02504576 | 0.20566454 | 0.00388983 | 0.559425944 |
| *PMFBP1* | 0.00076224 | 0.00900844 | 0.17306362 | 0.003466933 | 0.787149131 |
| *POU5F1* | 0.0026603 | 0.02410504 | 0.05677237 | 0.047558346 | 1.014923474 |
| *PPP1R11* | 0.00091637 | 0.06804128 | 0.06856516 | 0.010209267 | 0.763886964 |
| *PSMD14* | 0.00033656 | 0.00428091 | 0.06169975 | 0.004862257 | 0.891384462 |
| *PSOR1C1* | 0.00403958 | 0.01319398 | 0.03884967 | 0.102960973 | 0.990202199 |
| *RALGAPA2* | 0.00041099 | 7.5897E-05 | 0.05459431 | 0.007326771 | 0.973250129 |
| *RIMBP2* | 0.00160778 | 0 | 0.10625923 | 0.012234231 | 0.808566731 |
| *RNF39* | 0.00217146 | 0.04268575 | 0.04424423 | 0.04294345 | 0.874986307 |
| *SACM1L* | 0.00138771 | 0.01265404 | 0.09031849 | 0.012179124 | 0.792672555 |
| *SAMD3* | 0.00178214 | 8.1994E-05 | 0.20306306 | 0.00689441 | 0.785573739 |
| *SEMA6D* | 0.00103527 | 3.8249E-06 | 0.1586915 | 0.005041228 | 0.772742683 |
| *SGPP2* | 0.00090821 | 0.00012231 | 0.1523515 | 0.003938261 | 0.660640923 |
| *SLC41A2* | 0.00083339 | 0.0013528 | 0.05825233 | 0.013733355 | 0.959933742 |
| *SLC43A3* | 0.00060286 | 0.02201781 | 0.11820887 | 0.002537881 | 0.497631377 |
| *SLC44A4* | 0.00114936 | 0.02203171 | 0.04140163 | 0.026569001 | 0.95705508 |
| *SLT2* | 0.00074611 | 9.5314E-06 | 0.11904523 | 0.005040101 | 0.804170423 |
| *SMARC2* | 0.0013588 | 9.8568E-06 | 0.13191343 | 0.007580729 | 0.735946152 |
| *SMG6* | 0.00101546 | 0.00013985 | 0.19441147 | 0.003600611 | 0.689345669 |
| *SORBS2* | 0.00154635 | 2.8136E-07 | 0.13213861 | 0.009081373 | 0.776020577 |
| *STAT4* | 0.0008424 | 0.00029377 | 0.12284807 | 0.005698095 | 0.830963347 |
| *STK39* | 0.00138592 | 7.2581E-05 | 0.10738987 | 0.010243051 | 0.793699383 |
| *STX8* | 0.00122767 | 8.4409E-07 | 0.1805072 | 0.003877962 | 0.570187426 |
| *SUMO4* | 0.00101322 | 0.05277707 | 0.07741019 | 0.012918196 | 0.986953026 |
| *SUSD1* | 0.00095676 | 6.6496E-05 | 0.12994052 | 0.006156663 | 0.836158943 |
| *TCF19* | 0.00163821 | 0.02722069 | 0.04890716 | 0.032715045 | 0.976673455 |
| *TENM4* | 0.00107669 | 5.6273E-07 | 0.17615867 | 0.00397369 | 0.650140548 |
| *TFCP2L1* | 0.00092116 | 0.0013199 | 0.08050129 | 0.008695513 | 0.759914024 |
| *TLR4* | 0.00058999 | 0.04346907 | 0.1557513 | 0.001926148 | 0.508484303 |
| *TMEM132B* | 0.0010682 | 6.3923E-06 | 0.12510237 | 0.006394763 | 0.748921159 |
| *TNF* | 0.00023183 | 0.01357753 | 0.03728786 | 0.005363676 | 0.862691442 |
| *TNFAIP3* | 0.0007473 | 0.08480455 | 0.33596092 | 0.000595307 | 0.267630519 |
| *TRIM31* | 0.00194462 | 0.0153796 | 0.09192719 | 0.016317262 | 0.771359266 |
| *TTLL7* | 0.0005391 | 0.00231745 | 0.03903041 | 0.01024842 | 0.74197783 |
| *UBAC2* | 0.00066078 | 0.00053521 | 0.09926092 | 0.006044675 | 0.908019365 |
| *UBASH3B* | 0.00116633 | 5.4219E-05 | 0.12463549 | 0.007221057 | 0.771652069 |
| *UBD* | 0.00224815 | 0.04191855 | 0.16089466 | 0.00932287 | 0.667214369 |
| *ZNRD1* | 0.00142082 | 0.03006726 | 0.04379919 | 0.02739777 | 0.844585397 |

**Supplemental Table 10.** Genetic differentiation parameter estimates of Europeans (EUR) with respect to Africans (AFR), and its effect on nucleotide diversity in Europeans

| **Gene** | **AFR_EUR_Dxy** | **AFR_EUR_Hst** | **AFR_EUR_Fst** | **EUR_Pi/AFR_EUR_Fst** | **EUR_Pi/AFR_EUR_Dxy** |
| --- | --- | --- | --- | --- | --- |
| *ABC5* | 0.001412 | 0.000075 | 0.100525 | 0.010943 | 0.779197 |
| *API5* | 0.000413 | 0.018703 | 0.173589 | 0.001728 | 0.726256 |
| *ASB18* | 0.001509 | 0.004776 | 0.180450 | 0.005542 | 0.662568 |
| *ATP8A1* | 0.001033 | 0.000261 | 0.098049 | 0.009179 | 0.871318 |
| *BAG6* | 0.000838 | 0.011501 | 0.091561 | 0.007645 | 0.835478 |
| *BTNL2* | 0.003402 | 0.012698 | 0.016995 | 0.188291 | 0.940694 |
| *C10ORF11* | 0.000795 | 0.000000 | 0.167094 | 0.002992 | 0.628718 |
| *C6ORF10* | 0.002846 | 0.005320 | 0.072190 | 0.037401 | 0.948798 |
| *C6ORF15* | 0.003266 | 0.019265 | 0.019080 | 0.188681 | 1.102235 |
| *C6ORF47* | 0.000420 | 0.014030 | 0.033528 | 0.011930 | 0.952274 |
| *C6ORF85* | 0.001530 | 0.000045 | 0.150117 | 0.007328 | 0.718882 |
| *CCDC180* | 0.000971 | 0.004941 | 0.128244 | 0.005458 | 0.721022 |
| *CCHCR1* | 0.002864 | 0.010337 | 0.014996 | 0.193391 | 1.012509 |
| *CCR1* | 0.000422 | 0.039052 | 0.133073 | 0.002254 | 0.710094 |
| *CCR3* | 0.001098 | 0.027901 | 0.087291 | 0.011456 | 0.910614 |
| *CDH26* | 0.000714 | 0.005159 | 0.262583 | 0.001142 | 0.419941 |
| *CEP135* | 0.000558 | 0.005157 | 0.058716 | 0.008516 | 0.895281 |
| *COL12A1* | 0.000522 | 0.000404 | 0.127789 | 0.001565 | 0.383082 |
| *CPLX1* | 0.001543 | 0.001988 | 0.094168 | 0.013805 | 0.842408 |
| *CPVL* | 0.001156 | 0.000082 | 0.140615 | 0.006400 | 0.778582 |
| *CTNNA2* | 0.001125 | 0.000000 | 0.148768 | 0.005378 | 0.710891 |
| *DEPDC1* | 0.000804 | 0.020362 | 0.049995 | 0.014001 | 0.870791 |
| *DHFRP2* | 0.007067 | 0.024985 | 0.014712 | 0.482601 | 1.004626 |
| *DNMT3A* | 0.000573 | 0.000700 | 0.055874 | 0.008949 | 0.872941 |
| *DTL* | 0.000675 | 0.007949 | 0.110614 | 0.005424 | 0.889237 |
| *EBF2* | 0.001054 | 0.000187 | 0.141344 | 0.005660 | 0.759193 |
| *ERAP1* | 0.001441 | 0.003379 | 0.061116 | 0.024543 | 1.040678 |
| *FUT2* | 0.001895 | 0.045052 | 0.062705 | 0.023922 | 0.791561 |
| *GABBR1* | 0.000621 | 0.019467 | 0.008297 | 0.072315 | 0.965589 |
| *GALNT1* | 0.001085 | 0.000637 | 0.064009 | 0.015623 | 0.921322 |
| *GALT10* | 0.001328 | 0.000132 | 0.117350 | 0.009374 | 0.828447 |
| *GAS2* | 0.001315 | 0.000423 | 0.167481 | 0.005374 | 0.684662 |
| *GIMAP1* | 0.000846 | 0.072735 | 0.132964 | 0.004513 | 0.708830 |
| *GIMAP2* | 0.000864 | 0.046750 | 0.117293 | 0.005968 | 0.810147 |
| *GIMAP4* | 0.001077 | 0.037086 | 0.107608 | 0.007434 | 0.742523 |
| *HCG27* | 0.003998 | 0.015163 | 0.011426 | 0.358845 | 1.025491 |
| *HCG9* | 0.007599 | 0.021169 | 0.065147 | 0.099775 | 0.855368 |
| *HERPUD2* | 0.003998 | 0.015163 | 0.011426 | 0.358845 | 1.025491 |
| *HIVEP3* | 0.000981 | 0.000004 | 0.156547 | 0.004472 | 0.713444 |
| *HLA_B* | 0.016342 | 0.001889 | 0.026934 | 0.612616 | 1.009647 |
| *HLA_DQ* | 0.036176 | 0.003354 | 0.016535 | 2.249749 | 1.028309 |
| *HLA_F* | 0.002472 | 0.022757 | 0.035964 | 0.058391 | 0.849587 |
| *HLA_G* | 0.006193 | 0.023564 | 0.030697 | 0.188946 | 0.936536 |
| *HLAC* | 0.018984 | 0.003129 | 0.016678 | 1.109275 | 0.974479 |
| *HLADQB1* | 0.035234 | 0.001781 | 0.009714 | 3.685572 | 1.016071 |
| *HMP19* | 0.000685 | 0.009133 | 0.164031 | 0.002439 | 0.583915 |
| *HNF4G* | 0.001197 | 0.013143 | 0.158690 | 0.006932 | 0.919325 |
| *IFNG* | 0.000481 | 0.039100 | 0.097258 | 0.004113 | 0.831024 |
| *IL10* | 0.001114 | 0.042168 | 0.082892 | 0.013270 | 0.987203 |
| *IL12A* | 0.000877 | 0.034856 | 0.059971 | 0.013340 | 0.912036 |
| *IL17AAFR* | 0.000888 | 0.055895 | 0.135822 | 0.005154 | 0.788166 |
| *IL1A* | 0.001027 | 0.033194 | 0.055795 | 0.017923 | 0.973796 |
| *IL23R* | 0.000949 | 0.001274 | 0.057794 | 0.013842 | 0.842685 |
| *IL6* | 0.001082 | 0.065066 | 0.150816 | 0.005968 | 0.832133 |
| *KCNK9* | 0.001193 | 0.000152 | 0.138913 | 0.005759 | 0.670307 |
| *KLRC4* | 0.001072 | 0.033609 | 0.100728 | 0.006949 | 0.652741 |
| *KLRK1* | 0.001354 | 0.035483 | 0.162666 | 0.004918 | 0.590927 |
| *LILRA1* | 0.001995 | 0.014263 | 0.202056 | 0.005444 | 0.551510 |
| *LILRB1* | 0.002119 | 0.010055 | 0.116921 | 0.013684 | 0.755035 |
| *LOC100129342* | 0.000750 | 0.032040 | 0.196365 | 0.003565 | 0.933627 |
| *LOC100132252* | 0.001719 | 0.003235 | 0.081030 | 0.017278 | 0.814366 |
| *LOC107984355* | 0.000951 | 0.007402 | 0.133498 | 0.004494 | 0.630985 |
| *LOC285830* | 0.003417 | 0.015764 | 0.028848 | 0.110926 | 0.936442 |
| *LTN1* | 0.000626 | 0.005884 | 0.089969 | 0.005557 | 0.798859 |
| *LYST* | 0.000771 | 0.000471 | 0.173626 | 0.002304 | 0.518919 |
| *MEFV* | 0.001599 | 0.013875 | 0.062230 | 0.022497 | 0.875486 |
| *MICA* | 0.004379 | 0.009338 | 0.042159 | 0.094878 | 0.913433 |
| *MN1* | 0.000760 | 0.000376 | 0.125368 | 0.004786 | 0.788980 |
| *MOGAFR* | 0.001292 | 0.014070 | 0.059619 | 0.015096 | 0.696829 |
| *MSX2* | 0.001253 | 0.052144 | 0.245412 | 0.002445 | 0.478697 |
| *MUC21* | 0.001619 | 0.016486 | 0.017253 | 0.092738 | 0.988121 |
| *NAV2* | 0.001275 | 0.000000 | 0.170525 | 0.004691 | 0.627240 |
| *NOD2* | 0.000711 | 0.010178 | 0.096053 | 0.006247 | 0.843349 |
| *OSR1* | 0.000332 | 0.014196 | 0.088487 | 0.002260 | 0.603124 |
| *OVCH1* | 0.001096 | 0.003706 | 0.087037 | 0.010340 | 0.821281 |
| *PAX8* | 0.001087 | 0.005800 | 0.053431 | 0.018716 | 0.920293 |
| *PLEKHB1* | 0.001440 | 0.018330 | 0.203640 | 0.003928 | 0.555560 |
| *PMFBP1* | 0.000806 | 0.008134 | 0.120451 | 0.005811 | 0.868857 |
| *POU5F1* | 0.002522 | 0.014189 | 0.029626 | 0.087760 | 1.030750 |
| *PPP1R11* | 0.000945 | 0.039497 | 0.096530 | 0.007252 | 0.741020 |
| *PSMD14* | 0.000329 | 0.004181 | 0.071006 | 0.004225 | 0.911808 |
| *PSOR1C1* | 0.004252 | 0.009081 | 0.088544 | 0.045175 | 0.940657 |
| *RALGAPA2* | 0.000339 | 0.000128 | 0.078150 | 0.002559 | 0.589625 |
| *RIMBP2* | 0.001602 | 0.000000 | 0.112800 | 0.011525 | 0.811259 |
| *RNF39* | 0.001999 | 0.022618 | 0.096979 | 0.014436 | 0.700293 |
| *SACM1L* | 0.001452 | 0.006519 | 0.074012 | 0.017565 | 0.895220 |
| *SAMD3* | 0.001651 | 0.000169 | 0.165229 | 0.007868 | 0.787356 |
| *SEMA6D* | 0.001068 | 0.000006 | 0.178827 | 0.004474 | 0.749363 |
| *SGPP2* | 0.000916 | 0.000202 | 0.103034 | 0.006794 | 0.764427 |
| *SLC41A2* | 0.000742 | 0.001880 | 0.075972 | 0.007898 | 0.808997 |
| *SLC43A3* | 0.000644 | 0.010035 | 0.080716 | 0.004956 | 0.621221 |
| *SLC44A4* | 0.001163 | 0.017147 | 0.040281 | 0.027308 | 0.945978 |
| *SLT2* | 0.000757 | 0.000016 | 0.058277 | 0.012012 | 0.924471 |
| *SMARC2* | 0.001385 | 0.000032 | 0.221828 | 0.003606 | 0.577531 |
| *SMG6* | 0.001006 | 0.000206 | 0.118683 | 0.006741 | 0.795141 |
| *SORBS2* | 0.001542 | 0.000002 | 0.086857 | 0.014967 | 0.843234 |
| *STAT4* | 0.000855 | 0.000342 | 0.164389 | 0.003650 | 0.701533 |
| *STK39* | 0.001351 | 0.000110 | 0.127867 | 0.007821 | 0.740031 |
| *STX8* | 0.001263 | 0.000002 | 0.116917 | 0.007698 | 0.712691 |
| *SUMO4* | 0.001489 | 0.125437 | 0.275543 | 0.004718 | 0.873064 |
| *SUSD1* | 0.000935 | 0.000174 | 0.110669 | 0.007229 | 0.855871 |
| *TCF19* | 0.001732 | 0.014951 | 0.027187 | 0.069886 | 1.096701 |
| *TENM4* | 0.001059 | 0.000002 | 0.133050 | 0.006013 | 0.755329 |
| *TFCP2L1* | 0.000901 | 0.001700 | 0.118498 | 0.005063 | 0.665846 |
| *TLR4* | 0.000639 | 0.030590 | 0.125253 | 0.003194 | 0.625970 |
| *TMEM132B* | 0.001117 | 0.000012 | 0.128899 | 0.006206 | 0.716416 |
| *TNF* | 0.000259 | 0.006762 | 0.013679 | 0.014621 | 0.771760 |
| *TNFAIP3* | 0.000742 | 0.046074 | 0.232158 | 0.001723 | 0.539226 |
| *TRIM31* | 0.002100 | 0.013805 | 0.055961 | 0.033952 | 0.904858 |
| *TTLL7* | 0.000688 | 0.002327 | 0.137268 | 0.004371 | 0.872325 |
| *UBAC2* | 0.000719 | 0.000636 | 0.170168 | 0.003526 | 0.834237 |
| *UBASH3B* | 0.001189 | 0.000110 | 0.114932 | 0.008701 | 0.841099 |
| *UBD* | 0.002341 | 0.049420 | 0.298478 | 0.003350 | 0.427116 |
| *ZNRD1* | 0.001321 | 0.011900 | 0.092085 | 0.009774 | 0.681345 |

**Supplemental Table 11.** Genes with highest quartile genetic differentiation parameter values with respect to African population for East Asian, and European 1K Genomes populations.

Only the highest quartile values are presented for clarity.

|  | **African-East Asian differentiation** | | | | **African-European differentiation** | | | |
| --- | --- | --- | --- | --- | --- | --- | --- | --- |
| **Gene** | **Dxy** | **Fst** | **Pi/Fst** | **Pi/Dxy** | **Dxy** | **Fst** | **Pi/Fst** | **Pi/Dxy** |
| *API5* |  | 0.259816218 |  |  |  | 0.173588795 |  |  |
| *ASB18* |  | 0.235803671 |  |  |  | 0.180449526 |  |  |
| *BTNL2* | 0.005921291 |  | 0.139040939 | 1.300392174 | 0.003401745 |  | 0.188291449 |  |
| *C6ORF10 (TSBP1)* | 0.002837655 |  |  |  |  |  |  |  |
| *C6ORF15* | 0.003864848 |  |  | 0.983220968 | 0.003266093 |  | 0.188681168 | 1.102234516 |
| *CCHCR1* |  |  | 0.056513964 |  | 0.002864173 |  | 0.193390552 | 1.012508718 |
| *CDH26* |  | 0.229933931 |  |  |  | 0.262582705 |  |  |
| *DHFRP2* | 0.006939587 |  | 0.142617171 |  | 0.007067305 |  | 0.482600785 | 1.00462617 |
| *EBF2* |  | 0.217973266 |  |  |  |  |  |  |
| *ERAP1* |  |  |  |  |  |  |  | 1.040678078 |
| *FUT2* |  | 0.359038352 |  |  |  |  |  |  |
| *GABBR1* |  |  |  | 1.057803914 |  |  |  |  |
| *GALT10* |  | 0.280904079 |  |  |  |  |  |  |
| *HCG27* | 0.004309209 |  | 0.069537481 | 0.997862876 | 0.003998085 |  | 0.358845411 | 1.025490924 |
| *HCG9* | 0.007516202 |  | 0.085012184 |  | 0.007599069 |  | 0.09977476 |  |
| *HERPUD2* | 0.004309209 |  | 0.069537481 | 0.997862876 | 0.003998085 |  | 0.358845411 | 1.025490924 |
| *HLA_B* | 0.016586943 |  | 0.448183816 | 1.000787179 | 0.016342352 |  | 0.612616157 | 1.0096466 |
| *HLA_DQ* | 0.035045702 |  | 1.712056034 | 0.990135681 | 0.036175883 |  | 2.24974912 | 1.02830939 |
| *HLA_F* |  |  | 0.051784543 |  |  |  |  |  |
| *HLA_G* | 0.006015653 |  | 0.171923902 |  | 0.006193034 |  | 0.188946431 |  |
| *HLAC* | 0.01841791 |  | 0.343648389 |  | 0.018984499 |  | 1.10927539 | 0.974479235 |
| *HLADQB1* | 0.038062288 |  | 0.840770787 | 1.014127159 | 0.035233771 |  | 3.68557176 | 1.016070626 |
| *HMP19* |  | 0.291472228 |  |  |  |  |  |  |
| *HNF4G* |  | 0.329218533 |  |  |  |  |  |  |
| *IL10* |  |  |  |  |  |  |  | 0.987202548 |
| *IL1A* |  | 0.33105719 |  |  |  |  |  | 0.973796208 |
| *IL6* |  | 0.315357355 |  |  |  |  |  |  |
| *LILRA1* |  |  |  |  |  | 0.202055579 |  |  |
| *LOC100129342* |  |  |  | 0.977036941 |  | 0.196364814 |  |  |
| *LOC285830* | 0.003478451 |  | 0.076082587 |  | 0.003417189 |  | 0.110926227 |  |
| *LYST* |  | 0.401947798 |  |  |  | 0.173625837 |  |  |
| *MICA* | 0.004603194 |  | 0.058356508 |  | 0.004379082 |  | 0.094878298 |  |
| *MSX2* |  | 0.392648927 |  |  |  | 0.245412479 |  |  |
| *MUC21* |  |  |  |  |  |  | 0.092737696 | 0.988121381 |
| *NAV2* |  |  |  |  |  | 0.170525014 |  |  |
| *OSR1* |  | 0.319798114 |  |  |  |  |  |  |
| *PLEKHB1* |  |  |  |  |  | 0.203640191 |  |  |
| *POU5F1* |  |  |  | 1.014923474 |  |  |  | 1.030750321 |
| *PSOR1C1* | 0.004039579 |  | 0.102960973 | 0.990202199 | 0.004252348 |  |  |  |
| *PTPN22* |  | 0.343023 |  |  |  |  |  | 1.003747 |
| *RALGAPA2* |  |  |  | 0.973250129 |  |  |  |  |
| *SEMA6D* |  |  |  |  |  | 0.1788275 |  |  |
| *SLC41A2* |  |  |  | 0.959933742 |  |  |  |  |
| *SMARC2* |  |  |  |  |  | 0.221827969 |  |  |
| *SUMO4* |  |  |  | 0.986953026 |  | 0.275542803 |  |  |
| *TCF19* |  |  |  | 0.976673455 |  |  |  | 1.096701152 |
| *TNFAIP3* |  | 0.33596092 |  |  |  | 0.232158083 |  |  |
| *UBAC2* |  |  |  |  |  | 0.17016835 |  |  |
| *UBD* |  |  |  |  |  | 0.298477524 |  |  |

Fst: fixation index, Dxy: average number of nucleotide substitutions per site between two populations, Hst: haplotype diversity based differentiation

**Supplemental Table 12.** Genes with lowest quartile genetic differentiation parameter values with respect to African population for East Asian, and European 1K Genomes populations.

Only the lowest quartile values are presented for clarity.

|  | **African- East Asian differentiation** | | | | **African-European differentiation** | | | |
| --- | --- | --- | --- | --- | --- | --- | --- | --- |
| **Gene** | **Dxy** | **Fst** | **Pi/Fst** | **Pi/Dxy** | **Dxy** | **Fst** | **Pi/Fst** | **Pi/Dxy** |
| *API5* | 0.00038164 |  | 0.000769775 | 0.52405105 | 0.000413077 |  | 0.001728222 |  |
| *BTNL2* |  |  |  |  |  | 0.01699493 |  |  |
| *C10ORF11 (LRMDA)* |  |  |  |  |  |  | 0.002992327 |  |
| *C6ORF15* |  |  |  |  |  | 0.019079806 |  |  |
| *C6ORF47* | 0.00048594 |  |  |  | 0.000420047 |  |  |  |
| *CCHCR1* |  | 0.042467381 |  |  |  | 0.014995562 |  |  |
| *CCR1* | 0.00033504 |  | 0.001222986 |  | 0.000422479 |  | 0.002254396 |  |
| *CDH26* |  |  | 0.001304723 | 0.414504764 |  |  | 0.001142497 | 0.419940629 |
| *CEP135* | 0.00053182 |  |  |  | 0.000558484 |  |  |  |
| *COL12A1* |  |  |  |  | 0.000522081 |  | 0.001565075 | 0.383082319 |
| *CTLA4* | 0.000346638 |  |  |  | 0.000321733 | 0.027420825 |  |  |
| *DHFRP2* |  | 0.044875382 |  |  |  | 0.014711953 |  |  |
| *DNMT3A* | 0.00052854 |  |  |  | 0.000572776 |  |  |  |
| *DTL* | 0.00059926 |  |  |  |  |  |  |  |
| *ERAP1* |  | 0.038666464 |  |  |  |  |  |  |
| *FUT2* |  |  | 0.001114087 | 0.210317018 |  |  |  |  |
| *GABBR1* |  | 0.028219117 |  |  | 0.000621382 | 0.008297016 |  |  |
| *GALT10* |  |  | 0.002491954 | 0.509455183 |  |  |  |  |
| *HCG27* |  |  |  |  |  | 0.011425533 |  |  |
| *HERPUD2* |  |  |  |  |  | 0.011425533 |  |  |
| *HLA_B* |  | 0.037038374 |  |  |  | 0.026933668 |  |  |
| *HLA_DQ* |  | 0.020268028 |  |  |  | 0.016535177 |  |  |
| *HLA_F* |  | 0.04248372 |  |  |  |  |  |  |
| *HLA_G* |  | 0.031990898 |  |  |  |  |  |  |
| *HLAC* |  |  |  |  |  | 0.016677554 |  |  |
| *HLADQB1* |  |  |  |  |  | 0.009713554 |  |  |
| *HMP19* |  |  | 0.000686172 | 0.30158349 |  |  | 0.002438571 | 0.583914759 |
| *IFNG* | 0.00041096 |  |  |  | 0.000481334 |  |  |  |
| *IL1A* |  |  | 0.002114438 |  |  |  |  |  |
| *IL6* |  |  | 0.000951302 | 0.32255781 |  |  |  |  |
| *KLRK1* |  |  |  |  |  |  |  | 0.590926657 |
| *LILRA1* |  |  |  | 0.514290003 |  |  |  | 0.551510331 |
| *LOC100129342* |  |  |  |  |  |  | 0.003564793 |  |
| *LOC285830* |  | 0.042059558 |  |  |  |  |  |  |
| *LTN1* |  |  |  |  | 0.000625893 |  |  |  |
| *LYST* |  |  | 0.000497577 | 0.240282246 |  |  | 0.002303805 | 0.518919173 |
| *MOG* |  |  |  | 0.507002463 |  |  |  |  |
| *MSX2* |  |  | 0.000764041 | 0.227997021 |  |  | 0.002444863 | 0.478696557 |
| *MUC21* |  |  |  |  |  | 0.017252963 |  |  |
| *NOD2* | 0.0005754 |  | 0.002098611 | 0.52137946 |  |  |  |  |
| *OSR1* | 0.0003497 |  | 0.000312697 | 0.285959471 | 0.000331607 |  | 0.002260214 | 0.603123994 |
| *PLEKHB1* |  |  |  |  |  |  |  | 0.555559952 |
| *PSMD14* | 0.00033656 |  |  |  | 0.000329016 |  |  |  |
| *PSOR1C1* |  | 0.038849672 |  |  |  |  |  |  |
| *PTPN22* |  |  | 0.001242394 |  | 0.000544698 |  |  |  |
| *RALGAPA2* | 0.00041099 |  |  |  | 0.000339199 |  | 0.002559187 | 0.589625155 |
| *RNF39* |  | 0.044244233 |  |  |  |  |  |  |
| *SLC43A3* |  |  |  | 0.497631377 |  |  |  | 0.621221301 |
| *SLC44A4* |  | 0.041401632 |  |  |  |  |  |  |
| *SMARC2* |  |  |  |  |  |  |  | 0.577530778 |
| *TCF19* |  |  |  |  |  | 0.027187253 |  |  |
| *TLR4* | 0.00058999 |  | 0.001926148 | 0.508484303 |  |  | 0.003193536 | 0.625969814 |
| *TNF* | 0.00023183 | 0.037287858 |  |  | 0.000259148 | 0.013678583 |  |  |
| *TNFAIP3* |  |  | 0.000595307 | 0.267630519 |  |  | 0.001722964 | 0.539226222 |
| *TTLL7* | 0.0005391 | 0.039030407 |  |  |  |  |  |  |
| *UBAC2* |  |  |  |  |  |  | 0.00352592 |  |
| *UBD* |  |  |  |  |  |  | 0.003350336 | 0.427115547 |
| *ZNRD1* |  | 0.043799185 |  |  |  |  |  |  |

Fst: fixation index, Dxy: average number of nucleotide substitutions per site between two populations, Hst: haplotype diversity based differentiation

**Supplemental Table 13.** Comparison of BD associated SNP Fst vs. Gene Fst values. Fst values estimated comparing East Asian vs. African populations.

1. **SNPs with Fst values greater than their respective Gene Fst estimates**

| **SNP** | **Gene** | **SNP Fst** | **Gene Fst** |
| --- | --- | --- | --- |
| rs1343151 | IL23R | 0.63 | 0.15 |
| rs4242425 | EBF2 | 0.51 | 0.22 |
| rs610604 | TNFAIP3 | 0.51 | 0.34 |
| rs7561555 | ASB18 | 0.51 | 0.24 |
| rs4570167 | EBF2 | 0.50 | 0.22 |
| rs12141431 | IL23R, IL12RB2 | 0.49 | 0.15 |
| rs681343 | FUT2 | 0.45 | 0.36 |
| rs7758496 | SAMD3(TMEM200A) | 0.43 | 0.20 |
| rs4897380 | SAMD3 | 0.42 | 0.20 |
| rs103294 | LILRA1 | 0.40 | 0.19 |
| rs798887 | LILRB1 | 0.39 | 0.11 |
| rs9494885 | TNFAIP3 | 0.39 | 0.34 |
| rs1472224 | DTL | 0.36 | 0.09 |
| rs749240 | SMG6 | 0.34 | 0.19 |
| rs6803980 | CCR3 | 0.34 | 0.08 |
| rs9483115 | SAMD3(TMEM200A) | 0.33 | 0.20 |
| rs724324 | SAMD3(TMEM200A) | 0.32 | 0.20 |
| rs4936742 | UBASH3B | 0.31 | 0.12 |
| rs9990343 | CCR3 | 0.30 | 0.08 |
| rs4640857 | COL12A1 | 0.30 | 0.08 |
| rs7033529 | SMARCA2 | 0.29 | 0.13 |
| rs1615251 | LOC285830  ( HLA-F antisense RNA1) | 0.28 | 0.04 |
| rs4660590 | HIVEP3 | 0.28 | 0.19 |
| rs817277 | CDH26 | 0.28 | 0.23 |
| rs2373156 | CCR3 | 0.28 | 0.08 |
| rs7651539 | CCR3 | 0.28 | 0.08 |
| rs817283 | CDH26 | 0.26 | 0.23 |
| rs6692084 | DEPDC1 | 0.25 | 0.15 |
| rs7616215 | CCR1 | 0.24 | 0.16 |
| rs2782932 | SUSD1 | 0.23 | 0.13 |
| rs4852547 | CTNNA2 | 0.22 | 0.14 |
| rs3129045 | MOG | 0.22 | 0.11 |
| rs237024 | SUMO4 | 0.21 | 0.08 |
| rs7631551 | CCR1 | 0.20 | 0.16 |
| rs12119179 | IL23R, IL12RB2 | 0.19 | 0.15 |
| rs7649764 | CCR3 | 0.19 | 0.08 |
| rs9554573 | UBAC2 | 0.19 | 0.10 |
| rs11209033 | IL23R, IL12RB2 | 0.18 | 0.15 |
| rs1737031 | LOC285830 ( HLA-F antisense RNA1) | 0.18 | 0.04 |
| rs4493590 | SORBS2 | 0.18 | 0.13 |
| rs549630 | SLC43A3 | 0.17 | 0.12 |
| rs9261389 | TRIM31 | 0.17 | 0.09 |
| rs2593082 | CEP135 | 0.17 | 0.08 |
| rs11206377 | LOC100129342 | 0.17 | 0.14 |
| rs1436321 | OVCH1 | 0.16 | 0.12 |
| rs574710 | C6orf10 | 0.15 | 0.07 |
| rs1736951 | HLA-G | 0.15 | 0.03 |
| rs13435197 | SLIT2 | 0.15 | 0.12 |
| rs544358 | C6orf10 | 0.13 | 0.07 |
| rs539703 | C6orf10 | 0.13 | 0.07 |
| rs4959093 | C6orf10 | 0.13 | 0.07 |
| rs926591 | C6orf10 | 0.13 | 0.07 |
| rs13075270 | CCR3 | 0.13 | 0.08 |
| rs2523408 | HLA-G | 0.12 | 0.03 |
| rs2530710 | MUC21 | 0.12 | 0.05 |
| rs7999348 | UBAC2 | 0.12 | 0.10 |
| rs9517701 | UBAC2 | 0.12 | 0.10 |
| rs2061634 | CCDC180 | 0.11 | 0.09 |
| rs7332161 | UBAC2 | 0.11 | 0.10 |
| rs727263 | UBAC2 | 0.11 | 0.10 |
| rs9513584 | UBAC2 | 0.11 | 0.10 |
| rs6491493 | UBAC2 | 0.11 | 0.10 |
| rs4959053 | PSORS1C1 | 0.11 | 0.04 |
| rs9263804 | POU5F1 | 0.10 | 0.06 |
| rs3130501 | POU5F1 | 0.10 | 0.06 |
| rs3132524 | POU5F1 | 0.10 | 0.06 |
| rs2073723 | TCF19 | 0.10 | 0.05 |
| rs2523467 | MICA | 0.10 | 0.07 |
| rs11763983 | HERPUD2 | 0.08 | 0.06 |
| rs3130944 | HCG27 | 0.08 | 0.06 |
| rs1799724 | TNFα | 0.08 | 0.04 |
| rs1633041 | LOC285830  ( HLA-F antisense RNA1) | 0.06 | 0.04 |
| rs1632973 | HLA-G | 0.06 | 0.03 |
| rs1736963 | HLA-G | 0.06 | 0.03 |
| rs1611172 | HLA-G | 0.06 | 0.03 |
| rs753544 | HLA-G | 0.06 | 0.03 |
| rs1077433 | HLA-G | 0.06 | 0.03 |
| rs885940 | LOC285830 ( HLA-F antisense RNA1) | 0.06 | 0.04 |
| rs1610637 | LOC285830 ( HLA-F antisense RNA1) | 0.06 | 0.04 |
| rs1633002 | HLA-G | 0.06 | 0.03 |
| rs9266406 | HLA-B | 0.05 | 0.04 |
| rs9266409 | HLA-B | 0.05 | 0.04 |
| rs6910516 | HLA-B | 0.05 | 0.04 |
| rs29273 | GABBR1 | 0.05 | 0.03 |
| rs407238 | HLA-G | 0.04 | 0.03 |

1. **SNPs with Fst values less than their respective Gene Fst estimates**

| **SNP** | **Gene** | **SNP Fst** | **Gene Fst** |
| --- | --- | --- | --- |
| rs7753873 | TNFAIP3 | 0.32 | 0.34 |
| rs7354999 | LYST/NID1 | 0.28 | 0.40 |
| rs574750 | GALNT10 | 0.20 | 0.28 |
| rs10516130 | MSX2 | 0.20 | 0.39 |
| rs4141940 | SAMD3(TMEM200A) | 0.20 | 0.20 |
| rs16937370 | API5 | 0.19 | 0.26 |
| rs899276 | SAMD3(TMEM200A) | 0.18 | 0.20 |
| rs924080 | IL23R, IL12RB2 | 0.14 | 0.15 |
| rs17434565 | C10orf11 | 0.13 | 0.15 |
| rs1518111 | IL-10 | 0.11 | 0.17 |
| rs1800871 | IL-10 | 0.11 | 0.17 |
| rs1265048 | C6orf15 | 0.10 | 0.14 |
| rs3769393 | STK39 | 0.10 | 0.11 |
| rs2707110 | NAV2 | 0.10 | 0.16 |
| rs1554286 | IL-10 | 0.10 | 0.17 |
| rs9554581 | UBAC2 | 0.10 | 0.10 |
| rs984477 | UBAC2 | 0.10 | 0.10 |
| rs11069357 | UBAC2 | 0.09 | 0.10 |
| rs3825427 | UBAC2 | 0.09 | 0.10 |
| rs4435061 | TMEM132B | 0.09 | 0.13 |
| rs9517644 | UBAC2 | 0.09 | 0.10 |
| rs912130 | UBAC2 | 0.09 | 0.10 |
| rs10864912 | PAX8 | 0.09 | 0.13 |
| rs2390639 | STK39 | 0.09 | 0.11 |
| rs873764 | NAV2 | 0.08 | 0.16 |
| rs12134670 | DEPDC1 | 0.08 | 0.15 |
| rs10833804 | GAS2 | 0.08 | 0.20 |
| rs591804 | PLEKHB1 | 0.06 | 0.21 |
| rs4986790 | TLR4 | 0.06 | 0.16 |
| rs1323076 | C10orf11 | 0.06 | 0.15 |
| rs10266069 | GIMAP2 | 0.06 | 0.12 |
| rs10499194 | TNFAIP3 | 0.06 | 0.34 |
| rs11123169 | PAX8 | 0.06 | 0.13 |
| rs3094584 | MICA | 0.06 | 0.07 |
| rs1549332 | STX8 | 0.06 | 0.18 |
| rs2731031 | SLC41A2 | 0.05 | 0.06 |
| rs2100766 | ATP8A1 | 0.05 | 0.14 |
| rs1969624 | SACM1L | 0.05 | 0.09 |
| rs17006292 | TFCP2L1 | 0.05 | 0.08 |
| rs13092160 | CCR3 | 0.05 | 0.08 |
| rs17282391 | CCR1 | 0.04 | 0.16 |
| rs2980221 | HNF4G | 0.04 | 0.33 |
| rs2156215 | TENM4(ODZ4) | 0.04 | 0.18 |
| rs7761068 | DHFRP2 | 0.04 | 0.04 |
| rs1961261 | KCNK9 | 0.04 | 0.13 |
| rs1634717 | MUC21 | 0.04 | 0.05 |
| rs1632854 | MUC21 | 0.04 | 0.05 |
| rs1542755 | CCR3 | 0.03 | 0.08 |
| rs17375018 | IL23R | 0.03 | 0.15 |
| rs7574070 | STAT4 | 0.03 | 0.12 |
| rs2286900 | GIMAP1 | 0.03 | 0.10 |
| rs897200 | STAT4 | 0.03 | 0.12 |
| rs6926792 | HCG9 | 0.03 | 0.07 |
| rs10510749 | CCR1 | 0.03 | 0.16 |
| rs13084057 | CCR1 | 0.03 | 0.16 |
| rs13067058 | CCR3 | 0.03 | 0.08 |
| rs6911737 | HCG9 | 0.03 | 0.07 |
| rs6931776 | HCG9 | 0.03 | 0.07 |
| rs2074482 | PPP1R11 | 0.03 | 0.07 |
| rs9261189 | ZNRD1 | 0.03 | 0.04 |
| rs3869068 | ZNRD1 | 0.03 | 0.04 |
| rs10846924 | TMEM132B | 0.03 | 0.13 |
| rs6744214 | PSMD14 | 0.03 | 0.06 |
| rs9469615 | LOC100132252 | 0.03 | 0.11 |
| rs6733456 | PSMD14 | 0.03 | 0.06 |
| rs2892976 | UBAC2 | 0.03 | 0.10 |
| rs2617170 | KLRC4 | 0.03 | 0.07 |
| rs11965547 | SLC44A4 | 0.03 | 0.04 |
| rs10846917 | TMEM132B | 0.03 | 0.13 |
| rs12589991 | GALNTL1 | 0.02 | 0.15 |
| rs17562982 | SGPP2 | 0.02 | 0.15 |
| rs1495965 | IL23R, IL12RB2 | 0.02 | 0.15 |
| rs2242655 | C6orf47 | 0.02 | 0.12 |
| rs2077102 | BAG6(BAT3) | 0.02 | 0.11 |
| rs11163772 | TTLL7 | 0.02 | 0.04 |
| rs9517668 | UBAC2 | 0.02 | 0.10 |
| rs3905495 | HLA-C | 0.01 | 0.05 |
| rs2832137 | LTN1(RNF160) | 0.01 | 0.08 |
| rs11862324 | PMFBP1 | 0.01 | 0.17 |
| rs7572482 | STAT4 | 0.01 | 0.12 |
| rs9258205 | LOC285830  ( HLA-F antisense RNA1) | 0.01 | 0.04 |
| rs2844673 | MUC21 | 0.01 | 0.05 |
| rs317711 | CPVL | 0.01 | 0.18 |
| rs1909704 | HMP19 | 0.01 | 0.29 |
| rs10256482 | GIMAP2 | 0.01 | 0.12 |
| rs1608157 | GIMAP4 | 0.01 | 0.07 |
| rs1799964 | TNFα | 0.01 | 0.04 |
| rs1916012 | GIMAP4 | 0.01 | 0.07 |
| rs4666492 | OSR1 | 0.01 | 0.32 |
| rs2844845 | LOC285830  ( HLA-F antisense RNA1) | 0.00 | 0.04 |
| rs2517446 | MUC21 | 0.00 | 0.05 |
| rs2517411 | MUC21 | 0.00 | 0.05 |
| rs2252925 | MUC21 | 0.00 | 0.05 |
| rs2252926 | MUC21 | 0.00 | 0.05 |
| rs2523915 | MUC21 | 0.00 | 0.05 |
| rs6082210 | RALGAPA2 | 0.00 | 0.05 |
| rs4986791 | TLR4 | 0.00 | 0.16 |
| rs2073716 | CCHCR1 | 0.00 | 0.04 |
| rs2076530 | BTNL2 | 0.00 | 0.06 |
| rs2240063 | CCHCR1 | 0.00 | 0.04 |
| rs9260954 | HCG9 | 0.00 | 0.07 |
| rs9272346 | HLA-DQA1 | 0.00 | 0.02 |
| rs11209026 | IL23R | 0.00 | 0.15 |
| rs872837 | LOC107984355 | 0.00 | 0.19 |
| rs134006 | MN1 | 0.00 | 0.07 |
| rs2066847 | NOD2 | 0.00 | 0.14 |
| rs9261317 | RNF39 | 0.00 | 0.04 |
| rs6923832 | TRIM31 | 0.00 | 0.09 |
| rs1522596 | GIMAP4 | 0.00 | 0.07 |
| rs2066844 | NOD2 | 0.00 | 0.14 |
| rs9261265 | ZNRD1 | 0.00 | 0.04 |
| rs12194547 | C6orf85(LOC100507336) | 0.00 | 0.13 |
| rs11248047 | CPLX1 | 0.00 | 0.15 |
| rs1465825 | DNMT3A | 0.00 | 0.06 |
| rs17482078 | ERAP1 | 0.00 | 0.04 |
| rs6457617 | HLA-DQB1 | 0.00 | 0.05 |
| rs3116788 | HLA-F | 0.00 | 0.04 |
| rs1610584 | HLA-F | 0.00 | 0.04 |
| rs1610585 | HLA-F | 0.00 | 0.04 |
| rs1610593 | HLA-F | 0.00 | 0.04 |
| rs1611356 | HLA-F | 0.00 | 0.04 |
| rs1627465 | HLA-F | 0.00 | 0.04 |
| rs17810546 | IL12A | 0.00 | 0.18 |
| rs2617151 | KLRK1 | 0.00 | 0.15 |
| rs2523386 | LOC285830  ( HLA-F antisense RNA1) | 0.00 | 0.04 |
| rs9501063 | POU5F1 | 0.00 | 0.06 |
| rs470151 | SEMA6D | 0.00 | 0.16 |
| rs361525 | TNFα | 0.00 | 0.04 |
| rs17575643 | UBAC2 | 0.00 | 0.10 |
| rs6933331 | UBD | 0.00 | 0.16 |
| rs3025657 | UBD | 0.00 | 0.16 |

**Supplemental Table 14.** Cross population extended haplotype homozygosity (XP-EHH), and Integrated haplotype score (iHS) analyses of BD associated genes. Pairwise XP-EHH analyses compares YRI, CEU, and CHB populations. iHS scores are calculated within AFR, EAS, EUR, and SAS subpopulations.

Highest values from 10kb sliding window analyses are presented.

|  | **XP-EHH** | | | **iHS** | | | | | | | | | | | | | | | | | | | | | |
| --- | --- | --- | --- | --- | --- | --- | --- | --- | --- | --- | --- | --- | --- | --- | --- | --- | --- | --- | --- | --- | --- | --- | --- | --- | --- |
| **Gene** | **CEU-**  **YRI** | **CHB-CEU** | **CHB-**  **YRI** | **ACB-AFR** | **ASW-**  **AFR** | **ESN-AFR** | **GWD-**  **AFR** | **LWK-**  **AFR** | **MSL-AFR** | **YRI**  **-AFR** | **CDX-EAS** | **CHB-EAS** | **CHS-EAS** | **JPT-EAS** | **KHV-**  **EAS** | **CEU-EUR** | **FIN**  **-**  **EUR** | **GBR-EUR** | **IBS**  **-EUR** | **TSI**  **-EUR** | **BEB-**  **SAS** | **GIH-**  **SAS** | **ITU**  **-**  **SAS** | **PJL**  **-**  **SAS** | **STU-**  **SAS** |
| ABCB5 | 0.31 | 0.24 | 0.54 | 0.87 | 0.57 | 0.87 | 0.76 | 1.29 | 0.77 | 0.89 | 1.12 | 0.67 | 0.82 | 1.21 | 0.68 | 0.47 | 0.47 | 0.42 | 0.48 | 0.52 | 0.47 | 0.41 | 0.54 | 0.46 | 0.31 |
| API5 | 0.14 | 0.48 | 0.30 | 0.61 | 0.95 | 0.54 | 0.66 | 0.52 | 0.42 | 0.66 | 1.78 | 1.59 | 1.46 | 0.85 | 1.07 | 1.53 | 1.14 | 1.74 | **2.09** | 1.00 | 0.92 | 0.88 | 1.08 | 1.50 | 0.99 |
| ASB18 | 1.40 | 0.20 | 1.52 | 0.31 | 0.51 | 0.43 | 0.75 | 0.42 | 0.89 | 0.34 | 1.58 | 1.41 | 1.41 | 1.48 | 1.71 | 1.15 | 1.56 | **2.00** | **2.31** | **2.03** | 0.95 | 1.32 | 1.20 | 1.32 | 0.66 |
| ATP8A1 | 0.29 | 0.19 | 0.39 | 0.45 | 0.34 | 0.32 | 0.49 | 0.38 | 0.35 | 0.31 | 0.72 | 0.76 | 0.82 | 0.74 | 0.89 | 0.67 | 0.72 | 0.69 | 0.54 | 0.85 | 0.64 | 0.53 | 0.63 | 0.47 | 0.66 |
| BAG6 | 0.13 | 0.08 | 0.15 | 0.37 | 0.74 | 0.99 | 0.83 | 0.75 | 0.56 | 0.43 | 0.77 | 0.79 | 0.72 | 0.69 | 1.19 | 0.79 | 0.90 | 0.96 | 0.82 | 0.97 | 1.53 | 1.24 | 1.29 | 1.09 | 1.60 |
| BTNL2 | - | - | - | - | - | - | - | - | - | - | - | - | - | - | - | - | - | - | - | - | - | - | - | - | - |
| C10ORF11 | 0.93 | 0.41 | 0.59 | 0.78 | 0.58 | 0.98 | 0.93 | 1.50 | 0.80 | 0.82 | 1.31 | 0.78 | 1.23 | 1.35 | 1.54 | 0.82 | 0.70 | 0.66 | 0.48 | 0.64 | 0.76 | 1.05 | 0.84 | 0.50 | 0.81 |
| C6ORF10* | 0.66 | 0.48 | 0.19 | 0.32 | 0.40 | 0.30 | 0.52 | 0.51 | 0.31 | 0.30 | 1.85 | 1.24 | 1.28 | 0.83 | 1.56 | 0.79 | 0.70 | 0.74 | 1.16 | 1.28 | 1.37 | 0.79 | 0.97 | 1.58 | 1.44 |
| C6ORF15 | 0.31 | 0.24 | 0.11 | 0.60 | 0.69 | 1.05 | 0.91 | 1.03 | 0.90 | 0.50 | 0.94 | 0.62 | 0.53 | 0.42 | 0.77 | 0.50 | 0.37 | 0.55 | 0.60 | 0.66 | 0.72 | 0.82 | 0.66 | 0.67 | 0.96 |
| C6ORF47 | 0.27 | 0.08 | 0.32 | 1.02 | 0.99 | 1.59 | 1.27 | 0.84 | 0.85 | 1.01 | 0.69 | 0.72 | 1.14 | 0.78 | 1.47 | 1.37 | 1.63 | 1.67 | 0.98 | 1.88 | 1.61 | 1.57 | 1.34 | 0.96 | 1.64 |
| C6ORF85 | **2.23** | 0.79 | 1.25 | 0.72 | 1.09 | 0.61 | 0.70 | 0.72 | 0.84 | 0.73 | 0.64 | 0.59 | 0.36 | 1.03 | 0.40 | 1.87 | 1.63 | **2.03** | **2.13** | 1.82 | 1.32 | 1.65 | 1.05 | 1.52 | 0.95 |
| CCDC180 | 0.53 | 0.08 | 0.57 | 0.72 | 0.68 | 0.88 | 0.84 | 0.71 | 0.82 | 0.62 | 0.93 | 0.54 | 0.60 | 0.38 | 0.86 | 0.77 | 0.80 | 0.71 | 0.63 | 0.92 | 0.32 | 0.50 | 0.34 | 0.51 | 0.67 |
| CCHCR1 | - | - | - | - | - | - | - | - | - | - | - | - | - | - | - | - | - | - | - | - | - | - | - | - | - |
| CCR1 | 0.19 | 0.30 | 0.36 | 1.96 | 1.20 | **2.04** | **2.86** | 1.42 | **2.26** | **2.63** | 0.00 | 0.68 | 0.00 | 0.00 | 0.00 | 0.69 | 0.87 | 0.74 | 0.60 | 0.54 | 0.72 | 0.50 | 0.49 | 0.62 | 0.41 |
| CCR3 | 0.04 | 0.55 | 0.41 | **2.91** | 1.82 | **2.90** | **3.50** | **2.57** | **4.12** | **4.24** | 0.00 | 0.41 | 0.00 | 0.00 | 0.00 | 0.56 | 0.31 | 0.50 | 0.73 | 0.60 | 0.27 | 0.75 | 0.67 | 1.04 | 0.23 |
| CDH26 | 0.47 | 0.37 | 0.93 | 0.69 | 0.87 | 0.77 | 0.88 | 0.72 | 0.70 | 0.62 | - | - | 0.71 | - | - | 0.72 | 0.53 | 0.62 | 0.58 | 0.75 | 0.70 | 1.02 | 0.65 | 0.95 | 0.64 |
| CEP135 | 0.25 | 0.30 | 0.58 | 0.52 | 0.60 | 0.59 | 0.65 | 0.79 | 0.42 | 0.50 | 1.35 | 1.05 | 1.13 | 0.91 | 1.22 | 0.72 | 1.09 | 0.72 | 0.95 | 0.66 | 0.92 | 0.93 | 0.86 | 0.95 | 0.88 |
| COL12A1 | 0.92 | 0.22 | 0.72 | 0.61 | 0.81 | 0.52 | 0.42 | 0.55 | 0.59 | 0.55 | 0.52 | 0.60 | 0.47 | 0.90 | 0.67 | 0.44 | 0.00 | 0.58 | 0.43 | 0.28 | 0.52 | 0.38 | 0.59 | 0.87 | 0.85 |
| CPLX1 | 0.16 | 0.09 | 0.08 | 0.88 | 0.55 | 0.86 | 1.01 | 1.01 | 1.19 | 0.79 | 0.49 | 0.66 | 0.74 | 0.60 | 0.76 | 0.55 | 0.51 | 0.46 | 0.46 | 0.63 | 0.48 | 0.50 | 0.44 | 0.50 | 0.43 |
| CPVL | 0.94 | 0.34 | 1.28 | 1.08 | 0.51 | 0.96 | 0.57 | 0.88 | 0.66 | 0.70 | 1.32 | 1.08 | 0.86 | 1.08 | 0.66 | 0.63 | 0.48 | 0.62 | 0.67 | 0.73 | 0.93 | 0.79 | 0.92 | 1.07 | 0.84 |
| CTNNA2 | 0.81 | 0.79 | **1.59** | 0.67 | 0.67 | 0.47 | 0.58 | 0.41 | 0.47 | 0.66 | **1.60** | **1.82** | **2.47** | **2.23** | **2.11** | 0.53 | 1.52 | 0.82 | 0.82 | 0.70 | 0.74 | 0.94 | 0.81 | 0.37 | 0.41 |
| DEPDC1 | 0.54 | 0.41 | 1.12 | 0.48 | 0.74 | 0.88 | 0.76 | 0.50 | 0.90 | 0.76 | 0.43 | 0.53 | 0.38 | 0.83 | 0.71 | 1.04 | 0.95 | 0.99 | 1.13 | 1.51 | 0.48 | 0.41 | 0.33 | 0.23 | 0.51 |
| DHFRP2 | - | - | - | - | - | - | - | - | - | - | - | - | - | - | - | - | - | - | - | - | - | - | - | - | - |
| DNMT3A | 0.33 | 0.09 | 0.43 | 0.67 | 0.53 | 0.67 | 0.51 | 0.62 | 0.39 | 1.03 | 0.84 | 0.76 | 0.30 | 0.83 | 0.37 | 0.68 | 0.64 | 0.92 | 1.02 | 0.67 | 0.67 | 0.66 | 1.06 | 0.71 | 0.44 |
| DTL* | 0.08 | 0.35 | 0.43 | 0.94 | 1.05 | 0.51 | 0.83 | 1.42 | 0.77 | 0.94 | **1.77** | 1.01 | **2.29** | **1.73** | **1.78** | 0.40 | 0.30 | 0.67 | 0.49 | 0.37 | 0.39 | 0.77 | 0.52 | 0.68 | 0.73 |
| EBF2* | 0.58 | 0.94 | **1.63** | 0.46 | 0.42 | 0.41 | 0.58 | 0.35 | 0.38 | 0.42 | **1.93** | **2.30** | **2.40** | **1.89** | **1.98** | 0.71 | 0.54 | 0.68 | 0.70 | 0.57 | 0.62 | 0.54 | 0.64 | 0.65 | 0.58 |
| ERAP1* | 0.08 | 0.38 | 0.54 | 0.94 | 0.68 | 0.62 | 1.11 | 0.47 | 1.13 | 0.88 | 1.25 | 1.16 | 1.04 | 0.63 | 1.18 | 0.61 | 0.49 | 0.56 | 0.54 | 0.65 | 0.77 | 0.97 | 0.74 | 1.01 | 0.90 |
| FUT2* | 0.60 | 0.23 | 0.89 | 0.79 | 0.53 | 0.44 | 0.79 | 0.76 | 0.33 | 0.66 | 0.22 | 0.30 | 0.46 | 1.00 | 0.79 | 1.19 | 0.83 | 0.97 | 0.76 | 1.06 | 0.39 | 0.95 | 0.58 | 0.94 | 0.46 |
| GABBR1* | 0.17 | 0.12 | 0.16 | 0.30 | 0.37 | 0.43 | 0.24 | 0.44 | 0.49 | 0.36 | 0.67 | 0.76 | 0.87 | 0.95 | 0.81 | 0.77 | 0.65 | 0.76 | 1.00 | 0.75 | 1.03 | 0.63 | 0.49 | 0.86 | 0.90 |
| GALNT1 | 1.07 | 0.23 | 0.98 | 0.45 | 0.67 | 1.22 | 0.84 | 0.79 | 0.55 | 0.63 | 0.82 | 1.11 | 1.66 | 1.42 | 0.83 | 0.67 | 0.44 | 0.57 | 0.10 | 0.73 | 0.19 | 1.41 | 0.82 | 1.02 | 0.57 |
| GALNT10* | 0.12 | 1.23 | 1.18 | 0.86 | 0.93 | 1.33 | 0.70 | 1.30 | 1.22 | 1.33 | **2.09** | **1.99** | **2.09** | 1.44 | **2.00** | 0.67 | 0.71 | 0.42 | 0.85 | 0.75 | 0.84 | 0.80 | 0.68 | 0.53 | 0.67 |
| GAS2 | 1.44 | 0.41 | **1.95** | 0.45 | 0.83 | 0.41 | 0.60 | 0.39 | 0.43 | 0.66 | 1.76 | 1.47 | 0.90 | 1.55 | 1.29 | 1.62 | 1.33 | 1.52 | 1.68 | 1.40 | 1.36 | 1.62 | 1.55 | 1.39 | 1.59 |
| GIMAP1 | 1.38 | 0.19 | 1.20 | 0.58 | 0.35 | 0.37 | 0.39 | 0.47 | 0.49 | 0.53 | 0.79 | 0.58 | 0.83 | 0.69 | 0.59 | 0.94 | 0.85 | 1.09 | 1.08 | 1.26 | 0.72 | 0.82 | 0.71 | 0.68 | 0.83 |
| GIMAP2 | 0.27 | 0.04 | 0.26 | 0.43 | 0.39 | 0.43 | 0.43 | 0.50 | 0.53 | 0.42 | 0.61 | 0.57 | 0.55 | 0.61 | 0.74 | 0.56 | 0.74 | 0.74 | 0.76 | 0.70 | 0.74 | 0.87 | 0.78 | 0.71 | 0.90 |
| GIMAP4 | 0.21 | 0.06 | 0.16 | 0.46 | 0.71 | 0.51 | 0.52 | 0.64 | 0.61 | 0.57 | 0.97 | 0.79 | 0.93 | 0.86 | 0.98 | 1.08 | 1.29 | 1.43 | 1.56 | 1.53 | 1.13 | 1.19 | 1.27 | 1.04 | 1.18 |
| HCG27 | 0.23 | 0.28 | 0.06 | 0.45 | 0.74 | 0.57 | 0.54 | 0.55 | 0.66 | 0.46 | 0.97 | 1.05 | 0.70 | 1.09 | 1.38 | 0.99 | 1.00 | 0.98 | 1.47 | 1.41 | 0.99 | 0.86 | 1.07 | 1.11 | 1.67 |
| HCG9 | - | - | - | - | - | - | - | - | - | - | - | - | - | - | - | - | - | - | - | - | - | - | - | - | - |
| HERPUD2 | 1.09 | 0.51 | 0.65 | 0.94 | 0.54 | 0.67 | 0.57 | 0.39 | 0.70 | 0.91 | 0.88 | 0.90 | 0.93 | 0.90 | 0.65 | 0.59 | 0.88 | 0.65 | 0.97 | 0.68 | 0.38 | 0.72 | 0.89 | 1.26 | 1.10 |
| HIVEP3 | 1.03 | 0.15 | 1.12 | 0.82 | 0.82 | 1.03 | 0.92 | 0.78 | 0.70 | 0.87 | 0.80 | 0.66 | 1.26 | 0.91 | 0.69 | 0.41 | 0.62 | 0.52 | 0.39 | 0.57 | 0.44 | 0.66 | 0.55 | 0.46 | 0.55 |
| HLA-B | 0.08 | 0.05 | 0.07 | 0.97 | 1.24 | 1.14 | 1.68 | 1.00 | 0.79 | 0.98 | **3.17** | **3.04** | **2.24** | **2.74** | **3.10** | **3.65** | **1.98** | **2.66** | **2.31** | **2.15** | **2.53** | **2.02** | 1.48 | **2.22** | 1.72 |
| HLA-DQA1 | 0.29 | 0.22 | 0.40 | 1.36 | 1.00 | 1.32 | 0.79 | 1.38 | 0.76 | 1.92 | 1.43 | 1.25 | 1.61 | 1.72 | 1.19 | **2.27** | **1.64** | **2.10** | **1.83** | **2.05** | 1.43 | 1.37 | 0.97 | 1.38 | **2.00** |
| HLA-F* | 0.43 | 0.10 | 0.40 | 0.37 | 0.52 | 0.43 | 0.34 | 0.41 | 0.54 | 0.60 | 0.26 | 0.53 | 0.39 | 0.86 | 0.64 | 0.53 | 1.03 | 0.53 | 0.55 | 0.72 | 0.77 | 0.90 | 0.80 | 0.52 | 0.85 |
| HLA-G* | 0.65 | 0.03 | 0.63 | 1.53 | 1.22 | 1.57 | 1.55 | 1.49 | 1.63 | 1.46 | 0.74 | **2.43** | **1.74** | **2.11** | **2.60** | 1.10 | 0.74 | 0.70 | 1.31 | 1.81 | **2.36** | **1.80** | **2.31** | **1.61** | **1.78** |
| HLA-C | - | - | - | - | - | - | - | - | - | - | - | - | - | - | - | - | - | - | - | - | - | - | - | - | - |
| HLA-DQB1 | 0.29 | 0.22 | 0.40 | 1.36 | 1.00 | 1.32 | 0.79 | 1.38 | 0.76 | 1.92 | 1.43 | 1.25 | 1.61 | 1.72 | 1.19 | **2.27** | **1.64** | **2.10** | **1.83** | **2.05** | 1.43 | 1.37 | 0.97 | 1.38 | **2.00** |
| HMP19* | 1.19 | 0.06 | 1.10 | 0.60 | 0.72 | 0.75 | 1.06 | 0.37 | 0.93 | 0.33 | 0.65 | 0.96 | 1.79 | **1.97** | - | 1.00 | 1.18 | 0.84 | 0.97 | 1.37 | 0.24 | 0.43 | 0.26 | 0.76 | 0.30 |
| HNF4G* | 0.10 | 0.55 | 0.59 | 0.56 | 1.14 | 0.70 | 0.61 | 0.62 | 0.58 | 0.98 | 1.18 | 1.00 | 0.89 | 1.16 | 1.34 | 0.75 | 0.67 | 1.21 | 1.14 | 1.05 | 1.33 | 0.74 | 1.20 | 1.31 | 0.71 |
| IFNG | - | - | - | - | - | - | - | - | - | - | - | - | - | - | - | - | - | - | - | - | - | - | - | - | - |
| IL10 | 0.13 | 0.70 | 0.67 | 0.55 | 0.51 | 0.49 | 0.72 | 0.68 | 0.85 | 0.58 | 0.96 | 1.06 | 0.80 | 0.93 | 0.96 | 0.49 | 0.50 | 0.82 | 0.69 | 0.59 | 0.27 | 0.61 | 0.63 | 0.57 | 0.48 |
| IL12A | 0.69 | 0.86 | 1.53 | 0.44 | 0.61 | 0.56 | 0.39 | 0.48 | 0.61 | 0.51 | 1.49 | 1.41 | 1.38 | 1.27 | 1.68 | 0.65 | 0.49 | 0.63 | 0.69 | 0.58 | 0.41 | 0.50 | 1.01 | 0.50 | 0.66 |
| IL17A | - | - | - | - | - | - | - | - | - | - | - | - | - | - | - | - | - | - | - | - | - | - | - | - | - |
| IL1A* | 1.18 | 0.58 | 1.60 | 0.78 | 0.75 | 0.68 | 0.44 | 0.41 | 0.60 | 0.58 | 1.05 | 0.84 | 0.99 | 0.48 | 0.73 | 0.29 | 1.03 | 0.72 | 0.26 | 0.30 | 0.40 | 0.58 | 0.55 | 0.52 | 0.58 |
| IL23R* | 1.05 | 0.16 | 1.24 | 0.91 | 1.56 | 0.60 | 0.53 | 0.61 | 0.94 | 0.92 | 0.56 | 0.77 | 1.40 | 1.24 | 0.89 | 0.65 | 0.35 | 1.12 | 0.52 | 1.05 | 1.14 | 1.57 | 1.50 | 1.17 | 1.26 |
| IL6* | 0.59 | 0.33 | 1.04 | 0.53 | 0.49 | 0.58 | 0.41 | 0.58 | 0.38 | 0.35 | 1.08 | 1.39 | 1.67 | 1.70 | 0.91 | 0.62 | 0.63 | 0.41 | 0.43 | 0.46 | 0.64 | 0.51 | 0.60 | 0.72 | 0.62 |
| KCNK9 | 0.91 | 0.49 | 1.40 | 0.74 | 0.98 | 1.08 | 0.72 | 1.40 | 0.55 | 1.03 | **2.54** | **3.46** | **3.20** | **2.64** | **2.47** | 0.72 | 0.40 | 0.64 | 0.55 | 0.74 | 0.89 | 0.99 | 0.77 | 0.59 | 0.89 |
| KLRC4 | 0.70 | 0.18 | 0.51 | 0.66 | 0.57 | 1.16 | 0.70 | 0.91 | 0.80 | 1.09 | 0.60 | 0.74 | 0.37 | 0.91 | 0.69 | 0.72 | 1.12 | 0.61 | 0.75 | 0.98 | 1.39 | 0.92 | 1.28 | 0.99 | 1.02 |
| KLRK1 | 0.60 | 0.18 | 0.76 | 0.79 | 0.46 | 0.68 | 0.66 | 0.85 | 0.70 | 0.69 | 0.74 | 0.81 | 0.54 | 0.66 | 0.70 | 0.84 | 0.93 | 0.50 | 0.63 | 0.62 | 0.74 | 0.62 | 0.71 | 0.80 | 0.50 |
| LILRA1 | 0.67 | 0.25 | 0.50 | 1.16 | 1.06 | 1.03 | 1.38 | 1.02 | 0.93 | 1.01 | 0.66 | 0.46 | 0.13 | 0.57 | 0.36 | 0.29 | 0.05 | 0.80 | 0.44 | 0.49 | 1.03 | 1.04 | 0.78 | 0.74 | 1.04 |
| LILRB1 | 0.50 | 0.47 | 0.95 | 0.71 | 0.73 | 0.76 | 0.79 | 0.88 | 0.64 | 0.49 | 1.76 | 1.16 | 0.63 | 0.64 | 0.54 | 0.58 | 0.43 | 0.45 | 0.62 | 0.38 | 0.51 | 0.66 | 0.44 | 0.46 | 0.40 |
| LOC100129342 | - | - | - | - | - | - | - | - | - | - | - | - | - | - | - | - | - | - | - | - | - | - | - | - | - |
| LOC100132252 | - | - | - | - | - | - | - | - | - | - | - | - | - | - | - | - | - | - | - | - | - | - | - | - | - |
| LOC107984355 | 0.39 | 0.71 | 1.19 | 0.74 | 0.47 | 1.02 | 0.59 | 0.52 | 0.86 | 0.92 | 1.05 | 1.04 | 0.87 | 1.03 | 1.04 | 0.38 | 0.52 | 0.43 | 0.77 | 0.43 | 0.39 | 0.46 | 0.46 | 0.47 | 0.42 |
| LOC285830 | 0.45 | 0.09 | 0.45 | 0.31 | 0.50 | 0.34 | 0.23 | 0.43 | 0.43 | 0.58 | 0.26 | 0.52 | 0.37 | 0.72 | 0.70 | 0.58 | 0.91 | 0.56 | 0.61 | 0.67 | 0.63 | 0.86 | 0.95 | 0.52 | 0.90 |
| LTN1 | 0.71 | 0.06 | 0.65 | 1.26 | 1.07 | 1.12 | 1.41 | 1.10 | 1.20 | 1.71 | 1.75 | 1.07 | 1.46 | 1.38 | 1.39 | 1.25 | 0.94 | 0.74 | 0.92 | 0.90 | 0.93 | 1.13 | 0.81 | 0.55 | 0.70 |
| LYST | 0.55 | 1.18 | **2.10** | 0.70 | 0.76 | 0.45 | 0.70 | 0.86 | 1.07 | 0.64 | 0.97 | 1.33 | 1.13 | 0.75 | **2.06** | 0.61 | 0.79 | 0.76 | 0.53 | 0.50 | 0.55 | 0.90 | 0.54 | 0.62 | 1.02 |
| MEFV | 0.80 | 0.28 | 0.51 | 0.57 | 0.50 | 0.83 | 0.61 | 0.90 | 0.55 | 1.10 | 0.79 | 0.34 | 0.48 | 0.32 | 0.71 | 0.29 | 0.31 | 0.24 | 0.30 | 0.27 | 0.45 | 0.43 | 0.32 | 0.22 | 0.28 |
| MICA | 0.22 | 0.33 | 0.15 | 0.27 | 0.42 | 0.47 | 0.57 | 0.37 | 0.44 | 0.51 | 0.80 | 0.87 | 1.26 | 0.98 | 0.73 | 1.07 | 0.81 | 0.97 | 0.69 | 0.69 | 0.70 | 0.77 | 0.74 | 0.69 | 0.74 |
| MN1 | 0.61 | 0.15 | 0.55 | 0.46 | 0.72 | 0.63 | 0.65 | 0.86 | 0.68 | 0.56 | 0.99 | 0.82 | 1.30 | 1.01 | 1.27 | 0.80 | 0.88 | 0.86 | 0.62 | 0.75 | 0.75 | 0.62 | 1.01 | 0.85 | 0.99 |
| MOG* | - | - | - | - | - | - | - | - | - | - | - | - | - | - | - | - | - | - | - | - | - | - | - | - | - |
| MSX2 | - | - | - | - | - | - | - | - | - | - | - | - | - | - | - | - | - | - | - | - | - | - | - | - | - |
| MUC21 | - | - | - | - | - | - | - | - | - | - | - | - | - | - | - | - | - | - | - | - | - | - | - | - | - |
| NAV2 | 1.70 | 0.27 | **2.00** | 0.62 | 0.50 | 0.77 | 0.57 | 0.65 | 0.65 | 0.67 | **2.25** | 1.47 | 1.47 | 0.77 | **2.08** | 1.21 | 1.28 | 1.09 | 0.84 | 0.90 | 1.03 | 1.12 | 1.23 | 1.09 | 1.22 |
| NOD2* | 0.93 | 0.75 | **1.65** | 1.16 | 1.62 | 1.38 | 1.26 | 0.89 | 1.42 | 1.42 | **3.13** | **2.01** | **2.56** | **1.80** | **2.10** | 0.90 | 0.33 | 0.49 | 0.31 | 0.37 | 1.44 | 1.35 | 1.24 | 1.15 | 0.86 |
| OSR1* | - | - | - | - | - | - | - | - | - | - | - | - | - | - | - | - | - | - | - | - | - | - | - | - | - |
| OVCH1 | 0.98 | 0.12 | 1.02 | 0.95 | 1.17 | 1.19 | 0.99 | 0.82 | 0.81 | 0.78 | 0.89 | 0.53 | 0.45 | 0.55 | 0.97 | 1.08 | 0.87 | 1.17 | 1.56 | 1.38 | 1.00 | 1.24 | 0.87 | 0.91 | 1.23 |
| PAX8 | 0.56 | 0.25 | 0.87 | 0.84 | 0.70 | 0.59 | 0.65 | 0.60 | 0.81 | 0.70 | 1.11 | 0.93 | 1.38 | 0.87 | 0.81 | 0.36 | 0.41 | 0.55 | 0.87 | 0.59 | 0.45 | 0.52 | 0.62 | 0.73 | 0.48 |
| PLEKHB1 | 0.84 | 0.11 | 0.95 | 0.47 | 0.53 | 0.77 | 0.97 | 0.58 | 1.17 | 0.73 | 0.66 | 0.86 | 0.82 | 0.84 | 0.85 | 0.73 | 0.58 | 0.87 | 0.79 | 0.73 | 0.75 | 0.56 | 0.60 | 0.68 | 0.42 |
| PMFBP1 | 0.21 | 0.18 | 0.38 | 0.57 | 0.46 | 0.46 | 0.39 | 0.77 | 1.05 | 0.36 | 0.67 | 0.61 | 0.82 | 0.40 | 0.60 | 0.47 | 0.72 | 0.42 | 0.67 | 0.78 | 0.51 | 0.39 | 0.48 | 0.42 | 0.42 |
| POU5F1 | 0.29 | 0.08 | 0.17 | 0.42 | 0.62 | 0.51 | 0.53 | 0.42 | 0.45 | 0.52 | 0.38 | 1.29 | 0.70 | 0.63 | 1.22 | 1.12 | 1.27 | 1.47 | 1.59 | 1.57 | 0.81 | 1.00 | 1.38 | 1.42 | 1.62 |
| PPP1R11* | - | - | - | - | - | - | - | - | - | - | - | - | - | - | - | - | - | - | - | - | - | - | - | - | - |
| PSMD14 | 0.52 | 0.10 | 0.41 | 0.45 | 0.31 | 0.37 | 0.30 | 0.89 | 0.67 | 0.17 | 0.97 | 0.94 | 0.87 | 0.39 | 0.99 | 0.67 | 0.41 | 0.61 | 0.24 | 0.16 | 0.38 | 0.47 | 0.40 | 0.42 | 0.51 |
| PSOR1C1* | 0.07 | 0.07 | 0.09 | 0.74 | 0.50 | 0.87 | 0.56 | 0.56 | 0.72 | 0.68 | 0.95 | 0.65 | 0.73 | 0.54 | 0.52 | 0.50 | 0.40 | 0.44 | 0.52 | 0.57 | 0.55 | 0.71 | 0.71 | 0.70 | 0.71 |
| RALGAPA2* | 0.82 | 0.28 | 0.52 | 1.65 | 1.59 | 1.76 | **1.97** | 1.79 | 1.53 | 1.48 | **2.61** | **2.05** | **2.70** | **2.84** | **2.04** | 0.00 | **3.01** | **2.23** | **2.99** | 0.00 | **2.08** | **2.39** | **1.60** | 1.24 | 0.52 |
| RIMBP2 | 0.37 | 0.14 | 0.47 | 0.82 | 1.03 | 1.14 | 0.97 | 1.01 | 0.98 | 0.97 | 1.27 | 1.06 | 1.12 | 1.01 | 1.10 | 1.34 | 0.91 | 1.64 | 1.19 | 1.19 | 0.78 | 0.75 | 0.72 | 0.69 | 0.76 |
| RNF39* | - | - | - | - | - | - | - | - | - | - | - | - | - | - | - | - | - | - | - | - | - | - | - | - | - |
| SACM1L | 0.20 | 0.86 | 1.06 | 0.76 | 1.00 | 0.73 | 0.84 | 0.74 | 0.75 | 0.76 | 0.72 | 0.67 | 0.61 | 1.08 | 0.61 | 0.89 | 0.71 | 1.11 | 0.71 | 0.71 | 1.26 | 0.90 | 1.77 | 1.50 | 1.61 |
| SAMD3 | 0.66 | 0.29 | 0.91 | 0.99 | 0.87 | 0.78 | 1.35 | 1.08 | 1.22 | 1.07 | 1.48 | 1.37 | 1.40 | 1.24 | 1.58 | 1.36 | 1.31 | 1.16 | 1.52 | 1.72 | 1.05 | 1.38 | 1.31 | 1.18 | 0.82 |
| SEMA6D | 1.35 | 0.10 | 1.25 | 0.74 | 0.45 | 0.78 | 0.91 | 0.99 | 0.70 | 0.66 | **2.08** | 1.26 | 1.25 | 1.41 | 1.43 | 0.94 | 1.60 | 1.03 | 0.63 | 0.76 | 0.69 | 0.58 | 0.45 | 0.35 | 0.41 |
| SGPP2 | 0.51 | 0.30 | 0.76 | 0.87 | 0.46 | 0.82 | 0.87 | 0.86 | 0.82 | 0.65 | 1.15 | 0.85 | 1.10 | 1.16 | 0.95 | 1.04 | 1.05 | 0.94 | 1.02 | 0.61 | 1.12 | 1.17 | 0.56 | 1.10 | 0.58 |
| SLC41A2* | 1.17 | 0.37 | 0.60 | 1.15 | 0.97 | 1.14 | 1.04 | 1.08 | 0.75 | 1.19 | 1.24 | 1.20 | 1.42 | 1.39 | 1.30 | 0.40 | 0.78 | 0.45 | 0.40 | 0.41 | 1.48 | 0.86 | 1.11 | 1.34 | 0.82 |
| SLC43A3 | 0.54 | 0.47 | 1.01 | 0.65 | 0.89 | 0.54 | 0.59 | 0.49 | 0.64 | 0.63 | 0.87 | 1.07 | 0.92 | 0.70 | 1.20 | 0.50 | 0.76 | 0.51 | 0.46 | 0.60 | 1.36 | 1.24 | 1.24 | 1.26 | 1.14 |
| SLC44A4* | 0.19 | 0.06 | 0.16 | 0.30 | 0.50 | 0.48 | 0.55 | 0.27 | 0.84 | 0.45 | 0.74 | 0.69 | 0.44 | 0.37 | 0.69 | 0.58 | 0.39 | 0.56 | 0.79 | 0.79 | 0.77 | 0.64 | 0.79 | 0.77 | 0.83 |
| SLIT2 | 0.23 | 0.48 | 0.68 | 1.40 | 1.03 | 1.13 | 1.00 | 1.20 | 1.29 | 1.44 | 0.83 | 1.22 | 1.12 | 1.03 | 0.85 | **2.40** | **2.05** | **2.38** | **2.49** | **2.32** | 1.11 | 1.57 | 1.14 | 1.59 | 1.11 |
| SMARCA2* | 0.45 | 0.11 | 0.58 | 1.14 | 1.01 | 1.40 | 1.68 | 1.12 | 1.92 | 1.03 | 0.72 | 1.22 | 0.87 | 1.43 | 0.77 | 0.66 | 1.42 | 0.73 | 1.22 | 1.68 | 1.04 | 0.75 | 1.32 | 1.36 | 1.32 |
| SMG6 | 0.21 | 0.45 | 0.59 | 1.16 | 0.87 | 0.98 | 1.04 | 1.03 | 0.94 | 0.73 | 0.92 | 1.13 | 1.63 | 1.24 | 1.00 | 1.50 | 1.46 | 1.37 | 1.24 | 0.98 | 1.23 | 1.05 | 0.91 | 1.43 | 1.18 |
| SORBS2 | 0.24 | 0.61 | 0.81 | 1.02 | 1.10 | 1.16 | 1.12 | 1.01 | 1.21 | 1.24 | 0.90 | 1.10 | 0.87 | 1.39 | 0.98 | 0.41 | 0.44 | 0.39 | 0.40 | 0.37 | 0.53 | 0.56 | 0.50 | 0.40 | 0.61 |
| STAT4 | 0.95 | 0.40 | 0.55 | 1.00 | 0.49 | 1.69 | 0.79 | 1.20 | 0.91 | 0.94 | 0.42 | 0.42 | 0.48 | 0.59 | 0.48 | 1.40 | 1.61 | 1.25 | 1.48 | 1.56 | 1.57 | 1.91 | 1.56 | 1.91 | 1.83 |
| STK39 | 0.98 | 0.40 | 0.57 | 0.57 | 0.51 | 0.41 | 0.68 | 0.59 | 0.65 | 0.55 | 0.87 | 0.94 | 1.29 | 1.32 | 1.23 | 0.40 | 0.62 | 0.84 | 1.08 | 0.96 | 0.74 | 0.83 | 0.98 | 1.14 | 0.95 |
| STX8* | 0.30 | 0.69 | 0.96 | 0.99 | 0.82 | 1.40 | 0.87 | 1.06 | 0.86 | 0.79 | 1.63 | 1.79 | 1.72 | 1.62 | 1.53 | 0.65 | 0.71 | 0.64 | 0.61 | 0.64 | 0.61 | 0.65 | 0.54 | 0.72 | 0.68 |
| SUMO4 | 0.74 | 0.19 | 0.91 | 1.60 | 1.38 | 1.25 | 1.66 | 1.56 | 1.94 | 1.42 | 1.04 | 0.73 | 0.72 | 0.65 | 0.36 | 0.64 | 1.02 | 0.73 | 0.33 | 0.88 | 0.98 | 1.11 | 1.03 | 1.17 | 1.01 |
| SUSD1 | 0.28 | 0.09 | 0.19 | 0.63 | 0.31 | 0.75 | 0.56 | 0.76 | 0.68 | 0.61 | 1.37 | 1.46 | 1.06 | 0.99 | 1.42 | 0.76 | 1.38 | 0.68 | 0.60 | 0.94 | 0.51 | 0.92 | 0.67 | 0.93 | 1.05 |
| TCF19 | 0.29 | 0.08 | 0.17 | 0.42 | 0.62 | 0.51 | 0.53 | 0.42 | 0.45 | 0.52 | 0.38 | 1.29 | 0.70 | 0.63 | 1.22 | 1.12 | 1.27 | 1.47 | 1.59 | 1.57 | 0.81 | 1.00 | 1.38 | 1.42 | 1.62 |
| TENM4 | 0.95 | 0.60 | 1.51 | 0.31 | 0.33 | 0.59 | 0.55 | 0.70 | 0.34 | 0.36 | 0.76 | 1.32 | 1.48 | 1.44 | 0.97 | 0.61 | 0.43 | 0.56 | 0.63 | 0.59 | 1.10 | 1.24 | 1.13 | 0.64 | 1.17 |
| TFCP2L1* | 1.21 | 0.58 | 0.58 | 0.56 | 0.34 | 0.48 | 0.26 | 0.52 | 0.27 | 0.44 | 0.64 | 0.85 | 0.48 | 0.83 | 0.89 | 0.82 | 0.60 | 0.77 | 0.89 | 1.15 | 0.31 | 0.57 | 0.80 | 0.75 | 0.78 |
| TLR4 | 0.89 | 0.27 | 1.20 | 0.49 | 0.55 | 0.50 | 0.70 | 0.73 | 0.50 | 0.52 | 0.54 | 0.93 | 0.76 | 0.91 | 0.49 | 0.81 | 0.48 | 0.55 | 0.77 | 0.74 | 0.73 | 1.00 | 0.66 | 0.93 | 0.76 |
| TMEM132B | 0.58 | 0.68 | 1.28 | 0.78 | 0.77 | 0.92 | 0.62 | 0.83 | 0.84 | 0.94 | 1.89 | 1.51 | 1.56 | 1.43 | 1.49 | 0.40 | 0.51 | 0.33 | 0.51 | 0.35 | 0.34 | 0.49 | 0.52 | 0.31 | 0.63 |
| TNF | 0.11 | 0.07 | 0.18 | 1.12 | 1.01 | 0.98 | 1.00 | 0.70 | 1.01 | 1.13 | 0.49 | 0.65 | 0.30 | 0.73 | 0.51 | 0.46 | 0.19 | 0.37 | 0.60 | 0.61 | 0.99 | 1.10 | 1.25 | 0.95 | 1.09 |
| TNFAIP3 | 0.89 | 0.02 | 1.10 | 0.34 | 0.44 | 0.45 | 0.53 | 0.54 | 0.73 | 0.69 | 0.64 | 1.03 | 1.36 | 0.49 | 0.84 | 0.87 | 1.16 | 0.66 | 0.79 | 1.37 | 0.85 | 0.77 | 0.83 | 0.86 | 0.78 |
| TRIM31 | 0.08 | 0.07 | 0.11 | 0.53 | 0.43 | 0.21 | 0.76 | 0.57 | 0.85 | 0.71 | 0.41 | 0.65 | 0.68 | 0.36 | 0.55 | 0.95 | 0.37 | 0.80 | 0.91 | 1.03 | 0.56 | 0.64 | 0.34 | 0.90 | 0.73 |
| TTLL7* | 0.13 | 0.30 | 0.39 | 0.46 | 0.79 | 0.40 | 0.61 | 0.46 | 0.89 | 0.42 | 1.08 | 1.11 | 1.01 | 0.92 | 1.01 | 1.22 | 1.13 | 1.39 | 0.78 | 0.68 | 0.62 | 0.72 | 0.56 | 0.68 | 0.78 |
| UBAC2 | 0.38 | 0.11 | 0.41 | 0.89 | 0.72 | 0.51 | 0.72 | 0.71 | 0.84 | 0.97 | **1.83** | **1.67** | **1.81** | **1.94** | **1.69** | **2.00** | **2.20** | **2.08** | **2.03** | **1.98** | **2.02** | **2.11** | **2.63** | **2.49** | **2.18** |
| UBASH3B | 0.60 | 0.60 | 1.24 | 1.30 | 1.32 | 0.88 | 1.32 | 1.47 | 0.73 | 0.87 | **2.05** | **1.72** | **1.82** | **1.60** | **1.69** | 1.41 | 1.14 | 1.32 | 1.32 | 1.42 | 1.15 | 0.64 | 1.16 | 0.41 | 1.36 |
| UBD | - | - | - | - | - | - | - | - | - | - | - | - | - | - | - | - | - | - | - | - | - | - | - | - | - |
| ZNRD1* | - | - | - | - | - | - | - | - | - | - | - | - | - | - | - | - | - | - | - | - | - | - | - | - | - |

- Genes with highest or lowest quartile population genetic parameter and frequency spectrum test results unique to East Asians (EAS)

**Supplemental Table 15.** Direction of Selection (DoS), McDonald-Kreitman test P value, Neutrality Index (NI), and Alpha (α) parameter estimates of Behcet disease associated genes in the 1000 Genomes East Asian populations.

|  | **CDX** | | | | **CHB** | | | | **CHS** | | | | **JPT** | | | | **KHV** | | | |
| --- | --- | --- | --- | --- | --- | --- | --- | --- | --- | --- | --- | --- | --- | --- | --- | --- | --- | --- | --- | --- |
| **Gene** | **DoS^a^** | **P^b^** | **NI^c^** | α**^d^** | **DoS** | **P** | **NI** | α | **DoS** | **P** | **NI** | α | **DoS** | **P** | **NI** | α | **DoS** | **P** | **NI** | α |
| ABCB5 | -0.5 | 0.04 | 1.45 | -0.45 | -0.5 | 0.23 | 1.55 | -0.55 | -0.5 | 0.04 | 1.31 | -0.31 | -0.5 | 0.01 | 7.1 | -6.1 | -0.5 | 0.05 | 5.3 | -4.3 |
| API5 | -1 | 1 | 0 | 0 | 0 | 1 | 0 | 0 | 0 | 1 | 0 | 0 | 0 | 1 | 0 | 0 | 0 | 1 | 0 | 0 |
| ASB18 | -1 | 1 | 0 | 0 | -1 | 1 | 0 | 0 | -1 | 0.25 | 0 | 0 | -1 | 0.33 | 0 | 0 | -1 | 0.33 | 0 | 1 |
| ATP8A1 | -1 | 1 | 0 | 1 | -1 | 1 | 0 | 1 | -1 | 0.33 | 0 | 1 | -1 | 0.024 | 0 | 1 | -1 | 1 | 0 | 1 |
| BAG6 | -0.27 | 0.3 | 0 | 0 | 0.47 | 0.09 | 0.12 | 0.87 | 0.12 | 0.61 | 0.57 | 0.42 | 0.39 | 0.22 | 0.19 | 0.8 | 0.22 | 0.35 | 0.38 | 0.61 |
| BTNL2 | 0 | 0 | 0 | 0 | 0 | 0 | 0 | 0 | 0 | 0 | 0 | 0 | 0 | 0 | 0 | 0 | 0 | 0 | 0 | 0 |
| C10orf11 | -1 | 1 | 0 | 0 | -1 | 1 | 0 | 0 | -1 | 1 | 0 | 0 | -1 | 1 | 0 | 0 | -1 | 1 | 1 | 0 |
| C6orf10 | 0 | 1 | 0 | 0 | 0.05 | 1 | 0 | 1 | 0 | 1 | 0 | 0 | 0.05 | 1 | 0 | 1 | 0.06 | 1 | 0 | 1 |
| C6orf47 | -0.03 | 1 | 1.2 | -0.2 | -0.28 | 1 | 0 | 0 | 0.05 | 1 | 0.8 | 0.2 | -0.03 | 1 | 1.2 | -0.2 | -0.11 | 1 | 2 | -1 |
| SLC22A23/C6orf85 | -0.62 | 0.007 | 24 | -23 | -0.48 | 0.03 | 12.6 | -12 | -0.6 | 0.02 | 21 | -20 | -0.56 | 0.01 | 18 | -17 | -0.56 | 0.01 | 18 | -17 |
| CCDC180 | 0.04 | 1 | 0.75 | 0.25 | 0.04 | 1 | 0.75 | 0.25 | 0.09 | 0.61 | 0.56 | 0.43 | 0.17 | 0.38 | 0.37 | 0.62 | 0.04 | 1 | 0.75 | 0.25 |
| CCHCR1 | 0 | ─ | 0 | 0 | 0 | ─ | 0 | 0 | 0 | ─ | 0 | 0 | 0 | ─ | 0 | 0 | 0 | ─ | 0 | 0 |
| CCR1 | 0 | ─ | 0 | 0 | 0 | ─ | 0 | 0 | 0 | ─ | 0 | 0 | 0 | ─ | 0 | 0 | 0 | ─ | 0 | 0 |
| CCR3 | 0 | 1 | 0 | 0 | 0 | 1 | 0 | 0 | 0 | 1 | 0 | 0 | 0 | 1 | 0 | 0 | 0 | 1 | 0 | 0 |
| CDH26 | -0.01 | 1 | 1.07 | -0.07 | 0.23 | 0.37 | 0.35 | 0.64 | 0.13 | 0.6 | 0.53 | 0.46 | 0.23 | 0.55 | 0.35 | 0.64 | -0.01 | 1 | 1.07 | -0.07 |
| CEP135 | -0.03 | 1 | 1.2 | -0.2 | -0.28 | 1 | 0 | 0 | 0.21 | 1 | 0.4 | 0.6 | -0.28 | 1 | 0 | 0 | -0.28 | 1 | 0 | 0 |
| COL12A1 | -0.44 | 0.14 | 6.75 | -5.75 | -0.7 | 0.16 | 5.33 | -4.3 | -0.56 | 0.36 | 15.75 | -15 | -0.69 | 0.14 | 6.66 | -5.6 | -0.51 | 0.14 | 10.12 | -9.12 |
| CPLX1 | -0.35 | 0.3 | 5 | -4 | -0.3 | 0.55 | 3.75 | -2.75 | -0.05 | 1 | 1.25 | -0.25 | -0.05 | 1 | 1.25 | -0.25 | -0.22 | 1 | 2.5 | -1.5 |
| CPVL | 0.5 | 0.18 | 0 | 1 | 0.57 | 0.06 | 0 | 1 | 0.6 | 0.06 | 0 | 1 | 0.8 | 0.01 | 0 | 1 | 0.6 | 0.06 | 0 | 1 |
| CTNNA2 | 0 | ─ | 0 | 0 | 0 | 0 | 0 | 0 | 0 | 0 | 0 | 0 | 0 | 0 | 0 | 0 | 0 | 0 | 0 | 0 |
| DEPDC1 | 0 | 1 | 0 | 0 | 0 | 1 | 0 | 0 | 0 | 1 | 0 | 0 | 0 | 1 | 0 | 0 | 0 | 1 | 0 | 0 |
| DNMT3A | -0.16 | 1 | 2 | -1 | -0.33 | 1 | 4 | -3 | -0.16 | 1 | 2 | -1 | -0.5 | 0.4 | 2 | -1 | -0.33 | 1 | 2 | -1 |
| DTL | 0 | ─ | 0 | 0 | 0 | ─ | 0 | 0 | 0 | ─ | 0 | 0 | 0 | ─ | 0 | 0 | 0 | ─ | 0 | 0 |
| EBF2 | -1 | 1 | 0 | 1 | 1 | 1 | 0 | 1 | 0 | 1 | 0 | 0 | 0 | 1 | 0 | 0 | 0 | 1 | 0 | 0 |
| ERAP1 | -0.36 | 0.26 | 5 | -4 | -0.18 | 0.58 | 2.2 | -1.2 | -0.36 | 0.27 | 4.9 | -3.9 | -0.35 | 0.28 | 4.5 | -3.5 | -0.41 | 0.11 | 6.5 | -5.5 |
| FUT2 | -0.25 | 0.57 | 3 | -2 | -0.08 | 1 | 1.5 | -0.5 | -0.25 | 0.57 | 3 | -2 | -0.5 | 0.46 | 0 | 0 | -0.08 | 1 | 1.5 | -0.5 |
| GABBR1 | -0.1 | 0.73 | 1.52 | -0.52 | -0.2 | 0.17 | 2.32 | -1.32 | -0.2 | 0.21 | 2.43 | -1.43 | -0.03 | 1 | 1.14 | -0.14 | -0.17 | 0.35 | 2.08 | -1.08 |
| GALNT10 | 1 | 1 | 1.5 | 1 | 1 | 1 | 1 | 1 | 1 | 1 | 0.75 | 1 | 1 | 1 | 0.5 | 1 | 1 | 1 | 0.75 | 1 |
| GALNTL1 | -0.46 | 0.24 | 8 | -7 | -0.46 | 0.46 | 8 | -7 | -0.3 | 0.54 | 4 | -3 | -0.6 | 0.2 | 16 | -15 | -0.4 | 0.52 | 6 | -5 |
| GAS2 | 0 | 1 | 0 | 0 | 0 | 1 | 0 | 0 | 0 | 1 | 0 | 0 | 0 | 1 | 0 | 0 | -1 | 1 | 0 | 0 |
| GIMAP1 | 0.25 | 1 | 0.33 | 0.66 | 0.25 | 1 | 0.33 | 0.66 | 0.75 | 1 | 0 | 1 | 0.41 | 1 | 0.16 | 0.83 | 0.75 | 1 | 0 | 1 |
| GIMAP2 | 0.16 | 1 | 0.5 | 0.5 | 0.16 | 1 | 0.5 | 0.5 | 0.16 | 1 | 0.5 | 0.5 | 0.16 | 1 | 0.5 | 0.5 | 0.16 | 1 | 0.5 | 0.5 |
| GIMAP4 | 0 | ─ | 0 | 0 | 0 | 0 | 0 | 0 | 0 | 0 | 0 | 0 | 0 | 0 | 0 | 0 | 0 | 0 | 0 | 0 |
| HCG27 | 0.5 | 1 | 0 | 1 | 0.5 | 1 | 0 | 1 | 0.5 | 1 | 0 | 1 | 0.5 | 1 | 0 | 1 | 0.5 | 1 | 0 | 1 |
| HCG9 | 0 | ─ | 0 | 0 | 0 | 0 | 0 | 0 | 0 | 0 | 0 | 0 | 0 | 0 | 0 | 0 | 0 | 0 | 0 | 0 |
| HERPUD2 | -0.66 | 0.14 | 0 | 0 | -0.66 | 0.1 | 0 | 0 | -0.46 | 0.46 | 8 | -7 | -0.66 | 0.14 | 0 | 0 | -0.66 | 0.4 | 0 | 0 |
| HIVEP3 | -0.17 | 0.43 | 2 | -1 | -0.5 | 0.009 | 13.5 | -12.5 | -0.34 | 0.08 | 4.2 | -3.2 | -0.41 | 0.03 | 6.75 | -5.75 | -0.25 | 0.37 | 2.8 | -1.8 |
| HLA-B | 0.26 | 0.55 | 2.43 | 1 | 0.28 | 0.54 | 2.81 | 1 | 0.27 | 0.55 | 2.43 | 1 | 0.28 | 0.54 | 2.43 | 1 | 0.25 | 0.55 | 2.2 | 1 |
| HLA-C | -0.16 | 0.31 | 2.43 | -1.43 | -0.18 | 0.26 | 2.81 | -1.81 | -0.16 | 0.31 | 2.43 | -1.43 | -0.16 | 0.31 | 2.43 | -1.43 | -0.14 | 0.6 | 2.2 | -1.2 |
| HLA-DQA1 | 0 | ─ | 0 | 0 | 0 | ─ | 0 | 0 | 0 | ─ | 0 | 0 | 0 | ─ | 0 | 0 | 0 | ─ | 0 | 0 |
| HLA-DQB1 | 0 | ─ | 0 | 0 | 0 | ─ | 0 | 0 | 0 | ─ | 0 | 0 | 0 | ─ | 0 | 0 | 0 | ─ | 0 | 0 |
| HLA-F | -0.14 | 1 | 0 | 0 | 0 | 1 | 1 | 0 | 0.02 | 1 | 0.83 | 0.16 | 0.02 | 1 | 0.83 | 0.16 | 0.02 | 1 | 0.83 | 0.16 |
| HLA-G | 0.37 | 0.11 | 0.19 | 0.8 | 0.17 | 0.6 | 0.44 | 0.55 | 0.06 | 1 | 0.74 | 0.25 | 0.16 | 0.37 | 0.44 | 0.55 | 0.2 | 0.35 | 0.39 | 0.6 |
| HMP19 | 0.35 | 0.41 | 0.16 | 0.83 | 0.35 | 0.49 | 0.16 | 0.83 | 0.19 | 1 | 0.33 | 0.66 | 0.52 | 0.18 | 0.08 | 0.91 | 0.35 | 0.41 | 0.16 | 0.83 |
| HNF4G | -1 | 0.2 | 0 | 0 | -1 | 0.25 | 0 | 0 | -0.66 | 1 | 0 | 0 | -1 | 0.33 | 0 | 0 | -1 | 0.33 | 0 | 0 |
| IL-10 | 0 | 1 | 0 | 0 | 0 | 1 | 0 | 0 | 0 | 1 | 0 | 0 | 0 | 1 | 0 | 0 | 0 | 1 | 0 | 0 |
| IL12A | -0.16 | 1 | 2 | -1 | 0 | 1 | 0 | 0 | -1 | 0.33 | 0 | 0 | -0.5 | 1 | 0 | 0 | -1 | 0.33 | 0 | 0 |
| IL17F | -0.25 | 1 | 0 | 0 | -0.25 | 1 | 0 | 0 | -0.25 | 1 | 0 | 0 | -0.25 | 1 | 0 | 0 | -0.25 | 0.44 | 0 | 0 |
| IL23R | 0 | ─ | 0 | 0 | 0 | ─ | 0 | 0 | 0 | ─ | 0 | 0 | 0 | ─ | 0 | 0 | 0 | ─ | 0 | 0 |
| IL6 | 0 | 1 | 0 | 0 | 0 | 1 | 0 | 0 | 0 | 1 | 0 | 0 | 0 | 1 | 0 | 0 | 0 | 1 | 0 | 0 |
| KCNK9 | 0 | 1 | 0 | 0 | 0 | 1 | 0 | 0 | 0 | 1 | 0 | 0 | 0 | 1 | 0 | 0 | 0 | 1 | 0 | 0 |
| KLRC4 | 0 | 1 | 0 | 0 | 0 | 1 | 0 | 0 | 0 | 1 | 0 | 0 | 0 | 1 | 0 | 0 | 0 | 1 | 0 | 0 |
| KLRK1 | -1 | 0.33 | 0 | 1 | -1 | 0.33 | 0 | 1 | -1 | 1 | 0 | 1 | -1 | 0.33 | 0 | 1 | -1 | 1 | 0 | 1 |
| LILRA1 | -0.19 | 0.09 | 0.38 | 0.61 | 0.04 | 0.76 | 0.77 | 0.22 | 0.11 | 0.46 | 0.54 | 0.45 | 0.08 | 0.39 | 0.62 | 0.37 | 0.22 | 0.06 | 0.33 | 0.66 |
| LILRB1 | 0.01 | 1 | 0.95 | 0.05 | -0.08 | 0.71 | 1.63 | -0.63 | -0.05 | 1 | 1.31 | -0.31 | -0.1 | 0.71 | 1.83 | -0.83 | -0.11 | 0.71 | 1.89 | -0.89 |
| LINC01499(API5) | -1 | 1 | 0 | 0 | 0 | 1 | 0 | 0 | 0 | 1 | 0 | 0 | 0 | 1 | 0 | 0 | 0 | 1 | 0 | 0 |
| LOC285830 ( HLA-F antisense RNA1) | -0.14 | 1 | 0 | 0 | 0 | 1 | 1 | 0 | 0.02 | 1 | 0.83 | 0.16 | 0.02 | 1 | 0.83 | 0.16 | 0.02 | 1 | 0.83 | 0.16 |
| LTN1(RNF160) | 0.08 | 0.71 | 0.7 | 0.3 | 0.06 | 0.74 | 0.75 | 0.25 | 0.09 | 0.72 | 0.65 | 0.34 | 0.05 | 1 | 0.78 | 0.21 | 0.09 | 0.72 | 0.65 | 0.34 |
| LYST/NID1 | 0 | ─ | 0 | 0 | 0 | 0 | 0 | 0 | 0 | 0 | 0 | 0 | 0 | 0 | 0 | 0 | 0 | 0 | 0 | 0 |
| MEFV | 0.08 | 1 | 0.6 | 0.4 | 0.03 | 1 | 0.8 | 0.2 | 0.03 | 1 | 0.8 | 0.2 | -0.02 | 1 | 1.2 | -0.2 | -0.04 | 1 | 1.4 | -0.4 |
| MICA | -0.16 | 0.16 | 4.7 | -3.7 | -0.17 | 0.15 | 5.5 | -4.5 | -0.12 | 0.31 | 2.6 | -1.63 | -0.16 | 0.15 | 4.7 | -3.7 | -0.12 | 0.31 | 2.6 | -1.63 |
| MN1 | 0.52 | 1 | 0 | 1 | -0.47 | 0.13 | 0 | 0 | -0.47 | 1 | 0 | 0 | -0.22 | 0.6 | 2.66 | -1.66 | 0.2 | 1 | 0.44 | 0.55 |
| MOG | 0 | ─ | 0 | 0 | 0 | ─ | 0 | 0 | 0 | ─ | 0 | 0 | 0 | ─ | 0 | 0 | 0 | ─ | 0 | 0 |
| MSX2 | 0 | ─ | 0 | 0 | 0 | ─ | 0 | 0 | 0 | ─ | 0 | 0 | 0 | ─ | 0 | 0 | 0 | ─ | 0 | 0 |
| MUC21 | 0 | ─ | 0 | 0 | 0 | ─ | 0 | 0 | 0 | ─ | 0 | 0 | 0 | ─ | 0 | 0 | 0 | ─ | 0 | 0 |
| NAV2 | -0.36 | 1 | 4.6 | -3.6 | -0.41 | 1 | 10 | -9 | -0.2 | 1 | 4 | -3 | -0.03 | 1 | 1.16 | -0.16 | -0.2 | 1 | 2.33 | -1.33 |
| NOD2 | -0.03 | 1 | 1.14 | -0.14 | -0.2 | 1 | 2.85 | -1.85 | -0.03 | 1 | 1.14 | -0.14 | -0.2 | 1 | 2.85 | -1.85 | 0.03 | 1 | 0.85 | 0.14 |
| OSR1 | 0 | ─ | 0 | 0 | 0 | ─ | 0 | 0 | 0 | ─ | 0 | 0 | 0 | ─ | 0 | 0 | 0 | ─ | 0 | 0 |
| OVCH1 | -0.24 | 0.2 | 3.75 | -2.75 | -0.25 | 0.19 | 4.06 | -3.06 | -0.21 | 0.22 | 3.12 | -2.12 | -0.22 | 0.23 | 3.09 | -2.09 | -0.26 | 0.18 | 4.68 | -3.68 |
| PAX8 | 0 | ─ | 0 | 0 | 0 | ─ | 0 | 0 | 0 | ─ | 0 | 0 | 0 | ─ | 0 | 0 | 0 | ─ | 0 | 0 |
| PLEKHB1 | 0 | 1 | 0 | 0 | -0.2 | 1 | 0 | 0 | -0.2 | 1 | 0 | 0 | 0 | 1 | 0 | 0 | -0.2 | 1 | 0 | 0 |
| PMFBP1 | -0.38 | 0.26 | 0.44 | 0.55 | -0.38 | 0.11 | 1.4 | -0.4 | -0.24 | 0.35 | 3.75 | -2.75 | -0.38 | 0.12 | 0.76 | 0.23 | -0.38 | 0.11 | 0.89 | 0.1 |
| POU5F1 | 0.09 | 1 | 0.66 | 0.33 | -0.28 | 0.59 | 3.33 | -2.33 | 0.02 | 1 | 0.88 | 0.11 | -0.23 | 0.59 | 2.66 | -1.6 | -0.28 | 0.59 | 3.33 | -2.3 |
| PPP1R11 | 0 | ─ | 0 | 0 | 0 | ─ | 0 | 0 | 0 | ─ | 0 | 0 | 0 | ─ | 0 | 0 | 0 | ─ | 0 | 0 |
| PSMD14 | 0 | ─ | 0 | 0 | 0 | ─ | 0 | 0 | 0 | ─ | 0 | 0 | 0 | ─ | 0 | 0 | 0 | ─ | 0 | 0 |
| PSORS1C1 | -0.14 | 0.19 | 1.86 | -0.8 | -0.17 | 0.05 | 2.25 | -1.2 | -0.12 | 0.2 | 1.76 | -0.7 | -0.15 | 0.13 | 2.01 | -1.01 | -0.17 | 0.05 | 2.25 | -1.2 |
| RALGAPA2 | -0.16 | 1 | 2 | -1 | -0.16 | 1 | 2 | -1 | 0.33 | 1 | 0 | 1 | -0.16 | 1 | 2 | -1 | -0.16 | 1 | 3 | -1 |
| RIMBP2 | -0.38 | 1 | 5 | -4 | -0.54 | 1 | 12.5 | -11.5 | -0.25 | 1 | 3 | -2 | -0.38 | 1 | 5 | -4 | -0.26 | 1 | 3.33 | -2.33 |
| RNF39 | 0 | ─ | 0 | 0 | 0 | ─ | 0 | 0 | 0 | ─ | 0 | 0 | 0 | ─ | 0 | 0 | 0 | ─ | 0 | 0 |
| SACM1L | 0 | 1 | 0 | 0 | 0 | 1 | 0 | 0 | 0 | 1 | 0 | 0 | 0 | 1 | 0 | 0 | 0 | 1 | 0 | 0 |
| SAMD3  (TMEM200A) | -0.01 | 1 | 1.09 | -0.09 | -0.2 | 1 | 0 | 0 | -0.01 | 1 | 1.09 | -0.09 | -0.26 | 0.53 | 0 | 0 | -0.16 | 0.61 | 3.27 | -2.27 |
| SEMA6D | 0.07 | 1 | 0.75 | 0.25 | -0.3 | 0.32 | 3.5 | -2.5 | -0.17 | 1 | 2 | -1 | -0.17 | 1 | 2 | -1 | 0 | 1 | 1 | 0 |
| SGPP2 | 0 | ─ | 0 | 0 | 0 | ─ | 0 | 0 | 0 | ─ | 0 | 0 | 0 | ─ | 0 | 0 | 0 | ─ | 0 | 0 |
| SLC41A2 | 0.5 | 1 | 0 | 1 | 0.5 | 1 | 0 | 1 | 0.5 | 1 | 0 | 1 | -0.66 | 1 | 1 | 0 | 0.5 | 1 | 0 | 1 |
| SLC43A3 | 0.66 | 0.4 | 0 | 1 | 0 | 1 | 0 | 0 | 0.06 | 1 | 0.75 | 0.25 | -0.33 | 1 | 0 | 0 | 0.66 | 0.4 | 0 | 1 |
| SLC44A4 | -0.6 | 0.08 | 18 | -17 | -0.5 | 0.19 | 15 | -14 | -0.4 | 0.24 | 7.5 | -6.5 | -0.5 | 0.21 | 10.5 | -9.5 | -0.6 | 0.08 | 18 | -17 |
| SLIT2 | 0.04 | 1 | 0.83 | 0.16 | 0.175 | 1 | 0.41 | 0.58 | -0.5 | 1 | 1.66 | -0.66 | -0.025 | 1 | 1.11 | -0.11 | 0.04 | 1 | 0.83 | 0.16 |
| SMARCA2 | 0 | 1 | 0 | 0 | -0.5 | 1 | 0 | 0 | -0.25 | 1 | 0 | 0 | -1 | 0.2 | 0 | 0 | -0.5 | 0.16 | 0 | 0 |
| SMG6 | 0 | 1 | 0 | 0 | -1 | 1 | 0 | 0 | -1 | 1 | 0 | 0 | -1 | 1 | 0 | 0 | 0 | 1 | 0 | 0 |
| SORBS2 | -0.09 | 1 | 1.55 | -0.55 | -0.02 | 1 | 1.12 | -0.12 | 0.24 | 0.23 | 0.36 | 0.63 | 0.05 | 1 | 0.77 | 0.22 | -0.34 | 0.3 | 0.5 | 0.5 |
| STAT4 | 0 | ─ | 0 | 0 | 0 | ─ | 0 | 0 | 0 | ─ | 0 | 0 | 0 | ─ | 0 | 0 | 0 | ─ | 0 | 0 |
| STK39 | 0 | ─ | 0 | 0 | 0 | ─ | 0 | 0 | 0 | ─ | 0 | 0 | 0 | ─ | 0 | 0 | 0 | ─ | 0 | 0 |
| STX8 | -0.5 | 1 | 0 | 0 | -0.5 | 0.46 | 0 | 0 | -0.5 | 1 | 0 | 0 | 0.16 | 1 | 0.5 | 0.5 | -0.5 | 1 | 0 | 0 |
| SUMO4 | 0 | 1 | 0 | 0 | 0.5 | 1 | 0 | 1 | 0 | 1 | 0 | 0 | 0.33 | 1 | 0 | 1 | 0 | 1 | 0 | 0 |
| SUSD1 | -0.16 | 1 | 2 | -1 | -0.16 | 1 | 2 | -1 | -0.26 | 1 | 3 | -2 | -0.16 | 1 | 2 | -1 | -0.26 | 1 | 3 | -2 |
| TCF19 | 0.09 | 1 | 0.66 | 0.33 | -0.28 | 0.59 | 3.33 | -2.33 | 0.02 | 1 | 0.88 | 0.11 | -0.23 | 0.59 | 2.66 | -1.66 | -0.28 | 0.59 | 3.33 | -2.33 |
| TENM4(ODZ4) | -0.5 | 1 | 2.66 | 1 | -1 | 1 | 1.62 | 1 | 0.5 | 1 | 3 | 1 | -1 | 1 | 4.87 | 1 | -1 | 1 | 2.4 | 1 |
| TFCP2L1 | 0 | ─ | 0 | 0 | 0 | ─ | 0 | 0 | 0 | ─ | 0 | 0 | 0 | ─ | 0 | 0 | 0 | ─ | 0 | 0 |
| TLR4 | 0 | 1 | 0 | 0 | 0 | 1 | 0 | 0 | 0 | 1 | 0 | 0 | 0 | 1 | 0 | 0 | 0 | 1 | 0 | 0 |
| TMEM132B | -0.3 | 1 | 0.4 | 0.6 | -0.3 | 1 | 0.66 | 0.33 | 0 | 1 | 1 | 0 | -0.3 | 1 | 4 | -3 | -0.29 | 1 | 0.66 | 0.33 |
| TNFAIP3 | -0.66 | 0.4 | 0 | 0 | -1 | 0.33 | 0 | 0 | -1 | 0.1 | 0 | 0 | -1 | 0.33 | 0 | 0 | -1 | 0.33 | 0 | 0 |
| TNFalfa | -0.33 | 1 | 0 | 0 | -0.33 | 1 | 0 | 0 | -0.33 | 0.37 | 0 | 0 | -0.33 | 1 | 0 | 0 | -0.33 | 0.37 | 0 | 0 |
| TRIM31 | -0.16 | 1 | 1 | 0 | -0.5 | 1 | 0 | 0 | -0.42 | 1 | 0 | 0 | -0.4 | 1 | 0 | 0 | -0.5 | 1 | 0 | 0 |
| TTLL7 | 0 | ─ | 0 | 0 | 0 | ─ | 0 | 0 | 0 | ─ | 0 | 0 | 0 | ─ | 0 | 0 | 0 | ─ | 0 | 0 |
| UBAC2 | 0.58 | 0.19 | 0.06 | 0.93 | 0.33 | 0.46 | 0.2 | 0.8 | 0.33 | 0.46 | 0.2 | 0.8 | 0.16 | 1 | 0.4 | 0.6 | 0.16 | 1 | 0.4 | 0.6 |
| UBASH3B | -0.6 | 0.18 | 0 | 0 | -0.33 | 0.5 | 0 | 0 | -0.2 | 1 | 0 | 0.2 | -0.22 | 1 | 0 | 0 | -0.4 | 0.46 | 0 | 0 |
| UBD | 0 | ─ | 0 | 0 | 0 | ─ | 0 | 0 | 0 | ─ | 0 | 0 | 0 | ─ | 0 | 0 | 0 | ─ | 0 | 0 |
| ZNRD1 | 0 | ─ | 0 | 0 | 0 | ─ | 0 | 0 | 0 | ─ | 0 | 0 | 0 | ─ | 0 | 0 | 0 | ─ | 0 | 0 |

1. DoS: Direction of Selection
2. P value of 2x2 McDonald-Kreitman test
3. NI: Neutrality Index
4. Alpha value (proportion of adaptive substitutions)

**Supplemental Table 16.** Tissue specific expression profiles of BD associated genes with population genetic parameter and selection estimates unique to East Asians.

| **Gene** | **adrenal** | **appendix** | **bone marrow** | **brain** | **colon** | **duodenum** | **endometrium** | **esophagus** | **fat** | **gall bladder** | **heart** | **kidney** | **liver** | **lung** | **lymph node** | **ovary** | **pancreas** | **placenta** | **prostate** | **salivary gland** | **skin** | **small intestine** | **spleen** | **stomach** | **testis** | **thyroid** | **urinary bladder** |
| --- | --- | --- | --- | --- | --- | --- | --- | --- | --- | --- | --- | --- | --- | --- | --- | --- | --- | --- | --- | --- | --- | --- | --- | --- | --- | --- | --- |
| C6ORF10 | 0.0 | 0.0 | 0.0 | 0.0 | 0.0 | 0.0 | 0.0 | 0.0 | 0.0 | 0.0 | 0.0 | 0.0 | 0.0 | 0.0 | 0.0 | 0.0 | 0.0 | 0.0 | 0.0 | 0.0 | 0.0 | 0.0 | 0.0 | 0.0 | 6.3 | 0.0 | 0.0 |
| DTL | 0.2 | 2.8 | 8.0 | 0.4 | 1.6 | 1.2 | 0.8 | 1.1 | 0.4 | 0.5 | 0.1 | 0.1 | 0.6 | 1.3 | 3.2 | 0.1 | 0.0 | 2.7 | 0.3 | 0.0 | 1.0 | 1.7 | 1.6 | 0.9 | 9.7 | 0.4 | 1.2 |
| EBF2 | 0.4 | 0.5 | 0.0 | 0.0 | 0.1 | 0.0 | 0.2 | 0.5 | 8.5 | 1.9 | 4.4 | 0.3 | 0.0 | 0.3 | 0.4 | 0.2 | 0.1 | 0.2 | 0.5 | 0.3 | 0.1 | 0.1 | 0.0 | 0.0 | 0.5 | 2.9 | 1.0 |
| ERAP1 | 5.4 | 12.1 | 6.1 | 4.5 | 10.4 | 16.1 | 6.6 | 6.0 | 20.3 | 8.9 | 5.2 | 5.5 | 4.5 | 8.4 | 10.1 | 7.4 | 1.1 | 13.3 | 6.5 | 2.5 | 10.6 | 14.8 | 9.6 | 9.5 | 4.5 | 6.8 | 10.2 |
| FUT2 | 0.2 | 2.6 | 0.0 | 0.3 | 10.5 | 17.3 | 0.5 | 6.6 | 0.1 | 6.5 | 0.1 | 0.5 | 0.0 | 0.3 | 0.2 | 0.3 | 0.4 | 0.1 | 0.6 | 5.7 | 1.7 | 8.7 | 0.2 | 11.2 | 0.3 | 0.2 | 2.6 |
| GABBR1 | 7.1 | 5.1 | 5.6 | 71.2 | 2.1 | 1.9 | 13.0 | 3.3 | 5.4 | 6.9 | 4.3 | 2.5 | 0.5 | 6.7 | 5.4 | 6.8 | 0.6 | 3.7 | 9.0 | 2.0 | 9.1 | 3.1 | 14.5 | 2.6 | 3.5 | 4.4 | 4.1 |
| GALNT10 | 10.0 | 9.5 | 7.7 | 5.7 | 10.1 | 7.7 | 4.9 | 3.0 | 5.5 | 12.5 | 3.9 | 5.4 | 0.8 | 9.5 | 5.6 | 33.4 | 1.1 | 2.8 | 5.4 | 3.3 | 2.9 | 7.2 | 9.2 | 11.9 | 4.1 | 10.1 | 8.4 |
| HLA_F | 5.2 | 15.8 | 7.3 | 1.7 | 10.9 | 12.3 | 5.7 | 2.9 | 6.1 | 7.8 | 3.0 | 2.9 | 2.1 | 14.3 | 20.5 | 2.6 | 0.4 | 2.8 | 3.9 | 5.2 | 7.6 | 14.2 | 30.7 | 11.5 | 2.2 | 2.8 | 7.7 |
| HLA_G | 1.0 | 4.6 | 3.5 | 1.3 | 14.0 | 3.1 | 2.6 | 2.4 | 2.4 | 2.6 | 1.3 | 1.4 | 1.2 | 8.3 | 6.8 | 1.1 | 0.3 | 29.3 | 1.0 | 1.0 | 3.4 | 2.9 | 9.5 | 2.2 | 2.0 | 1.4 | 2.4 |
| HMP19 | 19.1 | 1.5 | 0.0 | 115.0 | 1.7 | 1.1 | 0.0 | 0.1 | 0.4 | 0.1 | 0.1 | 0.2 | 0.0 | 0.1 | 0.0 | 0.0 | 0.1 | 0.0 | 0.0 | 0.0 | 0.0 | 1.9 | 0.0 | 1.8 | 2.7 | 0.1 | 0.0 |
| HNF4G | 0.0 | 1.4 | 0.0 | 0.2 | 10.4 | 45.9 | 0.2 | 0.1 | 0.1 | 6.8 | 0.0 | 7.8 | 2.8 | 0.1 | 0.0 | 0.0 | 0.6 | 0.0 | 0.3 | 0.1 | 0.0 | 54.2 | 0.0 | 3.2 | 1.4 | 0.0 | 0.5 |
| IL1A | 0.0 | 0.5 | 0.2 | 0.5 | 0.1 | 0.0 | 1.9 | 4.6 | 0.0 | 1.1 | 0.0 | 0.0 | 0.0 | 0.8 | 0.1 | 0.0 | 0.0 | 0.5 | 0.0 | 0.0 | 0.2 | 0.0 | 0.1 | 0.0 | 5.2 | 0.1 | 2.1 |
| IL23R | 0.8 | 0.2 | 0.0 | 0.0 | 0.3 | 0.2 | 0.0 | 0.0 | 0.0 | 0.1 | 0.0 | 0.0 | 0.0 | 0.0 | 0.1 | 0.0 | 0.0 | 0.0 | 0.0 | 0.0 | 0.0 | 0.1 | 0.1 | 0.2 | 0.6 | 0.0 | 0.2 |
| IL6 | 2.9 | 6.8 | 4.9 | 0.5 | 0.3 | 0.3 | 0.9 | 5.3 | 1.9 | 7.8 | 0.9 | 0.3 | 1.4 | 4.3 | 1.4 | 0.0 | 0.6 | 1.5 | 2.4 | 0.0 | 0.0 | 0.2 | 0.8 | 0.2 | 0.2 | 0.2 | 10.7 |
| MOG | 0.0 | 0.0 | 0.0 | 52.1 | 0.0 | 0.0 | 0.0 | 0.0 | 0.0 | 0.0 | 0.5 | 0.0 | 0.0 | 0.0 | 0.0 | 0.0 | 0.0 | 0.0 | 0.0 | 0.0 | 0.0 | 0.0 | 0.0 | 0.0 | 0.0 | 0.0 | 0.0 |
| NOD2 | 0.1 | 2.4 | 3.2 | 0.1 | 0.3 | 0.4 | 0.2 | 2.2 | 0.1 | 0.6 | 0.1 | 0.1 | 0.1 | 1.0 | 1.0 | 0.1 | 0.0 | 0.4 | 0.2 | 0.5 | 2.3 | 0.3 | 1.1 | 0.4 | 0.3 | 0.1 | 0.7 |
| OSR1 | 0.5 | 1.3 | 0.0 | 0.2 | 2.2 | 0.5 | 5.3 | 1.3 | 2.8 | 3.1 | 2.6 | 0.3 | 0.0 | 2.8 | 0.2 | 1.1 | 0.2 | 1.8 | 3.1 | 5.4 | 1.1 | 0.9 | 0.1 | 0.9 | 3.1 | 0.5 | 4.4 |
| PPP1R11 | 24.2 | 32.3 | 18.0 | 40.2 | 35.4 | 43.8 | 39.6 | 24.8 | 21.2 | 36.4 | 25.8 | 30.3 | 19.0 | 37.4 | 31.1 | 31.3 | 8.3 | 31.7 | 35.7 | 18.5 | 19.0 | 46.0 | 28.0 | 35.2 | 30.5 | 45.2 | 32.1 |
| PSOR1C1 (C6orf16) | 0.0 | 0.1 | 0.0 | 0.1 | 0.5 | 0.4 | 0.1 | 0.1 | 0.1 | 0.3 | 0.1 | 0.1 | 0.0 | 0.3 | 0.0 | 0.1 | 0.0 | 0.3 | 0.1 | 0.0 | 0.9 | 0.4 | 0.0 | 0.7 | 11.6 | 0.1 | 0.1 |
| RALGAPA2 | 1.5 | 2.5 | 5.1 | 1.4 | 4.9 | 3.3 | 2.8 | 2.2 | 5.6 | 5.1 | 1.4 | 3.3 | 2.8 | 5.7 | 2.2 | 1.8 | 0.9 | 6.4 | 3.8 | 2.2 | 4.2 | 3.4 | 1.4 | 5.3 | 3.6 | 9.0 | 5.6 |
| SLC41A2 | 2.1 | 1.8 | 0.0 | 1.8 | 4.1 | 5.7 | 0.8 | 0.4 | 1.6 | 3.4 | 0.3 | 1.5 | 11.2 | 1.1 | 0.8 | 1.0 | 0.7 | 0.8 | 0.7 | 1.1 | 0.2 | 5.0 | 0.6 | 5.6 | 1.9 | 2.5 | 0.9 |
| SLC44A4 | 3.2 | 12.9 | 0.6 | 0.1 | 98.0 | 60.1 | 5.8 | 0.8 | 0.4 | 44.7 | 0.0 | 19.9 | 0.0 | 11.6 | 0.2 | 1.3 | 1.3 | 3.1 | 69.0 | 1.8 | 0.1 | 48.0 | 1.4 | 72.0 | 0.3 | 0.6 | 20.9 |
| SMARCA2 | 17.2 | 13.0 | 8.9 | 24.7 | 12.5 | 6.4 | 16.1 | 12.2 | 22.7 | 17.3 | 9.9 | 17.8 | 12.9 | 17.3 | 16.2 | 42.3 | 2.7 | 18.6 | 16.6 | 7.4 | 17.3 | 9.2 | 16.2 | 10.0 | 29.1 | 21.8 | 13.6 |
| STX8 | 8.7 | 5.3 | 6.2 | 7.2 | 6.1 | 4.8 | 6.4 | 5.6 | 9.6 | 6.8 | 6.9 | 7.0 | 5.2 | 5.4 | 6.4 | 7.1 | 2.1 | 6.0 | 7.0 | 4.3 | 5.1 | 5.2 | 6.1 | 5.4 | 14.5 | 9.4 | 8.2 |
| TFCP2L1 | 0.2 | 1.0 | 0.1 | 0.5 | 11.8 | 0.3 | 0.3 | 4.7 | 0.1 | 2.0 | 0.0 | 25.0 | 0.0 | 2.0 | 0.1 | 0.0 | 0.8 | 5.0 | 3.2 | 24.0 | 2.5 | 0.2 | 0.6 | 5.2 | 0.9 | 17.5 | 1.5 |
| TTLL7 | 1.6 | 0.9 | 0.0 | 17.7 | 1.1 | 1.1 | 2.3 | 2.1 | 2.7 | 3.0 | 1.2 | 0.4 | 0.4 | 2.3 | 0.3 | 0.5 | 0.6 | 0.1 | 3.5 | 0.8 | 0.3 | 1.2 | 0.4 | 1.2 | 2.9 | 1.4 | 2.1 |
| ZNRD1 | 6.5 | 10.8 | 7.5 | 3.2 | 8.4 | 8.6 | 8.5 | 6.6 | 5.1 | 7.2 | 4.3 | 6.4 | 3.5 | 6.3 | 12.3 | 8.0 | 1.6 | 5.9 | 6.5 | 3.0 | 5.9 | 7.9 | 8.8 | 7.8 | 9.6 | 5.3 | 8.7 |

**Supplemental Table 17.** Genes with genetic diversity, allele frequency spectrum, divergence, and selection profiles unique to East Asians.

| **Gene** | **AFR_EAS**  **Dxy** | **AFR_EAS**  **Hst** | **AFR_EAS**  **Fst** | **Pi/Fst** | **Pi/Dxy** | **HD** | **θk** | **Pi** | **θW** | **TD** | **FLD** | **FLF** | **XP-EHH CHB-CEU** | **XP-EHH CHB-YRI** | **iHS^a^** | **DoS** | **Tissue of Major Expression** |
| --- | --- | --- | --- | --- | --- | --- | --- | --- | --- | --- | --- | --- | --- | --- | --- | --- | --- |
| DTL | 0.000599 | 0.009907 | 0.090997 | 0.004396 | 0.667487 | 0.97 | 30.97 | 0.0004 | 64.73 | -1.52 | -9.65 | -5.5 | 0.35 | 0.43 | 2.29 | 0 | testis,bone marrow |
| EBF2 | 0.001048 | 0.000128 | 0.217973 | 0.003211 | 0.667933 | 1 | 135.43 | 0.0007 | 217.42 | -1.11 | -10.45 | -5.3 | 0.94 | 1.63 | 2.40 | 1 | fat, heart |
| ERAP1 | 0.001211 | 0.005824 | 0.038666 | 0.028448 | 0.908136 | 0.97 | 57.39 | 0.0011 | 58.06 | -0.03 | -5.62 | -2.64 | 0.38 | 0.54 | 1.25 | -0.35 | fat, duodenum, small intestine |
| FUT2 | 0.001902 | 0.041069 | 0.359038 | 0.001114 | 0.210317 | 0.88 | 4.26 | 0.0004 | 13.75 | -1.93 | -6.39 | -4.8 | 0.23 | 0.89 | 1.00 | -0.5 | duodenum, colon, stomach, small intestine |
| GABBR1 | 0.000756 | 0.013101 | 0.028219 | 0.02835 | 1.057804 | 0.94 | 25.63 | 0.0008 | 36.44 | -0.86 | -4.06 | -2.56 | 0.12 | 0.16 | 0.95 | -0.2 | brain, spleen, endometrium |
| GALNT10 | 0.001374 | 9.66E-05 | 0.280904 | 0.002492 | 0.509455 | 1 | 162.96 | 0.0007 | 250.25 | -1.03 | -9.56 | -4.83 | 1.23 | 1.18 | 2.09 | 1 | ubiquitous |
| HLA-F | 0.002536 | 0.0302 | 0.042484 | 0.051785 | 0.867652 | 0.81 | 8.47 | 0.0022 | 6.94 | 0.59 | -2.66 | -1.41 | 0.10 | 0.40 | 0.86 | -0.14 | spleen, lymph node,appendix, small intestine |
| HLA-G | 0.006016 | 0.042407 | 0.031991 | 0.171924 | 0.914281 | 0.8 | 22.6 | 0.0055 | 13.21 | 1.98 | -1.66 | 0.34 | 0.03 | 0.63 | 2.43 | 0.37 | placenta, colon, spleen |
| HMP19 | 0.000663 | 0.00705 | 0.291472 | 0.000686 | 0.301583 | 0.98 | 9.99 | 0.0002 | 60.33 | -2.43 | -10.29 | -6.44 | 0.06 | 1.10 | 1.97 | 0.52 | brain, adrenal, small intestine |
| HNF4G | 0.001358 | 0.035201 | 0.329219 | 0.002734 | 0.662785 | 0.88 | 25.21 | 0.0009 | 31.23 | -0.55 | -9.89 | -5.3 | 0.55 | 0.59 | 1.18 | 1 | small intestine, duodenum, colon |
| IL1A | 0.001228 | 0.110406 | 0.331057 | 0.002114 | 0.569922 | 0.57 | 8.09 | 0.0007 | 9.61 | -0.43 | -6.45 | -4.1 | 0.58 | 1.60 | 1.05 |  | testis, esophagus, endometrium |
| IL23R | 0.000944 | 0.001069 | 0.153596 | 0.003906 | 0.635414 | 1 | 74.9 | 0.0006 | 113.85 | -1 | -11.43 | -5.77 | 0.16 | 1.24 | 1.40 | 0 | adrenal, testis, colon |
| IL6 | 0.00093 | 0.124863 | 0.315357 | 0.000951 | 0.322558 | 0.5 | 1.53 | 0.0003 | 6.94 | -2.07 | -4.63 | -4.1 | 0.33 | 1.04 | 1.70 | 0 | urinary bladder, gall bladder |
| MOG | 0.001183 | 0.028672 | 0.111341 | 0.005389 | 0.507002 | 0.86 | 9.16 | 0.0006 | 24.69 | -1.8 | -8.51 | -5.57 |  |  |  | 0 | brain |
| NOD2 | 0.000575 | 0.031204 | 0.142952 | 0.002099 | 0.521379 | 0.88 | 12.18 | 0.0003 | 42.84 | -2.07 | -11.73 | -7.04 | 0.75 | 1.65 | 3.13 | -0.02 | bone marrow, appendix,skin, esophagus |
| OSR1 | 0.00035 | 0.170349 | 0.319798 | 0.000313 | 0.285959 | 0.44 | 0.48 | 0.0001 | 4.4 | -2.25 | -9.61 | -7.66 | ─ | ─ | ─ | 0 | endometrium, salivary gland, urinary bladder |
| PPP1R11 | 0.000916 | 0.068041 | 0.068565 | 0.010209 | 0.763887 | 0.62 | 2.16 | 0.0007 | 4.4 | -1.29 | -1.38 | -1.65 | ─ | ─ | ─ | 0 | ubiquitous |
| PSORS1C1 | 0.00404 | 0.013194 | 0.03885 | 0.102961 | 0.990202 | 0.97 | 101.02 | 0.004 | 75.68 | 0.98 | -0.76 | 0.33 | 0.07 | 0.09 | 0.73 | -0.17 | testis, skin, small intestine, stomach |
| RALGAPA2 | 0.000411 | 7.59E-05 | 0.054594 | 0.007327 | 0.97325 | 1 | 113.86 | 0.0004 | 260.66 | -1.66 | -13.06 | -6.78 | 0.28 | 0.52 | 2.84 | -0.16 | thyroid |
| RNF39 | 0.002171 | 0.042686 | 0.044244 | 0.042943 | 0.874986 | 0.84 | 10.65 | 0.0019 | 11.88 | -0.29 | -1.53 | -1.06 | ─ | ─ |  | 0 | ubiquitous |
| SLC41A2 | 0.000833 | 0.001353 | 0.058252 | 0.013733 | 0.959934 | 1 | 120.88 | 0.0008 | 148.28 | -0.54 | -10.17 | -4.84 | 0.37 | 0.60 | 1.42 | 0.5 | liver, duodenum, stomach, small intestine, colon |
| SLC44A4 | 0.001149 | 0.022032 | 0.041402 | 0.026569 | 0.957055 | 0.94 | 16.72 | 0.0011 | 20.82 | -0.56 | -4.38 | -2.67 | 0.47 | 1.01 | 0.74 | -0.6 | colon, stomach, prostate, duodenum |
| SMARCA2 | 0.001359 | 9.86E-06 | 0.131913 | 0.007581 | 0.735946 | 1 | 177.36 | 0.001 | 241.44 | -0.78 | -11.9 | -5.68 | 0.11 | 0.58 | 1.43 | -0.5 | ovary, testis, brain, fat |
| STX8 | 0.001228 | 8.44E-07 | 0.180507 | 0.003878 | 0.570187 | 1 | 217.81 | 0.0007 | 376.38 | -1.24 | -11.57 | -5.81 | 0.69 | 0.96 | 1.79 | -0.5 | ubiquitous |
| TFCP2L1 | 0.000921 | 0.00132 | 0.080501 | 0.008696 | 0.759914 | 0.33 | 49.23 | 0.0007 | 71 | -0.89 | -10.39 | -5.38 | 0.58 | 0.58 | 1.29 | 0 | kidney, salivary gland, thyroid, colon |
| TSBP1 | 0.002838 | 0.004326 | 0.067565 | 0.039961 | 0.95149 | 0.99 | 215.55 | 0.0027 | 144.55 | 1.44 | -0.19 | 0.93 | ─ | ─ | ─ | 0 | testis |
| TTLL7 | 0.000539 | 0.002317 | 0.03903 | 0.010248 | 0.741978 | 0.99 | 56.74 | 0.0004 | 116.65 | -1.51 | -9.93 | -5.45 | 0.30 | 0.39 | 1.11 | 0 | ubiquitous |
| ZNRD1 | 0.001421 | 0.030067 | 0.043799 | 0.027398 | 0.844585 | 0.81 | 4.43 | 0.0012 | 7.07 | -1 | -0.67 | -1 | ─ | ─ | ─ | ─ | ubiquitous |
| GAS2 | 0.001368 | 0.00037 | 0.195045 | 0.004614 | 0.65786 | 1 | 129.32 | 0.0009 | 154.69 | -0.48 | -9.7 | -4.59 | 0.41 | 1.95 | 1.76 | 0 | Liver, small intestine, stomach |
| LYST | 0.000832 | 0.000577 | 0.401948 | 0.000498 | 0.240282 | 1 | 38.18 | 0.0002 | 167.77 | -2.27 | -13.74 | -7.58 | 1.18 | 2.10 | 2.06 | 0 | Bone marrow, spleen, lymph node |
| NAV2 | 0.001279 | 0 | 0.161659 | 0.005567 | 0.703646 | 1 | 664.45 | 0.0009 | 875.55 | -0.71 | -10.54 | -4.95 | 0.27 | 2.00 | 2.25 | -0.41 | ubiquitous |
| CTNNA2 | 0.001158 | 0 | 0.136864 | 0.005845 | 0.690893 | 1 | 955.57 | 0.0008 | 1298.64 | -0.78 | -9.7 | -4.63 | 0.79 | 1.59 | 2.47 | 0 | Brain |
| KCNK9 | 0.00116 | 0.000199 | 0.125034 | 0.006398 | 0.689391 | 1 | 82.86 | 0.0008 | 123.99 | -0.97 | -12.62 | -6.26 | 0.49 | 1.40 | 3.46 | 0 | Brain, adrenal, Duodenum |
| SEMA6D | 0.001035 | 3.82E-06 | 0.158692 | 0.005041 | 0.772743 | 1 | 471.63 | 0.0008 | 623.29 | -0.72 | -10.41 | -4.91 | 0.10 | 1.25 | 2.08 | -0.17 | Small intestine, duodenum |
| UBAC2 | 0.000661 | 0.000535 | 0.099261 | 0.006045 | 0.908019 | 1 | 110.54 | 0.0006 | 187.66 | -1.21 | -10.66 | -5.47 | 0.11 | 0.41 | 1.94 | 0.33 | ubiquitous |
| UBASH3B | 0.001166 | 5.42E-05 | 0.124635 | 0.007221 | 0.771652 | 1 | 141.49 | 0.0009 | 176.71 | -0.59 | -8.72 | -4.21 | 0.60 | 1.24 | 2.05 | -0.2 | spleen, lymph node,bone marrow |

HD: Haplotype diversity; θ_k_:Theta-K; θ_W_:Theta-Watterson; TD:Tajima's D; FLD: Fu-Li’s D; FLF: Fu-Li’s F; Fst: fixation index, Dxy: average number of nucleotide substitutions per site between two populations; Hst: haplotype diversity based differentiation; XP-EHH: Cross population extended haplotype homozygosity; iHS: Integrated haplotype score; DoS: Direction of Selection

1. Highest iHS value is reported.
