# Supplementary material for: Diverse selection pressures shaping the genetic architecture of behçet disease susceptibility
Source: Front Genet. 2022 Sep 30;13:983646. doi: 10.3389/fgene.2022.983646 (PMC9561091; doi:10.3389/fgene.2022.983646)

Supplementary Figure Legends

Supplementary Figure 1.
Comparison of F_{st} (population differentiation) estimates of ancestral and derived BD associated alleles between East Asian (EAS) and African (AFR) populations.

Supplementary Figure 2.
(A) Histogram of BD associated SNPs' allele frequencies among African, East Asian, and European populations. (B) Plot of BD associated variants' allele frequency in East Asian populations versus the rank of BD association test P-values reported.

Supplementary Figure 3.
Regression analyses between the reported P-values of variants' association with BD and (A) Allele frequency difference between East Asian and African populations, (B) Allele frequency difference between East Asians and Europeans.
Thick black lines on the figures represent regression lines and confidence intervals derived from the linear regression analyses.

Supplementary Figure 4.
Regression analyses between the reported Odds Ratios (OR) of variants' association with BD, and (A) Allele frequency difference between East Asians and Europeans, (B) Allele frequency difference between East Asian and African populations, (C) Population differentiation (F_{st}) between East Asian and European populations, (D) Population differentiation (F_{st}) between East Asian and African populations.
Thick black lines on the figures represent regression lines and confidence intervals derived from the linear regression analyses.

Supplementary Figure 5.
Population genetic parameters significantly different among the African (AFR), East Asian (EAS), and European (EUR) populations. Only significant differences are indicated by black arrows. Significance levels: $*P < 0.05$; $**P < 0.01$
Pi: Nucleotide diversity; Hap: Number of haplotypes; FuLiD: Fu and Li's D statistic; FuLiF: Fu and Li's F statistic; F_{st}: Population differentiation; TajimaD: Tajima's D statistic.

Supplementary Figure 6.
(A) Cumulative gene expression (reported as RPKM (reads per kilobase per million reads)) profile of BD associated genes among 27 human tissues. The inlet box plot shows the distribution of cumulative gene expression observed in all tissues. (B) Gene expression profiles for genes with population genetic parameter estimates and selection profiles unique to East Asians among 27 human tissues.

Supplemental Figure 1

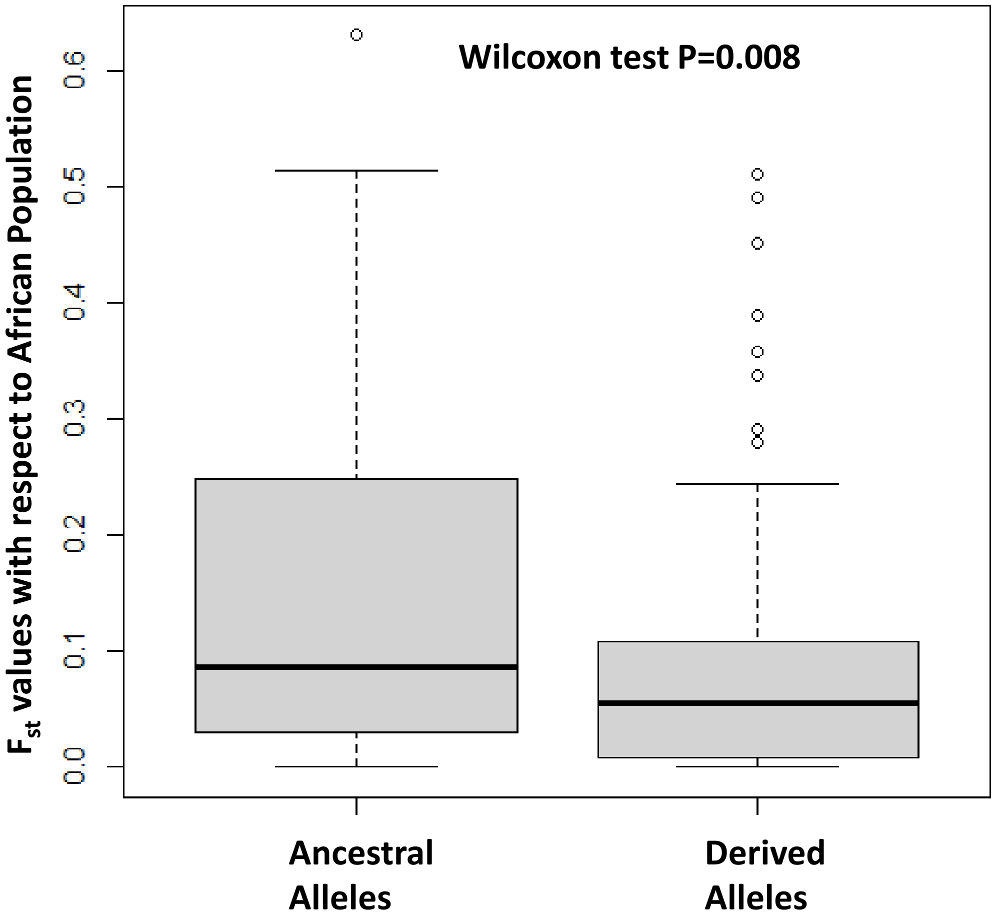

Supplemental Figure 2

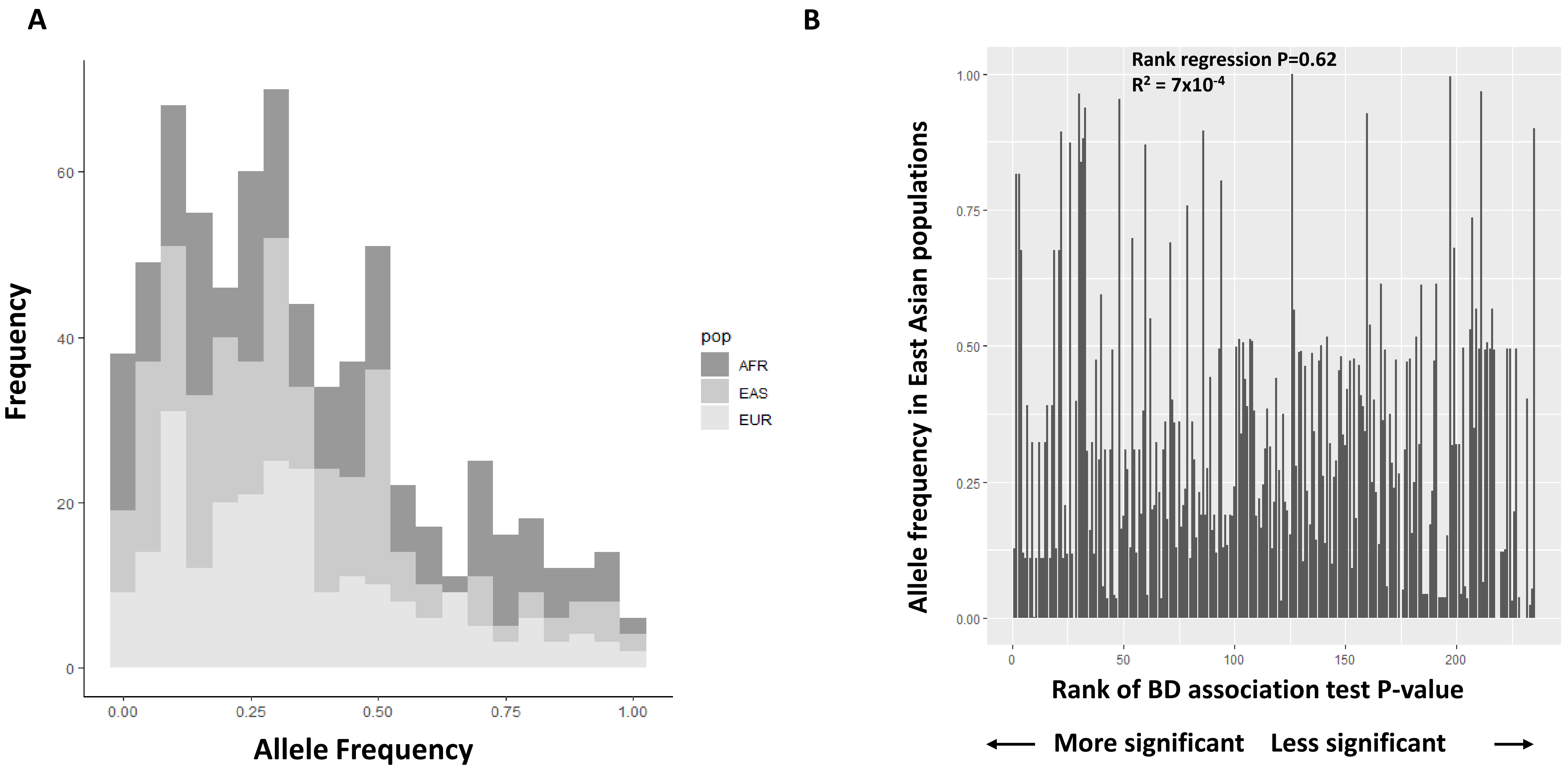

Supplemental Figure 3

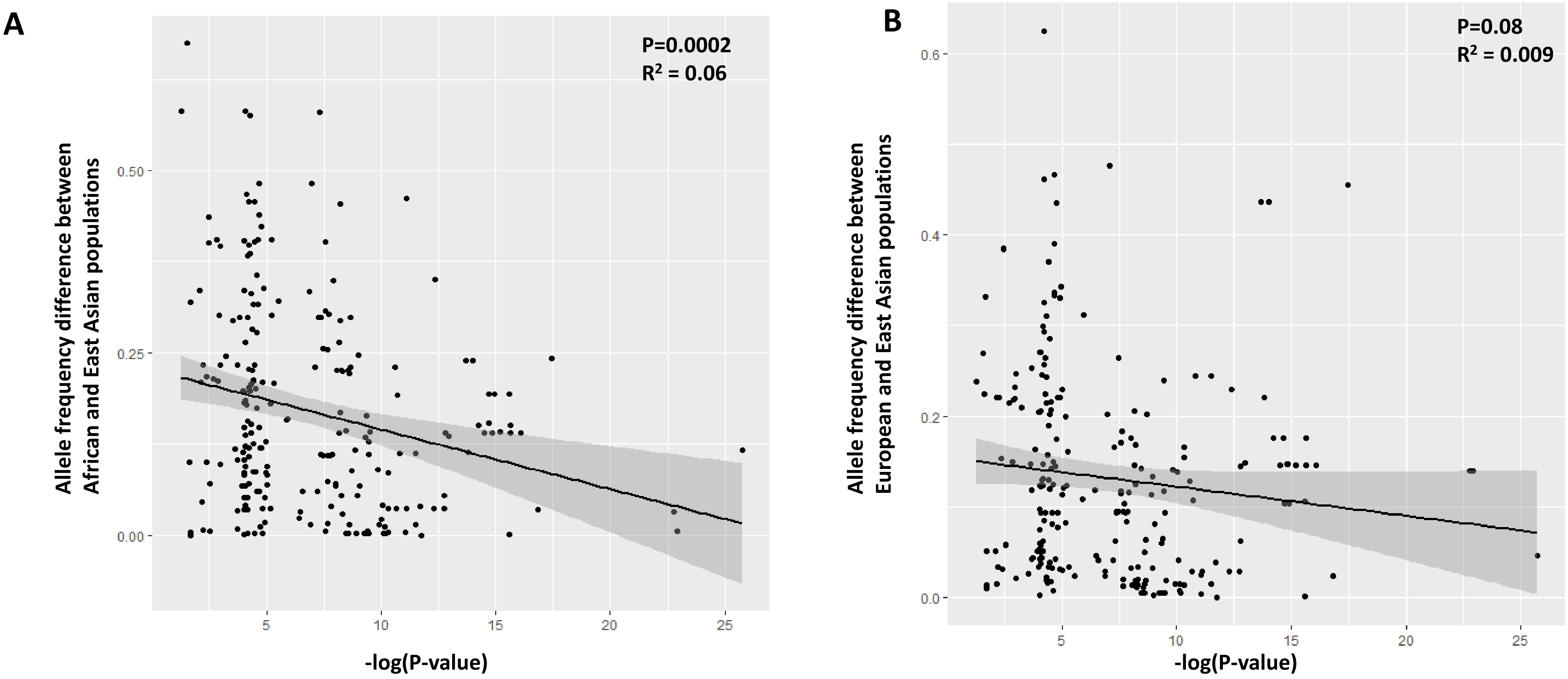

Supplemental Figure 4

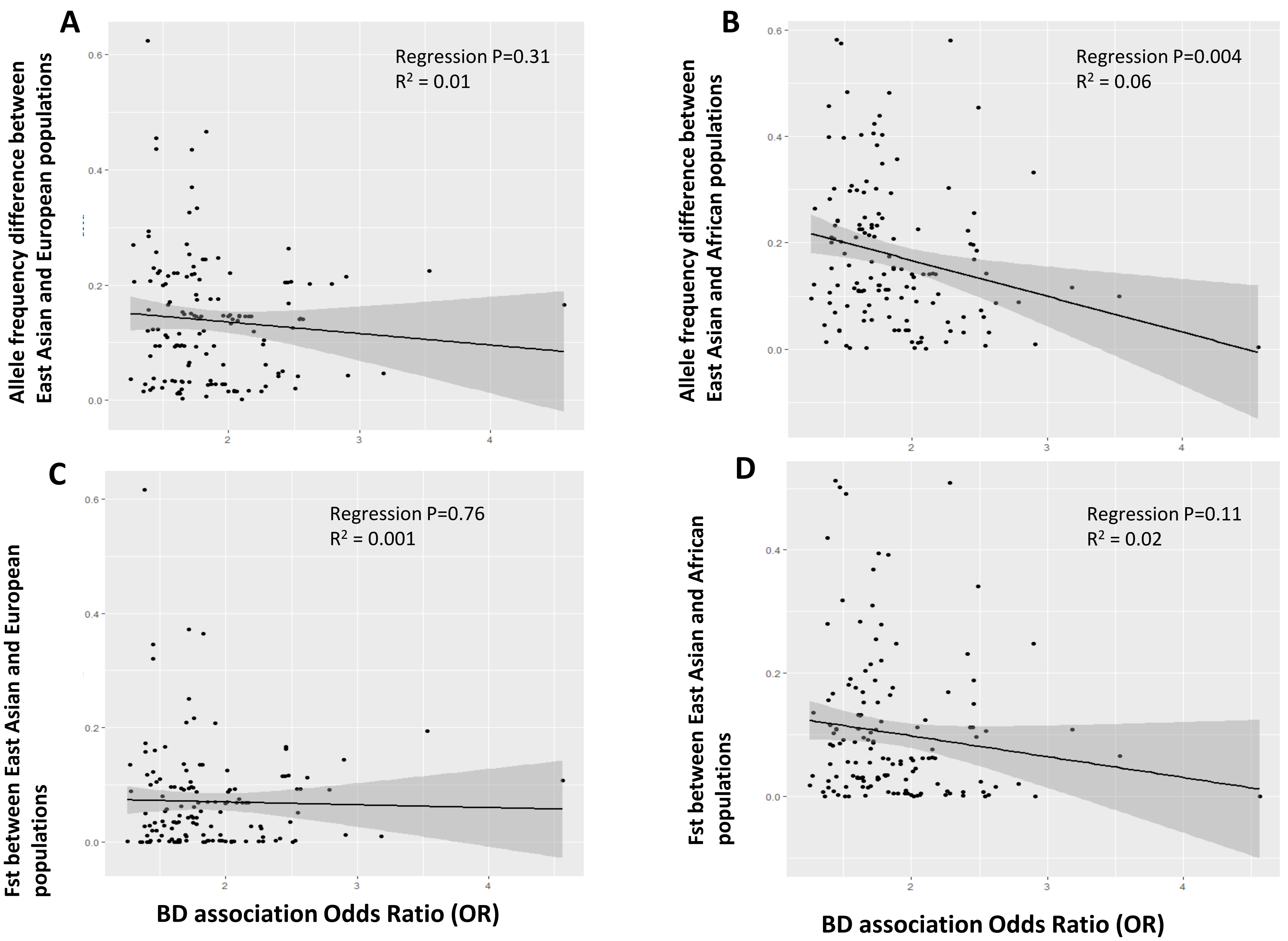

Supplemental Figure 5

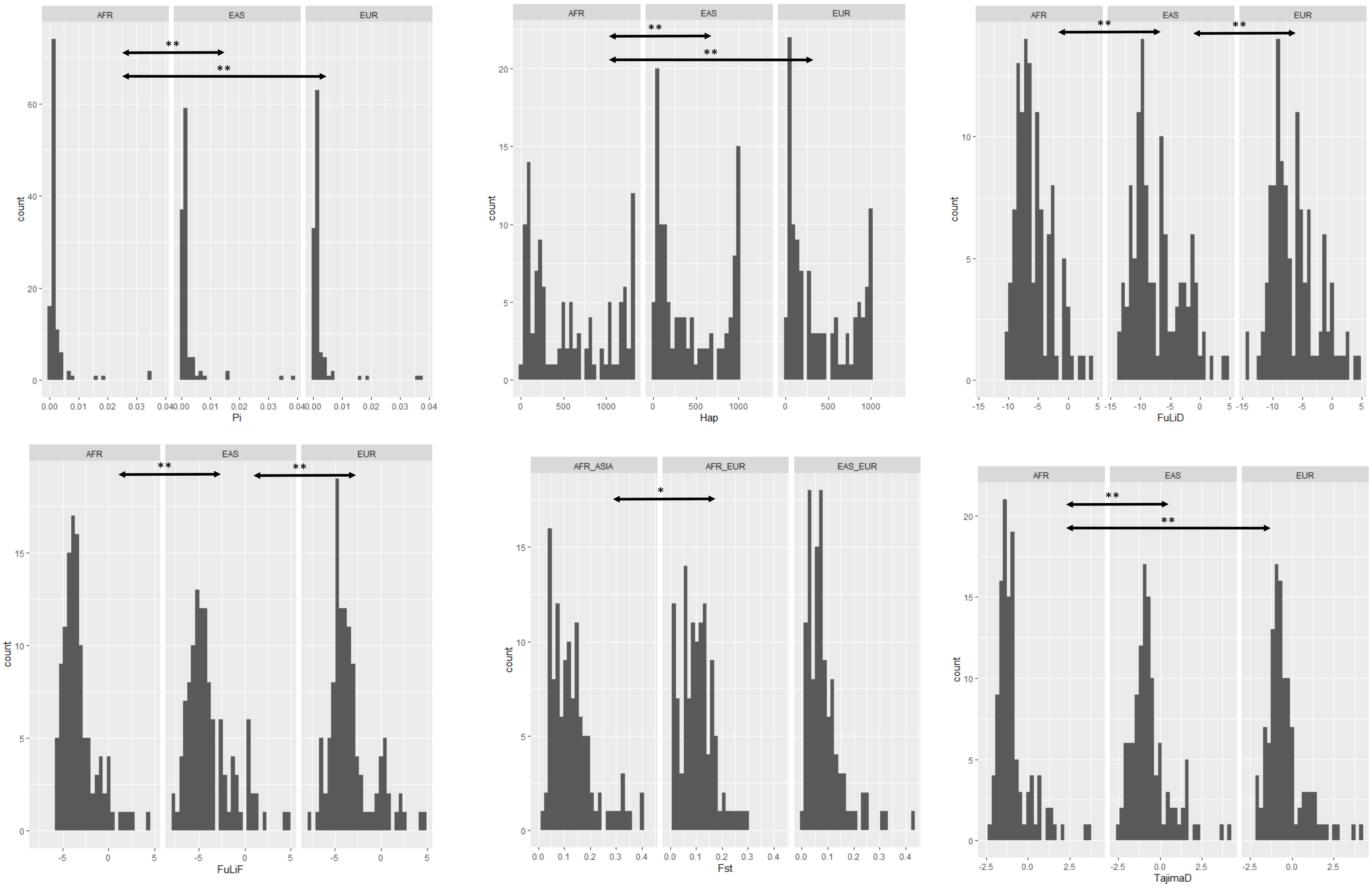

Supplemental Figure 6

A

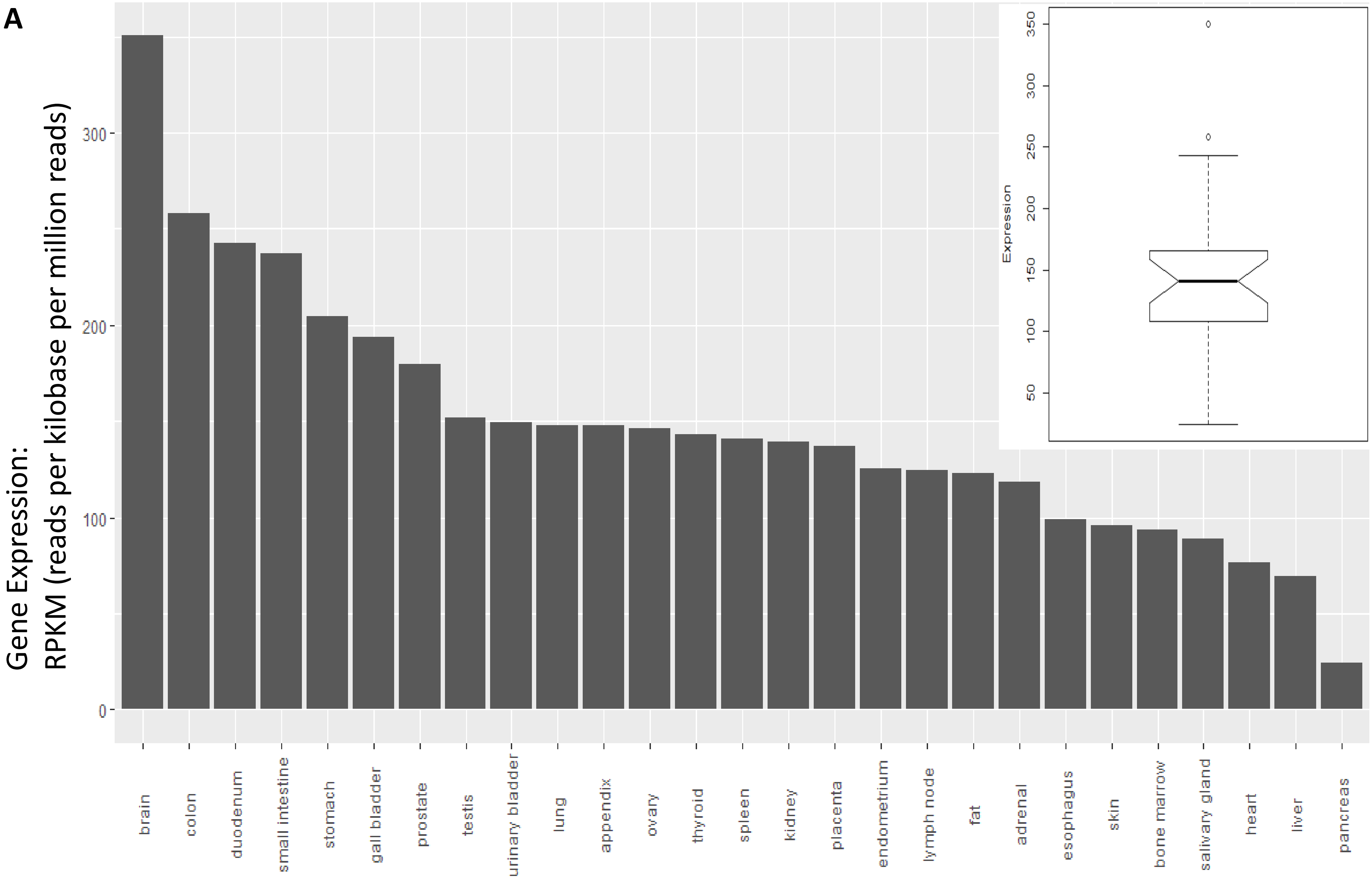

Supplemental Figure 6

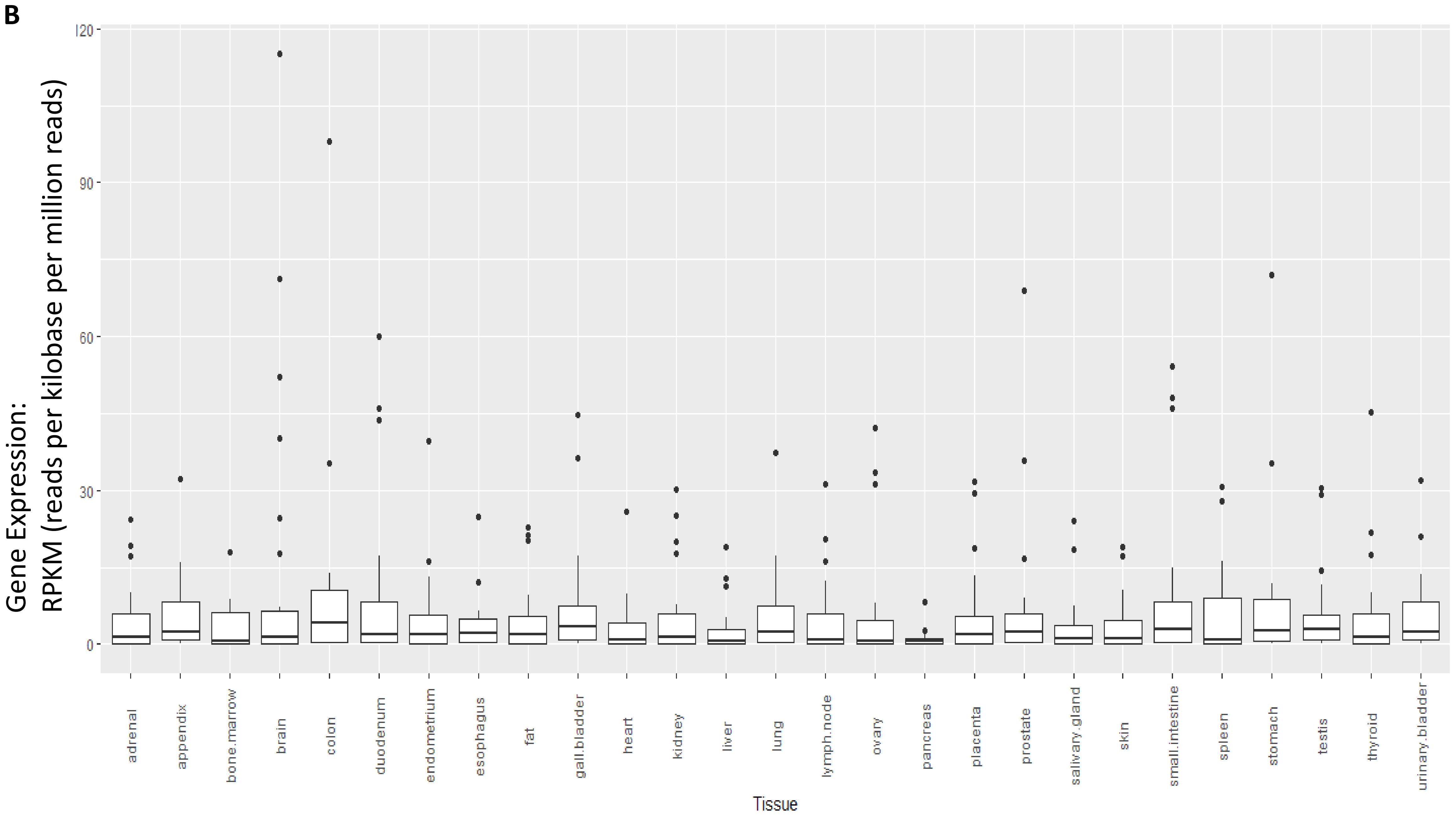

Supplement: Supplementary file 2 [file DataSheet2.zip › SupplementaryFigures.pdf]
